# What can NMR spectroscopy of selenoureas and phosphinidenes teach us about the π-accepting abilities of *N*-heterocyclic carbenes?†
†Electronic supplementary information (ESI) available: Full characterisation data including NMR spectra for new compounds; full DFT data and co-ordinates from computational studies. CCDC 1024807–1024818. For ESI and crystallographic data in CIF or other electronic format see DOI: 10.1039/c4sc03264k



**DOI:** 10.1039/c4sc03264k

**Published:** 2015-02-16

**Authors:** Sai V. C. Vummaleti, David J. Nelson, Albert Poater, Adrián Gómez-Suárez, David B. Cordes, Alexandra M. Z. Slawin, Steven P. Nolan, Luigi Cavallo

**Affiliations:** a KAUST Catalyst Center , Physical Sciences and Engineering Division , King Abdullah University of Science and Technology , Thuwal , 23955-6900 , Saudi Arabia . Email: luigi.cavallo@kaust.edu.sa; b EaStCHEM School of Chemistry , University of St Andrews , Purdie Building, North Haugh , St Andrews , Fife KY16 9ST , UK . Email: snolan@st-andrews.ac.uk; c WestCHEM Department of Pure and Applied Chemistry , University of Strathclyde , Thomas Graham Building, 295 Cathedral Street , Glasgow , G1 1XL , UK; d Institut de Química Computacional i Catàlisi and Department de Química , Universitat de Girona , Campus de Motilivi , E-17071 , Girona , Spain

## Abstract

The electronic nature of the interaction of NHCs with metal centres is of interest when exploring their properties, how these properties influence those of metal complexes, and how these properties might depend on ligand structure. Selenourea and phosphinidene complexes have been proposed to allow the measurement of the π-accepting ability of NHCs, independent of their σ-donating ability, *via* the collection of ^77^Se or ^31^P NMR spectra, respectively. Herein, the synthesis and characterisation of selenoureas derived from a range of imidazol-2-ylidenes, 4,5-dihydroimidazol-2-ylidenes and triazol-2-ylidenes are documented. Computational studies are used to explore the link between the shielding of the selenium centre and the electronic properties of the NHCs. Results show that *δ*_Se_ is correlated to the energy gap between a filled lone pair orbital on Se and the empty π* orbital corresponding to the Se–NHC bond. Bond energy decomposition analysis indicated no correlation between the orbital σ-contribution to bonding and the chemical shielding, while a good correlation was found between the π-contribution to bonding and the chemical shielding, confirming that this technique is indeed able to quantify the ability of NHCs to accept π-electron density. Calculations conducted on phosphinidene adducts yielded similar results. With the link between *δ*_Se_ and *δ*_P_ and π-back bonding ability clearly established, these compounds represent useful ways in which to fully understand and quantify this aspect of the electronic properties of NHCs.

## Abstract 2

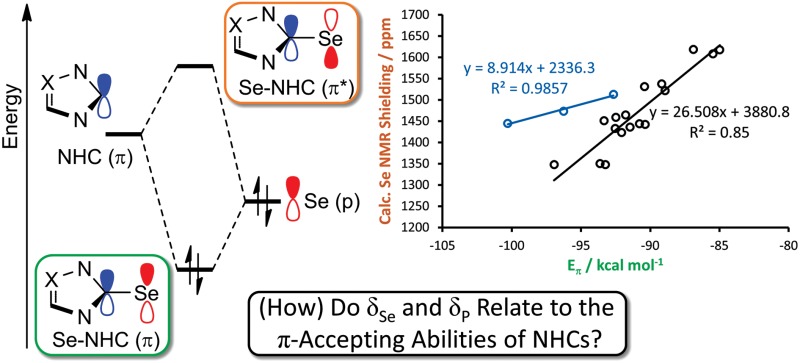
The relationship between the NMR chemical shifts of phosphinidene and selenourea compounds and the π-accepting ability of the related carbene ligands has been investigated.

## Introduction

The use of *N*-heterocyclic carbenes is now commonplace in a variety of fields of chemistry, including organometallic and main group synthesis, and catalysis.^1–5^ The exploration and quantification of their properties, *via* methods such as the Tolman Electronic Parameter (TEP)^6,7^ and Percent Buried Volume (%*V*_bur_)^8,9^ have allowed the electronic and steric properties of these exciting species to be elucidated and compared.^10,11^ The TEP is the most commonly used probe of the electronic properties of NHCs, and is based on the fact that the C–O bond in a metal carbonyl complex is weakened by d → π*_CO_ back-bonding; the frequency at which the C–O bond vibrates in the infra-red spectrum is therefore correlated to how electron-rich the metal centre is. Classically, [Ni(CO)_3_L] complexes have been used, although [MCl(CO)_2_L] (M = Rh or Ir)^12,13^ complexes provide less toxic alternatives.

NHCs‡
‡Trivial names and chemical names for the NHCs discussed herein: IPr, 1,3-bis(2,6-diisopropylphenyl)imidazol-2-ylidene; IPr^OMe^, 1,3-bis(2,6-diisopropyl-4-methoxyphenyl)imidazol-2-ylidene; SIPr, 1,3,-bis(2,6-diisopropylphenyl)-4,5-dihydroimidazol-2-ylidene; SIPr^OMe^, 1,3-bis(2,6-diisopropyl-4-methoxyphenyl)-4,5-dihydroimidazol-2-ylidene; IPr^Cl^, 1,3-bis(2,6-diisopropylphenyl)-4,5-dichloroimidazol-2-ylidene; IPr*, 1,3-bis(2,6-diphenylmethyl-4-methylphenyl)imidazol-2-ylidene; IPr*, 1,3-bis(2,6-diphenylmethyl-4-methoxyphenyl)imidazol-2-ylidene; I^i^Pr^Me^, 1,3-diisopropyl-4,5-dimethylimidazol-2-ylidene; IMes, 1,3-bis(2,4,6-trimethylphenyl)imidazol-2-ylidene; SIMes, 1,3-bis(2,4,6-trimethylphenyl)-4,5-dihydroimidazol-2-ylidene; IDD, 1,3-dicyclododecylimidazol-2-ylidene; SIDD, 1,3-dicyclododecyl-4,5-dihydroimidazol-2-ylidene; I^*t*^Bu, 1,3-di-*tert*-butylimidazol-2-ylidene; IMe, 1,3-bis(2,6-dimethylphenyl)imidazol-2-ylidene; IPent, 1,3-bis(2,6-diisopentylphenyl)imidazol-2-ylidene; IHept, 1,3-bis(2,6-diisoheptylphenyl)imidazol-2-ylidene; INon, 1,3-bis(2,6-diisononylphenyl)imidazol-2-ylidene; IAd, 1,3-diadamantylimidazol-2-ylidene; ICy, 1,3-dicyclohexylimidazol-2-ylidene; ITME, 1,3,4,5-tetramethylimidazol-2-ylidene; BI^i^Pr, 1,3-diisopropylbenzimidazol-2-ylidene; I^i^Pr, 1,3-diisopropylimidazol-2-ylidene; CAC-Mes, 1,3-dimesityl-5,5-dimethyl-4-oxo-3,4,5,6-tetrahydropyrimid-2-ylidene; 6-IPr, 1,3-bis(2,6-diisopropylphenyl)-3,4,5,6-tetrahydropyrimid-2-ylidene; ThIPr, 3-(2,6-diisopropylphenyl)-4,5,6,7-tetrahydrobenzothiazol-2-ylidene; CAAC-IPr, 1′-(2,6-diisopropylphenyl)-1′,2′,4′,5′,6′,7′-hexahydrospiro[cyclohexane-1,3′-indol-2-ylidene]. were originally believed to be purely σ-donors, with negligible contributions from π-bonding. Subsequent studies have established that NHCs can accept electron density *via* π-back donation to an extent that cannot be neglected when considering their electronic properties.^14–16^ In some cases, NHCs can even function as π-donors.^17^ As the TEP requires that all ligands (L) being compared have a similar degree of π-accepting ability (it reflects the net electron density at the metal centre) it is not merely an indicator of σ-donating ability. For example, 4,5-dihydroimidazol-2-ylidenes appear to be less electron-donating than the equivalent imidazol-2-ylidenes (from the TEP),^7^ yet in reality they are more σ-donating, but also more π-accepting.^18^

**Fig. 1:**
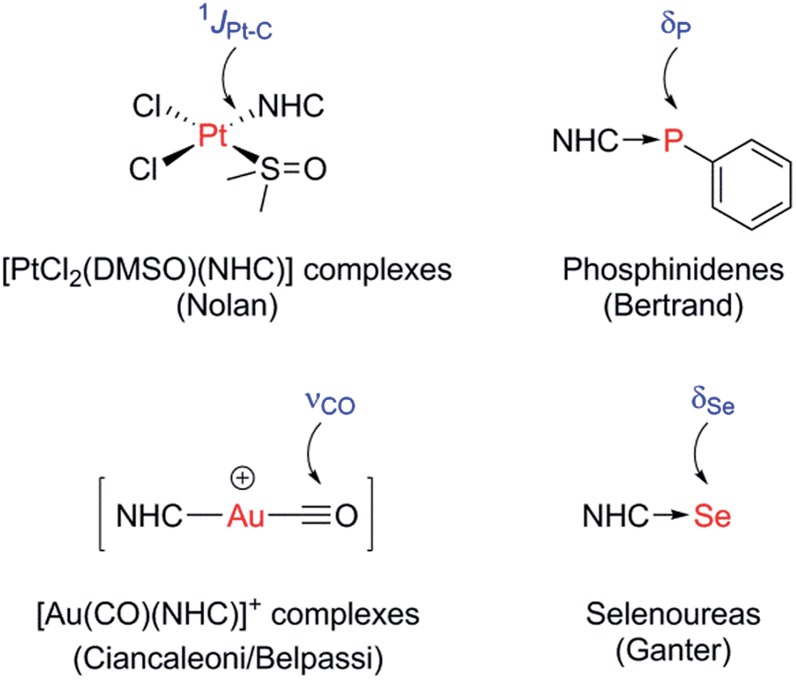
Methods used to quantify the electronic properties of carbenes.

A number of methods to assess the π-accepting ability of NHCs have been proposed (Fig. 1). Nolan demonstrated the use of ^1^ *J*_Pt–C_ coupling constants in [PtCl_2_(DMSO)(NHC)] complexes, prepared in one step from the free NHC and [PtCl_2_(DMSO)_2_].^18^ Bertrand used ^31^P NMR spectroscopy of phosphinidene adducts, which were prepared from the reaction of the free NHC with PPhCl_2_, followed by reduction with KC_8_ or Mg.^19^ Ganter utilised ^77^Se NMR spectroscopy of selenourea compounds, which were synthesised from the reaction of the imidazolium salt with KHMDS at –78 °C in the presence of elemental selenium;^20^ later work by us has shown that these can be prepared conveniently at room temperature using potassium *tert*-butoxide as the base.^21,22^ A linear correlation between *δ*_Se_ (for the selenoureas) and *δ*_P_ (for the phosphinidenes) has been demonstrated for seven examples.^20^ More recently, Ciancaleoni and Belpassi conducted a detailed and thorough theoretical study of [Ni(CO)_3_L] and [Au(CO)L] complexes, showing that while the *ν*_CO_ of the former are excellent indicators of the overall electron density at the nickel centre, the latter indicate solely the π-accepting ability of L.^23^ Only a limited number of such gold complexes have been disclosed, but a synthetic route is known.^24,25^ Belpassi and Zuccaccia have also examined the effect of ligand L on σ-donation and π-back bonding in [AuL(NHC)] complexes.^26^

The phosphinidene and selenourea systems are the only systems where a considerable number of experimental data points are available; relatively few (structurally quite similar) examples of platinum and gold complexes of the necessary forms have been disclosed. For the phosphinidenes and selenoureas, the chemical shift trends are consistent with by chemical intuition what one might expect the scale of π-accepting ability to look like. 4,5-Dihydroimidazol-2-ylidenes are known to be more π-accepting than their imidazol-2-ylidene congeners, while one would expect the presence of carbonyl groups on the backbone to increase the π-accepting ability. However, a thorough and detailed analysis of the bonding in such compounds has yet to be carried out, in order to assess how well these chemical shift scales reflect bonding characteristics. We report the synthesis and characterisation of a range of selenoureas derived from imidazol-2-ylidenes, 4,5-dihydroimidazol-2-ylidenes and triazol-2-ylidenes. The selenourea adducts were prepared in preference to the phosphinidenes due to the more convenient synthetic protocol, requiring only a single synthetic step directly from the moisture- and air-stable imidazolium, 4,5-dihydroimidazolium or triazolium salts, and a simple work-up on the laboratory bench. We have used computational methods to explore the nature of the bonding in these compounds; in particular, we wished to explore whether these species do indeed allow quantification of π-backbonding alone.

## Results and discussion

### Synthesis and characterisation of new selenoureas

A range of selenourea compounds were prepared from the corresponding free NHCs or imidazolium salts in a straightforward manner; full details of the synthesis and characterisation of these species can be found in the ESI.† Compounds were prepared either by addition of the free carbene to excess elemental selenium, or by deprotonation of the NHC salt in the presence of excess selenium. For the latter approach, KO^*t*^Bu was used to deprotonate imidazolium and 4,5-dihydroimidazolium salts. Attempts to use the same base with triazolium salts led to decomposition; instead, it was found that an excess of K_2_CO_3_ could deprotonate the triazolium salt *in situ*, in a manner somewhat analogous to that used to prepare a wide range of NHC–metal complexes (M = Cu, Au, Pd, Rh, Ir).^27–30^ While triazolium salts are more acidic than (4,5-dihydro)imidazolium species, the p*K*_a_ is still relatively high (*ca.* 17 in aqueous solution *versus ca.* 20–25 for (4,5-dihydro)imidazolium salts).^31,32^ All products were air- and moisture-stable and could be worked up using bench-grade solvents. Some species, such as [Se(I^*t*^Bu)], were unstable when stored in solution for extended periods, however. All new compounds were fully characterised by ^1^H, ^13^C{^1^H} and ^77^Se{^1^H} NMR spectroscopy in chloroform-*d*; the latter analysis was also performed in acetone-*d*_6_ (where solubility permitted) to allow comparison with the results of Ganter.^20^ All materials were determined to be analytically pure by elemental analysis.

**Fig. 2:**
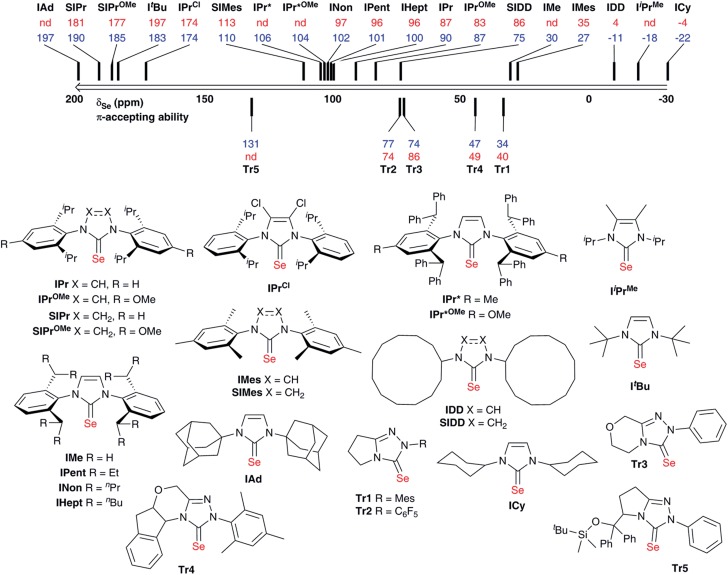
Selenourea compounds considered in this study, and their ^77^Se chemical shift values (*δ*_Se_) obtained in acetone-*d*_6_ (red) and chloroform-*d* (blue); not all compounds were sufficiently soluble in acetone-*d*_6_ for ^77^Se{^1^H} NMR analysis.

Data are presented in Fig. 2 for our complete set of selenoureas (24), which includes those synthesised as part of this study as well as those we have reported previously.^21,22^ The ^77^Se chemical shifts cover a range from 197 to –22 ppm, *versus* the range of 800–80 ppm reported by Ganter for a set of seven structurally very diverse selenoureas.^20^ This narrower range is somewhat expected, given that the majority of the compounds here feature a *N*,*N*′-diarylimidazol-2-ylidene motif, plus some selected triazol-2-ylidenes, while Ganter's original study covered a much wider range of carbene compounds. It should be noted at this point that the *δ*_Se_ values considered henceforth are those recorded in chloroform-*d*.

Some interesting trends are apparent from our data. Unsaturated imidazol-2-ylidenes bearing secondary alkyl *N*-substituents exhibit the lowest *δ*_Se_ (<0 ppm) and ought then to be the least π-accepting, followed by unsaturated *N*,*N*′-diarylimidazol-2-ylidenes and one example of a saturated *N*,*N*′-dialkyl-4,5-dihydroimidazol-2-ylidene (*ca.* 30–100 ppm). Saturated *N*,*N*′-diaryl species exhibit higher *δ*_Se_ (110–190 ppm), while IPr^Cl^, which bears chloride substituents on the backbone, also appears in this region (*δ*_Se_ = 174 ppm). Most interesting, I^*t*^Bu and IAd, which bear quaternary *N*-alkyl substituents, exhibit very high chemical shifts (*δ*_Se_ = 183 and 197 ppm, respectively). While saturated NHCs are known to be more π-accepting than unsaturated NHCs, this difference amongst *N*,*N*′-dialkylimidazol-2-ylidenes was very intriguing.

Notably, structurally similar unsaturated bis(aryl) NHCs led to quite different values of *δ*_Se_ (*cf.* [Se(IMes)] and [Se(IPr)]), although the selenoureas derived from IPr, IPent, IHept and INon (which differ only in the aryl 2,6-substitution pattern) all exhibit similar *δ*_Se_. For the triazol-2-ylidenes, Tr2 exhibited higher *δ*_Se_ than Tr1, while Tr5 exhibited the highest *δ*_Se_ of these compounds.

**Fig. 3:**
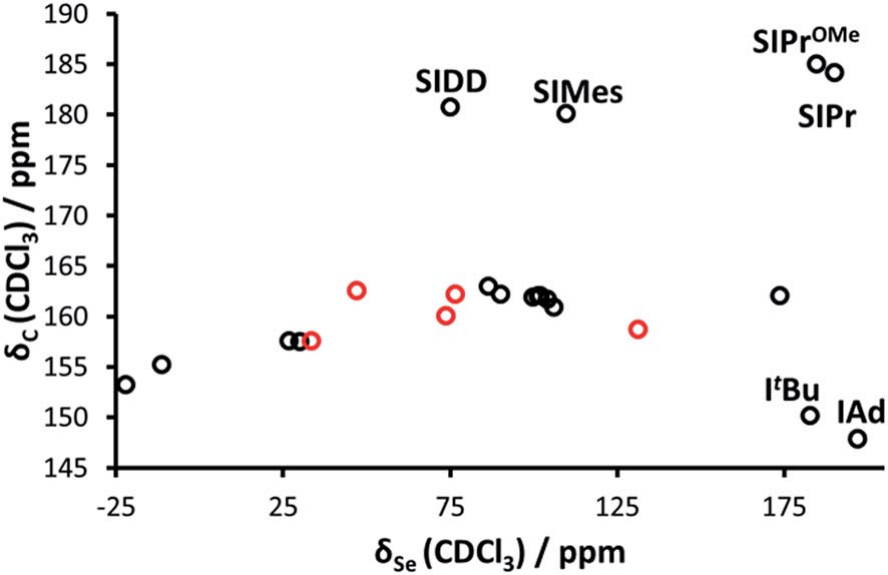
*δ*_C_ (carbene C2) plotted *versus δ*_Se_ for the selenourea compounds considered in this study.

Interestingly, while Bertrand demonstrated a linear correlation between *δ*_P_ and *δ*_C_ (for the carbene C2) in the phosphinidene adducts, the correlation is much poorer for selenourea compounds based on imidazol-2-ylidene compounds (Fig. 3), with selenoureas derived from saturated NHCs and bulky bis(alkyl) NHCs clearly lying away from the others. Bertrand's study does cover a greater variety of structures, and it should be noted that in the aforementioned study, IMes/IPr and SIMes/SIPr lie on opposite sides of the *δ*_P_/*δ*_C_ trendline.

**Fig. 4:**
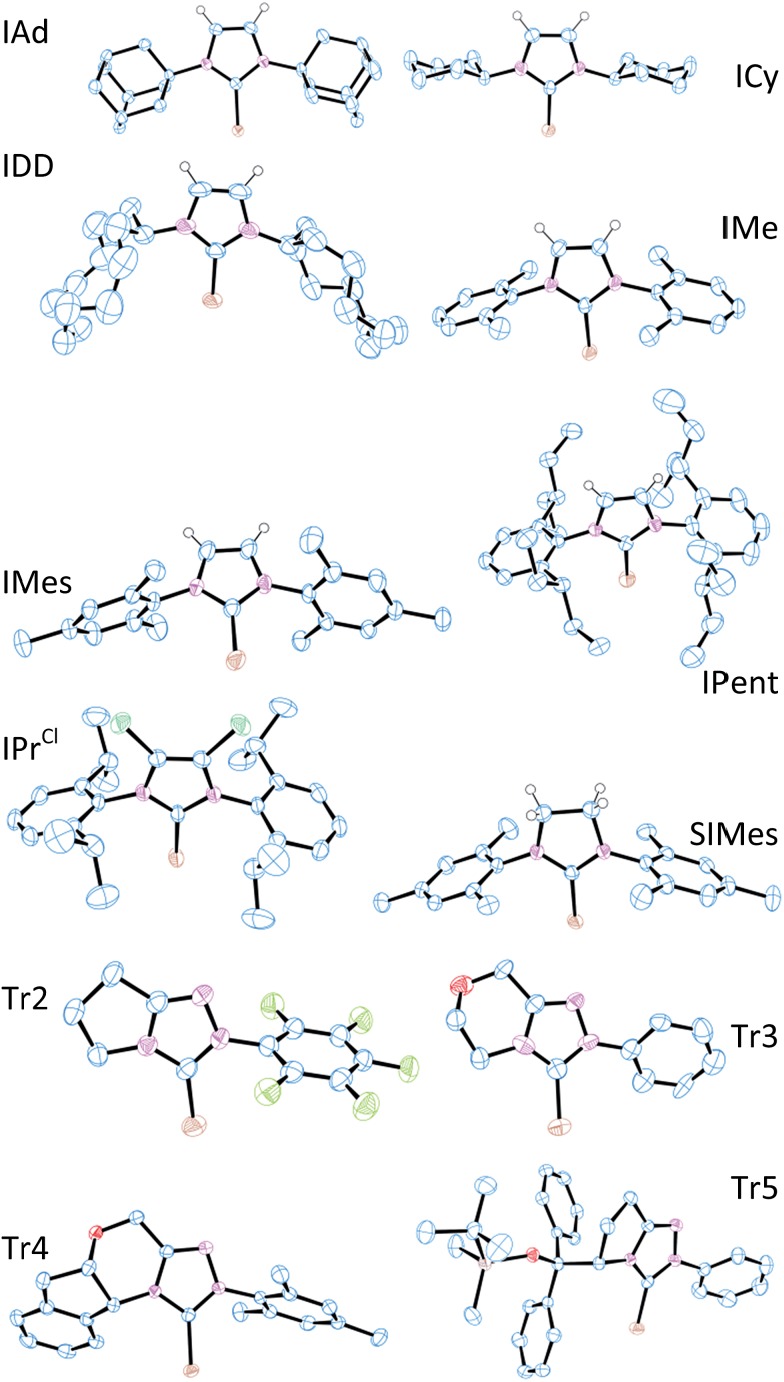
X-ray crystal structures for selenoureas derived from IAd, ICy, IDD, IMe, IMes, IPr^Cl^, IPent, SIMes, and Tr2–Tr5. All H atoms except the backbone H atoms have been excluded for clarity; thermal ellipsoids are drawn at 50% probability.

X-ray crystal structure data were obtained for most of these selenoureas (Fig. 4). The crystal structures of [Se(I^i^Pr^Me^)], [Se(IPr)], [Se(SIPr)], [Se(IPr*)], [Se(IPr^OMe^)], [Se(SIPr^OMe^)], and [Se(IPr*^OMe^)] are already known.^21,33^ Crystals suitable for these studies were typically prepared from slow diffusion of pentane or hexane into an acetone or dichloromethane solution of the compound. Unfortunately, suitable data for [Se(IHept)] and [Se(INon)] could not be obtained due to the highly disordered nature of the alkyl chains, while [Se(I^*t*^Bu)] and [Se(SIDD)] decomposed in solution. Full crystal structure data can be found in the ESI.† C–Se bond lengths varied between 1.82 and 1.86 Å, but there was no correlation between C–Se distance and *δ*_Se_. In one example ([Se(Tr3)]) where there were two independent structures in the unit cell, the C–Se bond lengths were 1.831(12) Å and 1.857(14) Å, suggesting that *ca.* 0.03 Å bond length differences are not meaningful. Some compounds exhibit rather short Se–H distances which are close to or within the sum of Van der Waals radii (*ca.* 3.1 Å) (*e.g.* [Se(IPr*)], 3.207 Å; [Se(IPr*^OMe^)], 3.002 Å; [Se(IPr)], 3.162 Å; [Se(ICy)] and [Se(IDD)], *ca.* 2.8 Å).

### Computational studies of selenoureas

With these data in hand, DFT calculations were approached. X-ray crystal structure data were used (where available) as a starting point for geometry optimisations. A total of 24 selenoureas (covering derivatives of imidazol-2-ylidenes, 4,5-dihydroimidazol-2-ylidenes and triazol-2-ylidenes) were considered using DFT methods. Calculations were carried out using the Amsterdam density functional suite^34–36^ at the BP86/TZ2P level of theory^37,38^ (see the ESI† for full computational details). As for a comparison between DFT and X-ray structures, focusing on the most relevant NHC–Se bond length, plotting the DFT optimised distances *versus* the X-ray distances of the selenoureas of Fig. 4 results in a clear correlation, with *R* ^2^ = 0.84.

**Fig. 5:**
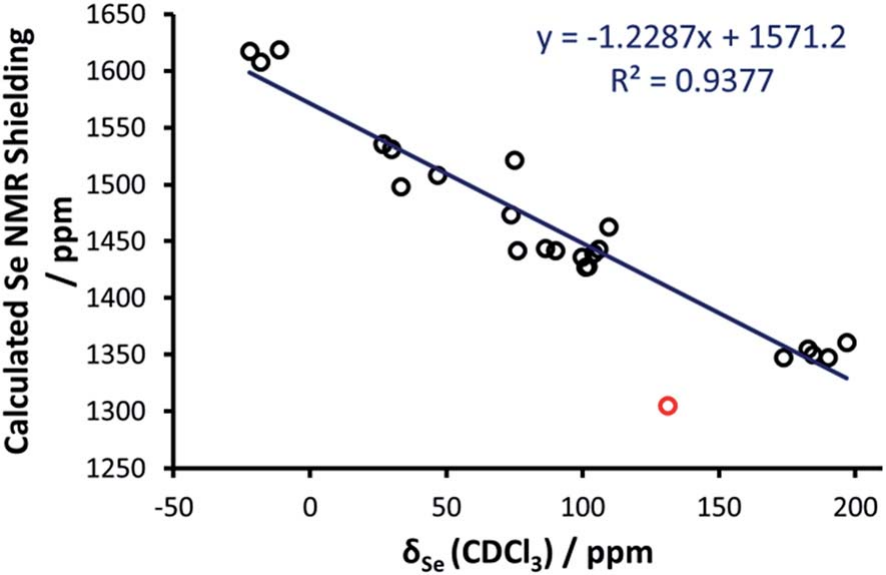
Calculated chemical shielding *versus* experimental NMR chemical shift for the selenium centres in the selenoureas in Fig. 2, where the red point corresponds to [Se(Tr5)] and is not included in the trendline.

Initial efforts were made to reproduce the experimental trend of the Se chemical shift in the selenoureas, which was seen as an important prerequisite before conducting more detailed analyses of the bonding and energies involved. Going into details, an excellent correlation (*R* ^2^ = 0.89) was obtained between the DFT isotropic shielding of the Se atom and the experimental chemical shift (Fig. 5). The only outlier was the triazole complex [Se(Tr5)] and by excluding this complex the correlation was improved significantly (*R* ^2^ = 0.94). Thus, complex [Se(Tr5)] was omitted from the analysis. The otherwise excellent correlation between the experimental Se chemical shift and the DFT calculated isotropic shielding validates the following analysis.

**Fig. 6:**
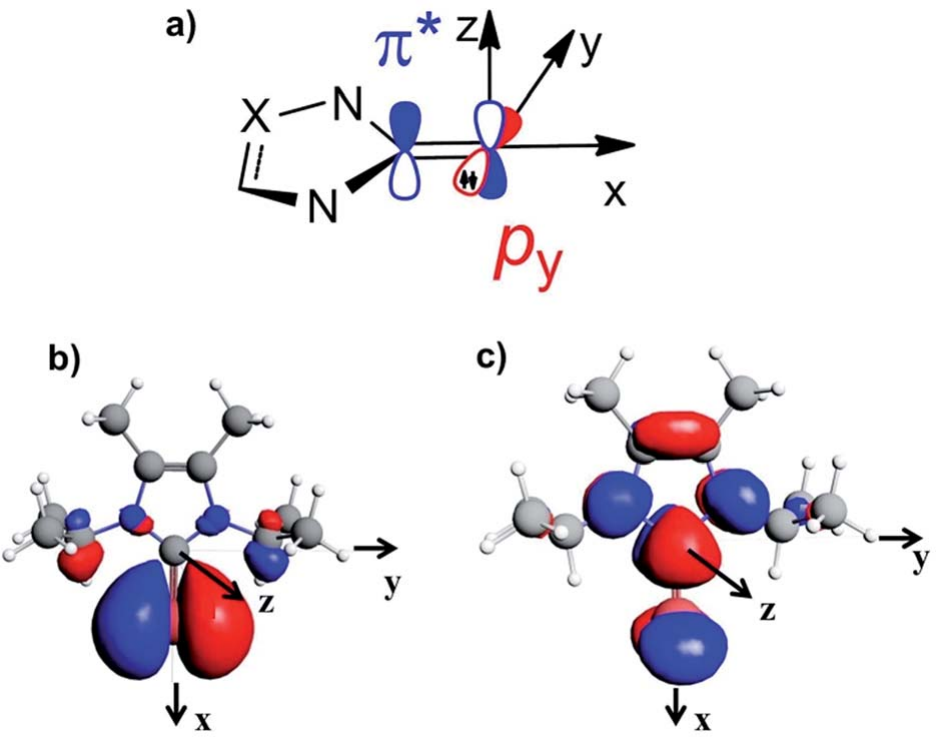
(a) Schematic representation of the filled Se(p_*y*_) and empty Se–NHC(π*) orbitals; (b) and (c) isodensity plots for the Se(p_*y*_) and Se–NHC(π*) orbitals of [Se(I^i^Pr^Me^)].

To rationalise the calculated NMR shielding of the Se atom in the 23 selenoureas considered (*i.e.* excluding [Se(Tr5)]), we examined the diamagnetic and paramagnetic shielding terms (*σ*_d_ and *σ*_p_). This analysis indicated that the main variable is the *σ*_p_ term, which oscillates in a range of 271 ppm, while the *σ*_d_ term remains almost the same, covering a range of only 3 ppm (see Table S2 in the ESI†). The paramagnetic shielding results from transitions of electrons between occupied and virtual orbitals, properly connected by symmetry, induced by the external magnetic field, and the amount of the shielding is related to the energy gap between these two orbitals.^39–41^ Analysis of the paramagnetic shielding tensor indicates that the largest changes are in the *σ*_p_ component oriented along the Se–NHC bond, *σ*_p_(*xx*), which corresponds to the *x* axis in Fig. 6(a). More detailed analysis of the paramagnetic shielding in terms of orbitals,^40,42^ indicated that *σ*_p_(*xx*) mainly depends on Se(p_*y*_) → Se–NHC(π*) transitions between the occupied p_*y*_ orbital on the Se atom and the virtual π* orbital of the selenourea; Fig. 6(b) and (c) show the Se(p_*y*_) and Se–NHC(π*) orbitals for [Se(I^i^Pr^Me^)].

**Fig. 7:**
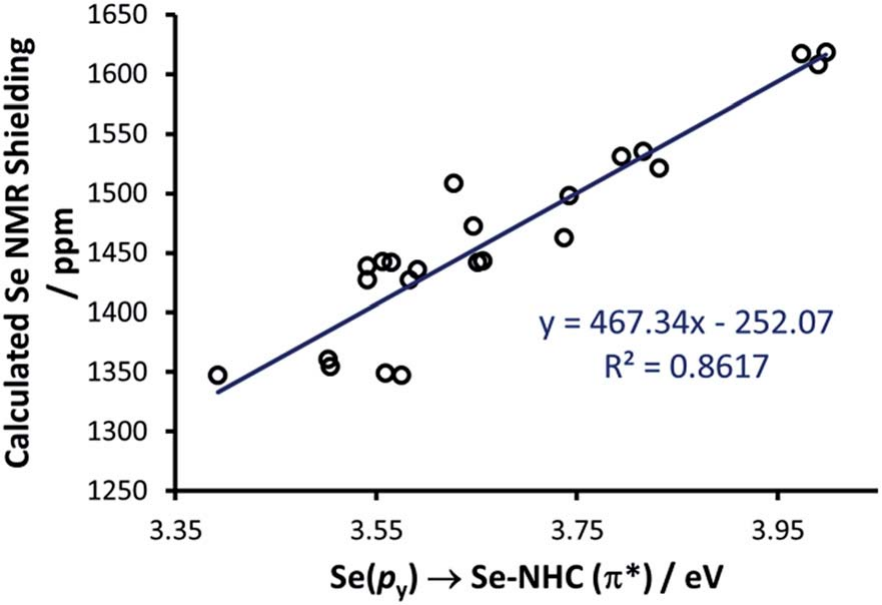
Se(p_*y*_) → Se–NHC(π*) energy gap *versus* calculated Se NMR shielding for 23 selenoureas.

**Fig. 8:**
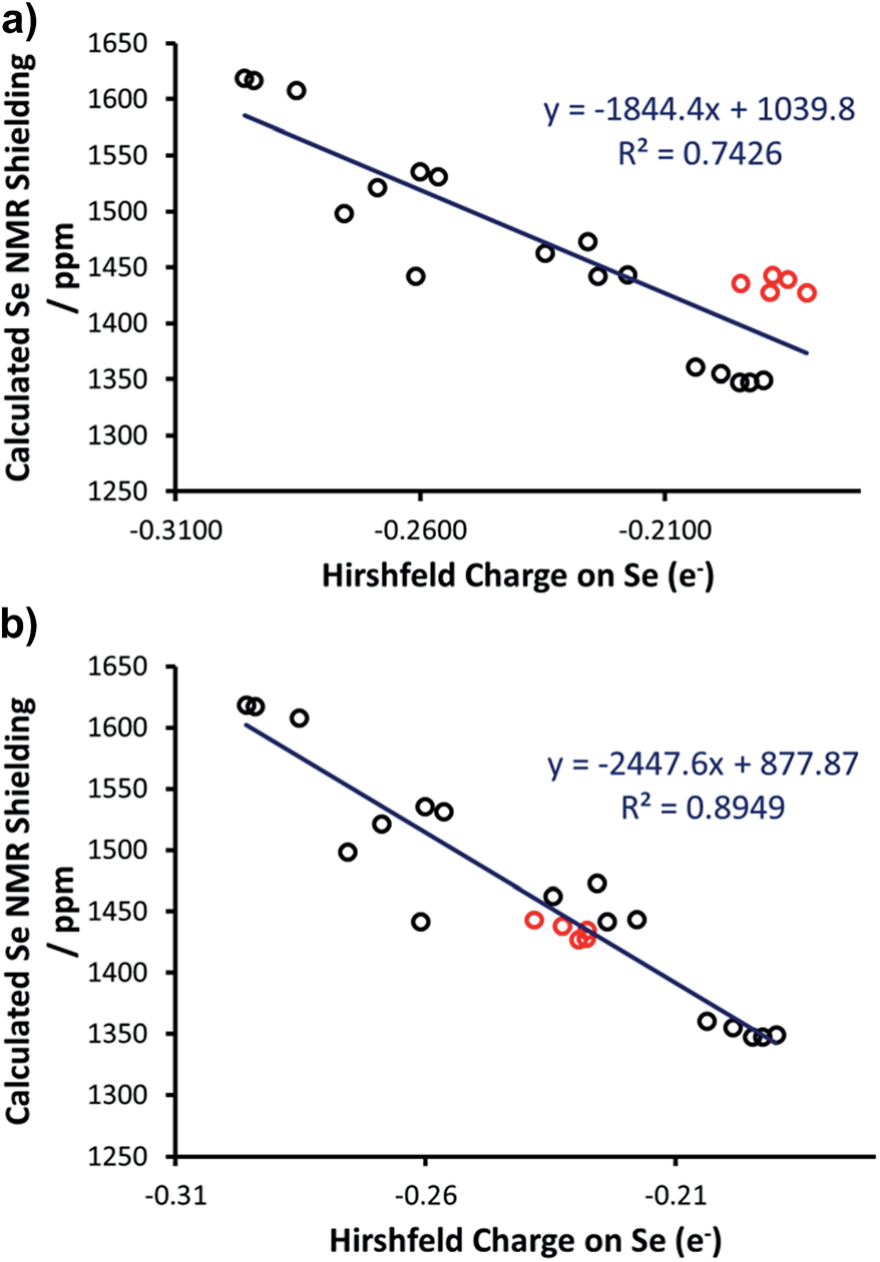
(a) Hirschfeld charge on Se *versus* calculated Se NMR shielding (excl. [Se(Tr5)]), with outliers highlighted in red; (b) Hirschfeld charge on Se *versus* calculated Se NMR shielding (excl. [Se(Tr5)]) with Se–H distances constrained to ≥3.5 Å.

**Fig. 9:**
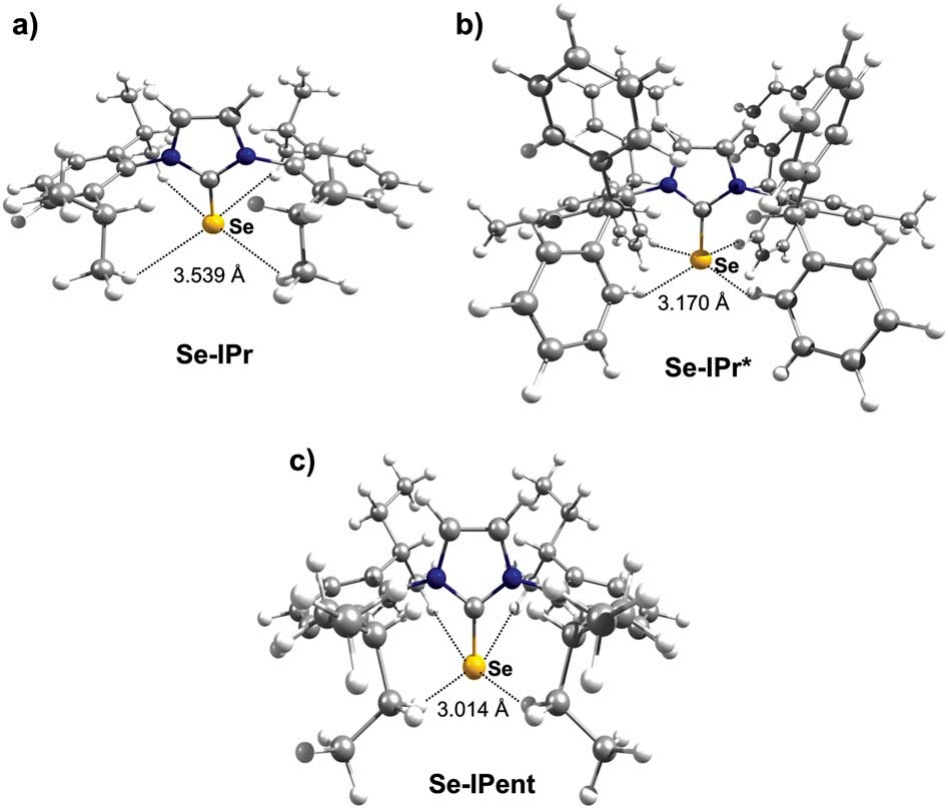
Optimized geometries for (a) [Se(IPr)], (b) [Se(IPr*)] and (c) [Se(IPent)], showing Se–H distances.

Thus, we plotted the calculated Se chemical shielding *versus* the Se(p_*y*_) → Se–NHC(π*) energy gap and we observed a reasonably good correlation (*R* ^2^ = 0.86) (Fig. 7); full results including the orbital energies of Se(p_*y*_), Se–NHC(π*), and their energy gap can be found in Table S3 of the ESI.† While the above analysis offers an explanation for the origin of the chemical shift of Se, the Se(p_*y*_) → Se–NHC(π*) energy gap depends on the overall electron density on the Se atom. For this reason, we searched for a correlation between the calculated isotropic shielding of Se and the Hirshfeld atomic charge on the Se atom (Fig. 8(a)); while the correlation is rather good (*R* ^2^ = 0.74), a group of outliers was evident on the plot (selenoureas of IPr*, IPr*^OMe^, IPent, IHept, and INon, highlighted in red). Inspection of the optimised geometries revealed short (3.0–3.2 Å) Se–H distances (Fig. 9). These distances are much shorter than those found in closely-related compounds (*e.g.* [Se(IPr)]).

Considering that Hirshfeld charges are built by partitioning electron density in each point of space to atoms according to a distance based criterion, we tested if imposing a minimum Se–H distance of 3.5 Å would improve the correlation between the isotropic Se shielding and the Hirshfeld charges. Therefore, restrained optimisations for all the outlier complexes of Fig. 8(a) were carried out with short Se–H distance frozen to 3.5 Å. This constraint resulted in geometries slightly higher in energy with the maximum energy penalty amounting to 3.0 kcal mol^–1^ (for [Se(IPent)]) and changed the DFT chemical shieldings by a maximum of 1.0 ppm (for [Se(IPr*^OMe^)]), indicating that these constrained geometries also capture the structure and the NMR properties of the system well. However, the Hirshfeld charges of the constrained geometries are clearly different, providing a fairly good correlation with the DFT chemical shielding (*R* ^2^ = 0.89) (Fig. 8(b)).

As a further test, the Hirshfeld charges were calculated for the [Se(IPr)] and [Se(IPent)] adducts using the hybrid B3LYP functional, but again the Se charge in the two systems is clearly different (0.214*e versus* 0.170*e*). Besides a possible weakness of the Hirshfeld charges in this case, a possible explanation is that the optimised geometries poorly represent the real behaviour in solution. Indeed, it has been clearly demonstrated that NHC ligands are flexible,^43^ and this could be particularly relevant for large ‘bulky but flexible’ NHCs such as IPr*, IPr*^OMe^, IHept, IPent and INon. However, answering this question is beyond the scope of the present work. Nevertheless, in the remainder of this work these restrained geometries were used for these selenoureas.

**Fig. 10:**
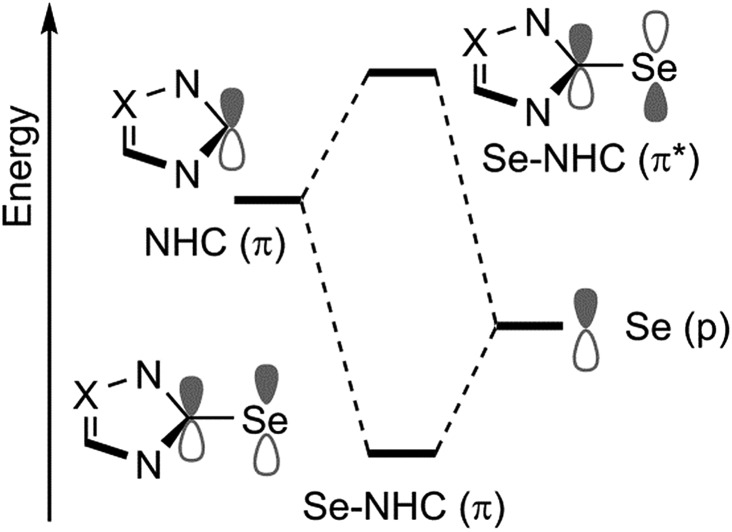
Schematic representation of the frontier molecular orbitals involving the Se(p) and NHC(π) orbitals. Se to NHC back-bonding occurs through in phase combination between a filled p orbital on Se with an empty π orbital of the NHC (Se–NHC (π)). Out of phase combination of the same orbitals (Se–NHC (π*)) is instead responsible for the paramagnetic shielding (Fig. 6(a)).

**Fig. 11:**
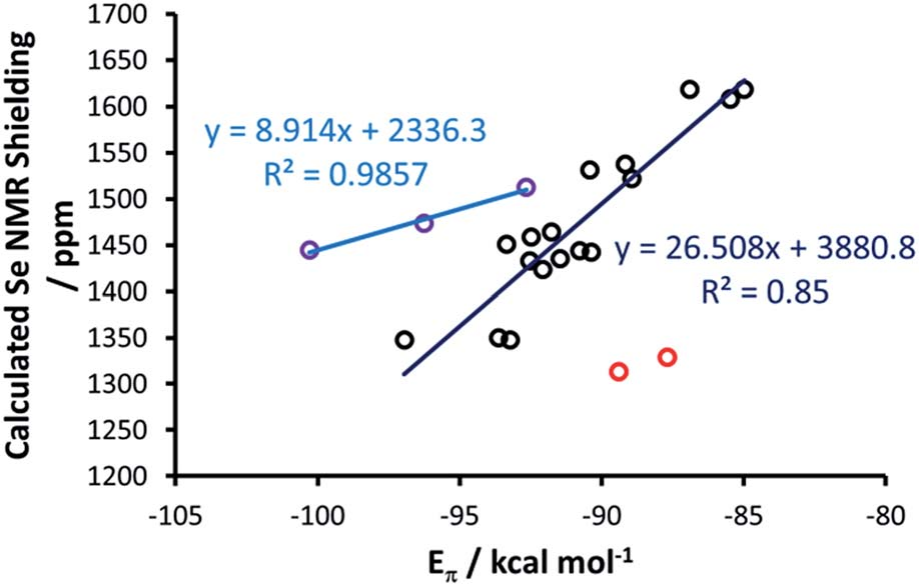
Calculated NMR shielding *versus E*_π_ for (4,5-dihydro)imidazol-2-ylidenes (black points), with the exception of [Se(IAd)] and [Se(I^*t*^Bu)] which are highlighted in red, and for triazol-2-ylidenes (purple points).

At this point, a bond energy decomposition analysis (BDA) on the Se–NHC complexes, rigidly fragmented into the Se and NHC moieties, was performed to shed light on the nature of the Se–NHC bond, with a focus on the extent of σ-donation from the NHC lone pair to the empty sp orbital of Se and, particularly, of π-back donation from a filled Se p_*z*_ orbital to the empty NHC π-orbital (see Fig. 10). To this end, the geometries of all the complexes were re-optimised under the constraint of *C*_S_ symmetry, with the NHC ring lying in the *σ*_*xy*_ plane, *i.e.* with the systems oriented as in Fig. 6(a), since this allows the orbital interaction energy contribution of the A′ and A′′ irreducible representations to be associated with the σ and π Se–NHC bonds (*E*_σ_ and *E*_π_), respectively (see the computational details in the ESI†). These constrained geometry optimisations were performed for all the compounds, including those derived from saturated NHCs and Tr1-3, since forcing them to be *C*_S_-symmetric requires only minor deformation. [Se(Tr4)] was excluded from this analysis, since forcing it into a plane would correspond to an unrealistic deformation. Both the *E*_σ_ and *E*_π_ plotted *versus* the calculated Se shielding result in poor correlations (*R* ^2^ = 0.25 and 0.28, respectively; see Fig. S3 in the ESI†). However, analysis of the residual errors (see Table S4 in the ESI†) indicated that the calculated Se shielding is consistently poorly correlated to *E*_σ_ for all systems, whereas in the case of *E*_π_ the correlation is normally good, and only a few systems have clearly larger residual errors, indicative of poor correlation. Specifically, the outliers are the IAd and the I^*t*^Bu selenoureas, with an *E*_π_ clearly too small, and the three selenoureas containing a triazole ring. Focusing on the latter, it was evident that the additional heteroatom in the triazol-2-ylidene ring completely altered the σ and π Se–NHC bonding scheme relative to classic imidazol-2-ylidene and 4,5-dihydroimidazol-2-ylidene complexes, justifying their separate treatment. Indeed, correlating *E*_π_ to the Se shielding in the triazol-2-ylidene adducts results in excellent correlation (*R* ^2^ = 0.99) (Fig. 11), but this only comprises three data points.

Regarding the imidazol-2-ylidene and 4,5-dihydro-imidazol-2-ylidene complexes, it should be noted that the IAd and I^*t*^Bu Se adducts already proved to be outliers in the correlation between the experimental *δ*_C_ (carbene C2) and the *δ*_Se_ (see Fig. 3) thus we decided to exclude them also from this analysis. Indeed, focusing on the remaining 17 imidazol-2-ylidene and 4,5-dihydroimidazol-2-ylidene complexes, a strong correlation (*R* ^2^ = 0.85) is gratifyingly achieved (Fig. 11), supporting the hypothesis that the *δ*_Se_ is indeed a measure of the π accepting capability of NHCs.

### Computational studies of phosphinidene adducts

**Fig. 12:**
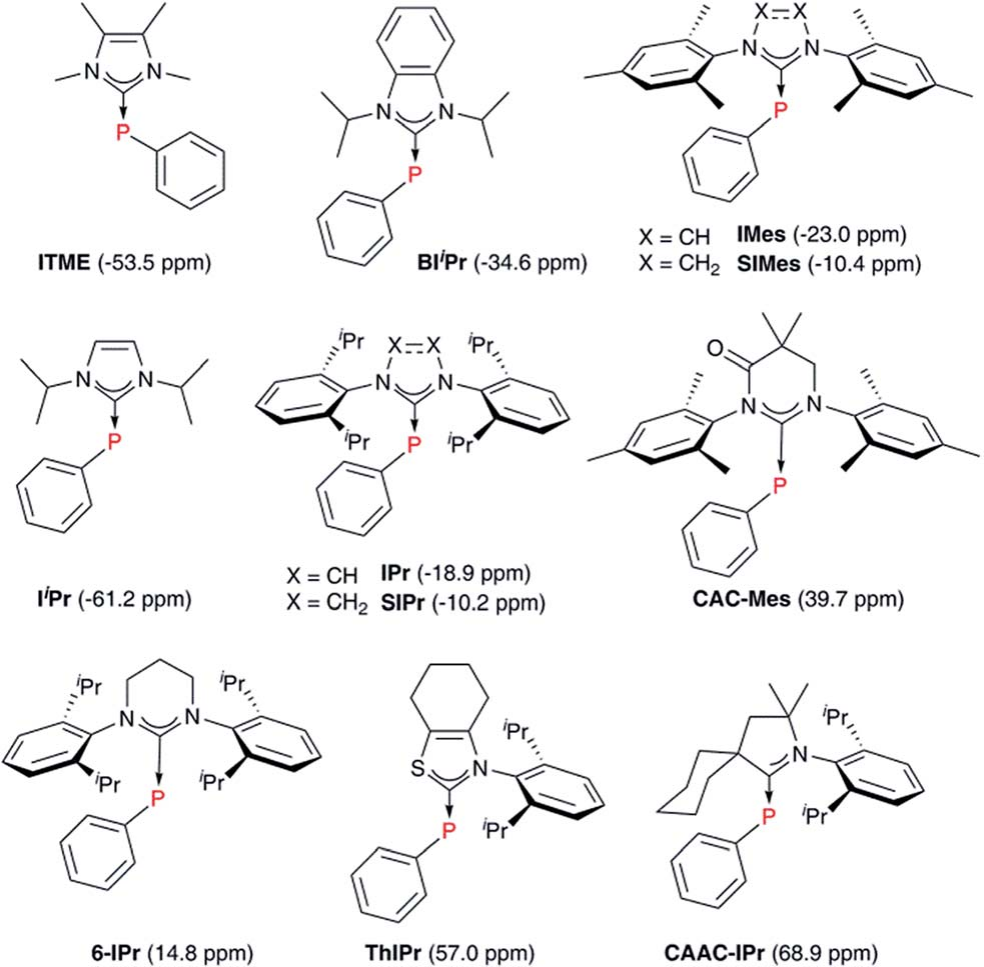
Phosphinidene adducts considered computationally.

**Fig. 13:**
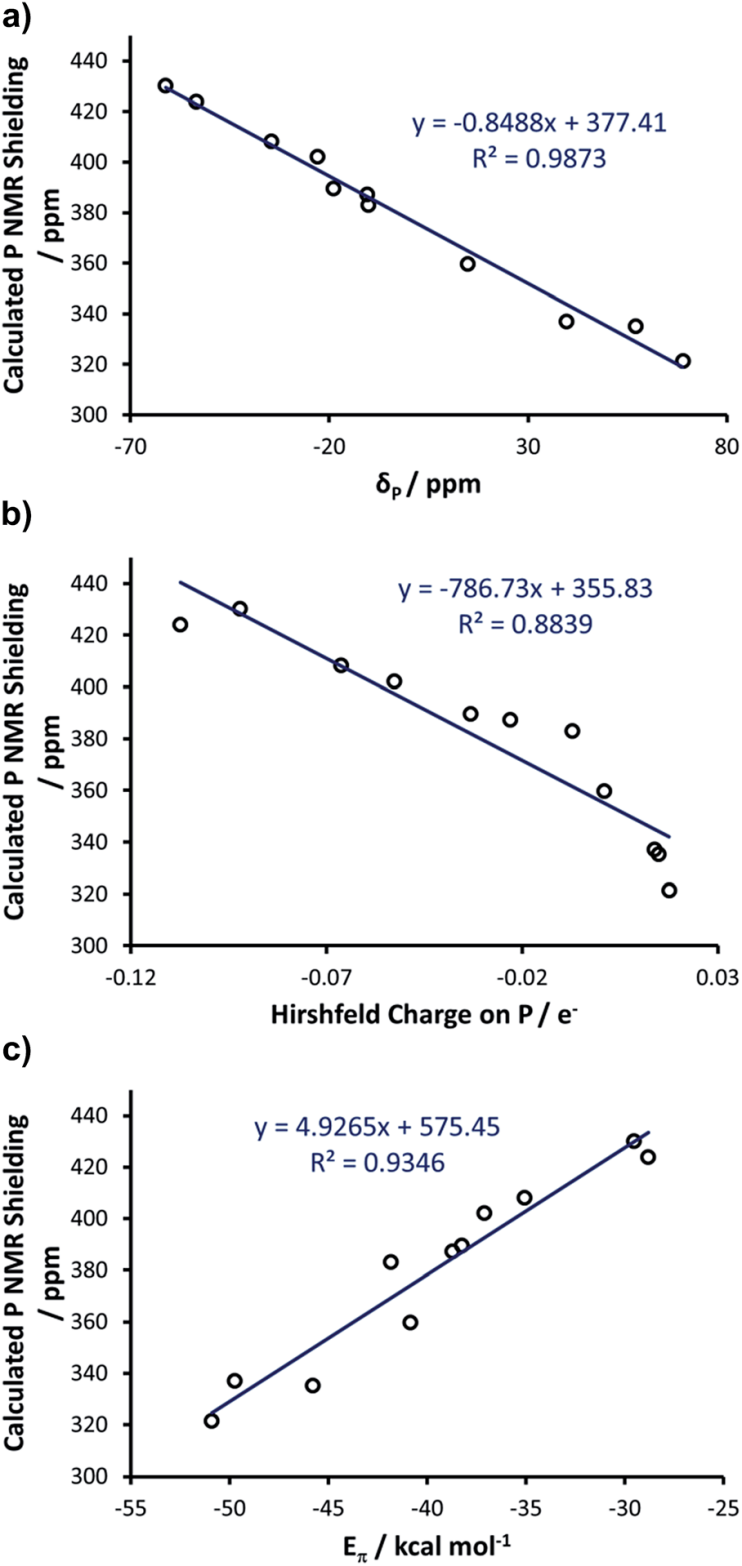
(a) Calculated P chemical shielding *versus* the experimental P chemical shift of carbene–phosphinidene adducts; (b) calculated NMR shielding *versus* the Hirshfeld charge on P; (c) calculated NMR shielding *versus E*_π_.

With these insights into the selenourea compounds in hand, the properties of the phosphinidene compounds were also explored, due to the similar way in which they are proposed to indicate the π-accepting properties (or lack thereof) of carbene compounds.^19^ The set of compounds in Fig. 12 was studied *in silico*. All of these species have been prepared and characterised by Bertrand and co-workers, although it should be noted that the solvent used for ^31^P NMR experiments varied (typically benzene-*d*_6_, but occasionally THF-*d*_8_ or CDCl_3_). These compounds were treated in a similar manner, yielding an excellent correlation (*R* ^2^ = 0.99) between the experimental *δ*_P_ chemical shift and the calculated chemical shielding (Fig. 13(a)); full results including the values of *σ*_d_ and *σ*_p_ components and Hirshfeld charges can be found in Table S5 of the ESI.† Subsequently, the calculated isotropic shielding of P was plotted *versus* the Hirshfeld atomic charge on the P atom, and a reasonably good correlation (*R* ^2^ = 0.88) was observed (Fig. 13(b)). For BDA, the geometry of all the phosphinidene complexes was re-optimised under the constraint of *C*_S_ symmetry. Similar to the selenium adducts, the calculated P shielding is poorly correlated to *E*_σ_ for all systems (*R* ^2^ = 0.10), whereas an excellent correlation (*R* ^2^ = 0.93) was found in the case of *E*_π_ (Fig. 13(c); see Table S6 in the ESI†). In short, consistent with the BDA in selenoureas, in the case of the NHC phosphinidene adducts we also found a good correlation between the chemical shielding and the amount of P to NHC back donation.

### Correlating Se and P

As final remarks, examination of data for the Se and PPh adducts with IMes, SIMes, IPr and SIPr allows for a comparison of the different NHCs, and of the relative strengths of the NHC–Se and NHC–PPh bonds. According to the *E*_σ_ and *E*_π_ for these systems (see Tables S4 and S6 in the ESI†), the Se–NHC bond is clearly stronger than the P–NHC bond, with *E*_σ_ and *E*_π_ for Se–NHC around 270 and 90 kcal mol^–1^ respectively, almost double the values for P–NHC (*ca.* 170 and 40 kcal mol^–1^, respectively). Nevertheless, for both systems the *E*_π_ is between 17 and 38% of *E*_σ_, highlighting the remarkable π-accepting capability of NHCs. As for a comparison between imidazol-2-ylidenes and 4,5-dihydroimidazol-2-ylidenes, the *E*_σ_ for SIMes and SIPr is 1–2 kcal mol^–1^ larger than that calculated for IMes and IPr, both for Se–NHC and P–NHC compounds. This slightly higher σ-donicity calculated for 4,5-dihydroimidazol-2-ylidenes than imidazol-2-ylidenes is consistent with previous analyses.^18^

Focusing on π-acidity, *E*_π_ for SIMes and SIPr is about 2–3 kcal mol^–1^ larger than that calculated for IMes and IPr, both for Se–NHC and P–NHC compounds. Although the difference is not particularly large, it is consistent with the higher π-accepting capability expected from 4,5-dihydroimidazol-2-ylidenes. Despite the difference in *E*_π_ being relatively small, it is responsible for the observed difference in the chemical shielding of ^77^Se, of about 80–100 ppm both experimentally and theoretically, between IMes and SIMes, as well as between IPr and SIPr. However, the window of about 250 ppm covered by experimental and DFT NMR data corresponds to a window in *E*_π_ of 12 kcal mol^–1^, and therefore this metric can identify and quantify considerable differences in π-accepting ability.

**Fig. 14:**
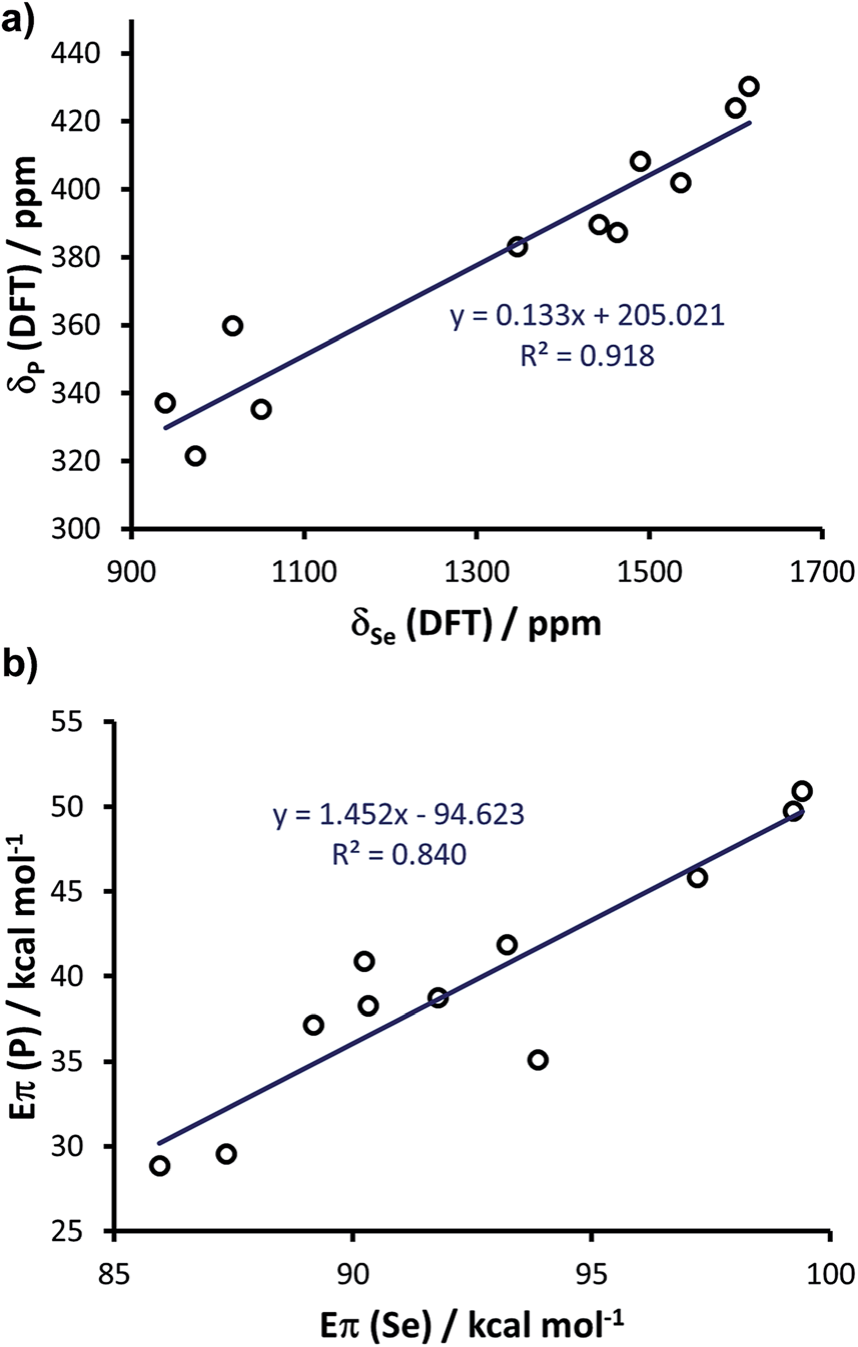
(a) Calculated chemical shielding of phosphinidenes *versus* selenoureas and (b) calculated *E*_π_ for 11 phosphinidenes (see Fig. 12) *versus* calculated *E*_π_ in the corresponding 11 selenoureas.

Since the above calculations indicated the capability of the chosen methodology to rationalise both selenourea and phosphinidene compounds independently, and in line with the evidence that there is a linear correlation between the experimental *δ*_p_ and *δ*_Se_,^20^ we explored further the possibility of correlating the two classes within a single framework. To this end, we enlarged the comparison to 11 systems, by calculating the selenoureas corresponding to all the phosphinidenes shown in Fig. 12 (see Table S7 in the ESI† for DFT ^77^Se shielding and *E*_π_ values). Plotting the DFT chemical shielding of ^77^Se *versus* that of ^31^P for the above 11 compounds results in a excellent linear correlation (*R* ^2^ = 0.92, similar to that reported by Ganter from experimental *δ*_Se_ and *δ*_P_ data) (Fig. 14(a)).^20^ At this point, we plotted *E*_π_ for the above 11 phosphinidenes *versus E*_π_ for the corresponding selenoureas, which results in quite a good correlation considering the enormous variation in structure amongst these examples (*R* ^2^ = 0.84) (Fig. 14(b)).

Analysis of Fig. 14(b) indicates that *E*_π_ for the phosphinidenes spans a 22 kcal mol^–1^ range in the 28.8–50.9 kcal mol^–1^ window, while selenoureas span a 13 kcal mol^–1^ range in the 86.0–99.4 kcal mol^–1^ window. The quantitative link between these two systems, established by this study, plus the known quantitative link between experimental *δ*_Se_ and *δ*_P_, allows for NHCs to be evaluated using either system and mapped onto the same quantitative scale.

While there is a clear quantitative trend that links these two systems, the different absolute values of *E*_π_ must result from the interactions of different orbitals on atoms with quite different properties (*i.e.* Se 4p_*z*_ and P 3p_*z*_). The good quantitative link between Se and P systems suggests very strongly that similar links between Se and/or P models and transition metal systems (which will interact *via* d → π* back-bonding) ought to be quantifiable. Such links require further, detailed calculations of model transition metal systems, and are beyond the scope of the current study.

## Conclusions

In conclusion, the link between the *δ*_Se_ chemical shifts of 24 selenoureas and *δ*_P_ chemical shifts of 11 phosphinidene compounds and the electronic properties of the NHCs from which they are derived have been probed. Experimentally, a range of (4,5-dihydro)imidazol-2-ylidene- and triazol-2-ylidene-based selenoureas have been prepared and fully characterised. For 24 selenoureas, NMR shieldings derived from calculations correlated well with those obtained experimentally (*R* ^2^ = 0.89, or 0.94 by excluding the triazol-2-ylidene-based complex [Se(Tr5)]). The calculated NMR shielding was found to be strongly correlated to the Se(p_*y*_) → Se–NHC(π*) energy gap (*R* ^2^ = 0.86) and the charge at the selenium centre (*R* ^2^ = 0.90). Finally, bond decomposition analysis indicated that the NMR shielding is correlated to the π-orbital interaction energy (*R* ^2^ = 0.85 for the imidazol-2-ylidenes, with two exceptions in the form of I^*t*^Bu and IAd; *R* ^2^ = 0.99 for 3 triazol-2-ylidenes). For 11 carbene–phosphinidene complexes, we observed an excellent correlation (*R* ^2^ = 0.99) between the experimental P chemical shift and the calculated chemical shielding. The latter was found to be strongly correlated to the charge at the phosphorous centre (*R* ^2^ = 0.89), as well as to the π orbital interaction energy (*R* ^2^ = 0.93). In summary, the chemical shifts of these NHC adducts do indeed reflect the importance of π-back bonding in the overall interaction with the selenium or phosphorus centre.

The key outcomes of this study are therefore fourfold: (i) a detailed analysis of the origin of the chemical shift measurements obtained experimentally has been carried out using DFT techniques; (ii) the link between *δ*_Se_ or *δ*_P_ and the ability of the corresponding NHC ligand to accept π-electron density has been established unambiguously, meaning that these techniques can be deployed with confidence to characterise and quantify the π-accepting capability of new and existing NHC ligands; (iii) the groundwork has been laid for the use of these calculations to predict the properties of NHCs; and finally (iv) data have been furnished for a series of key NHCs of interest to chemists utilising them as ligands in transition metal promoted catalysis, on their own as organocatalysts, or in conjunction with Lewis acids in frustrated Lewis pair promoted reactivity.
