# Supplementary material for: What can NMR spectroscopy of selenoureas and phosphinidenes teach us about the π-accepting abilities of N-heterocyclic carbenes?
Source: Chem Sci. 2015 Feb 16;6(3):1895–904. doi: 10.1039/c4sc03264k (PMC5812343; doi:10.1039/c4sc03264k)
Supplement: Supplementary file 1 [file SC-006-C4SC03264K-s001.pdf]

Electronic Supplementary Material (ESI) for Chemical Science.
This journal is © The Royal Society of Chemistry 2015

SUPPORTING INFORMATION FOR

What can NMR spectroscopy of selenoureas and phosphinidenes teach us about the π-accepting abilities of N-heterocyclic carbenes?

Sai V. C. Vummaleti,[†] David J. Nelson,[‡,§] Albert Poater,[¤] Adrián Gómez Suárez,[‡] David B. Cordes,[‡] Alexandra M. Z. Slawin,[‡] Steven P. Nolan,[‡,*] and Luigi Cavallo.[†,*]

† KAUST Catalyst Center, Physical Sciences and Engineering Division, King Abdullah University of Science and Technology, Thuwal, 23955-6900, Saudi Arabia; ‡ EaStCHEM School of Chemistry, University of St Andrews, Purdie Building, North Haugh, St Andrews, Fife, KY16 9ST, UK; § WestCHEM Department of Pure and Applied Chemistry, University of Strathclyde, Thomas Graham Building, 295 Cathedral Street, Glasgow, G1 1XL, UK; ¤ Institut de Química Computacional I Catàlisi and Department de Química, Universitat de Girona, Campus de Mo-tilivi, E-17071, Girona, Spain

Contents

Synthesis and Characterization of New Selenourea Compounds S2

NMR Spectra of New Selenourea Compounds S8

X-ray Crystal Structure Data for Selenourea Compounds S33

Computational Details S91

DFT Results S94

Coordinates S101

Synthesis and Characterization of New Selenourea Compounds

General. The selenoureas of IPr, SIPr, IMes, SIMes, IPr^{OMe}, $SIPr^{OMe}$, IPr*, IPr^{*OMe}, IPr^{Cl} and $I^{i}Pr^{Me}$ have been reported previously.[1-3] Imidazolium salts and free NHCs were prepared in-house using literature procedures.[4-7] $KO^{t}Bu$, K_2CO_3 and Se were used as received. THF was dried and degassed using an in-house solvent purification system. Chloroform-*d* and acetone-d_6 were used as received. Reactions were set up under an argon atmosphere in septum-fitted screw-cap vials, and worked up in the fume hood with bench-grade solvents (DCM, pentane). Bench grade solvents were also used to crystallize samples for X-ray analysis. NMR analyses were carried out using 11.7 T NMR spectrometers equipped with a BBFO-*z*-ATMA probe (^{1}H observe at 500 MHz; ^{19}F observe at 470 MHz; ^{13}C observe at 126 MHz; ^{77}Se observe at 95 MHz) or a QNP-*z* probe (^{1}H observe at 500 MHz; ^{13}C observe at 126 MHz). ^{1}H NMR spectra were referenced to the residual solvent signals and $^{13}C\{^{1}H\}$ NMR spectra were referenced to the resonances corresponding to the deuterated solvent,[8] while $^{77}Se\{^{1}H\}$ spectra were referenced externally to $(PhSe)_2$ and $^{19}F\{^{1}H\}$ NMR spectra were referenced externally to $CFCl_3$. *Safety note: selenium is highly toxic, and should be handled with care. In these experiments, solvents were removed carefully on the Schlenk line in a fume hood, and all contaminated materials were cleaned carefully or disposed of via a separate waste stream.*

Method A (from the imidazolium salt). The NHC salt (*ca.* 50 mg, weighed accurately) and $KO^{t}Bu$ (*ca*. 1.2 – 1.8 equiv.) were weighed into a vial. Excess Se (*ca*. 30 mg) was added, followed by a stirrer bar, and the vial was purged with Ar. Dry, degassed THF (1 mL) was added *via* the septum and the resulting suspension was stirred at room temperature overnight.

Method B (from the triazolium salt). The NHC salt (*ca.* 50 mg, weighed accurately) and *ca.* 5 equiv. K_2CO_3 were weighed into a septum-fitted vial, followed by excess Se (*ca.* 30 mg) and a stirrer bar. The vial was purged with Ar, dry and degassed THF (1 mL) was added *via* the septum and the resulting suspension was stirred at room temperature overnight.

Method C (from the free NHC). In an argon-filled glovebox, the free NHC was weighed into a screw-cap vial and dissolved in dry, degassed THF. On the Schlenk line, this solution was transferred *via* cannula to an oven-dried and Ar-purged vial containing excess Se and a stirrer bar. The resulting suspension was stirred at room temperature overnight.

Work-up. The solvent was evaporated and the resulting residue was suspended in DCM (*ca.* 2 mL) and filtered through a pad of celite. The pad was washed with further DCM (*ca.* 2 mL). The DCM was evaporated and the residue was washed with pentane (3 x *ca.* 1 mL). The IHept and INon derivatives are pentane-soluble so were not washed in this way. The resulting solids were dried under high vacuum to yield material that was pure by elemental analysis (C, H, N within 0.4% of the calculated values).

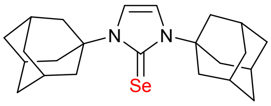

[Se(IAd)]. Obtained as a beige solid in 78% yield (47.2 mg) using method A. **^{1}H NMR** (500 MHz; $CDCl_3$): δ 6.97 (s, 2H, CH_{imid}), 2.75 (d, J = 2.7 Hz, 12H, CH_2), 2.21 (br., 6H, CH), 1.78 (app. d, J = 11.4 Hz, 6H, CH_2), 1.68 (app. d, J = 12.2 Hz, 6H, CH_2). **^{13}C{^{1}H} NMR** (126 MHz, $CDCl_3$): δ 147.92 (C_{carb}), 115.58 (CH_{imid}), 62.06 (CN), 40.13 (CH_2), 36.10 (CH_2), 30.25 (CH). **^{77}Se{^{1}H} NMR** (95 MHz, $CDCl_3$): δ 196.94. **Anal. Calcd.** For $C_{23}H_{32}N_2Se$ (415.47): C, 66.49; H, 7.76; N, 6.74. Found: C, 66.26; H, 7.88; N, 6.86. Insoluble in acetone.

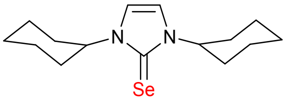

[Se(ICy)]. Obtained as a beige solid in 50% yield (41.0 mg) using method A. **^{1}H NMR** (500 MHz; $CDCl_3$): δ 6.88 (s, 2H, CH_{imid}), 4.88 (tt, J = 12.0, 3.8 Hz, 2H, NCH), 2.11-2.08 (m, 4H), 1.87-1.83 (m, 4H, CH_2), 1.76-1.72 (m, 2H, CH_2), 1.50 (ddt, J = 13.2, 13.2, 3.3 Hz, 4H, CH_2), 1.38 (ddd, J = 12.4, 12.4, 3.5 Hz, 4H, CH_2), 1.18 (ddt, J = 13.0, 13.0, 3.7 Hz, 2H, CH_2). **^{13}C{^{1}H} NMR** (126 MHz, $CDCl_3$): δ 153.22 (C_{carb}), 115.81 (CH_{imid}), 57.91 (NCH), 32.71 (CH_2), 25.53 (CH_2), 25.48 (CH_2). **^{77}Se{^{1}H} NMR** (95 MHz, $CDCl_3$): δ -22.13. **^{77}Se{^{1}H} NMR** (95 MHz, Acetone-d_6): δ -4.48. **Anal. Calcd.** For $C_{15}H_{24}N_2Se$ (311.32): C, 57.87; H, 7.77; N, 9.00. Found: C, 57.94 H, 7.73; N, 9.16.

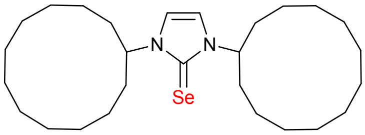

[Se(IDD)]. Obtained as a white solid in 81% yield (310.2 mg) using method B. **^{1}H NMR** (500 MHz; $CDCl_3$): δ 6.84 (s, 2H, CH_{imid}), 5.32-5.23 (m, 2H, NCH), 1.97-1.86 (m, 4H, CH_2), 1.75-1.62 (m, 4H, CH_2), 1.62-1.52 (m, 4H, CH_2), 1.45-1.23 (m, 30H, cyclododecyl. **^{13}C{^{1}H} NMR** (126 MHz, $CDCl_3$): δ 155.2 (C_{carb}), 116.3 (CH_{imid}), 55.4 (NCH), 29.9 (CH_2), 23.9 (CH_2), 23.8 (CH_2), 23.6 (CH_2), 21.6 (CH_2). **^{77}Se{^{1}H} NMR** (95 MHz, $CDCl_3$): δ -11.3. **^{77}Se{^{1}H} NMR** (95 MHz, Acetone): δ 3.6. **Anal. Calcd.** For $C_{27}H_{48}N_2Se$ (479.65): C, 67.61; H, 10.09; N, 5.84. Found: C, 67.48; H, 10.20; N, 5.97.

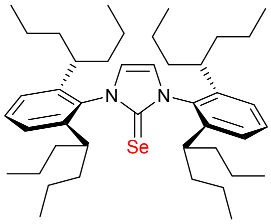

[Se(IHept)]. Obtained as an off-white solid in 61% yield (46.3 mg) using method A **^{1}H NMR** (500 MHz; $CDCl_3$): δ 7.42 (t, *J* = 7.7 Hz, 2H, C*H*$_{p\text{-Ar}}$), 7.21 (d, *J* = 7.8 Hz, 4H, C*H*$_{m\text{-Ar}}$), 6.84 (s, 2H, C*H*$_{imid}$), 2.42 (quintet, *J* = 7.0 Hz, 4H, C*H*), 1.83-1.75 (m, 4H, C*H*$_2$), 1.62-1.54 (m, 8H, C*H*$_2$), 1.50-1.40 (m, 8H, C*H*$_2$), 1.29-1.19 (m, 8H, C*H*$_2$), 1.16-1.09 (m, 4H, C*H*$_2$), 0.84 (t, *J* = 7.5 Hz, 12H, C*H*$_3$), 0.83 (t, *J* = 7.5 Hz, 12H, C*H*$_3$). **^{13}C{^{1}H} NMR** (126 MHz, $CDCl_3$): δ 161.94 (*C*$_{carb}$), 144.86 (*C*$_{o\text{-Ar}}$), 136.23(N*C*$_{Ar}$), 129.54 (*C*H$_{p\text{-Ar}}$), 124.69 (*C*H$_{m\text{-Ar}}$), 121.58 (*C*H$_{imid}$), 40.35 (*C*H), 39.53 (*C*H$_2$), 37.90 (*C*H$_2$), 21.45 (*C*H$_2$), 21.27 (*C*H$_2$), 14.78 (*C*H$_3$), 14.63 (*C*H$_3$). **^{77}Se{^{1}H} NMR** (95 MHz, $CDCl_3$): δ 99.77. **^{77}Se{^{1}H} NMR** (95 MHz, Acetone-*d*$_6$): δ 95.69. **Anal. Calcd.** For $C_{43}H_{68}N_2Se$ (691.97): C, 74.64; H, 9.90; N, 4.05. Found: C, 74.49; H, 9.98; N, 4.14.

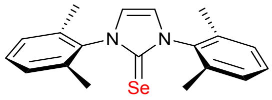

[Se(IMe)]. Obtained as a pale pink solid in 38% yield (27.4 mg) using method A. **^{1}H NMR** (500 MHz; $CDCl_3$): δ 7.31 (t, *J* = 7.6 Hz, 2H, C*H*$_{p\text{-Ar}}$), 7.21 (d, *J* = 7.6 Hz, 4H, C*H*$_{m\text{-Ar}}$), 6.99 (s, 2H, C*H*$_{imid}$), 2.18 (s, 12H, C*H*$_3$). **^{13}C{^{1}H} NMR** (126 MHz, $CDCl_3$): δ 157.48 (*C*$_{carb}$), 136.80 (*C*$_{o\text{-Ar}}$), 135.97 (N*C*$_{Ar}$), 129.75 (*C*H$_{p\text{-Ar}}$), 128.72 (*C*H$_{m\text{-Ar}}$), 120.21 (*C*H$_{imid}$), 18.32 (*C*H$_3$). **^{77}Se{^{1}H} NMR** (95 MHz, $CDCl_3$): δ 29.90. **Anal. Calcd.** For $C_{19}H_{20}N_2Se$ (355.34): C, 64.22; H, 5.67; N, 7.88. Found: C, 64.1; H, 5.72; N, 7.78. Insoluble in acetone.

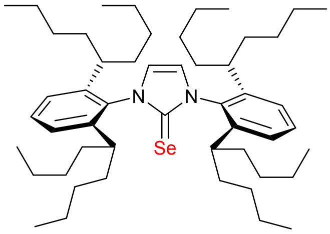

[Se(INon)]. Obtained as a pale orange solid in 68% yield (48.2 mg) using method A. **^{1}H NMR** (500 MHz; $CDCl_3$): δ 7.42 (t, *J* = 7.7 Hz, 2H, C*H*$_{p\text{-Ar}}$), 7.22 (d, *J* = 7.8 Hz, 4H, C*H*$_{m\text{-Ar}}$), 6.83 (s, 2H, C*H*$_{imid}$), 2.42 (quintet, *J* = 6.9 Hz, 4H, C*H*), 1.84-1.76 (m, 4H, C*H*$_2$), 1.66-1.54 (m, 8H, C*H*$_2$), 1.49-1.36 (m, 8H, C*H*$_2$), 1.29-1.19 (m, 26H, C*H*$_2$), 1.14-1.06 (m, 4H, C*H*$_2$), 0.85 (t, *J* = 7.1 Hz, 12H, C*H*$_3$), 0.83 (t, *J* = 7.1 Hz, 12H, C*H*$_3$). **^{13}C{^{1}H} NMR** (126 MHz, $CDCl_3$): δ 162.11 (*C*$_{carb}$), 144.95 (*C*$_{o\text{-Ar}}$), 136.29 (N*C*$_{Ar}$), 129.46 (*C*H$_{p\text{-Ar}}$), 124.69 (*C*H$_{m\text{-Ar}}$), 121.47 (*C*H$_{imid}$), 40.26 (*C*H), 36.93 (*C*H$_2$), 35.14 (*C*H$_2$), 30.39 (*C*H$_2$), 30.04 (*C*H$_2$), 23.53 (*C*H$_2$), 23.32 (*C*H$_2$), 14.21 (*C*H$_3$), 14.12 (*C*H$_3$). **^{77}Se{^{1}H}**

NMR (95 MHz, $CDCl_3$): δ 101.90. **^{77}Se{^{1}H} NMR** (95 MHz, Acetone): δ 97.05. **Anal. Calcd.** For $C_{51}H_{84}N_2Se$ (804.19): C, 76.17; H, 10.53; N, 3.48. Found: C, 76.05; H, 10.60; N, 3.56.

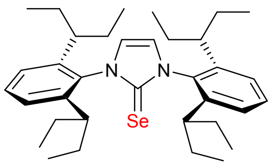

[Se(IPent)]. Obtained as a beige solid in 43% yield (33.2 mg) using method A. **^{1}H NMR** (500 MHz; $CDCl_3$): δ 7.43 (t, J = 7.8 Hz, 2H, $CH_{p\text{-Ar}}$), 7.21 (d, J = 7.8 Hz, 4H, $CH_{m\text{-Ar}}$), 6.86 (s, 2H, CH_{imid}), 2.29 (quintet, J = 6.9 Hz, 4H, C*H*), 1.92-183 (m, 4H, CH_2), 1.71-1.62 (m, 8H, CH_2), 1.54-1.45 (m, 4H, CH_2), 0.93 (t, J = 7.4 Hz, 12H, CH_3), 0.80 (t, J = 7.4 Hz, 12H, CH_3). **^{13}C{^{1}H} NMR** (126 MHz, $CDCl_3$): δ 162.11 (C_{carb}), 144.33 ($C_{o\text{-Ar}}$), 136.80 (NC_{Ar}), 129.53 ($CH_{p\text{-Ar}}$), 124.81 ($CH_{m\text{-Ar}}$), 121.59 (CH_{imid}), 42.88 (*C*H), 28.86 (CH_2), 27.62 (CH_2), 12.60 (CH_3), 12.42 (CH_3). **^{77}Se{^{1}H} NMR** (95 MHz, $CDCl_3$): δ 101.17. **^{77}Se{^{1}H} NMR** (95 MHz, Acetone): δ 95.90. **Anal. Calcd.** For $C_{35}H_{52}N_2Se$ (579.76): C, 72.51; H, 9.04; N, 4.83. Found: C, 72.48; H, 7.88; N, 6.87.

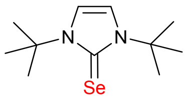

[Se(I^{t}Bu)]. Obtained as a yellow solid in 69% yield (44.9 mg) using method A. **^{1}H NMR** (500 MHz, $CDCl_3$): δ 6.98 (s, 2H, CH_{imid}), 1.90 (s, 18H, CH_3). **^{13}C{^{1}H} NMR** (126 MHz, $CDCl_3$): δ 150.15 (C_{carb}), 115.85 (CH_{imid}), 60.87 (N*C*), 29.06 (CH_3). **^{77}Se{^{1}H} NMR** (95 MHz, $CDCl_3$): δ 182.59. **^{77}Se{^{1}H} NMR** (95 MHz, Acetone): δ 197.26. **Anal. Calcd.** For $C_{11}H_{20}N_2Se$ (259.25): C, 50.96; H, 7.78; N, 10.81. Found: C, 50.82; H, 7.84; N, 10.69.

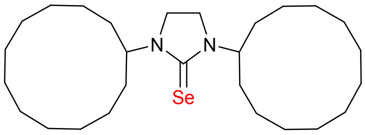

[Se(SIDD)]. Obtained as a white solid in 45% yield (28.0 mg) using method A. **^{1}H NMR** (500 MHz; $CDCl_3$): δ 5.06 (q, J = 6.6 Hz, 2H, NC*H*), 3.43 (s, 4H, $CH_{2\ imid}$), 1.79-1.72 (m, 4H, CH_2), 1.70-1.58 (m, 4H, CH_2), 1.42-1.24 (m, 36H, CH_2). **^{13}C{^{1}H} NMR** (126 MHz, $CDCl_3$): δ 180.76 (C_{carb}), 53.06 (N*C*H), 42.63 ($CH_{2\ imid}$), 27.87 (CH_2), 24.32 (CH_2), 24.13 (CH_2), 23.36 (CH_2), 22.72 (CH_2), 22.38 (CH_2). **^{77}Se{^{1}H} NMR** (95 MHz, $CDCl_3$): δ 75.01. **^{77}Se{^{1}H} NMR** (95 MHz, Acetone): δ 85.62. **Anal. Calcd.** For $C_{27}H_{50}N_2Se$ (481.66): C, 67.33; H, 10.46; N, 5.82. Found: C, 67.21; H, 10.55; N, 5.88.

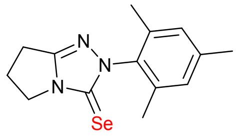

[Se(Tr1)]. Obtained as a white solid in 99% yield (50.4 mg) using method C. **^{1}H NMR** (500 MHz, $CDCl_3$): δ 6.96 (s, 2H, Ar CH), 4.07 (t, $^3J_{HH}$ = 7.3, 2H, NCH_2), 3.10 (t, $^3J_{HH}$ = 8.0, 2H, $CH_2C(N)=N$), 2.72 (app. quint., $^3J_{HH}$ = 7.6, NCH_2CH_2), 2.31 (s, 3H, Ar *p*-Me), 2.07 (s, 6H, Ar *o*-Me). **^{13}C{^{1}H} NMR** (126 MHz, $CDCl_3$): δ 158.6 (C=N), 157.6 (CSe), 140.1 (Ar *p*-C-CH_3), 136.0 (Ar C-N), 134.1 (Ar *o*-*C*-CH_3), 129.2 (Ar CH), 45.3 (NCH_2), 25.9 (NCH_2CH_2), 23.0 ($CH_2C(N)=N$), 21.3 (*p*-CH_3), 18.1 (*o*-CH_3). **^{77}Se{^{1}H} NMR** (95 MHz, $CDCl_3$): δ 33.5. **^{77}Se{^{1}H} NMR** (95 MHz, acetone-*d*$_6$): δ 40.2. **Anal.** Calcd for $C_{14}H_{17}N_3Se$: C, 54.90; H, 5.60; N, 13.72. Found: C, 54.87; H, 5.50; N, 13.65.

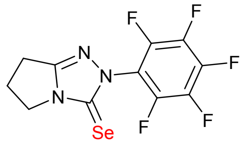

[Se(Tr2)]. Obtained as a pale orange solid in 87% yield (45.5 mg) using method C. **^{1}H NMR** (500 MHz, $CDCl_3$): δ 4.07 (t, $^3J_{HH}$ = 7.4, 2H, NCH_2), 3.14 (t, $^3J_{HH}$ = 8.1, 2H, $CH_2C(N)=N$), 2.76 (app. quint., $^3J_{HH}$ = 7.3, NCH_2CH_2), 2.31 (s, 3H, Ar *p*-Me), 2.07 (s, 6H, Ar *o*-Me). **^{13}C{^{1}H} NMR** (126 MHz, $CDCl_3$): δ 162.2 (CSe), 160.2 (C(N)=N), 144.2 (app. d, $^1J_{CF}$ = 257.5, Ar CF), 143.1 (app. d, $^1J_{CF}$ = 258.9, Ar CF), 138.1 (app. d, $^1J_{CF}$ = 252.8, Ar CF), 45.6 (NCH_2), 26.0 (NCH_2CH_2), 23.1 ($CH_2C(N)=N$). The Ar C-N signal could not be detected. **^{19}F{^{1}H} NMR** (470 MHz, $CDCl_3$): δ -141.7 – -141.8 (m, 2F), -149.7 (tt, $^3J_{FF}$ = 21.6, 2.9, 1F), -160.4 - -160.5 (m, 2F). **^{77}Se{^{1}H} NMR** (95 MHz, $CDCl_3$): δ 76.6. **^{77}Se{^{1}H} NMR** (95 MHz, acetone-*d*$_6$): δ 74.0. **Anal.** Calcd for $C_{11}H_6F_5N_3Se$: C, 37.31; H, 1.71; N, 11.87. Found: C, 37.42; H, 1.65; N, 12.04.

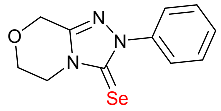

[Se(Tr3)]. Obtained as a beige solid in 88% yield (35.8 mg) using method C. **^{1}H NMR** (500 MHz, $CDCl_3$): δ 8.02 – 7.98 (m, 2H, Ar *o*-CH), 7.51 – 7.46 (m, 2H, Ar *m*-CH), 7.44 – 7.39 (m, 1H, Ar *p*-CH), 4.85 (s, 2H, $OCH_2C(N)=N$), 4.12 (t, $^3J_{HH}$ = 5.5, 2H, NCH_2CH_2O), 4.05 (t, $^3J_{HH}$ = 5.5, 2H, OCH_2CH_2N). **^{13}C{^{1}H} NMR** (126 MHz, $CDCl_3$): δ 160.1 (CSe), 147.4 (C(N)=CN), 138.3 (Ar C-N), 129.0 (Ar *m*-CH), 128.8 (Ar *p*-CH), 124.5 (Ar *o*-CH), 64.2 (NCH_2), 62.3 ($OCH_2C(N)=N$), 44.3 (OCH_2CH_2N) . **^{77}Se{^{1}H} NMR** (95 MHz, $CDCl_3$): δ 73.6. **^{77}Se{^{1}H} NMR** (95 MHz, acetone-*d*$_6$): δ 86.1. **Anal.** Calcd for $C_{11}H_{11}N_3OSe$: C, 47.15; H, 3.96; N, 15.00. Found: C, 47.34; H, 3.74; N, 15.08.

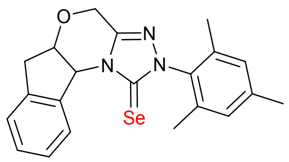

[Se(Tr4)]. Obtained as a pale orange solid in 96% yield (32.3 mg) using method C. **^{1}H NMR** (500 MHz, $CDCl_3$): δ 8.13 (d, $^{3}J_{HH}$ = 7.5, 1H, Ar CH), 7.38-7.33 (m, 2H, Ar CH), 7.32-7.26 (m, 1H, Ar CH), 7.05 (s, 2H, Ar CH), 5.91 (d, 1H, $^{3}J_{HH}$ = 3.4, CH), 4.96 (d, 1H, $^{2}J_{HH}$ = 15.8, CH_2), 4.78 (d, 1H, $^{2}J_{HH}$ = 15.8, CH_2), 4.75 (t, $^{3}J_{HH}$ = 4.0, 1H, CH), 3.40 (dd, $^{2}J_{HH}$ = 16.5, $^{3}J_{HH}$ = 4.3, 1H, CH_2), 3.25 (d, $^{2}J_{HH}$ = 16.5, 1H, CH_2), 2.38 (s, 3H, Ar *p*-Me), 2.16 (s, 3H, Ar *o*-Me), 2.12 (s, 3H, *o*-Me). **^{13}C{^{1}H} NMR** (126 MHz, $CDCl_3$): δ 162.6 (CSe), 147.4 (C(=N)N), 140.4 (*p*-Ar-*C*CH_3), 140.0 (Ar C), 138.3 (Ar C), 136.0 (*o*-Ar-*C*CH_3), 135.7 (*o*-Ar-*C*CH_3), 133.7 (Ar C-N), 129.4 (Ar *m*-CH), 129.4 (Ar *m*-CH), 129.1 (Ar CH), 127.4 (Ar CH), 127.1 (Ar CH), 125.2 (Ar CH), 78.5 (CH), 61.1 (CH), 60.5 (CH_2), 37.8 (CH_2), 21.4 (*p*-CH_3), 18.2 (*o*-CH_3), 17.9 (*o*-CH_3). **^{77}Se{^{1}H} NMR** (95 MHz, $CDCl_3$): δ 46.9. **^{77}Se{^{1}H} NMR** (95 MHz, acetone-d_6): δ 48.9. **Anal.** Calcd for $C_{21}H_{21}N_3OSe$: C, 61.46; H, 5.16; N, 10.24. Found: C, 61.40; H, 5.20; N, 10.14.

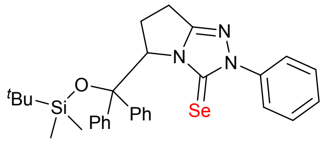

[Se(Tr5)]. Obtained as a pale yellow solid in 83% yield (46.8 mg) using method C. **^{1}H NMR** (500 MHz, $CDCl_3$): δ 7.77 (app. t, J = 8.1, 4H, C(OTBS)Ph_2 ArH), 7.42 (app. t, J = 8.1, 4H, C(OTBS)Ph_2 ArH), 7.39 – 7.24 (m, 7H, C(OTBS)Ph_2 *p*-ArH and NPh ArH), 5.72 (d, $^{3}J_{HH}$ = 8.6, 1H, CH(C(OTBS)Ph_2)), 3.24 (dd, $^{2}J_{HH}$ = 13.8, $^{3}J_{HH}$ = 8.9, 1H, CH_2CH_2C(=N)N), 2.82 – 2.71 (m, 1H, CH_2CH_2C(=N)N), 2.35 (dd, $^{2}J_{HH}$ = 16.7, $^{3}J_{HH}$ = 9.9, 1H, CH_2CH_2C(=N)N), 1.57 (dt, $^{2}J_{HH}$ = 17.0, $^{3}J_{HH}$ = 9.6, 1H, CH_2CH_2C(=N)N), 1.01 (s, 9H, Si$(CH_3)_2$(C$(CH_3)_3$)), -0.35 (s, 3H, Si$(CH_3)_2$(C$(CH_3)_3$)), -0.39 (s, 3H, Si$(CH_3)_2$(C$(CH_3)_3$)). **^{13}C{^{1}H} NMR** (126 MHz, $CDCl_3$): δ 160.4 (C(=N)N), 158.7 (CSe), 140.2 (ArC), 140.0 (ArC), 139.1 (ArC), 131.5 (C(OTBS)Ph_2 ArCH), 129.0 (Ar CH), 128.7 (Ar CH), 128.7 (Ar CH), 128.4 (Ar CH), 128.2 (Ar CH), 127.6 (Ar CH), 127.6 (Ar CH), 125.4 (Ar CH), 82.4 (C(OTBS)Ph_2), 64.8 (*CH*C(OTBS)Ph_2), 30.3 (CH_2CH_2C(=N)N), 26.5 (OSi$(CH_3)_2$(C$(CH_3)_3$)), 20.9 (CH_2CH_2C(=N)N), 19.2 (OSi$(CH_3)_2$(*C*$(CH_3)_3$)), -2.8 (OSi$(CH_3)_2$(C$(CH_3)_3$)), -3.3 (OSi$(CH_3)_2$(C$(CH_3)_3$)). **^{77}Se{^{1}H} NMR** (95 MHz, $CDCl_3$): δ 131.2. **Anal.** Calcd for $C_{30}H_{35}N_3OSeSi$: C, 64.27; H, 6.29; N, 7.49. Found: C, 64.66; H, 6.22; N, 7.39. Insoluble in acetone.

NMR Spectra of New Selenourea Compounds

[Se(IAd)]

^{1}H in $CDCl_3$

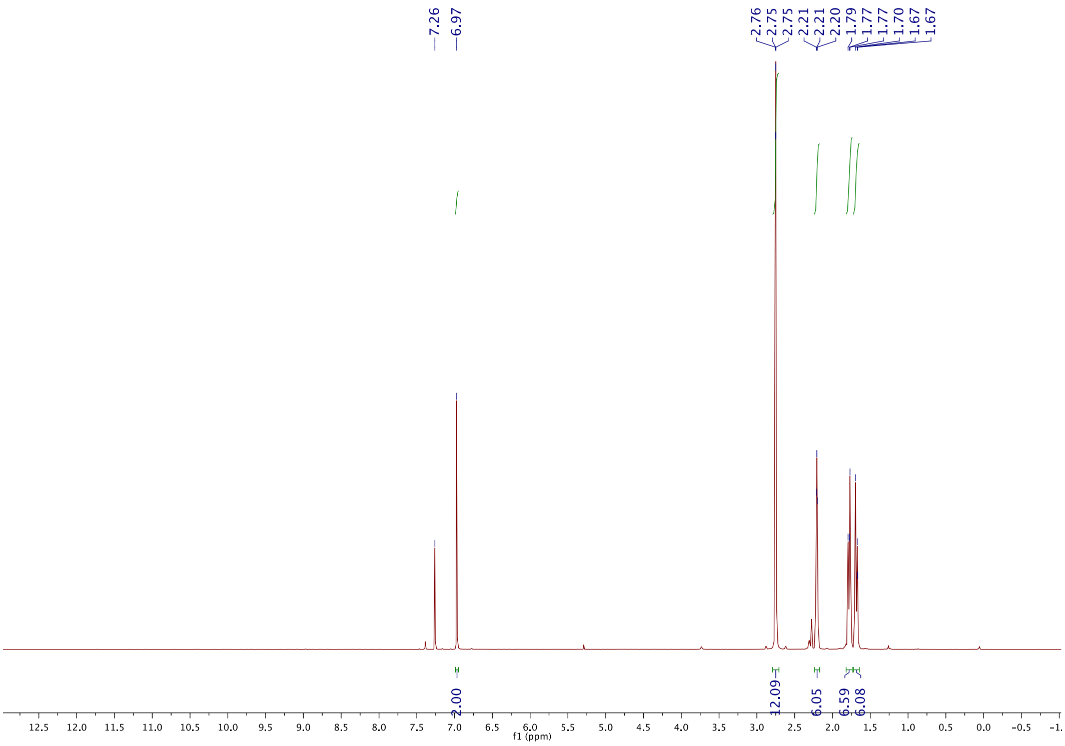

$^{13}C\{^{1}H\}$ DEPTQ NMR in $CDCl_3$

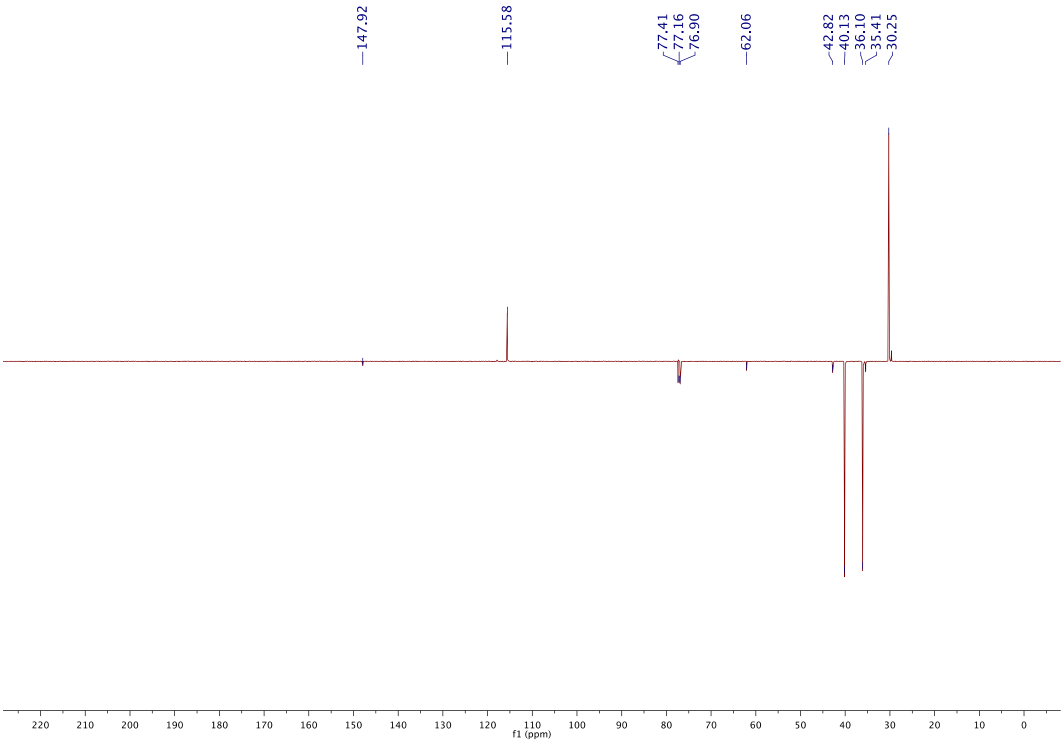

$^{77}Se\{^1H\}$ NMR in $CDCl_3$

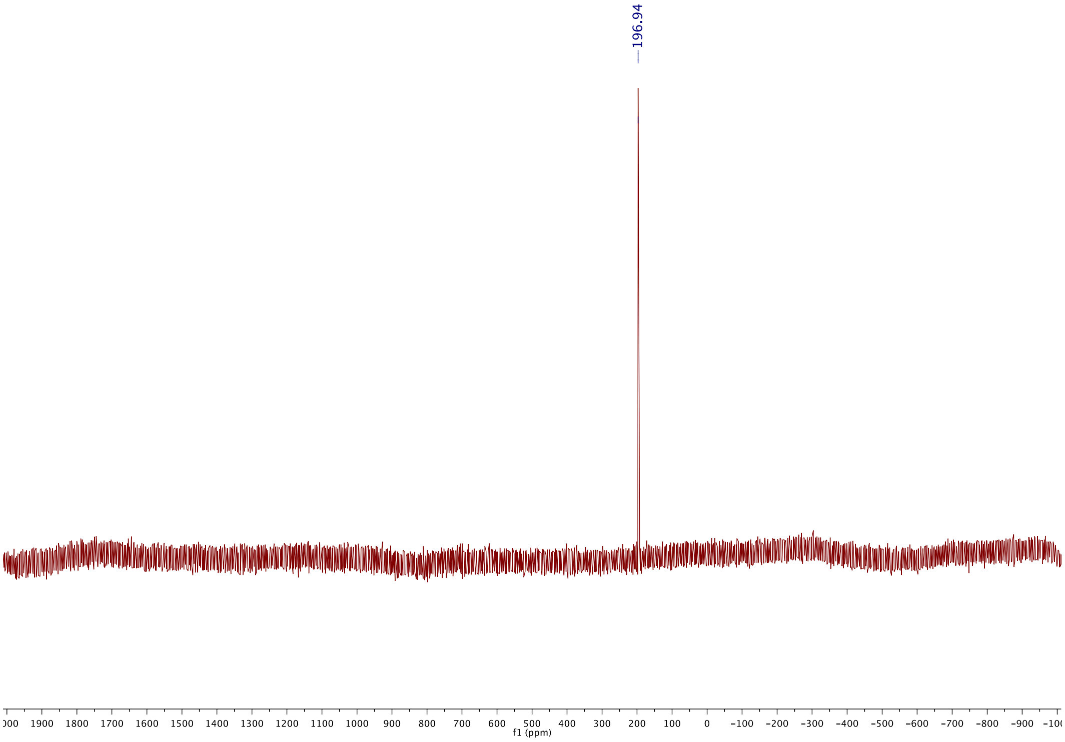

[Se(ICy)]

^1H NMR in $CDCl_3$

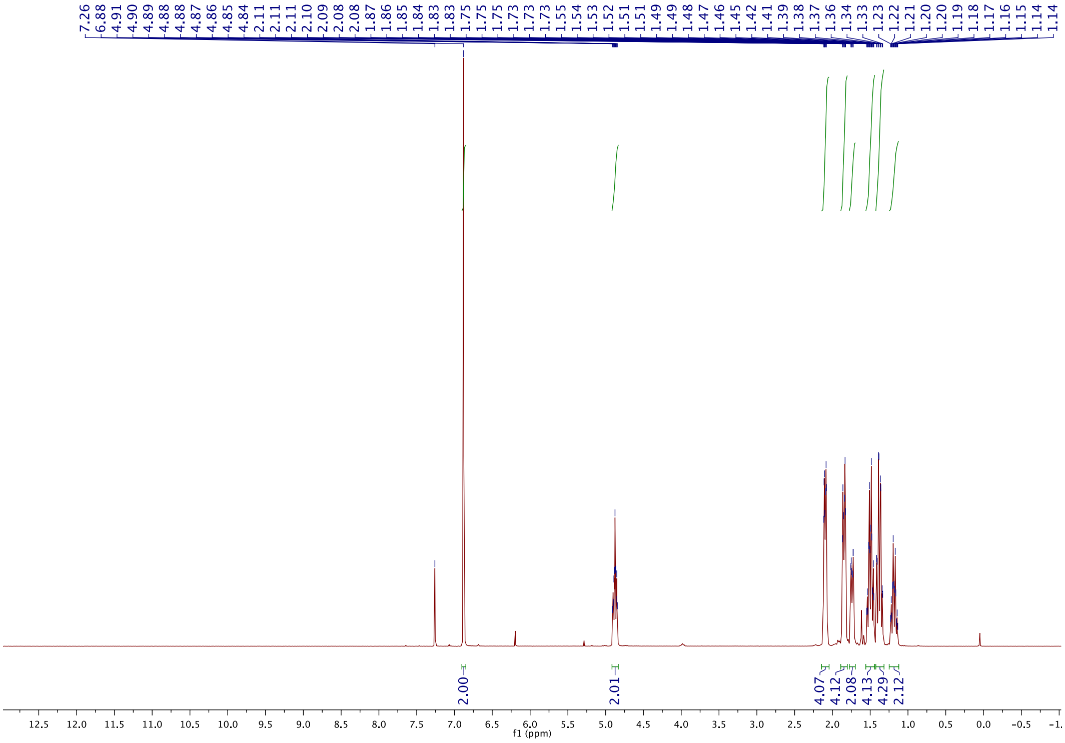

$^{13}C\{^1H\}$ DEPTQ NMR in $CDCl_3$

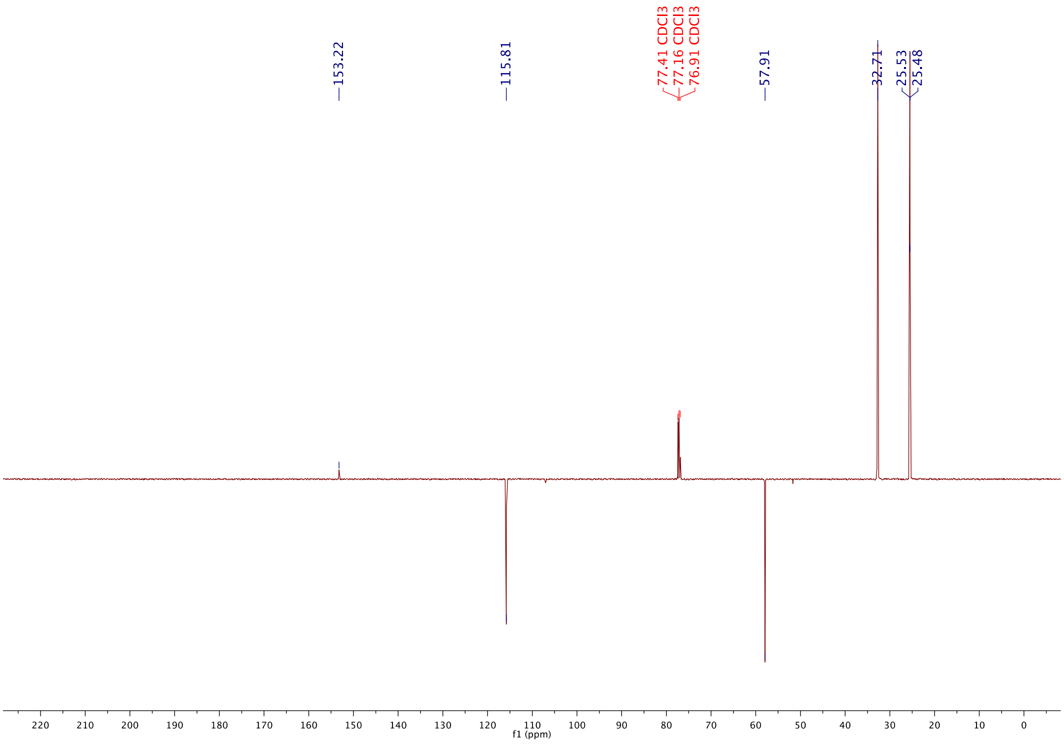

$^{77}Se\{^1H\}$ NMR in $CDCl_3$

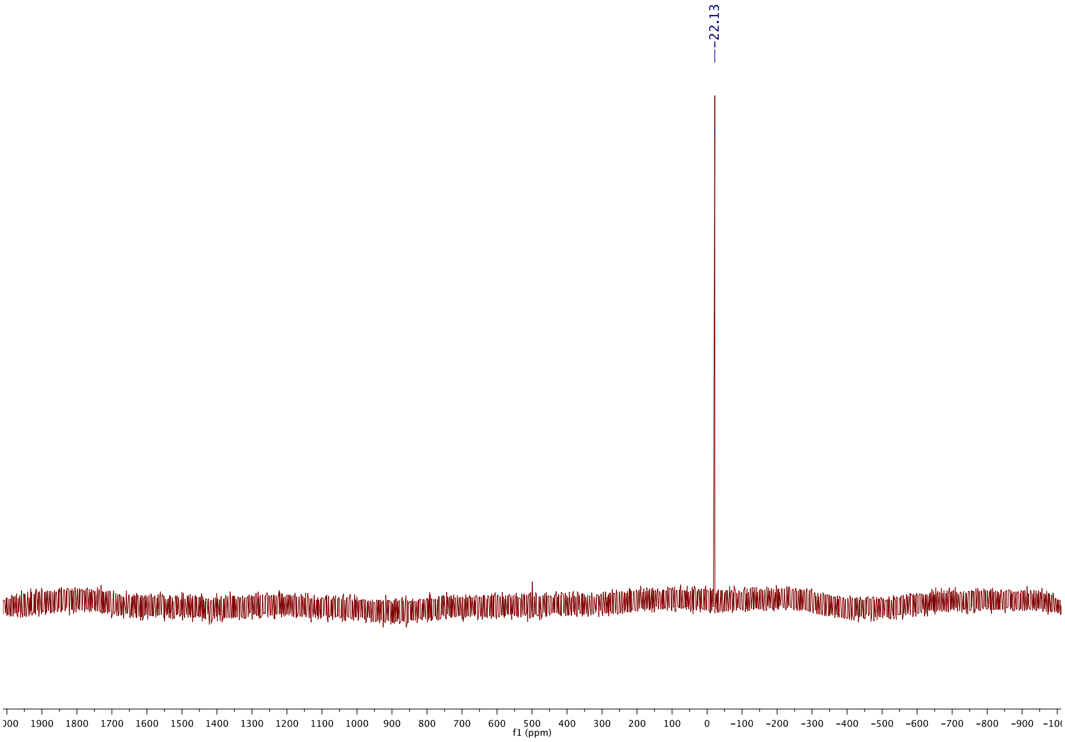

$^{77}Se\{^{1}H\}$ NMR in acetone-d_6

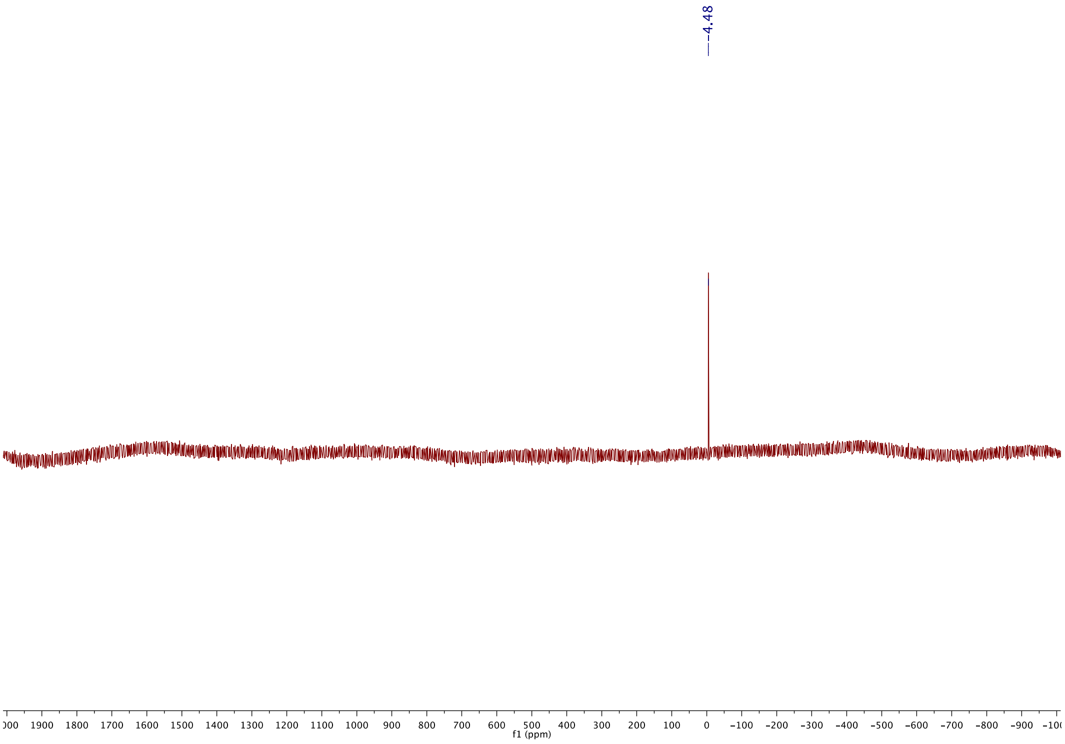

[Se(SIDD)]

^{1}H NMR in $CDCl_3$

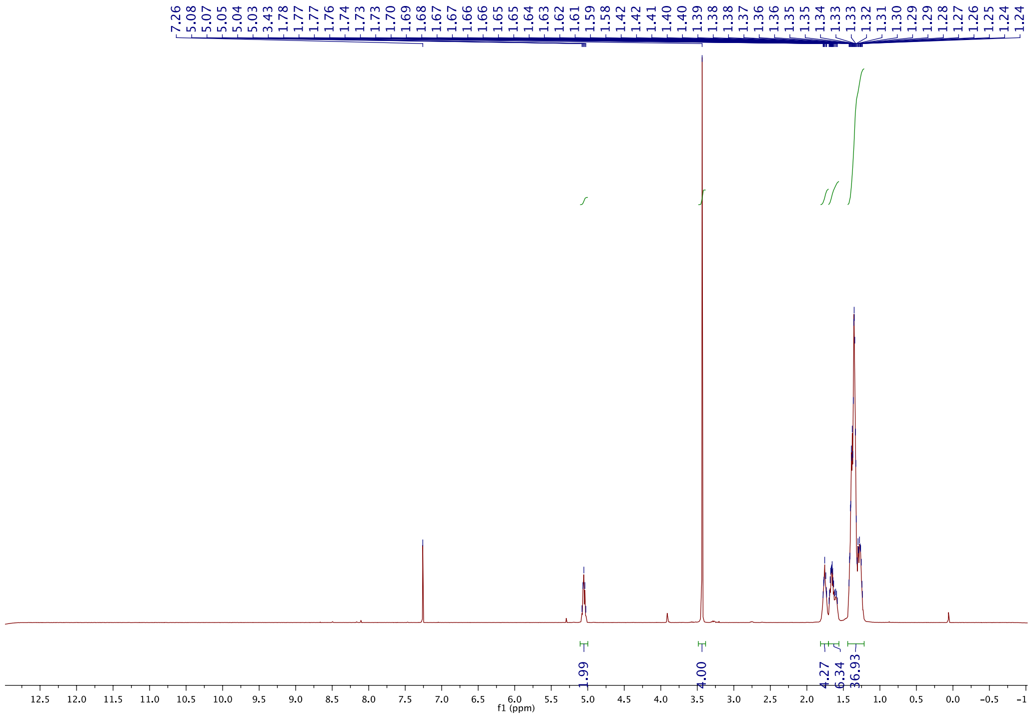

$^{13}C\{^1H\}$ DEPTQ NMR in $CDCl_3$

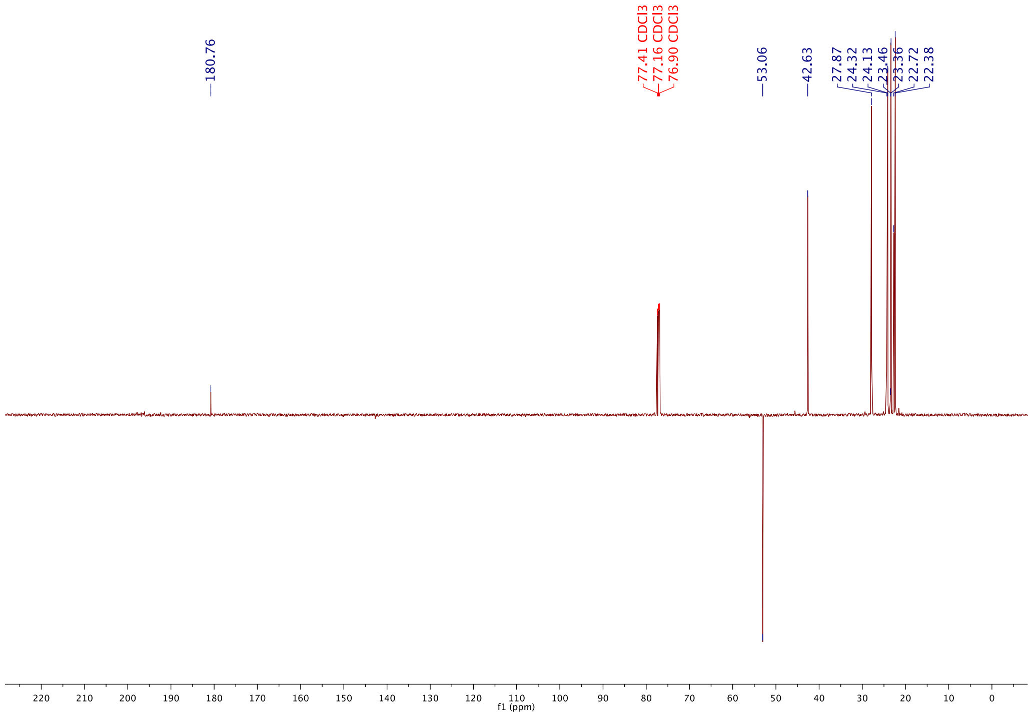

$^{77}Se\{^1H\}$ NMR in $CDCl_3$

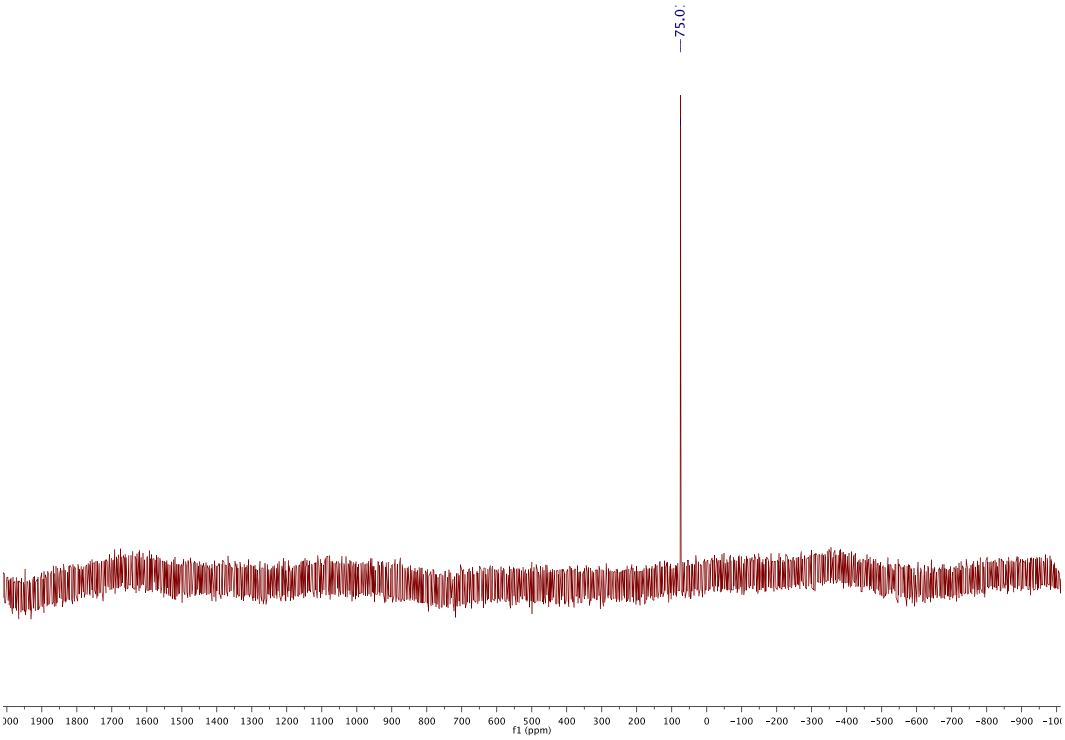

$^{77}Se\{^{1}H\}$ NMR in acetone-d_6

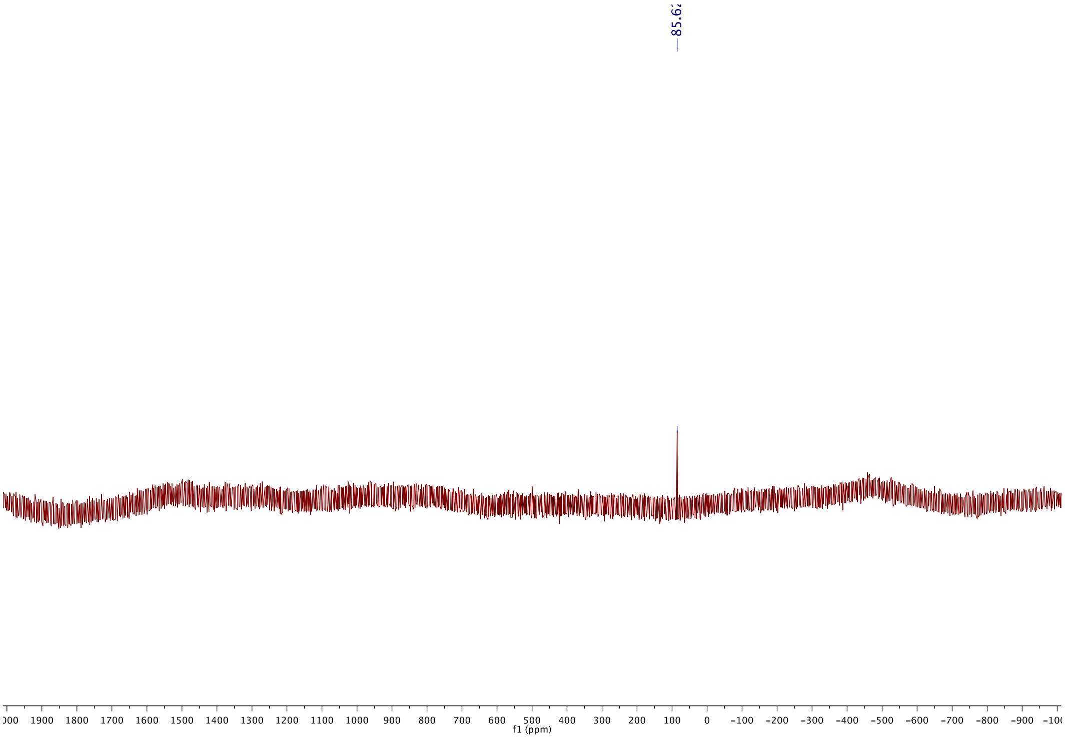

[Se(IHept)]

^{1}H NMR in $CDCl_3$

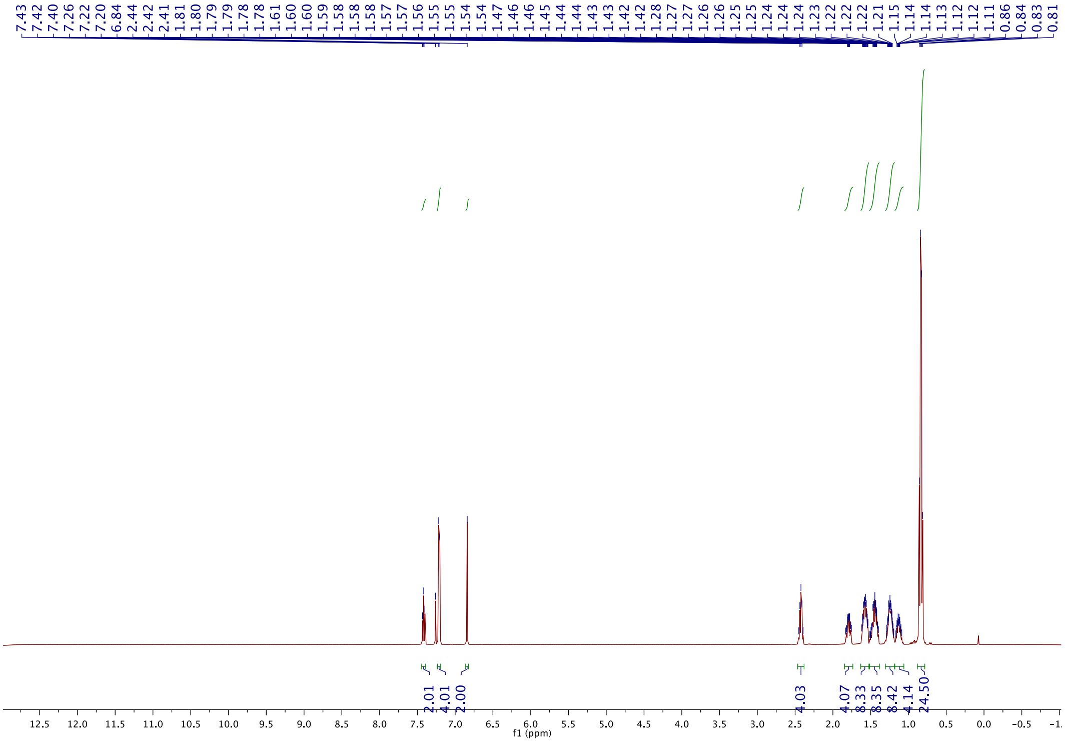

$^{13}C\{^{1}H\}$ DEPTQ NMR in $CDCl_3$

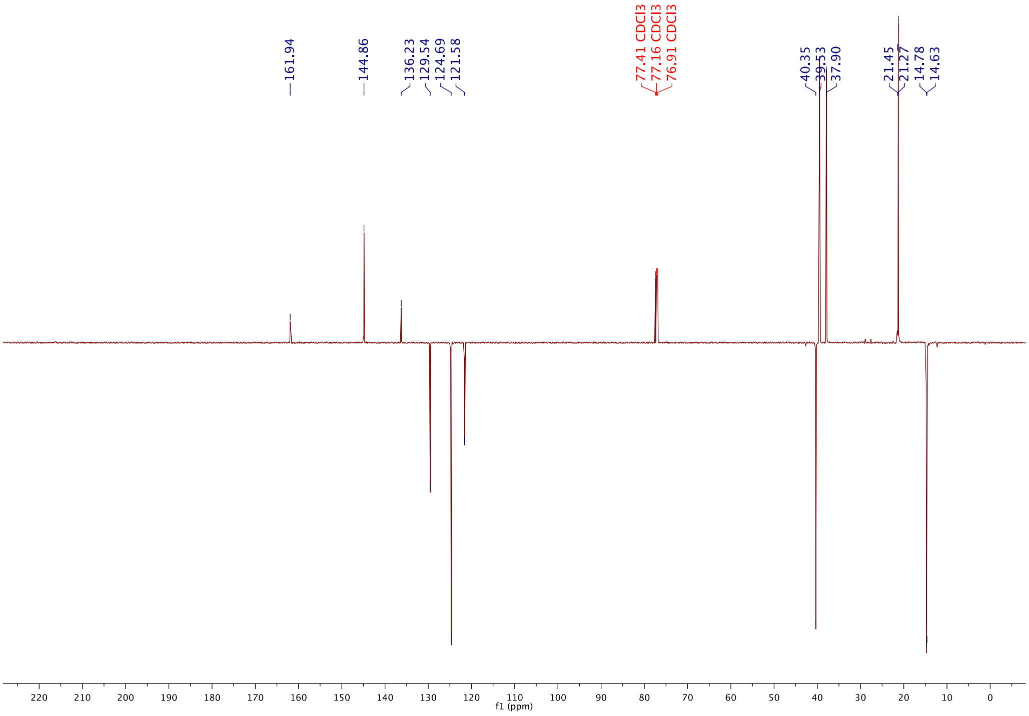

$^{77}Se\{^{1}H\}$ NMR in $CDCl_3$

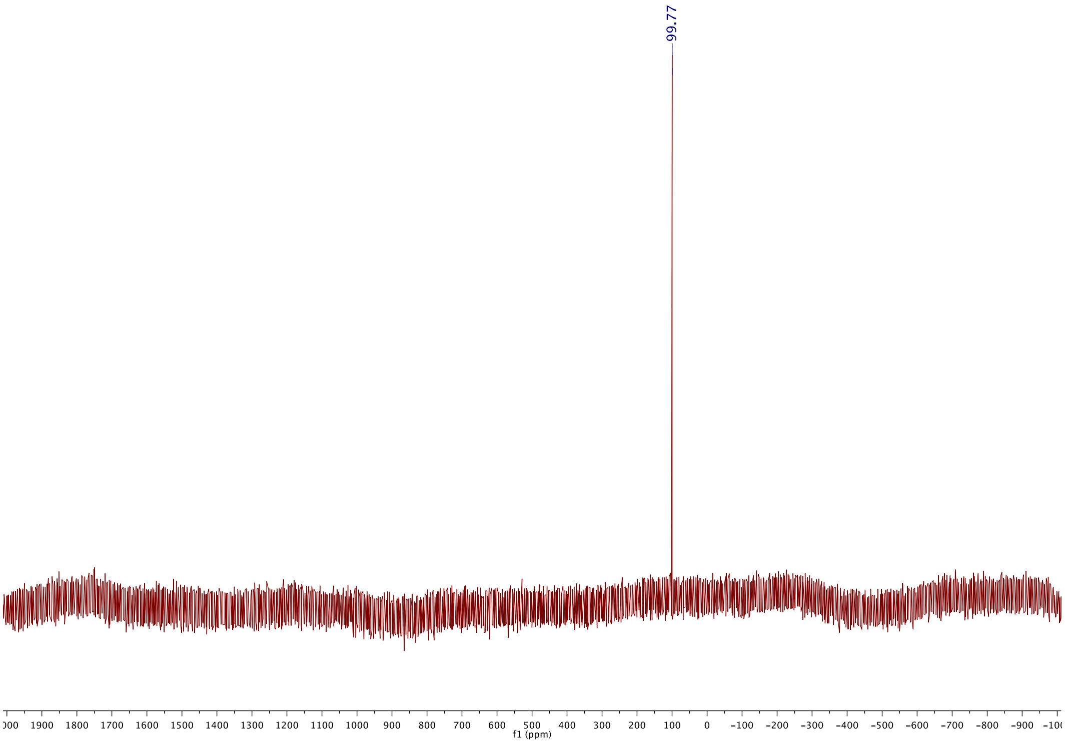

⁷⁷Se{¹H} NMR in acetone-d₆

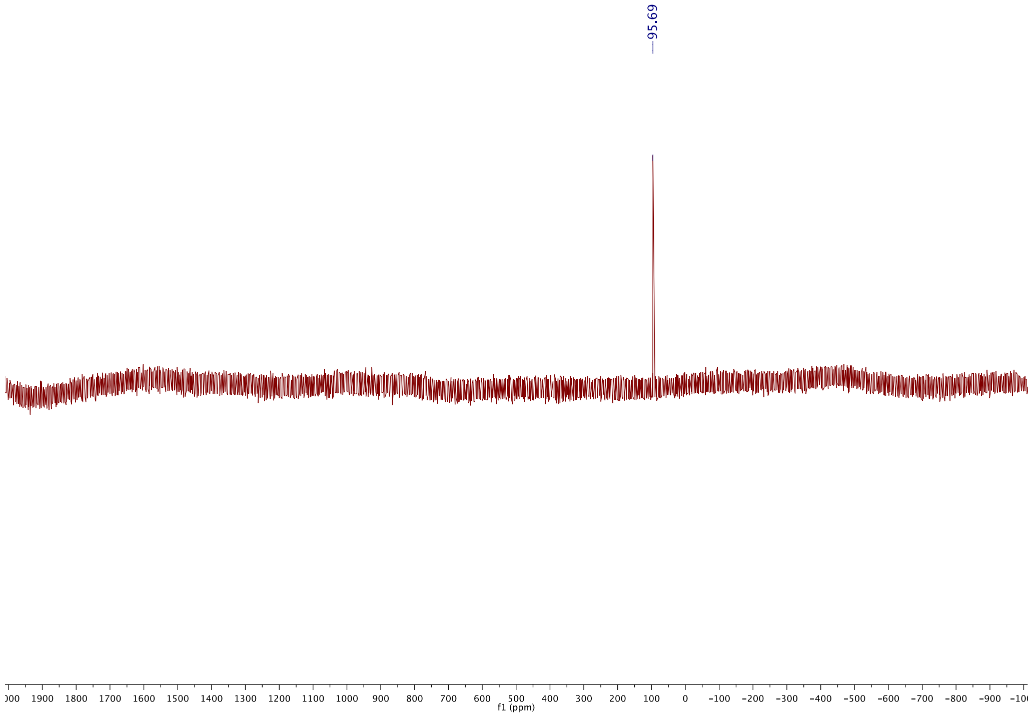

[Se(IMe)]

¹H NMR in $CDCl_3$

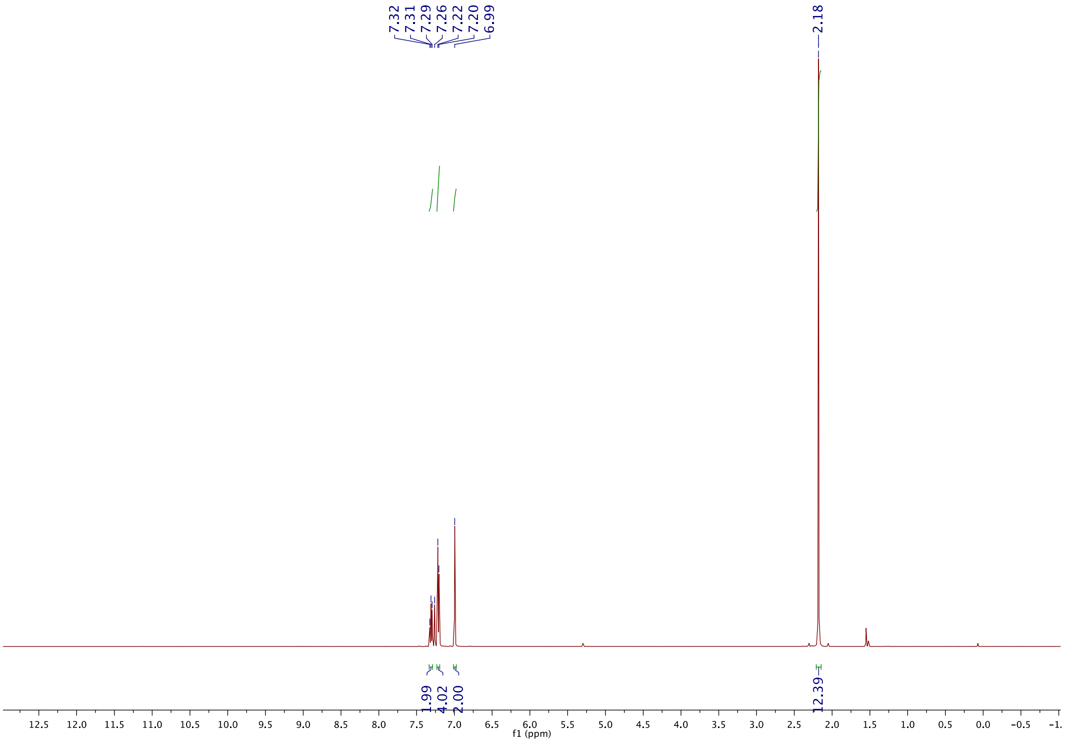

$^{13}C\{^{1}H\}$ DEPTQ NMR in $CDCl_3$

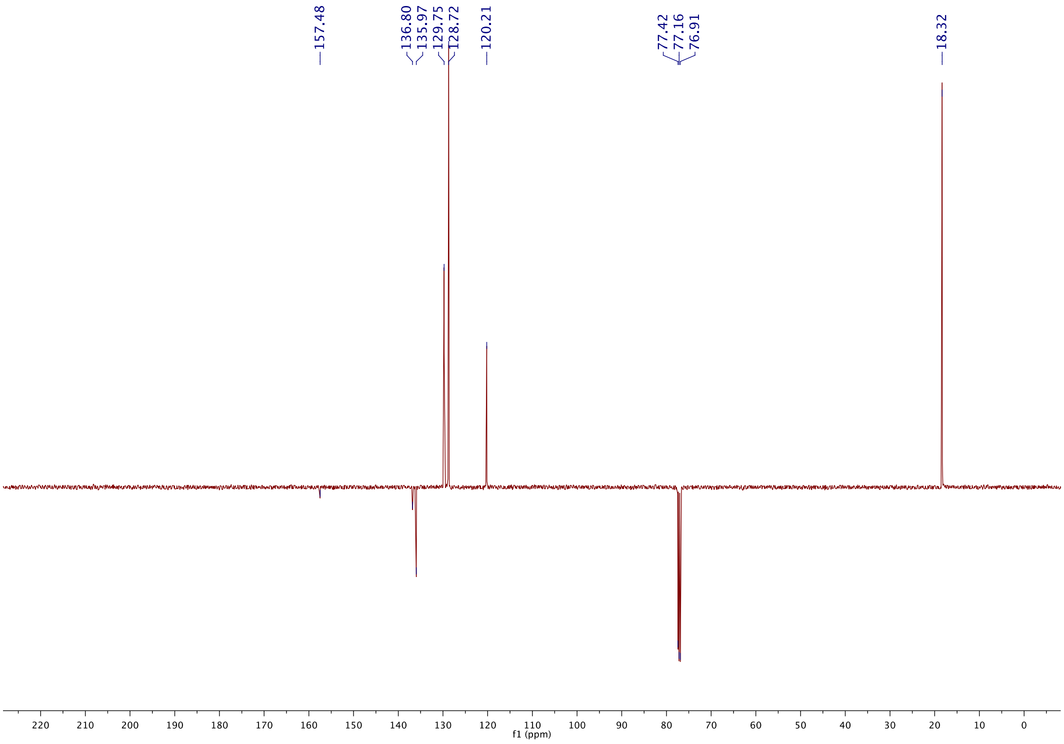

$^{77}Se\{^{1}H\}$ NMR in $CDCl_3$

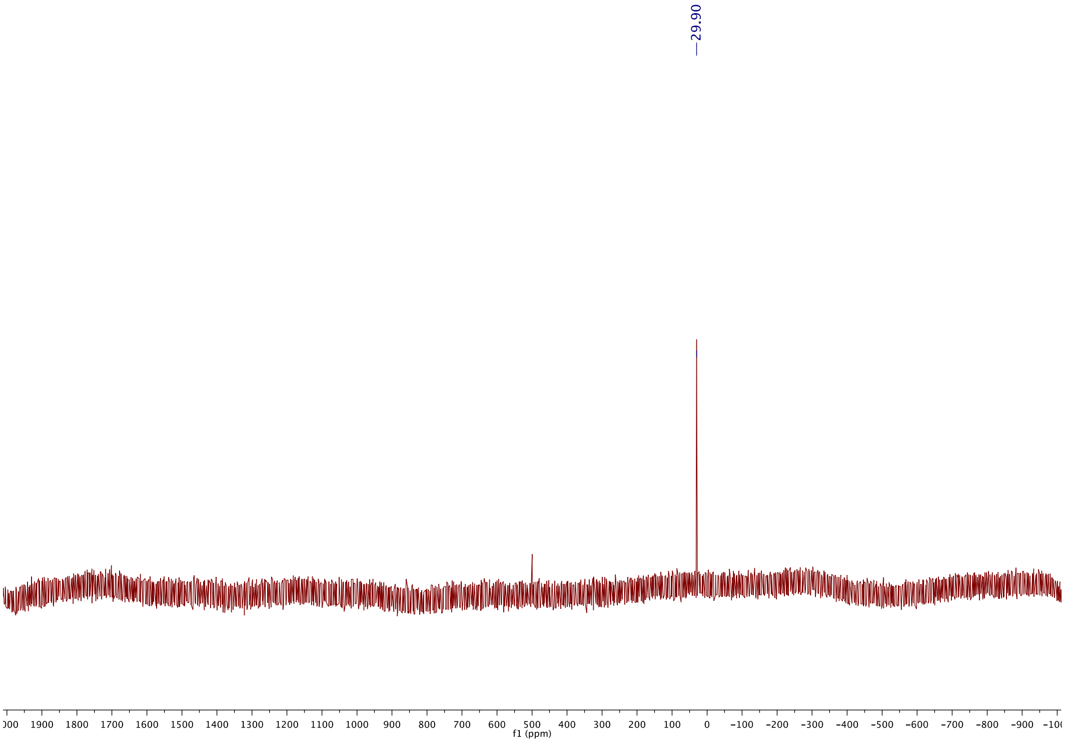

[Se(INon)]

^{1}H NMR in $CDCl_3$

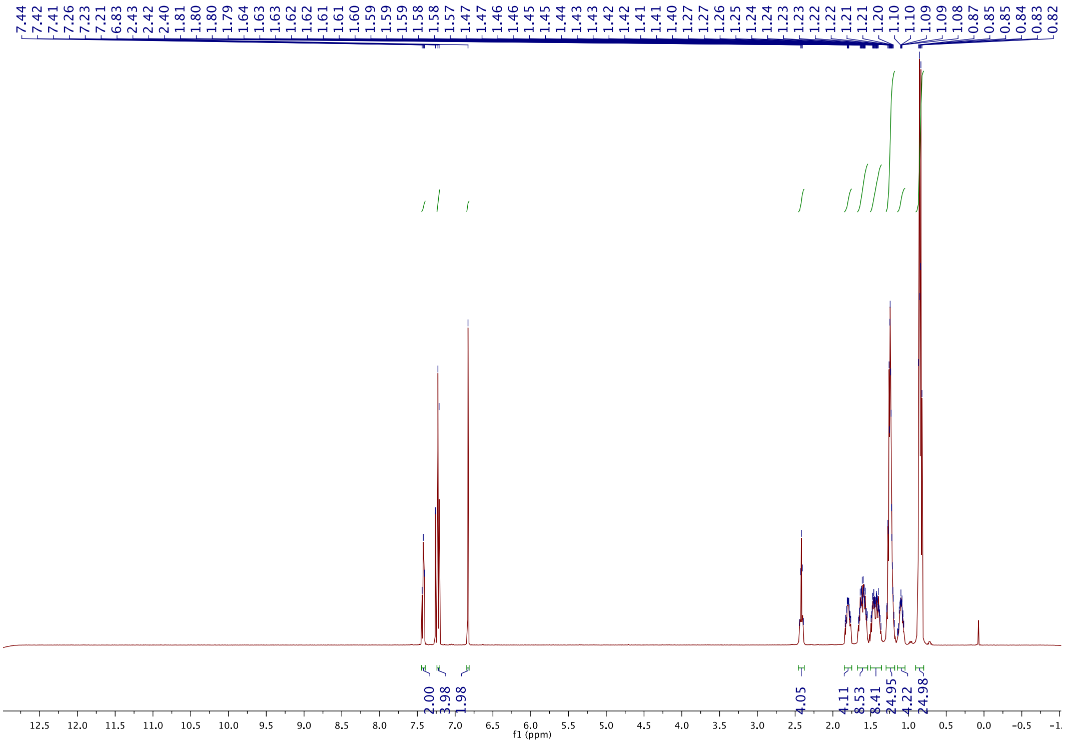

^{13}C{^{1}H} DEPTQ NMR in $CDCl_3$

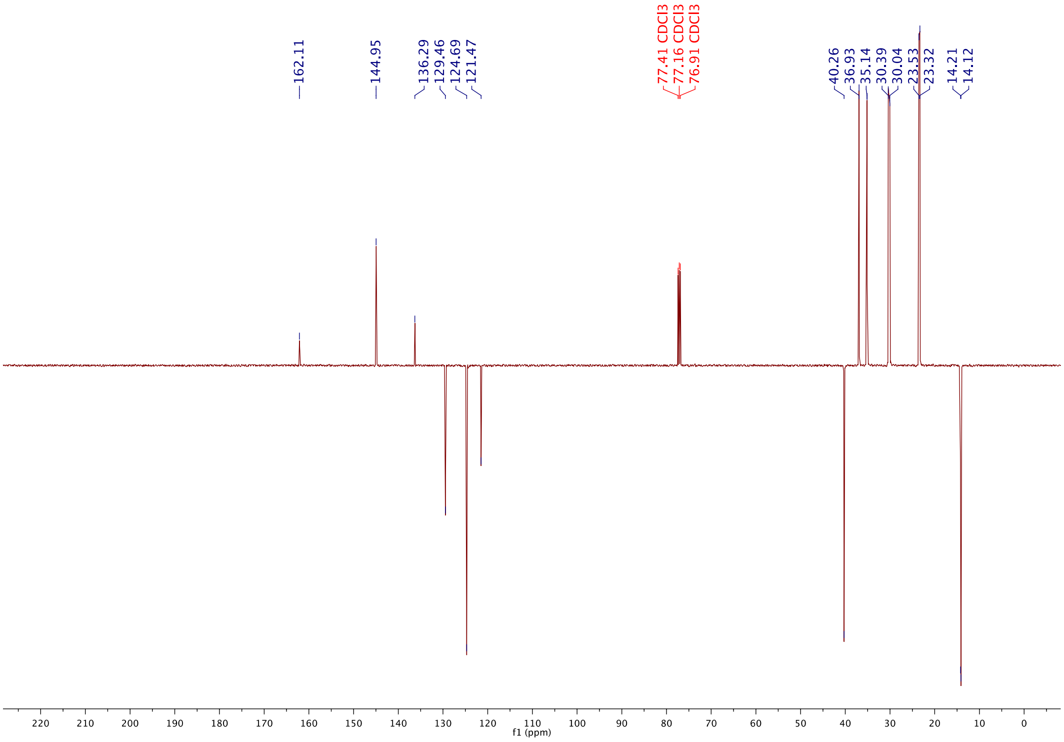

$^{77}Se\{^{1}H\}$ NMR in $CDCl_3$

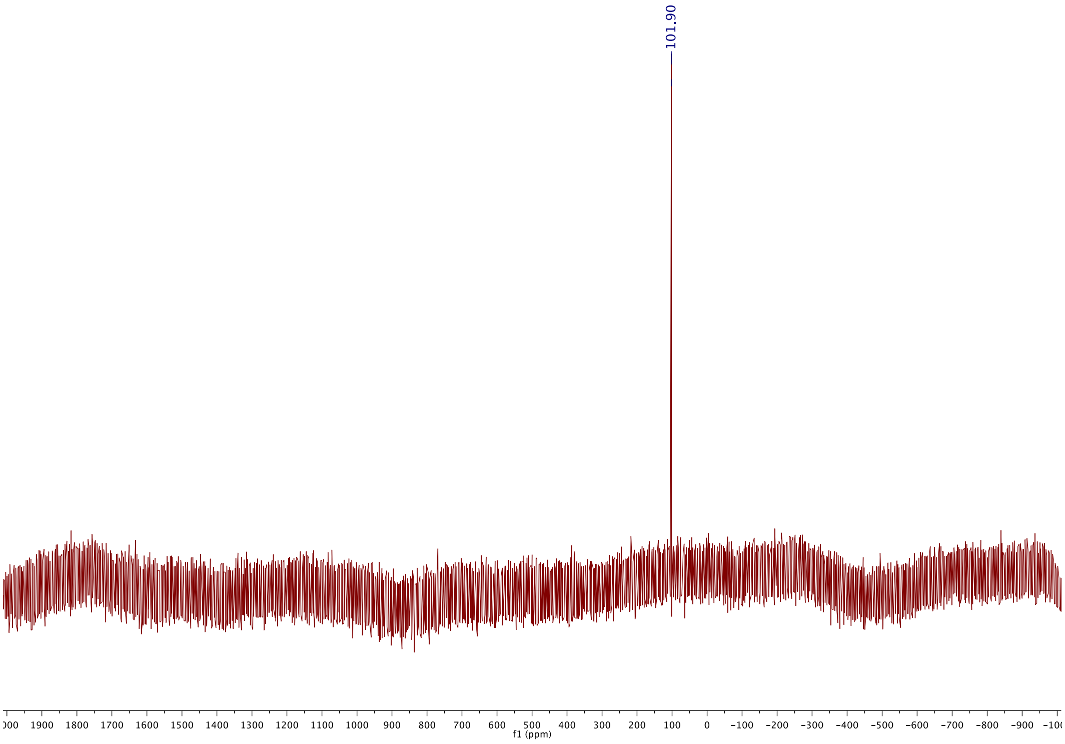

$^{77}Se\{^{1}H\}$ NMR in acetone-d_6

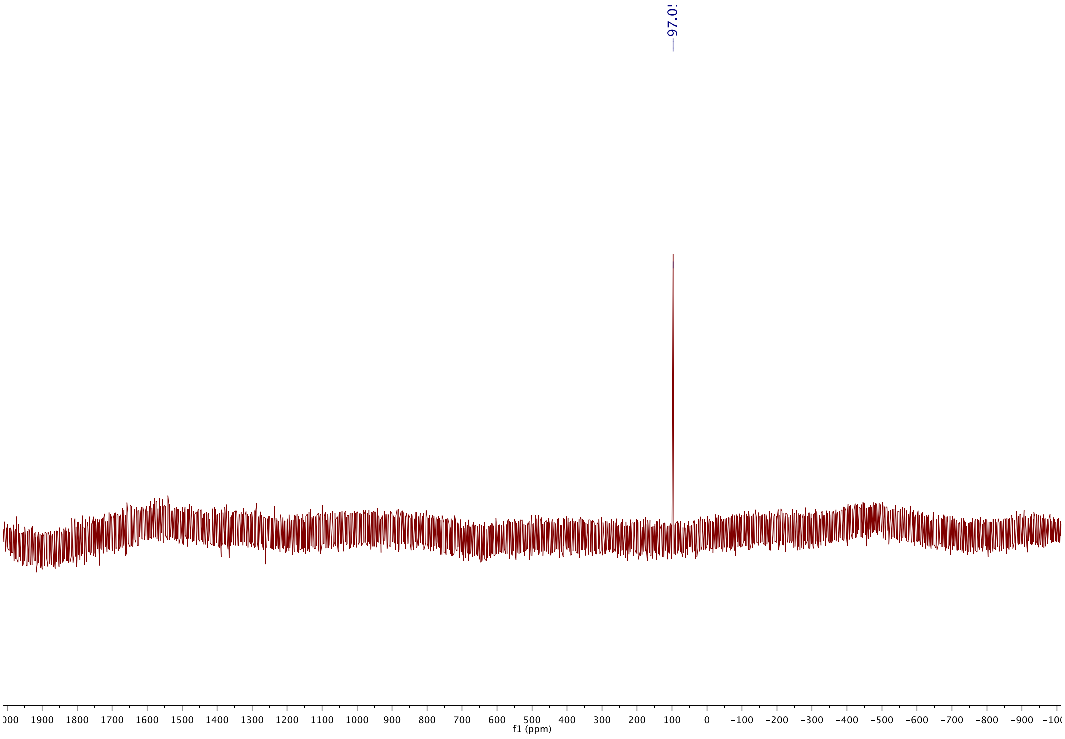

[Se(IPent)]

^{1}H NMR in $CDCl_3$

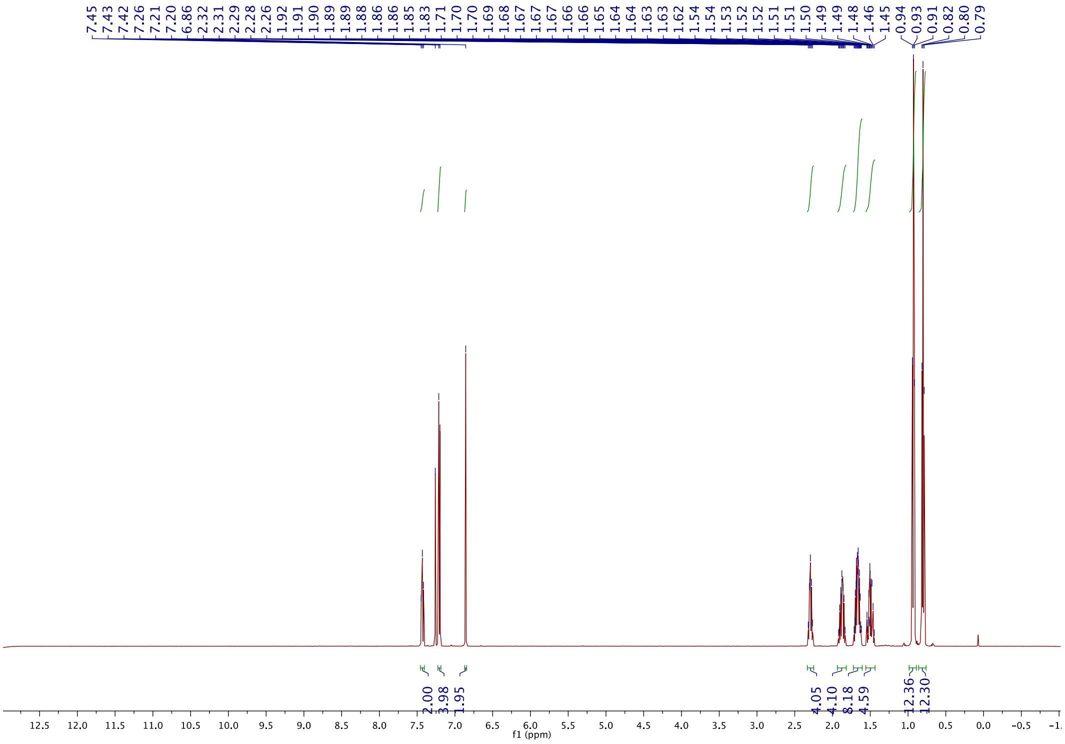

$^{13}C\{^{1}H\}$ DEPTQ NMR in $CDCl_3$

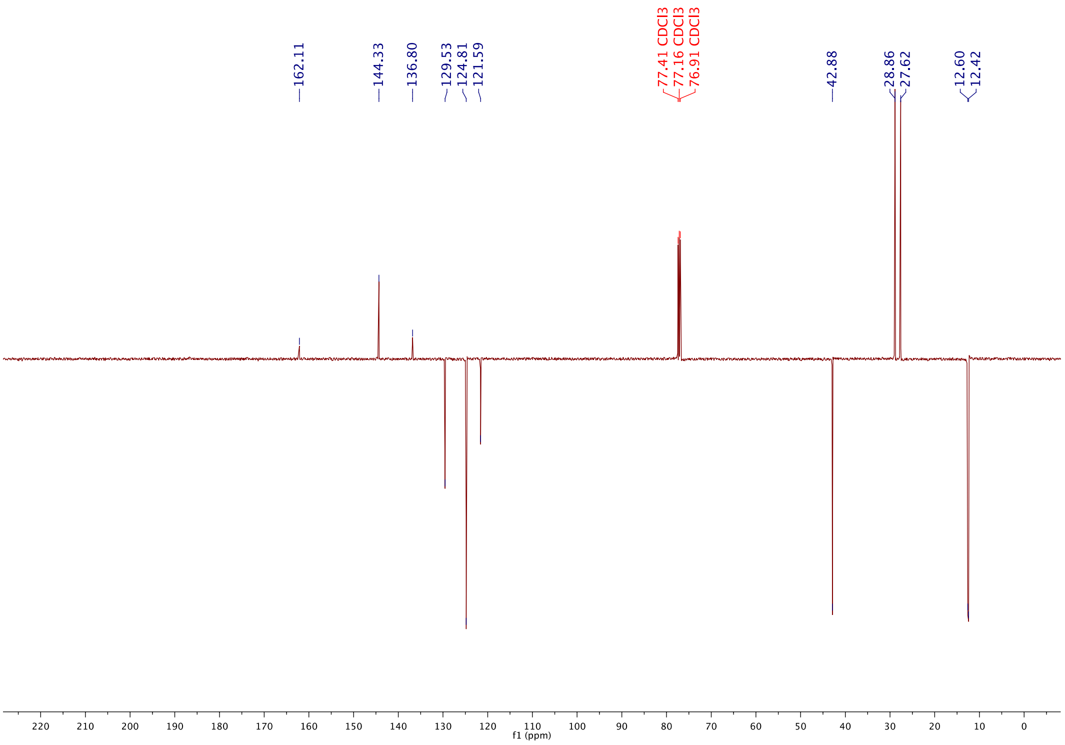

$^{77}Se\{^1H\}$ NMR in $CDCl_3$

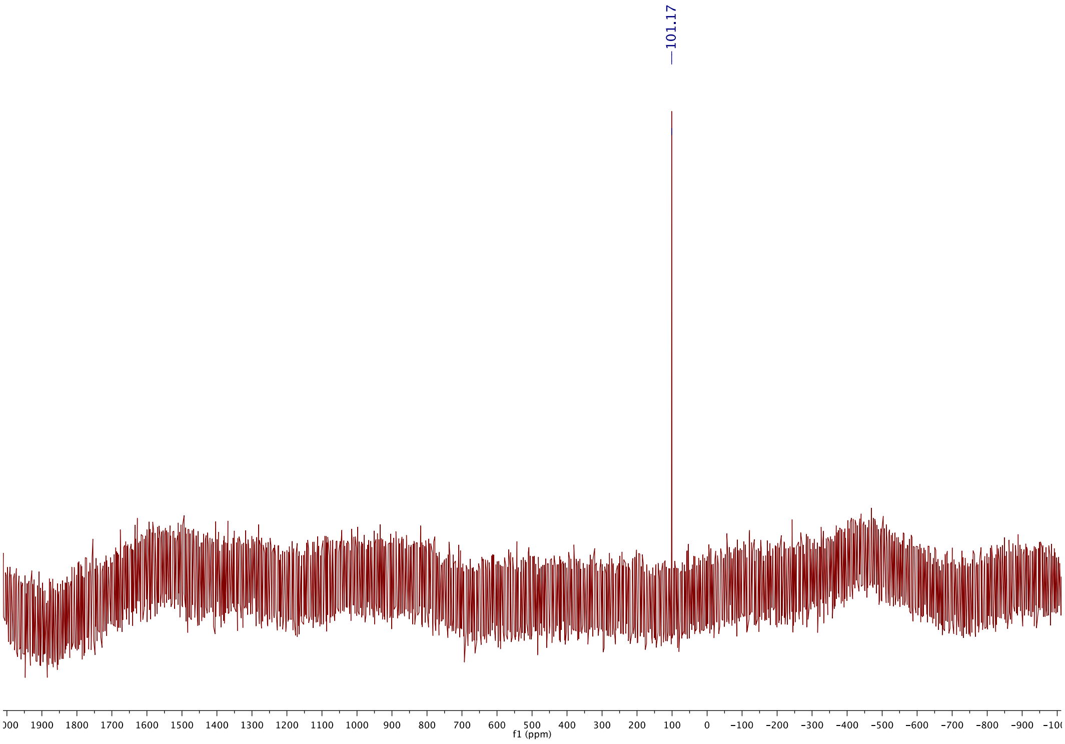

$^{77}Se\{^1H\}$ NMR in acetone-d_6

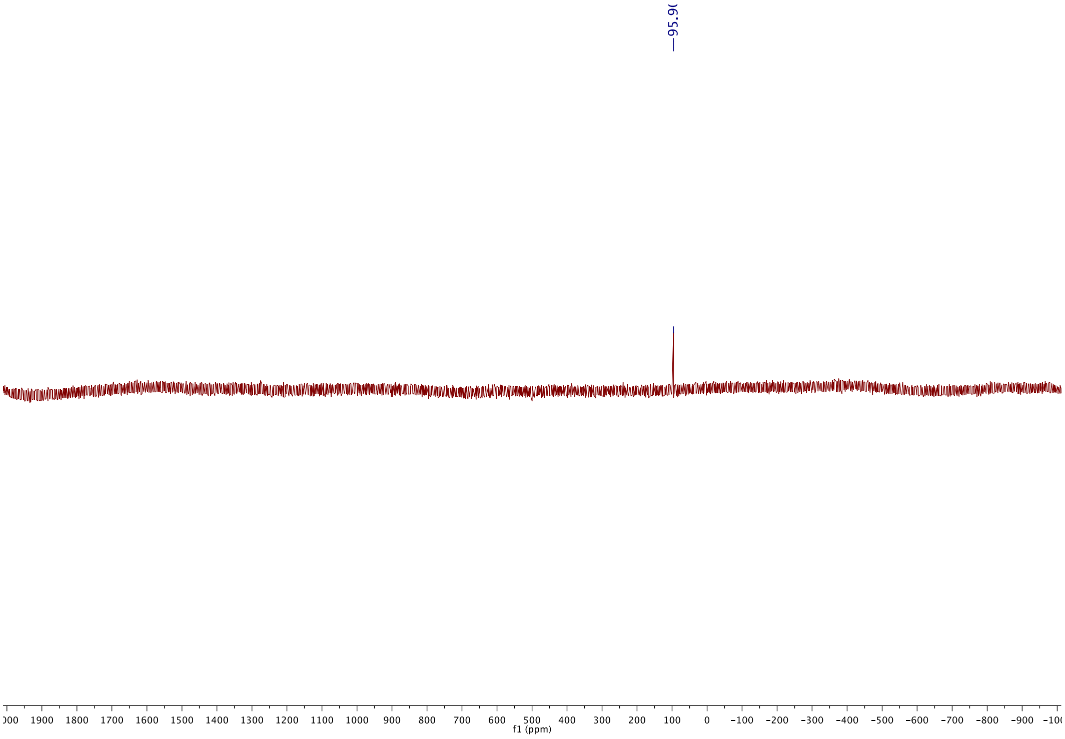

[Se(I^{t}Bu)]

^{1}H NMR in $CDCl_3$

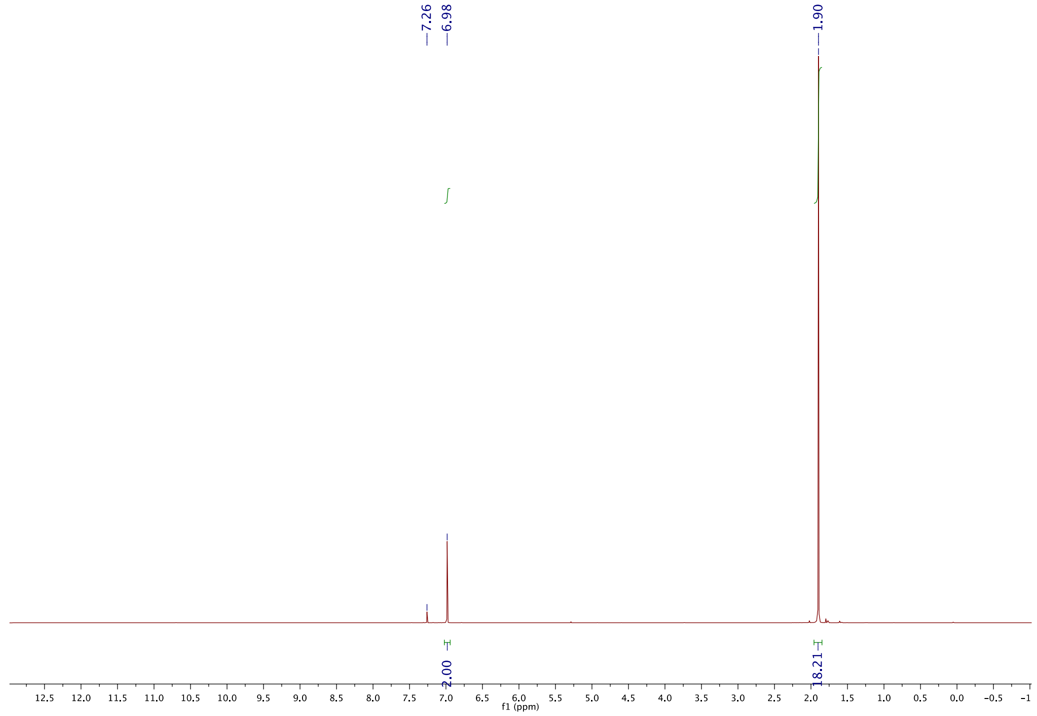

$^{13}C\{^1H\}$ DEPTQ NMR in $CDCl_3$

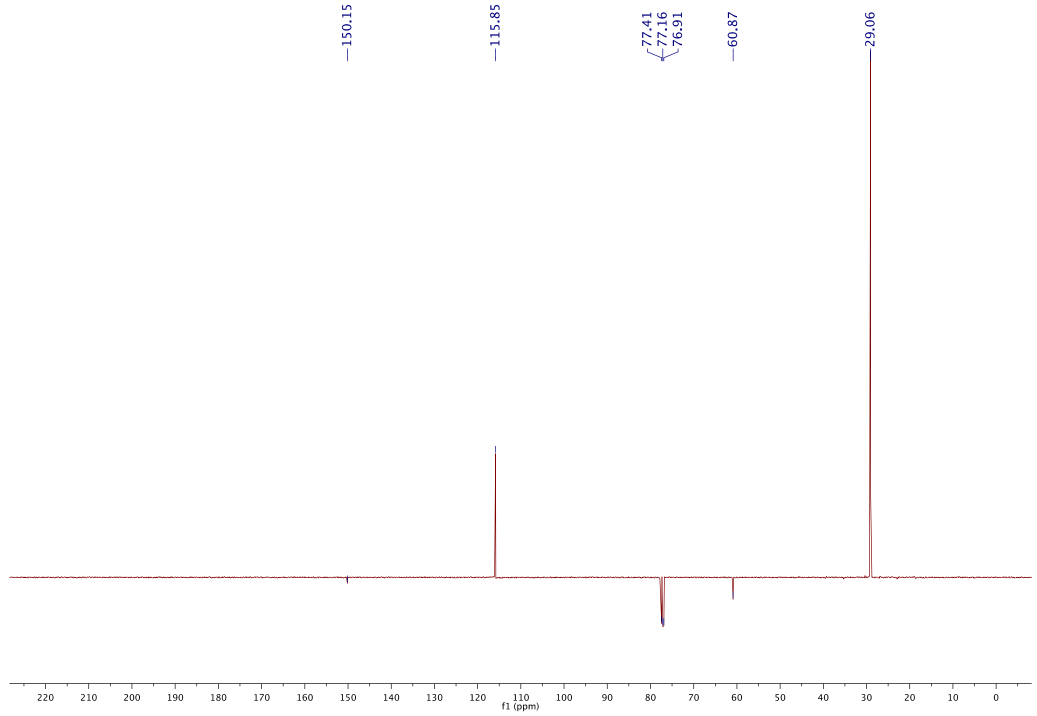

$^{77}Se\{^{1}H\}$ NMR in $CDCl_3$

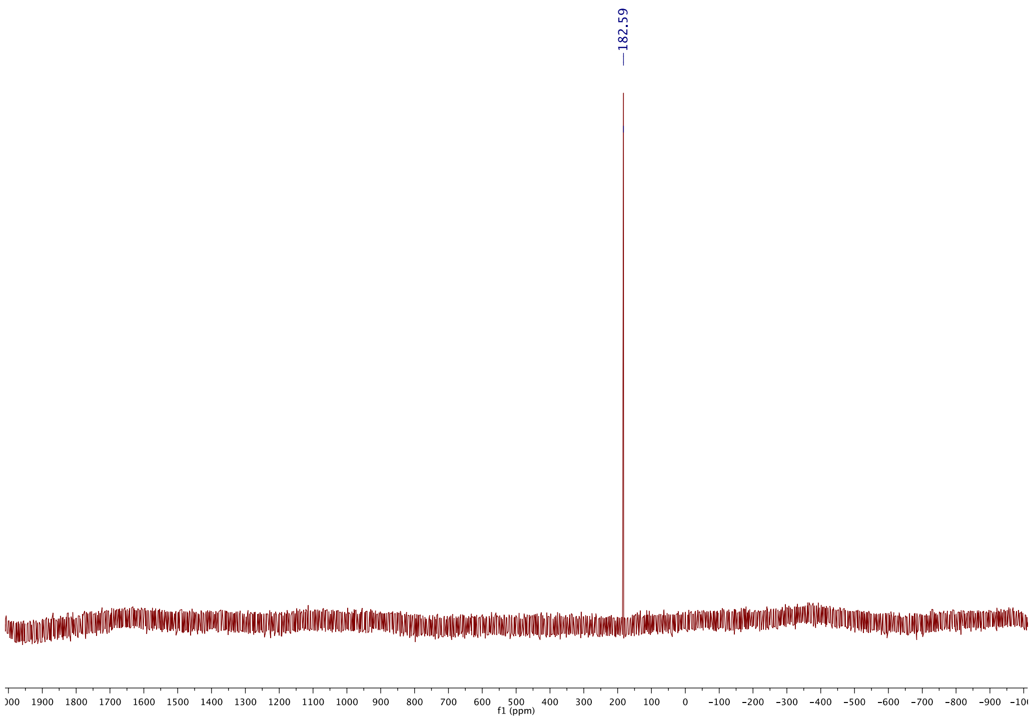

$^{77}Se\{^{1}H\}$ NMR in acetone-d_6

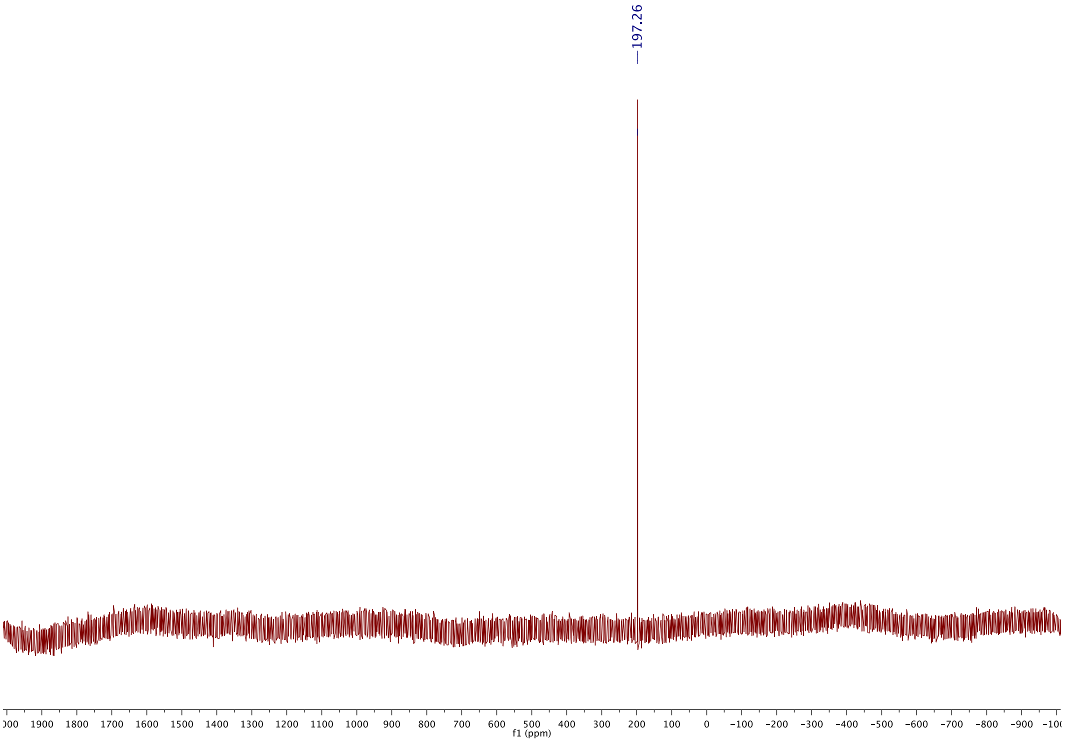

[Se(Tr1)]

^{1}H NMR in $CDCl_3$

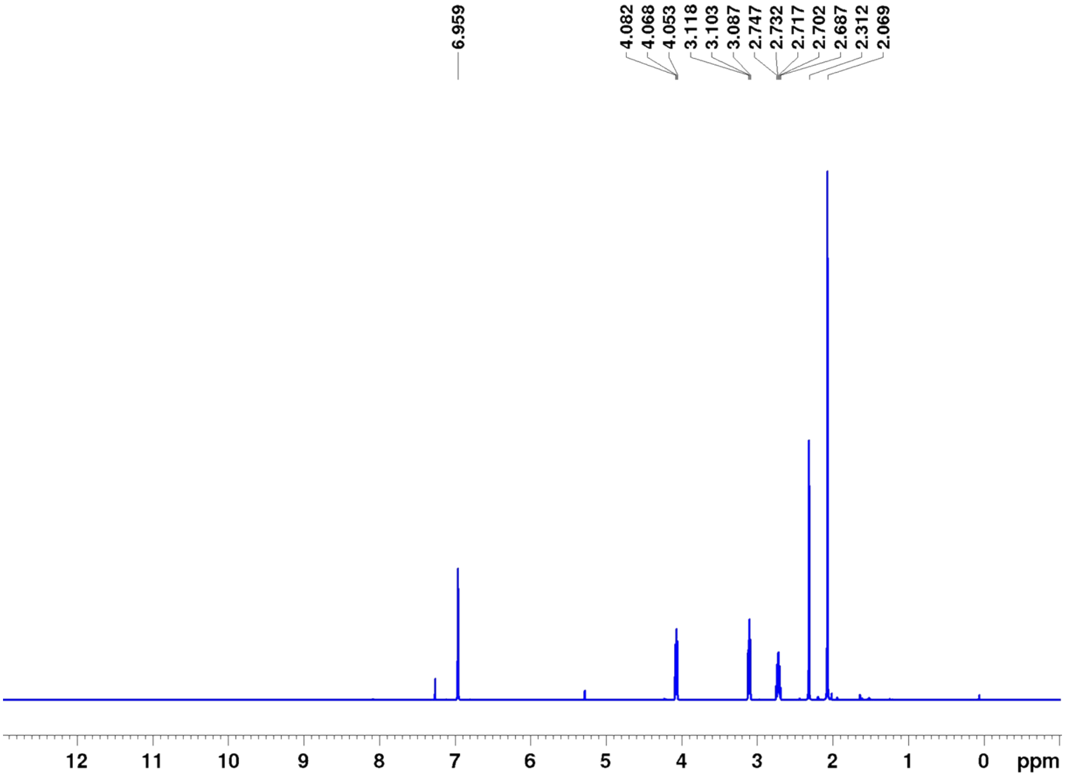

^{13}C{^{1}H} NMR in $CDCl_3$

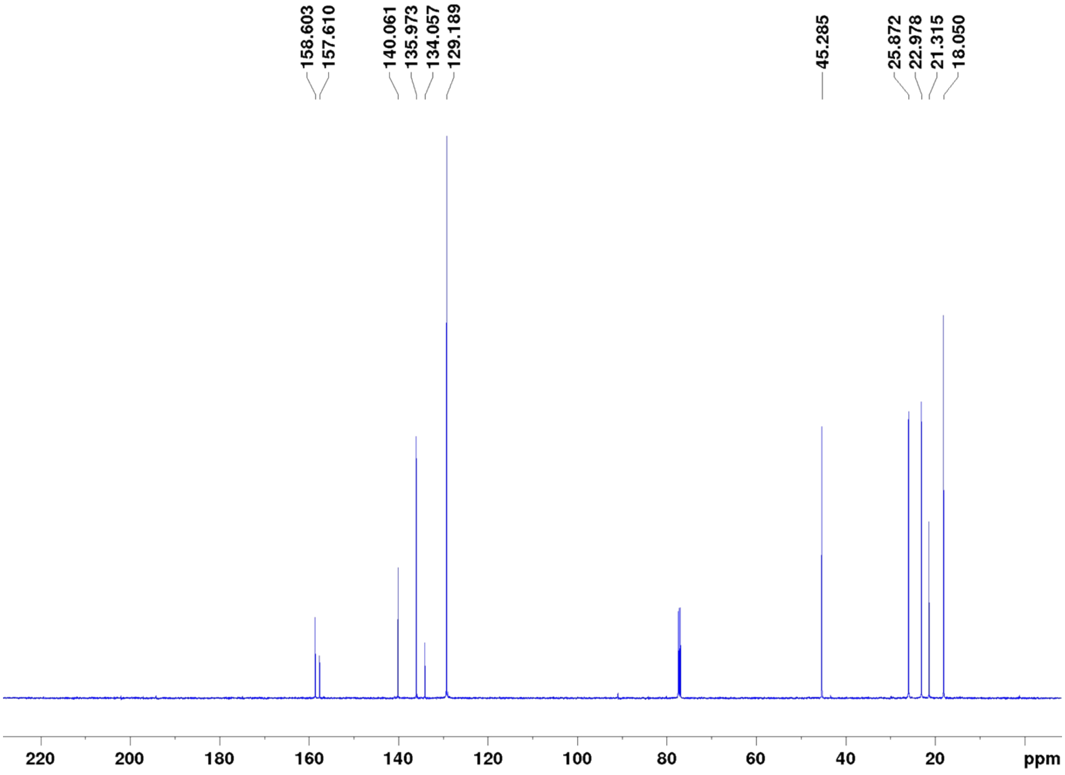

$^{77}Se\{^{1}H\}$ NMR in $CDCl_3$

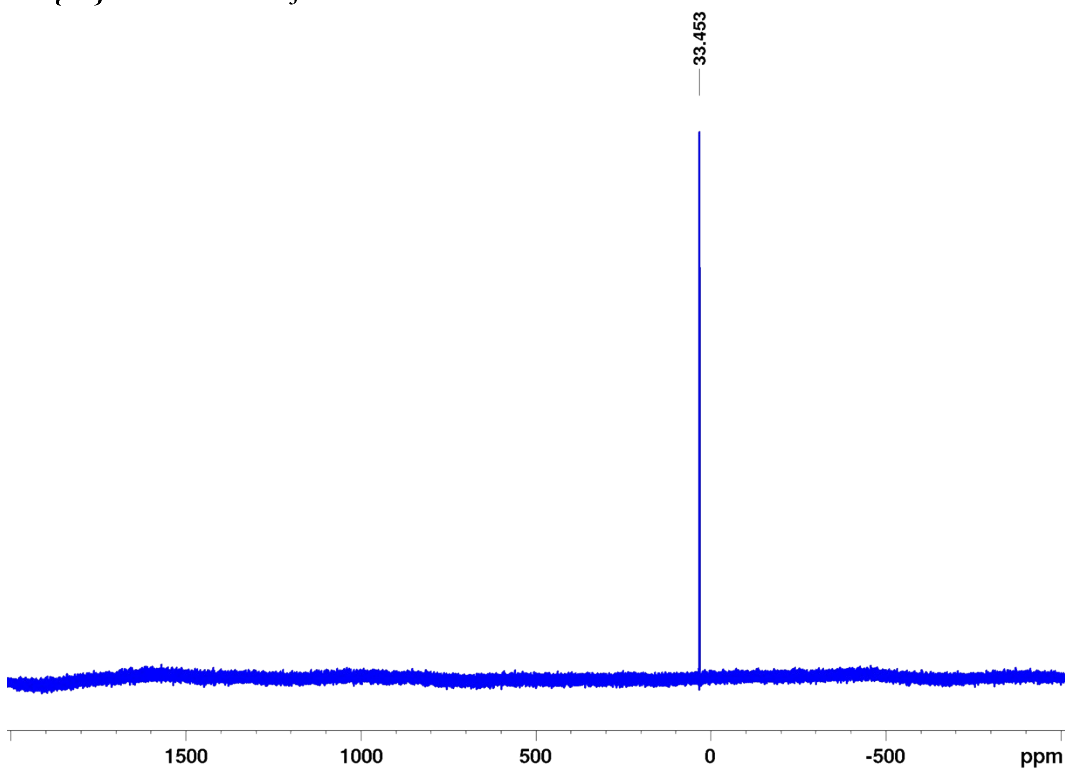

$^{77}Se\{^{1}H\}$ NMR in acetone-d_6

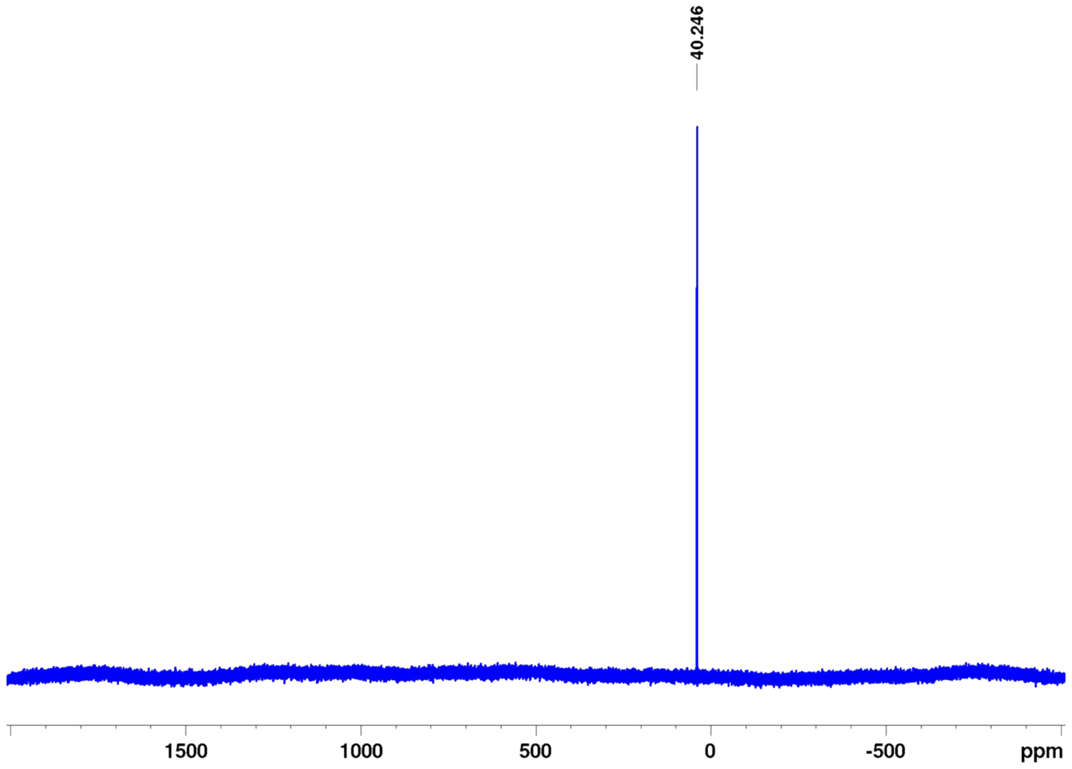

[Se(Tr2)]

^1H NMR in $CDCl_3$

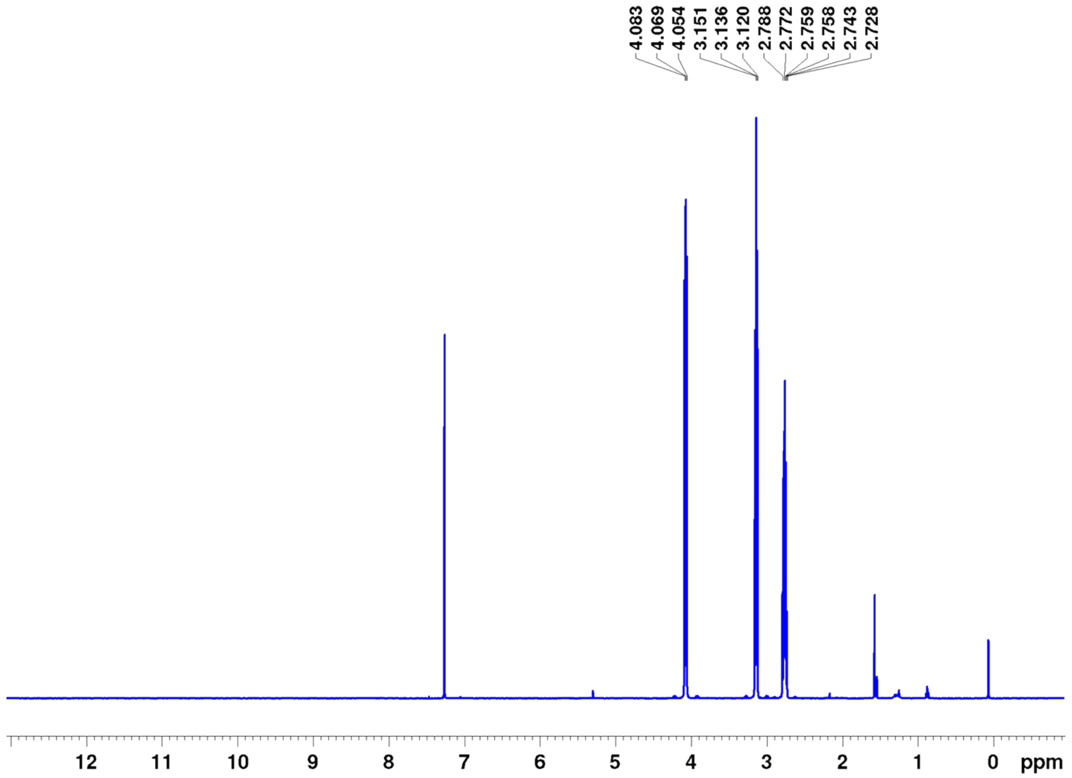

$^{13}C\{^1H\}$ NMR in $CDCl_3$

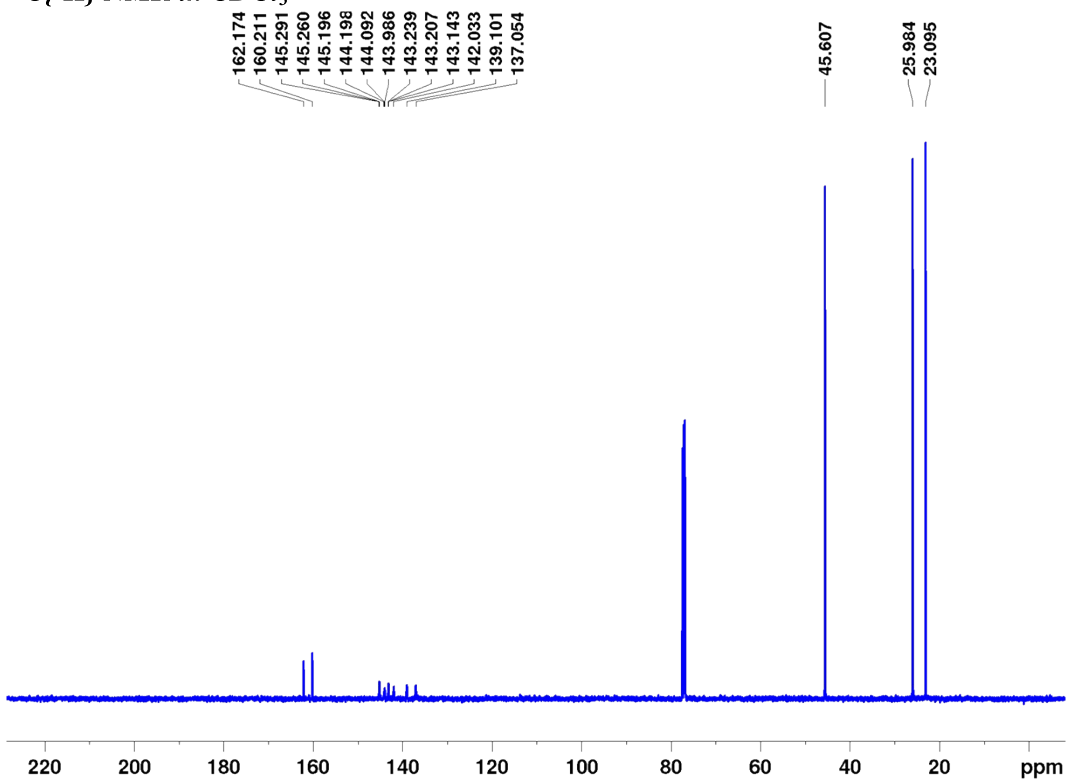

$^{19}F\{^{1}H\}$ NMR in $CDCl_3$

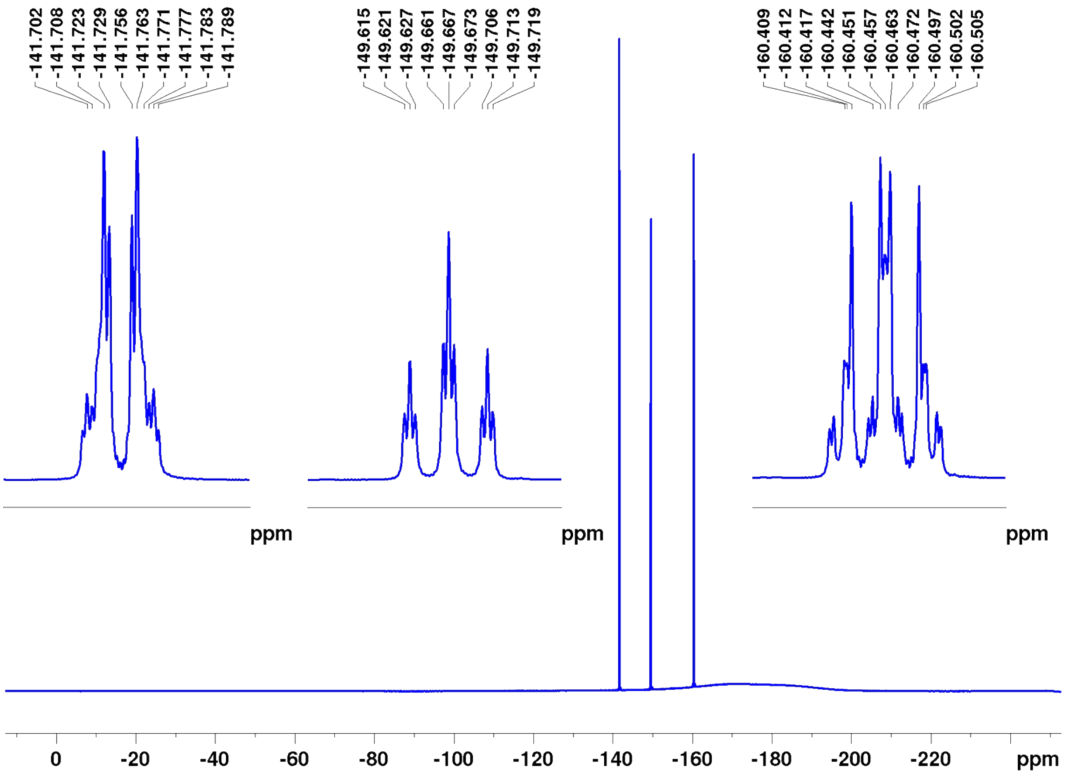

$^{77}Se\{^{1}H\}$ NMR in $CDCl_3$

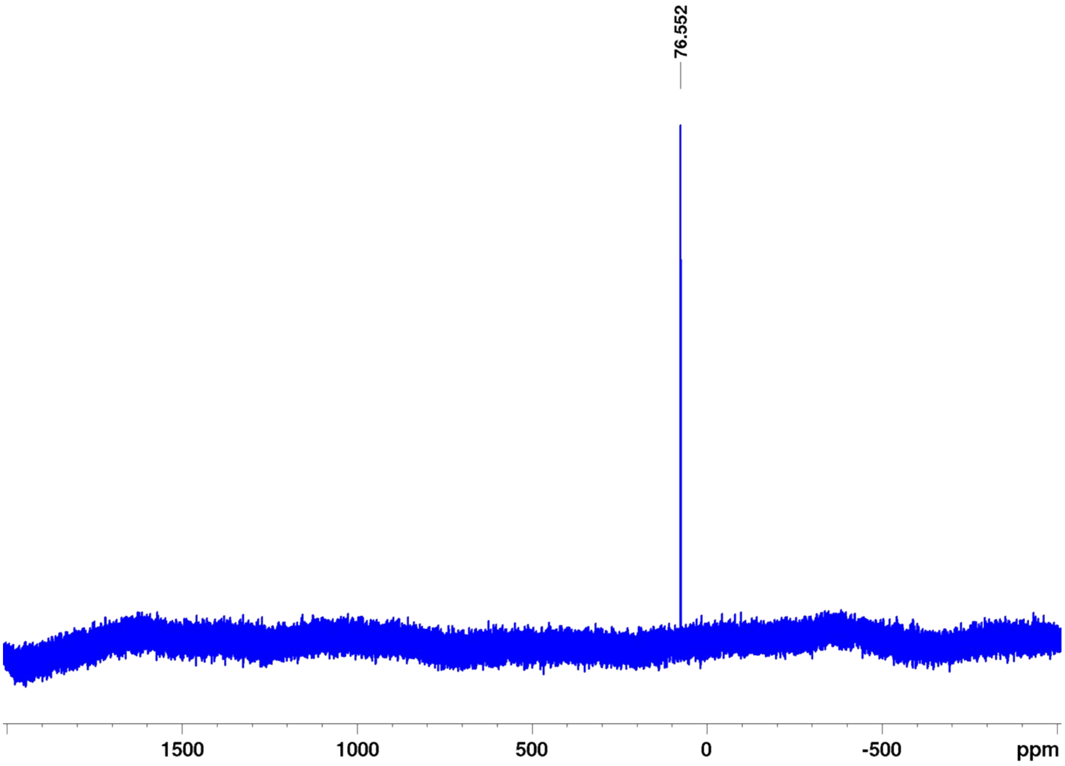

$^{77}Se\{^1H\}$ NMR in acetone-d_6

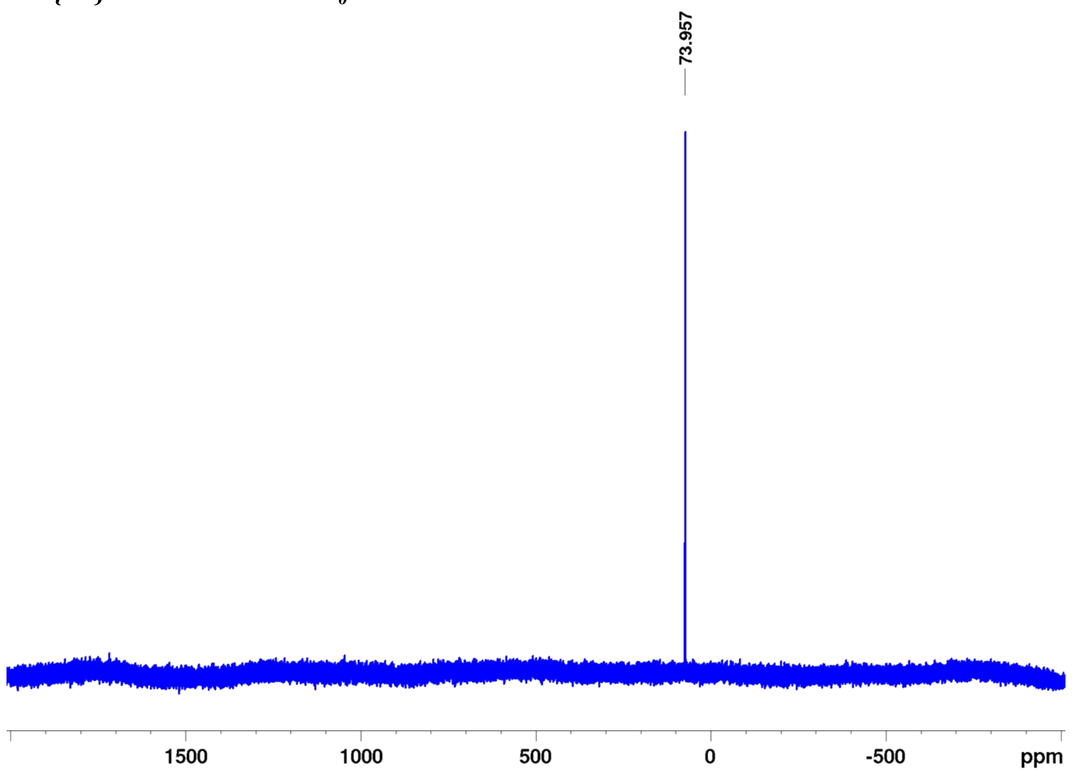

[Se(Tr3)]

^1H NMR in $CDCl_3$

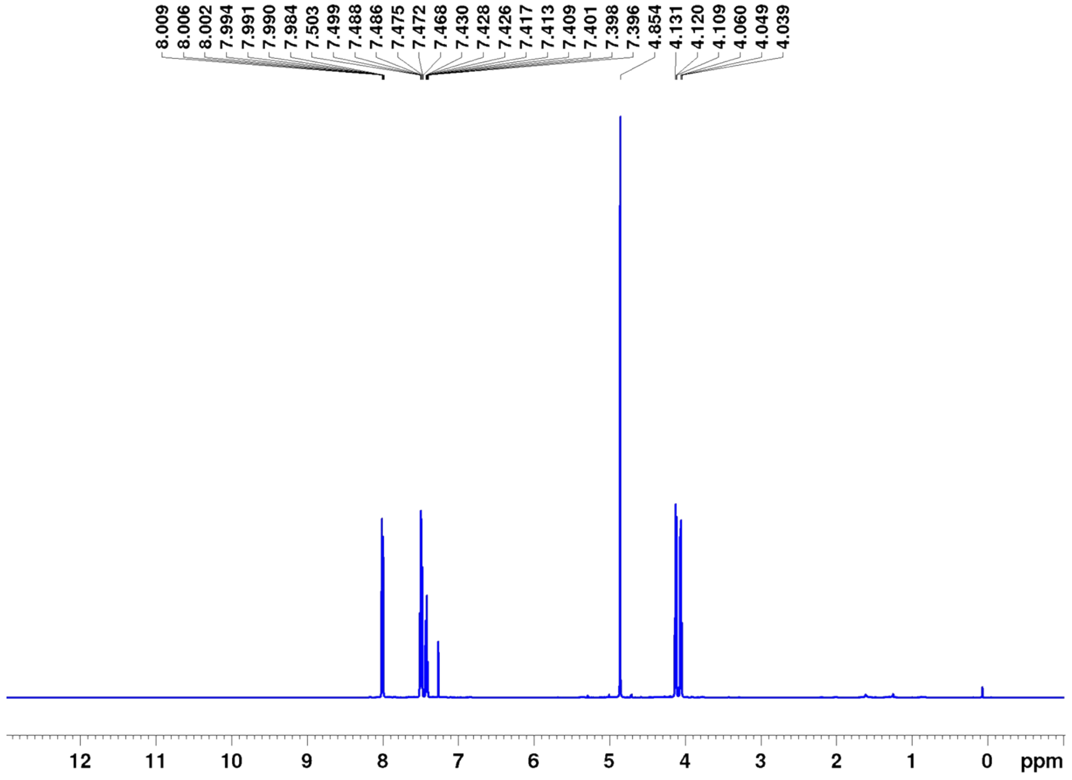

$^{13}C\{^{1}H\}$ NMR in $CDCl_3$

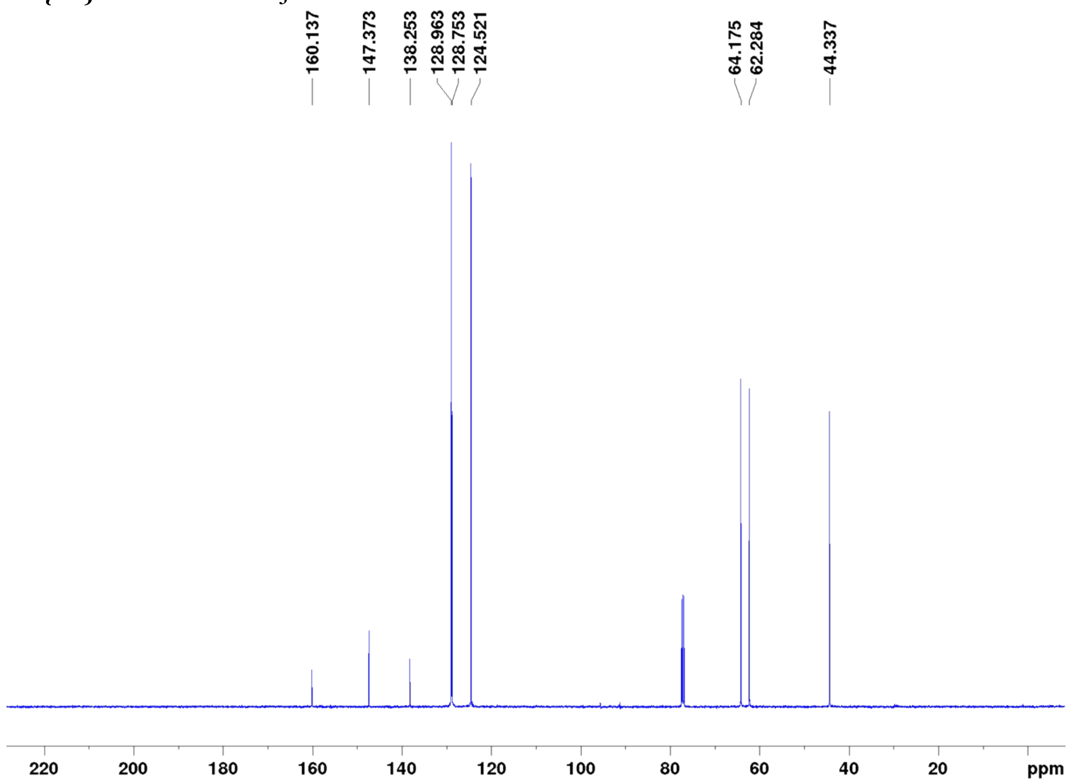

$^{77}Se\{^{1}H\}$ NMR in $CDCl_3$

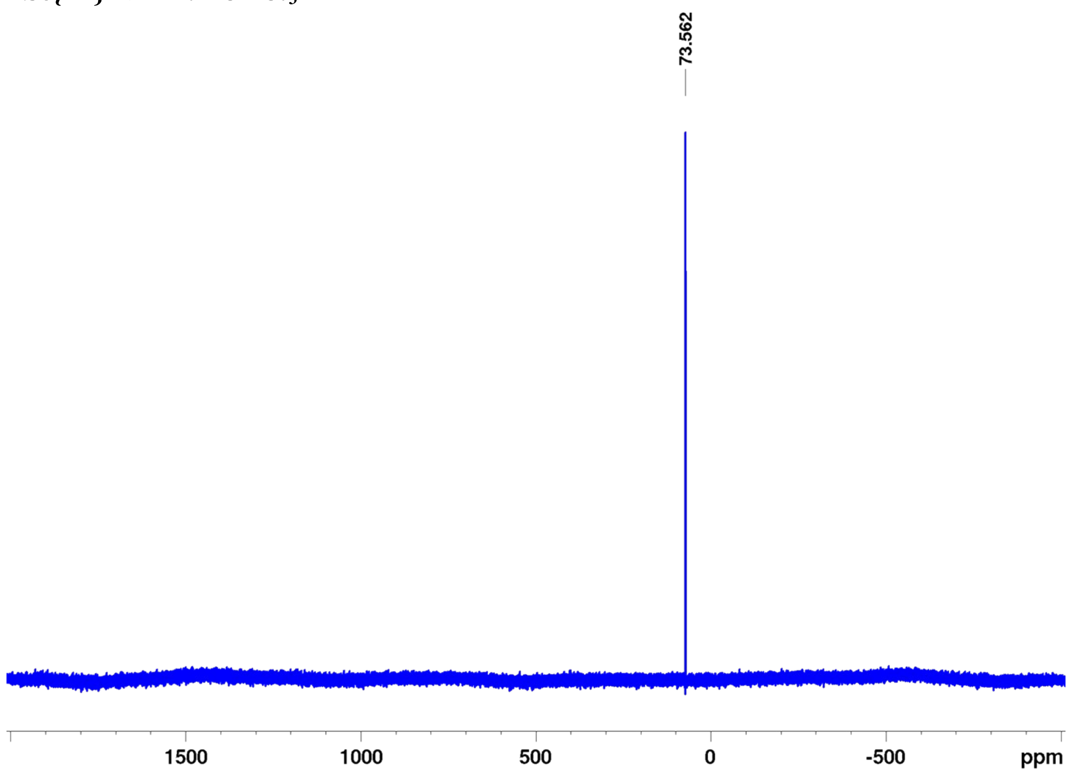

$^{77}Se\{^1H\}$ NMR in acetone-d_6

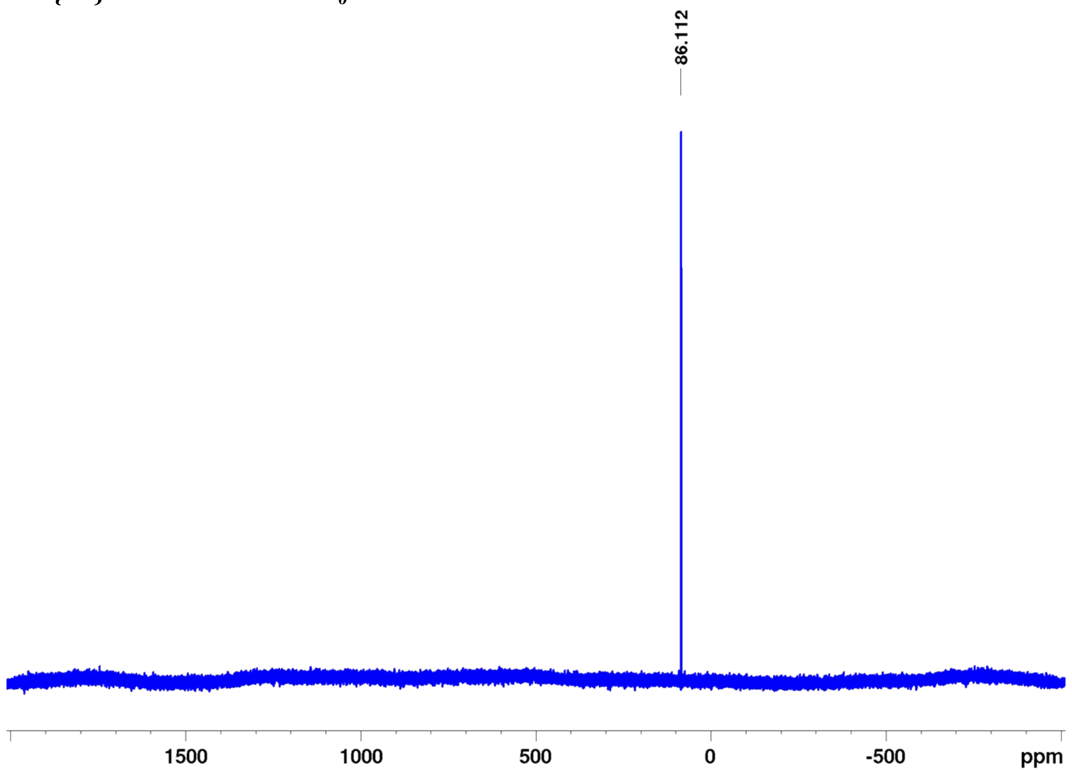

[Se(Tr4)]

^1H NMR in $CDCl_3$

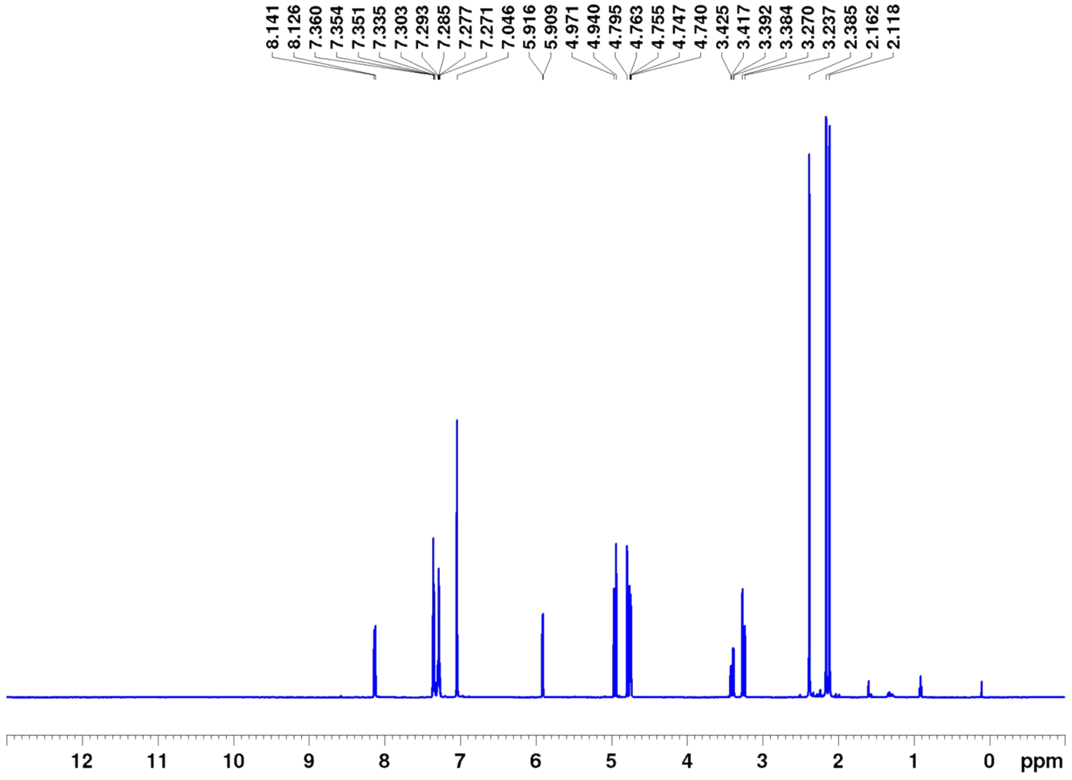

$^{13}C\{^{1}H\}$ NMR in $CDCl_3$

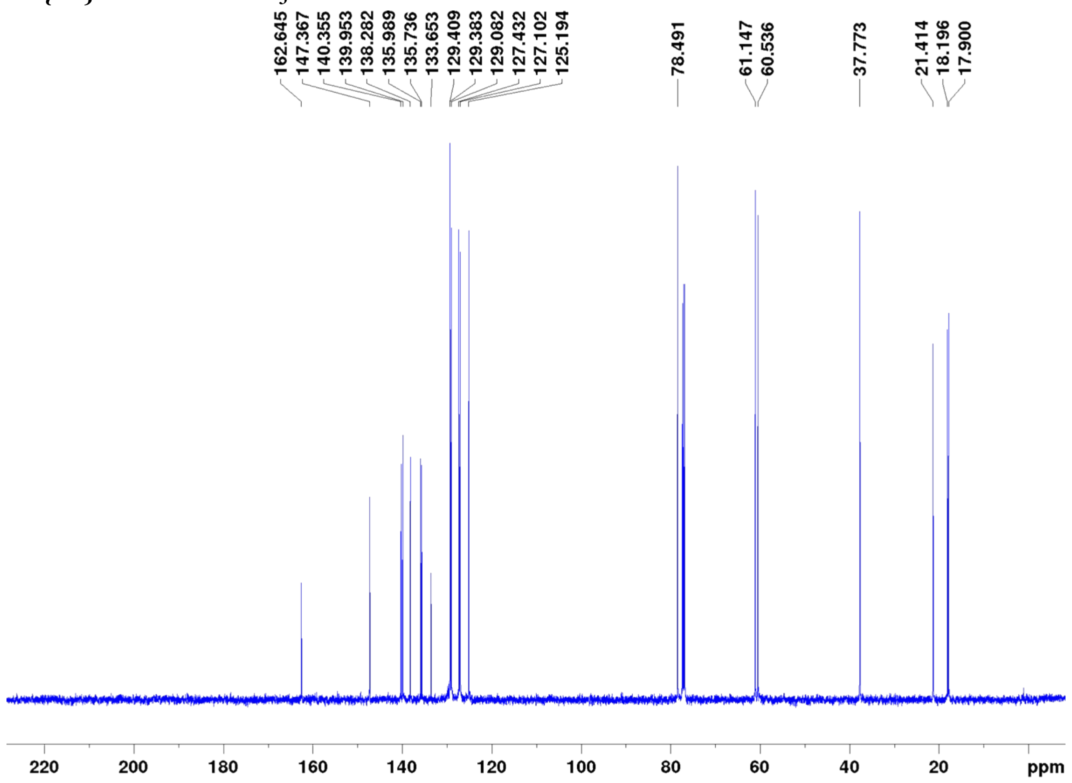

$^{77}Se\{^{1}H\}$ NMR in $CDCl_3$

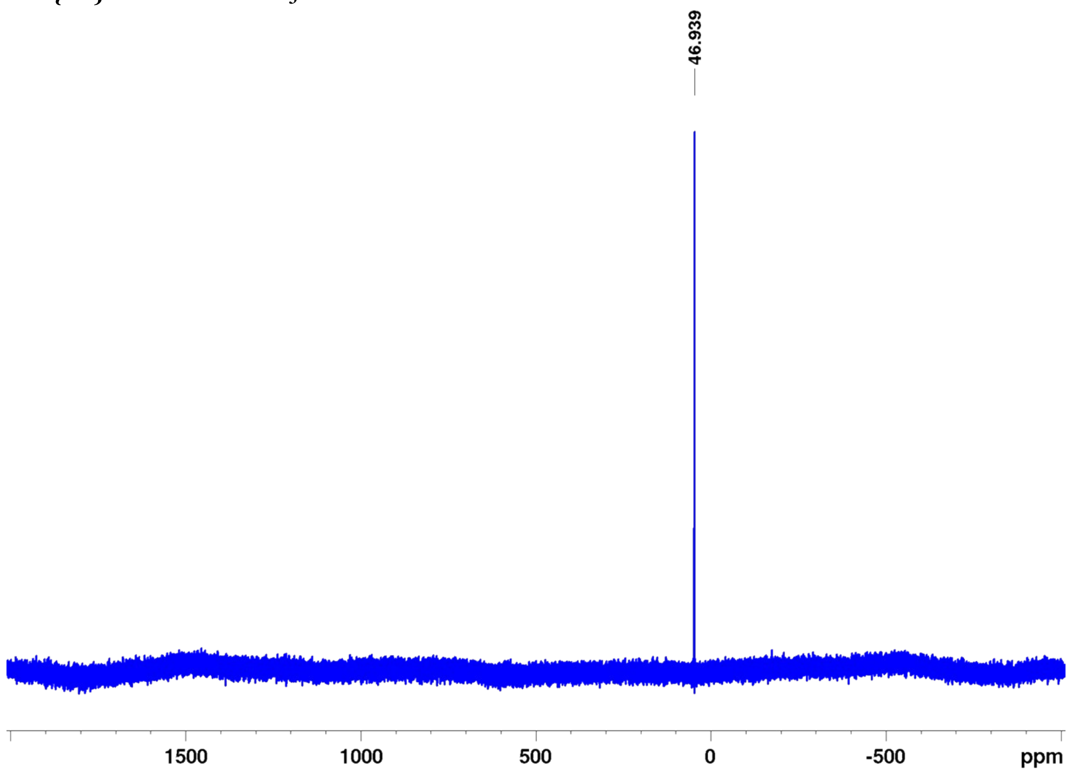

^{77}Se{^{1}H} NMR in acetone-d$_6$

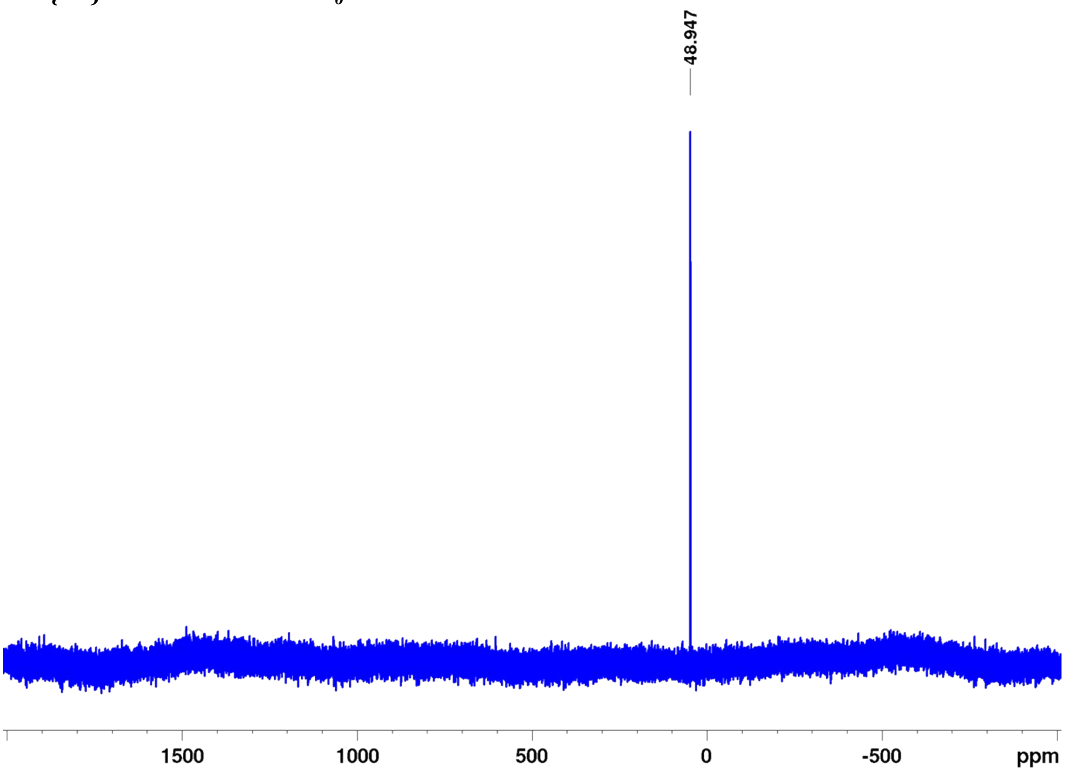

[Se(Tr5)]

^{1}H NMR in CDCl$_3$

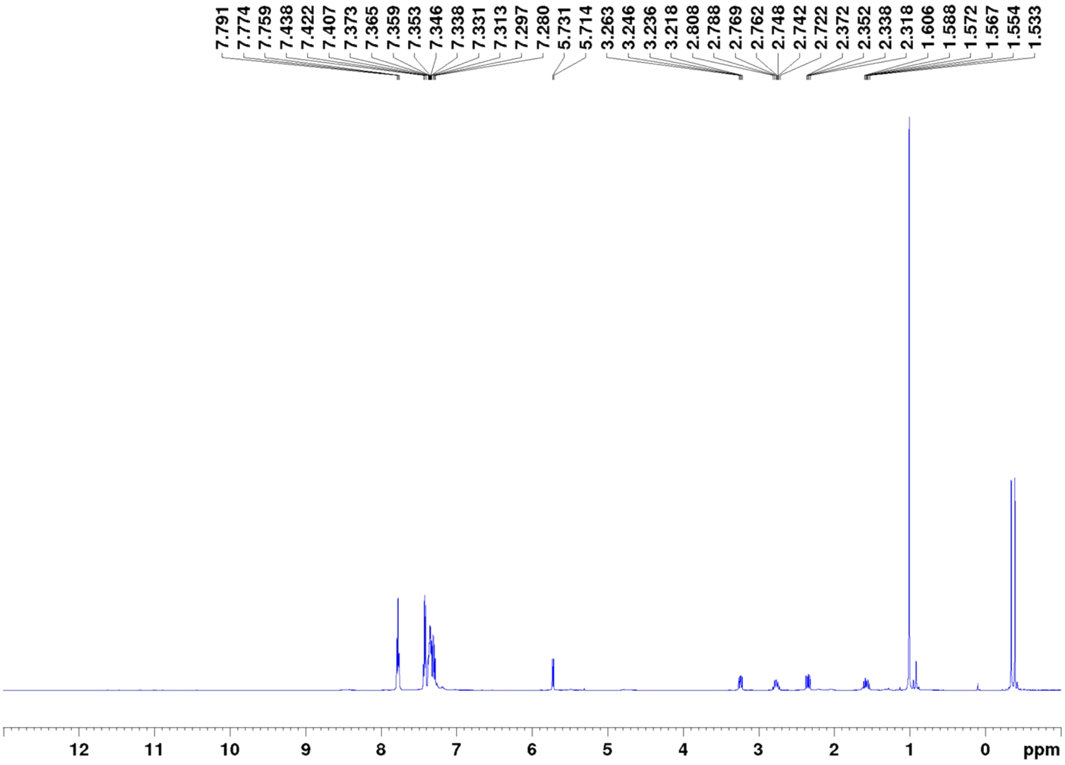

$^{13}C\{^{1}H\}$ NMR in $CDCl_3$

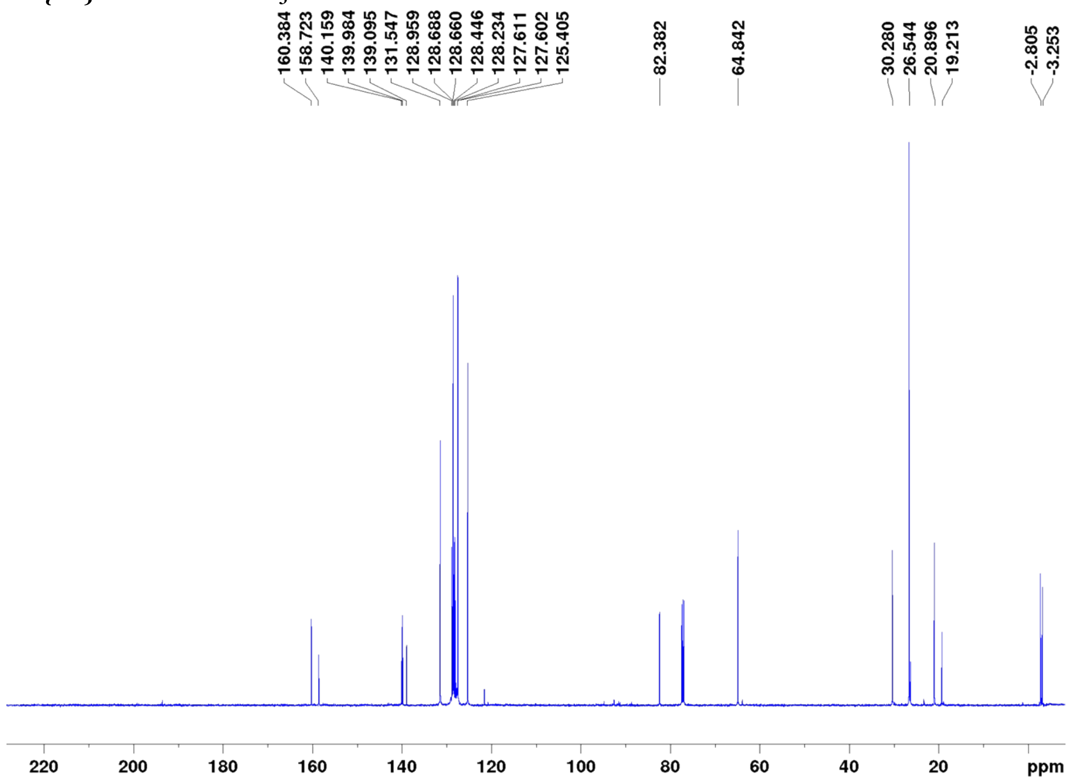

$^{77}Se\{^{1}H\}$ NMR in $CDCl_3$

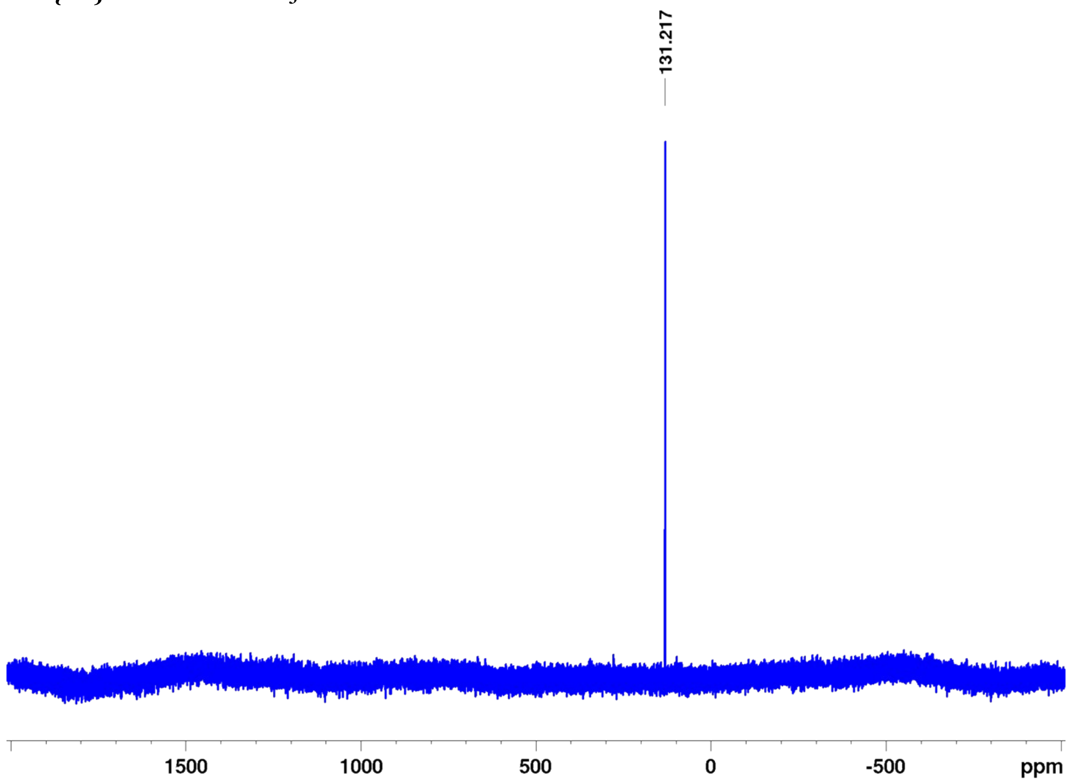

X-ray Crystal Structure Data for Selenourea Compounds

[Se(IAd)]

Experimental

Data Collection

A colorless prism crystal of $C_{23}H_{32}N_2Se$ having approximate dimensions of 0.120 x 0.050 x 0.050 mm was mounted in a loop. All measurements were made on a Rigaku XtaLAB P200 diffractometer using multi-layer mirror monochromated Mo-Kα radiation.

Cell constants and an orientation matrix for data collection corresponded to a primitive orthorhombic cell with dimensions:

a = 23.300(2) Å

b = 7.0807(7) Å

c = 11.6415(13) Å

V = 1920.6(3) Å^3

For Z = 4 and F.W. = 415.48, the calculated density is 1.437 g/cm^3. Based on the reflection conditions of:

0kl: k+l = 2n

h0l: h = 2n

packing considerations, a statistical analysis of intensity distribution, and the successful solution and refinement of the structure, the space group was determined to be:

$Pna2_1$ (#33)

The data were collected at a temperature of -100 $\pm$ 1°C to a maximum 2θ value of 50.8^{o}. Readout was performed in the 0.172 mm pixel mode.

Data Reduction

Of the 22163 reflections were collected, where 3522 were unique (R_{int} = 0.0402); equivalent reflections were merged. Data were collected and processed using CrystalClear (Rigaku). [1]

The linear absorption coefficient, μ, for Mo-Kα radiation is 19.651 cm^{-1}. An empirical absorption correction was applied which resulted in transmission factors ranging from 0.746 to 0.906. The data were corrected for Lorentz and polarization effects.

Structure Solution and Refinement

The structure was solved by heavy-atom Patterson methods[2] and expanded using Fourier techniques. The non-hydrogen atoms were refined anisotropically. Hydrogen atoms were refined using the riding model. The final cycle of full-matrix least-squares refinement[3] on F^2 was based on 3522 observed reflections and 235 variable parameters and converged (largest parameter shift was 0.00 times its esd) with unweighted and weighted agreement factors of:

$$R1 = \Sigma \, ||Fo| - |Fc|| \, / \, \Sigma \, |Fo| = 0.0238$$

$$wR2 = [\, \Sigma \, (\, w \, (Fo^2 - Fc^2)^2 \,) / \, \Sigma \, w(Fo^2)^2]^{1/2} = 0.0568$$

The goodness of fit[4] was 1.01. Unit weights were used. Plots of Σ w $(|Fo| - |Fc|)^2$ versus |Fo|, reflection order in data collection, sin θ/λ and various classes of indices showed no unusual trends. The maximum and minimum peaks on the final difference Fourier map corresponded to 1.05 and -0.15 $e^-/Å^3$, respectively. The final Flack parameter [5] was 0.007(4), indicating that the present absolute structure is correct. [6]

Neutral atom scattering factors were taken from International Tables for Crystallography (IT), Vol. C, Table 6.1.1.4 [7]. Anomalous dispersion effects were included in Fcalc[8]; the values for $\Delta f'$ and $\Delta f''$ were those of Creagh and McAuley[9]. The values for the mass attenuation coefficients are those of Creagh and Hubbell[10]. All calculations were performed using the CrystalStructure[11] crystallographic software package except for refinement, which was performed using SHELXL2013[12].

References

(1) CrystalClear: Data Collection and Processing Software, Rigaku Corporation (1998-2014). Tokyo 196-8666, Japan.

(2) PATTY: Beurskens, P.T., Admiraal, G., Behm, H., Beurskens, G., Smits, J.M.M. and Smykalla, C. (1991). Z. f. Kristallogr. Suppl.4, p.99.

(3) Least Squares function minimized: (SHELXL2013)

$\Sigma w(F_o^2 - F_c^2)^2$ where w = Least Squares weights.

(4) Goodness of fit is defined as:

$[\Sigma w(F_o^2 - F_c^2)^2/(N_o - N_v)]^{1/2}$

where: N_O = number of observations

N_V = number of variables

(5) Parsons, S. and Flack, H. (2004), Acta Cryst. A60, s61.

(6) Flack, H.D. and Bernardinelli (2000), J. Appl. Cryst. 33, 114-1148.

(7) International Tables for Crystallography, Vol.C (1992). Ed. A.J.C. Wilson, Kluwer Academic Publishers, Dordrecht, Netherlands, Table 6.1.1.4, pp. 572.

(8) Ibers, J. A. & Hamilton, W. C.; Acta Crystallogr., 17, 781 (1964).

(9) Creagh, D. C. & McAuley, W.J .; "International Tables for Crystallography", Vol C, (A.J.C. Wilson, ed.), Kluwer Academic Publishers, Boston, Table 4.2.6.8, pages 219-222 (1992).

(10) Creagh, D. C. & Hubbell, J.H..; "International Tables for Crystallography", Vol C, (A.J.C. Wilson, ed.), Kluwer Academic Publishers, Boston, Table 4.2.4.3, pages 200-206 (1992).

(11) CrystalStructure 4.1: Crystal Structure Analysis Package, Rigaku Corporation (2000-2014). Tokyo 196-8666, Japan.

(12) SHELXL2013: Sheldrick, G. M. (2008). Acta Cryst. A64, 112-122.

EXPERIMENTAL DETAILS

A. Crystal Data

Empirical Formula	$C_{23}H_{32}N_2Se$
Formula Weight	415.48
Crystal Color, Habit	colorless, prism
Crystal Dimensions	0.120 X 0.050 X 0.050 mm
Crystal System	orthorhombic
Lattice Type	Primitive
Lattice Parameters	a = 23.300(2) Å
	b = 7.0807(7) Å
	c = 11.6415(13) Å
	V = 1920.6(3) Å^3

Space Group	$Pna2_1$ (#33)
Z value	4
D_{calc}	1.437 g/cm^3
F_{000}	872.00
μ(MoKα)	19.651 cm^{-1}

B. Intensity Measurements

Diffractometer	XtaLAB P200
Radiation	MoKα (λ = 0.71075 Å)
	multi-layer mirror monochromated
Voltage, Current	45kV, 66mA
Temperature	-100.0^{o}C
Detector Aperture	83.8 x 70.0 mm
Pixel Size	0.172 mm
$2\theta_{max}$	50.8^{o}
No. of Reflections Measured	Total: 22163
	Unique: 3522 (R_{int} = 0.0402)
	Parsons quotients (Flack x parameter): 1490
Corrections	Lorentz-polarization
	Absorption
	(trans. factors: 0.746 - 0.906)

C. Structure Solution and Refinement

Structure Solution	Patterson Methods (DIRDIF99 PATTY)

Refinement	Full-matrix least-squares on F^2
Function Minimized	Σ w $(Fo^2 - Fc^2)^2$
Least Squares Weights	w = 1/ [$\sigma^2(Fo^2) + (0.0357 \cdot P)^2$
	+ 0.0000 · P]
	where P = (Max(Fo^2,0) + $2Fc^2$)/3
$2\theta_{max}$ cutoff	50.8^o
Anomalous Dispersion	All non-hydrogen atoms
No. Observations (All reflections)	3522
No. Variables	235
Reflection/Parameter Ratio	14.99
Residuals: R1 (I>2.00σ(I))	0.0238
Residuals: R (All reflections)	0.0257
Residuals: wR2 (All reflections)	0.0568
Goodness of Fit Indicator	1.015
Flack parameter (Parsons' quotients = 1490)	0.007(4)
Max Shift/Error in Final Cycle	0.000
Maximum peak in Final Diff. Map	1.05 $e^-/Å^3$
Minimum peak in Final Diff. Map	-0.15 $e^-/Å^3$

[Se(ICy)]

Experimental

Data Collection

A colorless prism crystal of $C_{15}H_{24}N_2Se$ having approximate dimensions of 0.150 x 0.120 x 0.040 mm was mounted in a loop. All measurements were made on a Rigaku XtaLAB P200 diffractometer using multi-layer mirror monochromated Mo-Kα radiation.

Cell constants and an orientation matrix for data collection corresponded to a primitive monoclinic cell with dimensions:

a = 11.8147(15) Å

b = 11.1443(13) Å β = 108.210(3)^{o}

c = 12.2088(14) Å

V = 1527.0(3) Å^{3}

For Z = 4 and F.W. = 311.33, the calculated density is 1.354 g/cm^{3}. The reflection conditions of:

h0l: l = 2n

0k0: k = 2n

uniquely determine the space group to be:

$P2_1/c$ (#14)

The data were collected at a temperature of -100 $\pm$ 1°C to a maximum 2θ value of 50.8^{o}. Readout was performed in the 0.172 mm pixel mode.

Data Reduction

Of the 18233 reflections were collected, where 2808 were unique (R_{int} = 0.0483); equivalent reflections were merged. Data were collected and processed using CrystalClear (Rigaku). [1]

The linear absorption coefficient, μ, for Mo-Kα radiation is 24.463 cm^{-1}. An empirical absorption correction was applied which resulted in transmission factors ranging from 0.766 to 0.907. The data were corrected for Lorentz and polarization effects.

Structure Solution and Refinement

The structure was solved by heavy-atom Patterson methods[2] and expanded using Fourier techniques. The non-hydrogen atoms were refined anisotropically. Hydrogen atoms were refined using the riding model. The final cycle of full-matrix least-squares refinement[3] on F^2 was based on 2808 observed reflections and 163 variable parameters and converged (largest parameter shift was 0.00 times its esd) with unweighted and weighted agreement factors of:

$$R1 = \Sigma \,||Fo| - |Fc|| \,/\, \Sigma \,|Fo| = 0.0330$$

$$wR2 = [\, \Sigma \,(w\,(Fo^2 - Fc^2)^2)/ \Sigma \, w(Fo^2)^2]^{1/2} = 0.0998$$

The goodness of fit[4] was 1.05. Unit weights were used. Plots of Σ w ($|Fo| - |Fc|$)^2 versus $|Fo|$, reflection order in data collection, sin θ/λ and various classes of indices showed no unusual trends. The maximum and minimum peaks on the final difference Fourier map corresponded to 0.89 and -0.42 $e^-/Å^3$, respectively.

Neutral atom scattering factors were taken from International Tables for Crystallography (IT), Vol. C, Table 6.1.1.4 [5]. Anomalous dispersion effects were included in Fcalc[6]; the values for $\Delta f'$ and $\Delta f''$ were those of Creagh and McAuley[7]. The values for the mass attenuation coefficients are those of Creagh and Hubbell[8]. All calculations were performed using the CrystalStructure[9] crystallographic software package except for refinement, which was performed using SHELXL2013[10].

References

(1) CrystalClear: Data Collection and Processing Software, Rigaku Corporation (1998-2014). Tokyo 196-8666, Japan.

(2) PATTY: Beurskens, P.T., Admiraal, G., Behm, H., Beurskens, G., Smits, J.M.M. and Smykalla, C. (1991). Z. f. Kristallogr. Suppl.4, p.99.

(3) Least Squares function minimized: (SHELXL2013)

$\Sigma w(F_o^2-F_c^2)^2$ where w = Least Squares weights.

(4) Goodness of fit is defined as:

$[\Sigma w(F_o^2-F_c^2)^2/(N_o-N_v)]^{1/2}$

where: N_o = number of observations

N_v = number of variables

(5) International Tables for Crystallography, Vol.C (1992). Ed. A.J.C. Wilson, Kluwer Academic Publishers, Dordrecht, Netherlands, Table 6.1.1.4, pp. 572.

(6) Ibers, J. A. & Hamilton, W. C.; Acta Crystallogr., 17, 781 (1964).

(7) Creagh, D. C. & McAuley, W.J .; "International Tables for Crystallography", Vol C, (A.J.C. Wilson, ed.), Kluwer Academic Publishers, Boston, Table 4.2.6.8, pages 219-222 (1992).

(8) Creagh, D. C. & Hubbell, J.H..; "International Tables for Crystallography", Vol C, (A.J.C. Wilson, ed.), Kluwer Academic Publishers, Boston, Table 4.2.4.3, pages 200-206 (1992).

(9) CrystalStructure 4.1: Crystal Structure Analysis Package, Rigaku Corporation (2000-2014). Tokyo 196-8666, Japan.

(10) SHELXL2013: Sheldrick, G. M. (2008). Acta Cryst. A64, 112-122.

EXPERIMENTAL DETAILS

A. Crystal Data

Empirical Formula	$C_{15}H_{24}N_2Se$
Formula Weight	311.33
Crystal Color, Habit	colorless, prism
Crystal Dimensions	0.150 X 0.120 X 0.040 mm
Crystal System	monoclinic
Lattice Type	Primitive
Lattice Parameters	a = 11.8147(15) Å
	b = 11.1443(13) Å
	c = 12.2088(14) Å
	β = 108.210(3) ^{o}
	V = 1527.0(3) Å^3
Space Group	$P2_1/c$ (#14)
Z value	4
D_{calc}	1.354 g/cm^3
F_{000}	648.00
μ(MoKα)	24.463 cm^{-1}

B. Intensity Measurements

Diffractometer	XtaLAB P200
Radiation	MoKα (λ = 0.71075 Å)
	multi-layer mirror monochromated
Voltage, Current	45kV, 66mA
Temperature	-100.0^{o}C
Detector Aperture	83.8 x 70.0 mm
Pixel Size	0.172 mm
$2\theta_{max}$	50.8^{o}
No. of Reflections Measured	Total: 18233
	Unique: 2808 (R_{int} = 0.0483)
Corrections	Lorentz-polarization
	Absorption
	(trans. factors: 0.766 - 0.907)

C. Structure Solution and Refinement

Structure Solution	Patterson Methods (DIRDIF99 PATTY)
Refinement	Full-matrix least-squares on F^2
Function Minimized	Σ w $(Fo^2 - Fc^2)^2$
Least Squares Weights	w = 1/ [$\sigma^2(Fo^2) + (0.0730 \cdot P)^2$
	+ 0.0000 · P]
	where P = (Max(Fo^2,0) + 2Fc^2)/3
$2\theta_{max}$ cutoff	50.8^{o}
Anomalous Dispersion	All non-hydrogen atoms
No. Observations (All reflections)	2808
No. Variables	163

Reflection/Parameter Ratio	17.23
Residuals: R1 (I>2.00σ(I))	0.0330
Residuals: R (All reflections)	0.0393
Residuals: wR2 (All reflections)	0.0998
Goodness of Fit Indicator	1.047
Max Shift/Error in Final Cycle	0.000
Maximum peak in Final Diff. Map	0.89 $e^-/Å^3$
Minimum peak in Final Diff. Map	-0.42 $e^-/Å^3$

[Se(IDD)]

Experimental

Data Collection

A colorless prism crystal of $C_{27}H_{48}N_2Se$ having approximate dimensions of 0.210 x 0.060 x 0.030 mm was mounted in a loop. All measurements were made on a Rigaku XtaLAB P200 diffractometer using multi-layer mirror monochromated Mo-Kα radiation.

Cell constants and an orientation matrix for data collection corresponded to a primitive monoclinic cell with dimensions:

a = 14.4058(13) Å

b = 7.4133(7) Å β = 97.856(3)^o

c = 25.023(3) Å

V = 2647.2(4) $Å^3$

For Z = 4 and F.W. = 479.65, the calculated density is 1.203 g/cm^3. The reflection conditions of:

h0l: h+l = 2n

0k0: k = 2n

uniquely determine the space group to be:

$P2_1/n$ (#14)

The data were collected at a temperature of -100 $\pm$ 1°C to a maximum 2θ value of 50.8°. Readout was performed in the 0.172 mm pixel mode.

Data Reduction

Of the 31247 reflections were collected, where 4854 were unique (R_{int} = 0.0379); equivalent reflections were merged. Data were collected and processed using CrystalClear (Rigaku). [1]

The linear absorption coefficient, μ, for Mo-Kα radiation is 14.341 cm^{-1}. An empirical absorption correction was applied which resulted in transmission factors ranging from 0.877 to 0.958. The data were corrected for Lorentz and polarization effects.

Structure Solution and Refinement

The structure was solved by direct methods[2] and expanded using Fourier techniques. The non-hydrogen atoms were refined anisotropically. Hydrogen atoms were refined using the riding model. The final cycle of full-matrix least-squares refinement[3] on F^2 was based on 4854 observed reflections and 271 variable parameters and converged (largest parameter shift was 0.00 times its esd) with unweighted and weighted agreement factors of:

$$R1 = \Sigma ||Fo| - |Fc|| / \Sigma |Fo| = 0.0704$$

$$wR2 = [\Sigma (w (Fo^2 - Fc^2)^2)/ \Sigma w(Fo^2)^2]^{1/2} = 0.2216$$

The goodness of fit[4] was 1.07. Unit weights were used. Plots of Σ w $(|Fo| - |Fc|)^2$ versus |Fo|, reflection order in data collection, sin θ/λ and various classes of indices showed no unusual trends. The maximum and minimum peaks on the final difference Fourier map corresponded to 1.71 and -0.54 $e^-/Å^3$, respectively.

Neutral atom scattering factors were taken from International Tables for Crystallography (IT), Vol. C, Table 6.1.1.4 [5]. Anomalous dispersion effects were included in Fcalc[6]; the values for Δf' and Δf" were those of Creagh and McAuley[7]. The values for the mass attenuation coefficients are those of Creagh and Hubbell[8]. All calculations were performed using the CrystalStructure[9] crystallographic software package except for refinement, which was performed using SHELXL2013[10].

References

(1) CrystalClear: Data Collection and Processing Software, Rigaku Corporation (1998-2014). Tokyo 196-8666, Japan.

(2) SIR2011: Burla, M. C., Caliandro, R., Camalli, M., Carrozzini, B., Cascarano, G. L., Giacovazzo, C., Mallamo, M., Mazzone, A., Polidori, G. and Spagna, R. (2012). J. Appl. Cryst. 45, 357-361.

(3) Least Squares function minimized: (SHELXL2013)

$\Sigma w(F_o^2-F_c^2)^2$ where w = Least Squares weights.

(4) Goodness of fit is defined as:

$[\Sigma w(F_o^2-F_c^2)^2/(N_o-N_v)]^{1/2}$

where: N_o = number of observations

N_v = number of variables

(5) International Tables for Crystallography, Vol.C (1992). Ed. A.J.C. Wilson, Kluwer Academic Publishers, Dordrecht, Netherlands, Table 6.1.1.4, pp. 572.

(6) Ibers, J. A. & Hamilton, W. C.; Acta Crystallogr., 17, 781 (1964).

(7) Creagh, D. C. & McAuley, W.J .; "International Tables for Crystallography", Vol C, (A.J.C. Wilson, ed.), Kluwer Academic Publishers, Boston, Table 4.2.6.8, pages 219-222 (1992).

(8) Creagh, D. C. & Hubbell, J.H..; "International Tables for Crystallography", Vol C, (A.J.C. Wilson, ed.), Kluwer Academic Publishers, Boston, Table 4.2.4.3, pages 200-206 (1992).

(9) CrystalStructure 4.1: Crystal Structure Analysis Package, Rigaku Corporation (2000-2014). Tokyo 196-8666, Japan.

(10) SHELXL2013: Sheldrick, G. M. (2008). Acta Cryst. A64, 112-122.

EXPERIMENTAL DETAILS

A. Crystal Data

Empirical Formula	$C_{27}H_{48}N_2Se$
Formula Weight	479.65
Crystal Color, Habit	colorless, prism

Crystal Dimensions	0.210 X 0.060 X 0.030 mm
Crystal System	monoclinic
Lattice Type	Primitive
Lattice Parameters	a = 14.4058(13) Å
	b = 7.4133(7) Å
	c = 25.023(3) Å
	β = 97.856(3) ^{o}
	V = 2647.2(4) Å^{3}
Space Group	$P2_1/n$ (#14)
Z value	4
D_{calc}	1.203 g/cm^{3}
F_{000}	1032.00
μ(MoKα)	14.341 cm^{-1}

B. Intensity Measurements

Diffractometer	XtaLAB P200
Radiation	MoKα (λ = 0.71075 Å)
	multi-layer mirror monochromated
Voltage, Current	45kV, 66mA
Temperature	-100.0^{o}C
Detector Aperture	83.8 x 70.0 mm
Pixel Size	0.172 mm
$2\theta_{max}$	50.8^{o}
No. of Reflections Measured	Total: 31247
	Unique: 4854 (R_{int} = 0.0379)

Corrections	Lorentz-polarization
	Absorption
	(trans. factors: 0.877 - 0.958)

C. Structure Solution and Refinement

Structure Solution	Direct Methods (SIR2011)
Refinement	Full-matrix least-squares on F^2
Function Minimized	Σ w $(Fo^2 - Fc^2)^2$
Least Squares Weights	w = 1/ [$\sigma^2(Fo^2)$ + $(0.1301 \cdot P)^2$
	+ 4.9833 · P]
	where P = (Max(Fo^2,0) + 2Fc^2)/3
$2\theta_{max}$ cutoff	50.8^o
Anomalous Dispersion	All non-hydrogen atoms
No. Observations (All reflections)	4854
No. Variables	271
Reflection/Parameter Ratio	17.91
Residuals: R1 (I>2.00σ(I))	0.0704
Residuals: R (All reflections)	0.0841
Residuals: wR2 (All reflections)	0.2216
Goodness of Fit Indicator	1.066
Max Shift/Error in Final Cycle	0.001
Maximum peak in Final Diff. Map	1.71 $e^-/Å^3$
Minimum peak in Final Diff. Map	-0.54 $e^-/Å^3$

[Se(IMe)]

Experimental

Data Collection

A colorless platelet crystal of $C_{19}H_{20}N_2Se$ having approximate dimensions of 0.180 x 0.150 x 0.020 mm was mounted in a loop. All measurements were made on a Rigaku XtaLAB P200 diffractometer using multi-layer mirror monochromated Mo-Kα radiation.

Cell constants and an orientation matrix for data collection corresponded to a primitive monoclinic cell with dimensions:

a = 8.3318(11) Å

b = 7.4265(9) Å β = 98.798(3)^{o}

c = 13.6459(19) Å

V = 834.42(19) Å^3

For Z = 2 and F.W. = 355.34, the calculated density is 1.414 g/cm^3. Based on the reflection conditions of:

h0l: h+l = 2n

packing considerations, a statistical analysis of intensity distribution, and the successful solution and refinement of the structure, the space group was determined to be:

P2/n (#13)

The data were collected at a temperature of -100 $\pm$ 1°C to a maximum 2θ value of 50.7^{o}. Readout was performed in the 0.172 mm pixel mode.

Data Reduction

Of the 9772 reflections were collected, where 1532 were unique (R_{int} = 0.0564); equivalent reflections were merged. Data were collected and processed using CrystalClear (Rigaku). [1]

The linear absorption coefficient, μ, for Mo-Kα radiation is 22.487 cm^{-1}. An empirical absorption correction was applied which resulted in transmission factors ranging from 0.655 to 0.956. The data were corrected for Lorentz and polarization effects.

Structure Solution and Refinement

The structure was solved by heavy-atom Patterson methods[2] and expanded using Fourier techniques. The non-hydrogen atoms were refined anisotropically. Hydrogen atoms were refined using the riding model. The final cycle of full-matrix least-squares refinement[3] on F^2 was based on 1532 observed reflections and 103 variable parameters and converged (largest parameter shift was 0.00 times its esd) with unweighted and weighted agreement factors of:

$$R1 = \Sigma \,||Fo| - |Fc|| \,/\, \Sigma \,|Fo| = 0.0277$$

$$wR2 = [\, \Sigma \,(\, w\,(Fo^2 - Fc^2)^2\,)/\, \Sigma\, w(Fo^2)^2]^{1/2} = 0.0736$$

The goodness of fit[4] was 1.02. Unit weights were used. Plots of $\Sigma\, w\,(|Fo| - |Fc|)^2$ versus $|Fo|$, reflection order in data collection, sin θ/λ and various classes of indices showed no unusual trends. The maximum and minimum peaks on the final difference Fourier map corresponded to 0.54 and -0.26 $e^-/Å^3$, respectively.

Neutral atom scattering factors were taken from International Tables for Crystallography (IT), Vol. C, Table 6.1.1.4 [5]. Anomalous dispersion effects were included in Fcalc[6]; the values for $\Delta f'$ and $\Delta f''$ were those of Creagh and McAuley[7]. The values for the mass attenuation coefficients are those of Creagh and Hubbell[8]. All calculations were performed using the CrystalStructure[9] crystallographic software package except for refinement, which was performed using SHELXL2013[10].

References

(1) CrystalClear: Data Collection and Processing Software, Rigaku Corporation (1998-2014). Tokyo 196-8666, Japan.

(2) PATTY: Beurskens, P.T., Admiraal, G., Behm, H., Beurskens, G., Smits, J.M.M. and Smykalla, C. (1991). Z. f. Kristallogr. Suppl.4, p.99.

(3) Least Squares function minimized: (SHELXL2013)

$\Sigma w(F_o^2 - F_c^2)^2$ where w = Least Squares weights.

(4) Goodness of fit is defined as:

$[\Sigma w(F_o^2 - F_c^2)^2/(N_o - N_v)]^{1/2}$

where: N_o = number of observations

N_V = number of variables

(5) International Tables for Crystallography, Vol.C (1992). Ed. A.J.C. Wilson, Kluwer Academic Publishers, Dordrecht, Netherlands, Table 6.1.1.4, pp. 572.

(6) Ibers, J. A. & Hamilton, W. C.; Acta Crystallogr., 17, 781 (1964).

(7) Creagh, D. C. & McAuley, W.J .; "International Tables for Crystallography", Vol C, (A.J.C. Wilson, ed.), Kluwer Academic Publishers, Boston, Table 4.2.6.8, pages 219-222 (1992).

(8) Creagh, D. C. & Hubbell, J.H..; "International Tables for Crystallography", Vol C, (A.J.C. Wilson, ed.), Kluwer Academic Publishers, Boston, Table 4.2.4.3, pages 200-206 (1992).

(9) CrystalStructure 4.1: Crystal Structure Analysis Package, Rigaku Corporation (2000-2014). Tokyo 196-8666, Japan.

(10) SHELXL2013: Sheldrick, G. M. (2008). Acta Cryst. A64, 112-122.

EXPERIMENTAL DETAILS

A. Crystal Data

Empirical Formula	$C_{19}H_{20}N_2Se$
Formula Weight	355.34
Crystal Color, Habit	colorless, platelet
Crystal Dimensions	0.180 X 0.150 X 0.020 mm
Crystal System	monoclinic
Lattice Type	Primitive
Lattice Parameters	a = 8.3318(11) Å
	b = 7.4265(9) Å
	c = 13.6459(19) Å
	β = 98.798(3) ^{o}
	V = 834.42(19) $Å^3$
Space Group	P2/n (#13)
Z value	2

D_{calc}	1.414 g/cm^3
F_{000}	364.00
μ(MoKα)	22.487 cm^{-1}

B. Intensity Measurements

Diffractometer	XtaLAB P200
Radiation	MoKα (λ = 0.71075 Å)
	multi-layer mirror monochromated
Voltage, Current	45kV, 66mA
Temperature	-100.0^{o}C
Detector Aperture	83.8 x 70.0 mm
Pixel Size	0.172 mm
$2\theta_{max}$	50.7^{o}
No. of Reflections Measured	Total: 9772
	Unique: 1532 (R_{int} = 0.0564)
Corrections	Lorentz-polarization
	Absorption
	(trans. factors: 0.655 - 0.956)

C. Structure Solution and Refinement

Structure Solution	Patterson Methods (DIRDIF99 PATTY)
Refinement	Full-matrix least-squares on F^2
Function Minimized	Σ w (Fo^2 - Fc^2)^2
Least Squares Weights	w = 1/ [$\sigma^2(Fo^2)$ + (0.0489 · P)^2
	+ 0.0000 · P]

	where P = (Max(Fo^2,0) + 2Fc^2)/3
$2\theta_{max}$ cutoff	50.7°
Anomalous Dispersion	All non-hydrogen atoms
No. Observations (All reflections)	1532
No. Variables	103
Reflection/Parameter Ratio	14.87
Residuals: R1 (I>2.00σ(I))	0.0277
Residuals: R (All reflections)	0.0308
Residuals: wR2 (All reflections)	0.0736
Goodness of Fit Indicator	1.018
Max Shift/Error in Final Cycle	0.001
Maximum peak in Final Diff. Map	0.54 $e^-/Å^3$
Minimum peak in Final Diff. Map	-0.26 $e^-/Å^3$

[Se(IMes)]

Experimental

Data Collection

A yellow prism crystal of $C_{21}H_{24}N_2Se$ having approximate dimensions of 0.150 x 0.060 x 0.060 mm was mounted in a loop. All measurements were made on a Rigaku XtaLAB P200 diffractometer using multi-layer mirror monochromated Mo-Kα radiation.

Cell constants and an orientation matrix for data collection corresponded to a primitive orthorhombic cell with dimensions:

a = 16.043(2) Å

b = 7.3937(9) Å

c = 16.055(2) Å

V = 1904.3(4) $Å^3$

For Z = 4 and F.W. = 383.39, the calculated density is 1.337 g/cm^3. The reflection conditions of:

0kl: k = 2n

h0l: l = 2n

hk0: h+k = 2n

uniquely determine the space group to be:

Pbcn (#60)

The data were collected at a temperature of -100 $\pm$ 1°C to a maximum 2θ value of 50.8^{o}. Readout was performed in the 0.172 mm pixel mode.

Data Reduction

Of the 21284 reflections were collected, where 1745 were unique (R_{int} = 0.0597); equivalent reflections were merged. Data were collected and processed using CrystalClear (Rigaku). [1]

The linear absorption coefficient, μ, for Mo-Kα radiation is 19.760 cm^{-1}. An empirical absorption correction was applied which resulted in transmission factors ranging from 0.749 to 0.888. The data were corrected for Lorentz and polarization effects.

Structure Solution and Refinement

The structure was solved by direct methods[2] and expanded using Fourier techniques. The non-hydrogen atoms were refined anisotropically. Hydrogen atoms were refined using the riding model. The final cycle of full-matrix least-squares refinement[3] on F^2 was based on 1745 observed reflections and 113 variable parameters and converged (largest parameter shift was 0.00 times its esd) with unweighted and weighted agreement factors of:

$$R1 = \Sigma \,||Fo| - |Fc|| \,/\, \Sigma \,|Fo| = 0.0686$$

$$wR2 = [\,\Sigma\,(\,w\,(Fo^2 - Fc^2)^2\,)/\,\Sigma\,w(Fo^2)^2]^{1/2} = 0.1917$$

The goodness of fit[4] was 1.11. Unit weights were used. Plots of Σ w (|Fo| - |Fc|)^2 versus |Fo|, reflection order in data collection, sin θ/λ and various classes of indices showed no unusual trends. The maximum and minimum peaks on the final difference Fourier map corresponded to 2.44 and -0.94 e$^-$/Å^3, respectively.

Neutral atom scattering factors were taken from International Tables for Crystallography (IT), Vol. C, Table 6.1.1.4 [5]. Anomalous dispersion effects were included in Fcalc[6]; the values for Δf' and Δf" were those of Creagh and McAuley[7]. The values for the mass attenuation coefficients are those of Creagh and Hubbell[8]. All calculations were performed using the CrystalStructure[9] crystallographic software package except for refinement, which was performed using SHELXL2013[10].

References

(1) CrystalClear: Data Collection and Processing Software, Rigaku Corporation (1998-2014). Tokyo 196-8666, Japan.

(2) SHELXS2013: Sheldrick, G. M. (2008). Acta Cryst. A64, 112-122.

(3) Least Squares function minimized: (SHELXL2013)

$\Sigma w(F_o^2-F_c^2)^2$ where w = Least Squares weights.

(4) Goodness of fit is defined as:

$[\Sigma w(F_o^2-F_c^2)^2/(N_o-N_v)]^{1/2}$

where: N_o = number of observations

N_v = number of variables

(5) International Tables for Crystallography, Vol.C (1992). Ed. A.J.C. Wilson, Kluwer Academic Publishers, Dordrecht, Netherlands, Table 6.1.1.4, pp. 572.

(6) Ibers, J. A. & Hamilton, W. C.; Acta Crystallogr., 17, 781 (1964).

(7) Creagh, D. C. & McAuley, W.J .; "International Tables for Crystallography", Vol C, (A.J.C. Wilson, ed.), Kluwer Academic Publishers, Boston, Table 4.2.6.8, pages 219-222 (1992).

(8) Creagh, D. C. & Hubbell, J.H..; "International Tables for Crystallography", Vol C, (A.J.C. Wilson, ed.), Kluwer Academic Publishers, Boston, Table 4.2.4.3, pages 200-206 (1992).

(9) CrystalStructure 4.1: Crystal Structure Analysis Package, Rigaku Corporation (2000-2014). Tokyo 196-8666, Japan.

(10) SHELXL2013: Sheldrick, G. M. (2008). Acta Cryst. A64, 112-122.

EXPERIMENTAL DETAILS

A. Crystal Data

Empirical Formula	$C_{21}H_{24}N_2Se$
Formula Weight	383.39
Crystal Color, Habit	yellow, prism
Crystal Dimensions	0.150 X 0.060 X 0.060 mm
Crystal System	orthorhombic
Lattice Type	Primitive
Lattice Parameters	a = 16.043(2) Å
	b = 7.3937(9) Å
	c = 16.055(2) Å
	V = 1904.3(4) $Å^3$
Space Group	Pbcn (#60)
Z value	4
D_{calc}	1.337 g/cm^3
F_{000}	792.00
μ(MoKα)	19.760 cm^{-1}

B. Intensity Measurements

Diffractometer	XtaLAB P200
Radiation	MoKα (λ = 0.71075 Å)
	multi-layer mirror monochromated
Voltage, Current	45kV, 66mA
Temperature	-100.0^{o}C
Detector Aperture	83.8 x 70.0 mm

Pixel Size	0.172 mm
$2\theta_{max}$	50.8^{o}
No. of Reflections Measured	Total: 21284
	Unique: 1745 (R_{int} = 0.0597)
Corrections	Lorentz-polarization
	Absorption
	(trans. factors: 0.749 - 0.888)

C. Structure Solution and Refinement

Structure Solution	Direct Methods (SHELXS2013)
Refinement	Full-matrix least-squares on F^2
Function Minimized	Σ w $(Fo^2 - Fc^2)^2$
Least Squares Weights	w = 1/ [$\sigma^2(Fo^2)$ + $(0.1188 \cdot P)^2$
	+ 2.6816 · P]
	where P = (Max(Fo^2,0) + 2Fc^2)/3
$2\theta_{max}$ cutoff	50.8^{o}
Anomalous Dispersion	All non-hydrogen atoms
No. Observations (All reflections)	1745
No. Variables	113
Reflection/Parameter Ratio	15.44
Residuals: R1 (I>2.00σ(I))	0.0686
Residuals: R (All reflections)	0.0791
Residuals: wR2 (All reflections)	0.1917
Goodness of Fit Indicator	1.106
Max Shift/Error in Final Cycle	0.000

Maximum peak in Final Diff. Map 2.44 $e^-/Å^3$

Minimum peak in Final Diff. Map -0.94 $e^-/Å^3$

[Se(IPent)]

Experimental

<u>Data Collection</u>

A colorless prism crystal of $C_{35}H_{52}N_2Se$ having approximate dimensions of 0.150 x 0.060 x 0.020 mm was mounted in a loop. All measurements were made on a Rigaku XtaLAB P200 diffractometer using multi-layer mirror monochromated Mo-Kα radiation.

Cell constants and an orientation matrix for data collection corresponded to a primitive monoclinic cell with dimensions:

a = 20.433(2) Å

b = 7.6327(6) Å β = 106.660(3)^o

c = 21.563(2) Å

V = 3221.7(6) $Å^3$

For Z = 4 and F.W. = 579.77, the calculated density is 1.195 g/cm^3. The reflection conditions of:

h0l: l = 2n

0k0: k = 2n

uniquely determine the space group to be:

$P2_1/c$ (#14)

The data were collected at a temperature of -100 $\pm$ 1°C to a maximum 2θ value of 50.8^o. Readout was performed in the 0.172 mm pixel mode.

Data Reduction

Of the 37896 reflections were collected, where 5921 were unique (R_{int} = 0.0499); equivalent reflections were merged. Data were collected and processed using CrystalClear (Rigaku). [1]

The linear absorption coefficient, μ, for Mo-Kα radiation is 11.901 cm^{-1}. An empirical absorption correction was applied which resulted in transmission factors ranging from 0.867 to 0.976. The data were corrected for Lorentz and polarization effects.

Structure Solution and Refinement

The structure was solved by heavy-atom Patterson methods[2] and expanded using Fourier techniques. The non-hydrogen atoms were refined anisotropically. Hydrogen atoms were refined using the riding model. The final cycle of full-matrix least-squares refinement[3] on F^2 was based on 5921 observed reflections and 351 variable parameters and converged (largest parameter shift was 0.00 times its esd) with unweighted and weighted agreement factors of:

$$R1 = \Sigma \, ||Fo| - |Fc|| \, / \, \Sigma \, |Fo| = 0.0448$$

$$wR2 = [\, \Sigma \, (w \, (Fo^2 - Fc^2)^2 \,) / \, \Sigma \, w(Fo^2)^2]^{1/2} = 0.1139$$

The goodness of fit[4] was 1.03. Unit weights were used. Plots of $\Sigma \, w \, (|Fo| - |Fc|)^2$ versus $|Fo|$, reflection order in data collection, sin θ/λ and various classes of indices showed no unusual trends. The maximum and minimum peaks on the final difference Fourier map corresponded to 0.63 and -0.33 $e^-/Å^3$, respectively.

Neutral atom scattering factors were taken from International Tables for Crystallography (IT), Vol. C, Table 6.1.1.4 [5]. Anomalous dispersion effects were included in Fcalc[6]; the values for $\Delta f'$ and $\Delta f''$ were those of Creagh and McAuley[7]. The values for the mass attenuation coefficients are those of Creagh and Hubbell[8]. All calculations were performed using the CrystalStructure[9] crystallographic software package except for refinement, which was performed using SHELXL2013[10].

References

(1) CrystalClear: Data Collection and Processing Software, Rigaku Corporation (1998-2014). Tokyo 196-8666, Japan.

(2) PATTY: Beurskens, P.T., Admiraal, G., Behm, H., Beurskens, G., Smits, J.M.M. and Smykalla, C. (1991). Z. f. Kristallogr. Suppl.4, p.99.

(3) Least Squares function minimized: (SHELXL2013)

$$\Sigma w(F_o^2-F_c^2)^2 \quad \text{where w = Least Squares weights.}$$

(4) Goodness of fit is defined as:

$$[\Sigma w(F_o^2-F_c^2)^2/(N_o-N_v)]^{1/2}$$

where: N_o = number of observations

N_v = number of variables

(5) International Tables for Crystallography, Vol.C (1992). Ed. A.J.C. Wilson, Kluwer Academic Publishers, Dordrecht, Netherlands, Table 6.1.1.4, pp. 572.

(6) Ibers, J. A. & Hamilton, W. C.; Acta Crystallogr., 17, 781 (1964).

(7) Creagh, D. C. & McAuley, W.J .; "International Tables for Crystallography", Vol C, (A.J.C. Wilson, ed.), Kluwer Academic Publishers, Boston, Table 4.2.6.8, pages 219-222 (1992).

(8) Creagh, D. C. & Hubbell, J.H..; "International Tables for Crystallography", Vol C, (A.J.C. Wilson, ed.), Kluwer Academic Publishers, Boston, Table 4.2.4.3, pages 200-206 (1992).

(9) CrystalStructure 4.1: Crystal Structure Analysis Package, Rigaku Corporation (2000-2014). Tokyo 196-8666, Japan.

(10) SHELXL2013: Sheldrick, G. M. (2008). Acta Cryst. A64, 112-122.

EXPERIMENTAL DETAILS

A. Crystal Data

Empirical Formula	$C_{35}H_{52}N_2Se$
Formula Weight	579.77
Crystal Color, Habit	colorless, prism
Crystal Dimensions	0.150 X 0.060 X 0.020 mm
Crystal System	monoclinic
Lattice Type	Primitive

Lattice Parameters	a = 20.433(2) Å
	b = 7.6327(6) Å
	c = 21.563(2) Å
	β = 106.660(3) ^{o}
	V = 3221.7(6) Å^{3}
Space Group	$P2_1/c$ (#14)
Z value	4
D_{calc}	1.195 g/cm^{3}
F_{000}	1240.00
μ(MoKα)	11.901 cm^{-1}

B. Intensity Measurements

Diffractometer	XtaLAB P200
Radiation	MoKα (λ = 0.71075 Å)
	multi-layer mirror monochromated
Voltage, Current	45kV, 66mA
Temperature	-100.0^{o}C
Detector Aperture	83.8 x 70.0 mm
Pixel Size	0.172 mm
$2\theta_{max}$	50.8^{o}
No. of Reflections Measured	Total: 37896
	Unique: 5921 (R_{int} = 0.0499)
Corrections	Lorentz-polarization
	Absorption
	(trans. factors: 0.867 - 0.976)

C. Structure Solution and Refinement

Structure Solution	Patterson Methods (DIRDIF99 PATTY)
Refinement	Full-matrix least-squares on F^2
Function Minimized	Σ w $(Fo^2 - Fc^2)^2$
Least Squares Weights	w = 1/ [$\sigma^2(Fo^2)$ + $(0.0500 \cdot P)^2$
	+ 3.3801 · P]
	where P = $(Max(Fo^2,0) + 2Fc^2)/3$
$2\theta_{max}$ cutoff	50.8^o
Anomalous Dispersion	All non-hydrogen atoms
No. Observations (All reflections)	5921
No. Variables	351
Reflection/Parameter Ratio	16.87
Residuals: R1 (I>2.00σ(I))	0.0448
Residuals: R (All reflections)	0.0608
Residuals: wR2 (All reflections)	0.1139
Goodness of Fit Indicator	1.033
Max Shift/Error in Final Cycle	0.001
Maximum peak in Final Diff. Map	0.63 $e^-/Å^3$
Minimum peak in Final Diff. Map	-0.33 $e^-/Å^3$

[Se(IPr^{Cl})]

Experimental

Data Collection

A colorless prism crystal of $C_{35}H_{52}N_2Se$ having approximate dimensions of 0.150 x 0.060 x 0.020 mm was mounted in a loop. All measurements were made on a Rigaku XtaLAB P200 diffractometer using multi-layer mirror monochromated Mo-Kα radiation.

Cell constants and an orientation matrix for data collection corresponded to a primitive monoclinic cell with dimensions:

a = 20.433(2) Å

b = 7.6327(6) Å β = 106.660(3)^{o}

c = 21.563(2) Å

V = 3221.7(6) Å^3

For Z = 4 and F.W. = 579.77, the calculated density is 1.195 g/cm^3. The reflection conditions of:

h0l: l = 2n

0k0: k = 2n

uniquely determine the space group to be:

$P2_1/c$ (#14)

The data were collected at a temperature of -100 $\pm$ 1°C to a maximum 2θ value of 50.8^{o}. Readout was performed in the 0.172 mm pixel mode.

Data Reduction

Of the 37896 reflections were collected, where 5921 were unique (R_{int} = 0.0499); equivalent reflections were merged. Data were collected and processed using CrystalClear (Rigaku). [1]

The linear absorption coefficient, μ, for Mo-Kα radiation is 11.901 cm^{-1}. An empirical absorption correction was applied which resulted in transmission factors ranging from 0.867 to 0.976. The data were corrected for Lorentz and polarization effects.

Structure Solution and Refinement

The structure was solved by heavy-atom Patterson methods[2] and expanded using Fourier techniques. The non-hydrogen atoms were refined anisotropically. Hydrogen atoms were refined using the riding model. The final cycle of full-matrix least-squares refinement[3] on F^2 was based on 5921 observed reflections and 351 variable parameters and converged (largest parameter shift was 0.00 times its esd) with unweighted and weighted agreement factors of:

$$R1 = \Sigma \,||Fo| - |Fc|| \,/\, \Sigma\, |Fo| = 0.0448$$

$$wR2 = [\, \Sigma\, (\, w\, (Fo^2 - Fc^2)^2\,)/\, \Sigma\, w(Fo^2)^2]^{1/2} = 0.1139$$

The goodness of fit[4] was 1.03. Unit weights were used. Plots of $\Sigma\, w\, (|Fo| - |Fc|)^2$ versus $|Fo|$, reflection order in data collection, sin θ/λ and various classes of indices showed no unusual trends. The maximum and minimum peaks on the final difference Fourier map corresponded to 0.63 and -0.33 $e^-/Å^3$, respectively.

Neutral atom scattering factors were taken from International Tables for Crystallography (IT), Vol. C, Table 6.1.1.4 [5]. Anomalous dispersion effects were included in Fcalc[6]; the values for $\Delta f'$ and $\Delta f''$ were those of Creagh and McAuley[7]. The values for the mass attenuation coefficients are those of Creagh and Hubbell[8]. All calculations were performed using the CrystalStructure[9] crystallographic software package except for refinement, which was performed using SHELXL2013[10].

References

(1) CrystalClear: Data Collection and Processing Software, Rigaku Corporation (1998-2014). Tokyo 196-8666, Japan.

(2) PATTY: Beurskens, P.T., Admiraal, G., Behm, H., Beurskens, G., Smits, J.M.M. and Smykalla, C. (1991). Z. f. Kristallogr. Suppl.4, p.99.

(3) Least Squares function minimized: (SHELXL2013)

$\Sigma w(F_o^2 - F_c^2)^2$ where w = Least Squares weights.

(4) Goodness of fit is defined as:

$[\Sigma w(F_o^2 - F_c^2)^2/(N_o - N_v)]^{1/2}$

where: N_o = number of observations

N_V = number of variables

(5) International Tables for Crystallography, Vol.C (1992). Ed. A.J.C. Wilson, Kluwer Academic Publishers, Dordrecht, Netherlands, Table 6.1.1.4, pp. 572.

(6) Ibers, J. A. & Hamilton, W. C.; Acta Crystallogr., 17, 781 (1964).

(7) Creagh, D. C. & McAuley, W.J .; "International Tables for Crystallography", Vol C, (A.J.C. Wilson, ed.), Kluwer Academic Publishers, Boston, Table 4.2.6.8, pages 219-222 (1992).

(8) Creagh, D. C. & Hubbell, J.H..; "International Tables for Crystallography", Vol C, (A.J.C. Wilson, ed.), Kluwer Academic Publishers, Boston, Table 4.2.4.3, pages 200-206 (1992).

(9) CrystalStructure 4.1: Crystal Structure Analysis Package, Rigaku Corporation (2000-2014). Tokyo 196-8666, Japan.

(10) SHELXL2013: Sheldrick, G. M. (2008). Acta Cryst. A64, 112-122.

EXPERIMENTAL DETAILS

A. Crystal Data

Empirical Formula	$C_{35}H_{52}N_2Se$
Formula Weight	579.77
Crystal Color, Habit	colorless, prism
Crystal Dimensions	0.150 X 0.060 X 0.020 mm
Crystal System	monoclinic
Lattice Type	Primitive
Lattice Parameters	a = 20.433(2) Å
	b = 7.6327(6) Å
	c = 21.563(2) Å
	β = 106.660(3) ^{o}
	V = 3221.7(6) Å^3
Space Group	$P2_1/c$ (#14)
Z value	4

D_{calc}	1.195 g/cm^3
F_{000}	1240.00
μ(MoKα)	11.901 cm^{-1}

B. Intensity Measurements

Diffractometer	XtaLAB P200
Radiation	MoKα (λ = 0.71075 Å)
	multi-layer mirror monochromated
Voltage, Current	45kV, 66mA
Temperature	-100.0^{o}C
Detector Aperture	83.8 x 70.0 mm
Pixel Size	0.172 mm
$2\theta_{max}$	50.8^o
No. of Reflections Measured	Total: 37896
	Unique: 5921 (R_{int} = 0.0499)
Corrections	Lorentz-polarization
	Absorption
	(trans. factors: 0.867 - 0.976)

C. Structure Solution and Refinement

Structure Solution	Patterson Methods (DIRDIF99 PATTY)
Refinement	Full-matrix least-squares on F^2
Function Minimized	Σ w $(Fo^2 - Fc^2)^2$
Least Squares Weights	w = 1/ [$\sigma^2(Fo^2)$ + $(0.0500 \cdot P)^2$
	+ 3.3801 $\cdot$ P]

	where P = (Max(Fo^2,0) + $2Fc^2$)/3
$2\theta_{max}$ cutoff	50.8^o
Anomalous Dispersion	All non-hydrogen atoms
No. Observations (All reflections)	5921
No. Variables	351
Reflection/Parameter Ratio	16.87
Residuals: R1 (I>2.00σ(I))	0.0448
Residuals: R (All reflections)	0.0608
Residuals: wR2 (All reflections)	0.1139
Goodness of Fit Indicator	1.033
Max Shift/Error in Final Cycle	0.001
Maximum peak in Final Diff. Map	0.63 $e^-/Å^3$
Minimum peak in Final Diff. Map	-0.33 $e^-/Å^3$

[Se(SIMes)]

Experimental

Data Collection

A colorless chip crystal of $C_{21}H_{26}N_2Se$ having approximate dimensions of 0.120 x 0.080 x 0.060 mm was mounted in a loop. All measurements were made on a Rigaku XtaLAB P200 diffractometer using multi-layer mirror monochromated Mo-Kα radiation.

Cell constants and an orientation matrix for data collection corresponded to a primitive orthorhombic cell with dimensions:

a = 15.9194(19) Å

b = 7.4418(7) Å

c = 16.1915(19) Å

V = 1918.2(4) $Å^3$

For Z = 4 and F.W. = 385.41, the calculated density is 1.334 g/cm^3. The reflection conditions of:

0kl: k = 2n

h0l: l = 2n

hk0: h+k = 2n

uniquely determine the space group to be:

Pbcn (#60)

The data were collected at a temperature of -100 $\pm$ 1°C to a maximum 2θ value of 50.8^{o}. Readout was performed in the 0.172 mm pixel mode.

Data Reduction

Of the 21695 reflections were collected, where 1762 were unique (R_{int} = 0.0308); equivalent reflections were merged. Data were collected and processed using CrystalClear (Rigaku). [1]

The linear absorption coefficient, μ, for Mo-Kα radiation is 19.620 cm^{-1}. An empirical absorption correction was applied which resulted in transmission factors ranging from 0.822 to 0.889. The data were corrected for Lorentz and polarization effects.

Structure Solution and Refinement

The structure was solved by direct methods[2] and expanded using Fourier techniques. The non-hydrogen atoms were refined anisotropically. Hydrogen atoms were refined using the riding model. The final cycle of full-matrix least-squares refinement[3] on F^2 was based on 1762 observed reflections and 113 variable parameters and converged (largest parameter shift was 0.00 times its esd) with unweighted and weighted agreement factors of:

$$R1 = \Sigma \, ||Fo| - |Fc|| \, / \, \Sigma \, |Fo| = 0.0239$$

$$wR2 = [\, \Sigma \, (\, w \, (Fo^2 - Fc^2)^2 \,)/ \, \Sigma \, w(Fo^2)^2]^{1/2} = 0.0715$$

The goodness of fit[4] was 1.09. Unit weights were used. Plots of Σ w (|Fo| - |Fc|)^2 versus |Fo|, reflection order in data collection, sin θ/λ and various classes of indices showed no unusual trends. The maximum and minimum peaks on the final difference Fourier map corresponded to 0.21 and -0.36 e$^-$/Å^3, respectively.

Neutral atom scattering factors were taken from International Tables for Crystallography (IT), Vol. C, Table 6.1.1.4 [5]. Anomalous dispersion effects were included in Fcalc[6]; the values for Δf' and Δf" were those of Creagh and McAuley[7]. The values for the mass attenuation coefficients are those of Creagh and Hubbell[8]. All calculations were performed using the CrystalStructure[9] crystallographic software package except for refinement, which was performed using SHELXL2013[10].

References

(1) CrystalClear: Data Collection and Processing Software, Rigaku Corporation (1998-2014). Tokyo 196-8666, Japan.

(2) SHELXS2013: Sheldrick, G. M. (2008). Acta Cryst. A64, 112-122.

(3) Least Squares function minimized: (SHELXL2013)

$\Sigma w(F_o^2-F_c^2)^2$ where w = Least Squares weights.

(4) Goodness of fit is defined as:

$[\Sigma w(F_o^2-F_c^2)^2/(N_o-N_v)]^{1/2}$

where: N_o = number of observations

N_v = number of variables

(5) International Tables for Crystallography, Vol.C (1992). Ed. A.J.C. Wilson, Kluwer Academic Publishers, Dordrecht, Netherlands, Table 6.1.1.4, pp. 572.

(6) Ibers, J. A. & Hamilton, W. C.; Acta Crystallogr., 17, 781 (1964).

(7) Creagh, D. C. & McAuley, W.J .; "International Tables for Crystallography", Vol C, (A.J.C. Wilson, ed.), Kluwer Academic Publishers, Boston, Table 4.2.6.8, pages 219-222 (1992).

(8) Creagh, D. C. & Hubbell, J.H..; "International Tables for Crystallography", Vol C, (A.J.C. Wilson, ed.), Kluwer Academic Publishers, Boston, Table 4.2.4.3, pages 200-206 (1992).

(9) CrystalStructure 4.1: Crystal Structure Analysis Package, Rigaku Corporation (2000-2014). Tokyo 196-8666, Japan.

(10) SHELXL2013: Sheldrick, G. M. (2008). Acta Cryst. A64, 112-122.

EXPERIMENTAL DETAILS

A. Crystal Data

Empirical Formula	$C_{21}H_{26}N_2Se$
Formula Weight	385.41
Crystal Color, Habit	colorless, chip
Crystal Dimensions	0.120 X 0.080 X 0.060 mm
Crystal System	orthorhombic
Lattice Type	Primitive
Lattice Parameters	a = 15.9194(19) Å
	b = 7.4418(7) Å
	c = 16.1915(19) Å
	V = 1918.2(4) $Å^3$
Space Group	Pbcn (#60)
Z value	4
D_{calc}	1.334 g/cm^3
F_{000}	800.00
μ(MoKα)	19.620 cm^{-1}

B. Intensity Measurements

Diffractometer	XtaLAB P200
Radiation	MoKα (λ = 0.71075 Å)
	multi-layer mirror monochromated
Voltage, Current	45kV, 66mA
Temperature	-100.0^{o}C
Detector Aperture	83.8 x 70.0 mm

Pixel Size	0.172 mm
$2\theta_{max}$	50.8^{o}
No. of Reflections Measured	Total: 21695
	Unique: 1762 (R_{int} = 0.0308)
Corrections	Lorentz-polarization
	Absorption
	(trans. factors: 0.822 - 0.889)

C. Structure Solution and Refinement

Structure Solution	Direct Methods (SHELXS2013)
Refinement	Full-matrix least-squares on F^2
Function Minimized	Σ w $(Fo^2 - Fc^2)^2$
Least Squares Weights	w = 1/ [$\sigma^2(Fo^2)$ + $(0.0356 \cdot P)^2$
	+ 0.8647 · P]
	where P = $(Max(Fo^2,0) + 2Fc^2)/3$
$2\theta_{max}$ cutoff	50.8^{o}
Anomalous Dispersion	All non-hydrogen atoms
No. Observations (All reflections)	1762
No. Variables	113
Reflection/Parameter Ratio	15.59
Residuals: R1 ($I>2.00\sigma(I)$)	0.0239
Residuals: R (All reflections)	0.0295
Residuals: wR2 (All reflections)	0.0715
Goodness of Fit Indicator	1.093
Max Shift/Error in Final Cycle	0.001

Maximum peak in Final Diff. Map 0.21 $e^-/Å^3$

Minimum peak in Final Diff. Map -0.36 $e^-/Å^3$

[Se(Tr2)]

Experimental

Data Collection

A colorless prism crystal of $C_{11}H_6F_5N_3Se$ having approximate dimensions of 0.210 x 0.120 x 0.030 mm was mounted in a loop. All measurements were made on a Rigaku XtaLAB P200 diffractometer using multi-layer mirror monochromated Mo-Kα radiation.

Cell constants and an orientation matrix for data collection corresponded to a primitive monoclinic cell with dimensions:

a = 9.3746(16) Å

b = 15.527(3) Å β = 90.357(5)^o

c = 8.3524(14) Å

V = 1215.7(3) $Å^3$

For Z = 4 and F.W. = 354.14, the calculated density is 1.935 g/cm^3. The reflection conditions of:

h0l: l = 2n

0k0: k = 2n

uniquely determine the space group to be:

$P2_1/c$ (#14)

The data were collected at a temperature of -100 $\pm$ 1°C to a maximum 2θ value of 50.8^o. Readout was performed in the 0.172 mm pixel mode.

Data Reduction

Of the 14536 reflections were collected, where 2221 were unique (R_{int} = 0.0864); equivalent reflections were merged. Data were collected and processed using CrystalClear (Rigaku). [1]

The linear absorption coefficient, μ, for Mo-Kα radiation is 31.448 cm^{-1}. An empirical absorption correction was applied which resulted in transmission factors ranging from 0.621 to 0.910. The data were corrected for Lorentz and polarization effects.

Structure Solution and Refinement

The structure was solved by heavy-atom Patterson methods[2] and expanded using Fourier techniques. The non-hydrogen atoms were refined anisotropically. Hydrogen atoms were refined using the riding model. The final cycle of full-matrix least-squares refinement[3] on F^2 was based on 2221 observed reflections and 181 variable parameters and converged (largest parameter shift was 0.00 times its esd) with unweighted and weighted agreement factors of:

$$R1 = \Sigma \,||Fo| - |Fc|| \,/\, \Sigma \,|Fo| = 0.0480$$

$$wR2 = [\,\Sigma \,(w\,(Fo^2 - Fc^2)^2\,) / \Sigma \,w(Fo^2)^2]^{1/2} = 0.1182$$

The goodness of fit[4] was 0.96. Unit weights were used. Plots of Σ w $(|Fo| - |Fc|)^2$ versus |Fo|, reflection order in data collection, sin θ/λ and various classes of indices showed no unusual trends. The maximum and minimum peaks on the final difference Fourier map corresponded to 1.30 and -0.30 $e^-/Å^3$, respectively.

Neutral atom scattering factors were taken from International Tables for Crystallography (IT), Vol. C, Table 6.1.1.4 [5]. Anomalous dispersion effects were included in Fcalc[6]; the values for $\Delta f'$ and $\Delta f''$ were those of Creagh and McAuley[7]. The values for the mass attenuation coefficients are those of Creagh and Hubbell[8]. All calculations were performed using the CrystalStructure[9] crystallographic software package except for refinement, which was performed using SHELXL2013[10].

References

(1) CrystalClear: Data Collection and Processing Software, Rigaku Corporation (1998-2014). Tokyo 196-8666, Japan.

(2) PATTY: Beurskens, P.T., Admiraal, G., Behm, H., Beurskens, G., Smits, J.M.M. and Smykalla, C. (1991). Z. f. Kristallogr. Suppl.4, p.99.

(3) Least Squares function minimized: (SHELXL2013)

$$\Sigma w(F_o^2 - F_c^2)^2 \quad \text{where w = Least Squares weights.}$$

(4) Goodness of fit is defined as:

$$[\Sigma w(F_o^2 - F_c^2)^2/(N_o - N_v)]^{1/2}$$

where: N_o = number of observations

N_v = number of variables

(5) International Tables for Crystallography, Vol.C (1992). Ed. A.J.C. Wilson, Kluwer Academic Publishers, Dordrecht, Netherlands, Table 6.1.1.4, pp. 572.

(6) Ibers, J. A. & Hamilton, W. C.; Acta Crystallogr., 17, 781 (1964).

(7) Creagh, D. C. & McAuley, W.J .; "International Tables for Crystallography", Vol C, (A.J.C. Wilson, ed.), Kluwer Academic Publishers, Boston, Table 4.2.6.8, pages 219-222 (1992).

(8) Creagh, D. C. & Hubbell, J.H..; "International Tables for Crystallography", Vol C, (A.J.C. Wilson, ed.), Kluwer Academic Publishers, Boston, Table 4.2.4.3, pages 200-206 (1992).

(9) CrystalStructure 4.1: Crystal Structure Analysis Package, Rigaku Corporation (2000-2014). Tokyo 196-8666, Japan.

(10) SHELXL2013: Sheldrick, G. M. (2008). Acta Cryst. A64, 112-122.

EXPERIMENTAL DETAILS

A. Crystal Data

Empirical Formula	$C_{11}H_6F_5N_3Se$
Formula Weight	354.14
Crystal Color, Habit	colorless, prism
Crystal Dimensions	0.210 X 0.120 X 0.030 mm
Crystal System	monoclinic
Lattice Type	Primitive

Lattice Parameters	a = 9.3746(16) Å
	b = 15.527(3) Å
	c = 8.3524(14) Å
	β = 90.357(5) ^{o}
	V = 1215.7(3) Å^{3}
Space Group	P2_1/c (#14)
Z value	4
D_{calc}	1.935 g/cm^{3}
F_{000}	688.00
μ(MoKα)	31.448 cm^{-1}

B. Intensity Measurements

Diffractometer	XtaLAB P200
Radiation	MoKα (λ = 0.71075 Å)
	multi-layer mirror monochromated
Voltage, Current	45kV, 66mA
Temperature	-100.0^{o}C
Detector Aperture	83.8 x 70.0 mm
Pixel Size	0.172 mm
$2\theta_{max}$	50.8^{o}
No. of Reflections Measured	Total: 14536
	Unique: 2221 (R_{int} = 0.0864)
Corrections	Lorentz-polarization
	Absorption
	(trans. factors: 0.621 - 0.910)

C. Structure Solution and Refinement

Structure Solution	Patterson Methods (DIRDIF99 PATTY)
Refinement	Full-matrix least-squares on F^2
Function Minimized	Σ w $(Fo^2 - Fc^2)^2$
Least Squares Weights	w = 1/ [$\sigma^2(Fo^2)$ + $(0.0749 \cdot P)^2$
	+ 0.0000 · P]
	where P = (Max(Fo^2,0) + $2Fc^2$)/3
$2\theta_{max}$ cutoff	50.8^{o}
Anomalous Dispersion	All non-hydrogen atoms
No. Observations (All reflections)	2221
No. Variables	181
Reflection/Parameter Ratio	12.27
Residuals: R1 (I>2.00σ(I))	0.0480
Residuals: R (All reflections)	0.0679
Residuals: wR2 (All reflections)	0.1182
Goodness of Fit Indicator	0.958
Max Shift/Error in Final Cycle	0.000
Maximum peak in Final Diff. Map	1.30 $e^-/Å^3$
Minimum peak in Final Diff. Map	-0.30 $e^-/Å^3$

[Se(Tr3)]

Experimental

Data Collection

A colorless platelet crystal of $C_{11}H_{11}N_3OSe$ having approximate dimensions of 0.100 x 0.090 x 0.020 mm was mounted in a loop. All measurements were made on a Rigaku XtaLAB P200 diffractometer using multi-layer mirror monochromated Mo-Kα radiation.

Cell constants and an orientation matrix for data collection corresponded to a primitive triclinic cell with dimensions:

$a = 7.8283(13)$ Å $\alpha = 97.930(6)^{o}$

$b = 7.9731(14)$ Å $\beta = 94.637(5)^{o}$

$c = 18.447(3)$ Å $\gamma = 90.042(5)^{o}$

$V = 1136.6(4)$ Å^3

For Z = 4 and F.W. = 280.19, the calculated density is 1.637 g/cm^3. Based on a statistical analysis of intensity distribution, and the successful solution and refinement of the structure, the space group was determined to be:

P-1 (#2)

The data were collected at a temperature of -100 $\pm$ 1°C to a maximum 2θ value of 51.0^{o}. Readout was performed in the 0.172 mm pixel mode.

Data Reduction

Of the 13206 reflections were collected, where 4171 were unique (R_{int} = 0.1195); equivalent reflections were merged. Data were collected and processed using CrystalClear (Rigaku). [1]

The linear absorption coefficient, μ, for Mo-Kα radiation is 32.859 cm^{-1}. An empirical absorption correction was applied which resulted in transmission factors ranging from 0.536 to 0.936. The data were corrected for Lorentz and polarization effects.

Structure Solution and Refinement

The structure was solved by heavy-atom Patterson methods[2] and expanded using Fourier techniques.

The crystal is a non-merohedral twin with twin law:

1.00000	0.00000	0.00000
0.00000	-1.00000	0.00000
0.38100	0.63900	1.00000

Twin component #1 comprises 12.02% of the crystal.

The non-hydrogen atoms were refined anisotropically. Hydrogen atoms were refined using the riding model. The final cycle of full-matrix least-squares refinement[3] on F^2 was based on 4171 observed reflections and 290 variable parameters and converged (largest parameter shift was 0.00 times its esd) with unweighted and weighted agreement factors of:

$$R1 = \Sigma \,||Fo| - |Fc|| \,/\, \Sigma\, |Fo| = 0.1235$$

$$wR2 = [\, \Sigma\, (w (Fo^2 - Fc^2)^2)/ \Sigma\, w(Fo^2)^2]^{1/2} = 0.3513$$

The goodness of fit[4] was 1.17. Unit weights were used. Plots of $\Sigma\, w\, (|Fo| - |Fc|)^2$ versus $|Fo|$, reflection order in data collection, sin θ/λ and various classes of indices showed no unusual trends. The maximum and minimum peaks on the final difference Fourier map corresponded to 2.49 and -1.17 $e^-/Å^3$, respectively.

Neutral atom scattering factors were taken from International Tables for Crystallography (IT), Vol. C, Table 6.1.1.4 [5]. Anomalous dispersion effects were included in Fcalc[6]; the values for $\Delta f'$ and $\Delta f''$ were those of Creagh and McAuley[7]. The values for the mass attenuation coefficients are those of Creagh and Hubbell[8]. All calculations were performed using the CrystalStructure[9] crystallographic software package except for refinement, which was performed using SHELXL2013[10].

References

(1) CrystalClear: Data Collection and Processing Software, Rigaku Corporation (1998-2014). Tokyo 196-8666, Japan.

(2) PATTY: Beurskens, P.T., Admiraal, G., Behm, H., Beurskens, G., Smits, J.M.M. and Smykalla, C. (1991). Z. f. Kristallogr. Suppl.4, p.99.

(3) Least Squares function minimized: (SHELXL2013)

$\Sigma w(F_o^2-F_c^2)^2$ where w = Least Squares weights.

(4) Goodness of fit is defined as:

$[\Sigma w(F_o^2-F_c^2)^2/(N_o-N_v)]^{1/2}$

where: N_o = number of observations

N_v = number of variables

(5) International Tables for Crystallography, Vol.C (1992). Ed. A.J.C. Wilson, Kluwer Academic Publishers, Dordrecht, Netherlands, Table 6.1.1.4, pp. 572.

(6) Ibers, J. A. & Hamilton, W. C.; Acta Crystallogr., 17, 781 (1964).

(7) Creagh, D. C. & McAuley, W.J .; "International Tables for Crystallography", Vol C, (A.J.C. Wilson, ed.), Kluwer Academic Publishers, Boston, Table 4.2.6.8, pages 219-222 (1992).

(8) Creagh, D. C. & Hubbell, J.H..; "International Tables for Crystallography", Vol C, (A.J.C. Wilson, ed.), Kluwer Academic Publishers, Boston, Table 4.2.4.3, pages 200-206 (1992).

(9) CrystalStructure 4.1: Crystal Structure Analysis Package, Rigaku Corporation (2000-2014). Tokyo 196-8666, Japan.

(10) SHELXL2013: Sheldrick, G. M. (2008). Acta Cryst. A64, 112-122.

EXPERIMENTAL DETAILS

A. Crystal Data

Empirical Formula	$C_{11}H_{11}N_3OSe$
Formula Weight	280.19
Crystal Color, Habit	colorless, platelet
Crystal Dimensions	0.100 X 0.090 X 0.020 mm
Crystal System	triclinic
Lattice Type	Primitive
Lattice Parameters	a = 7.8283(13) Å
	b = 7.9731(14) Å
	c = 18.447(3) Å
	α = 97.930(6) ^{o}
	β = 94.637(5) ^{o}

	γ = 90.042(5) ^{o}
	V = 1136.6(4) Å^3
Space Group	P-1 (#2)
Z value	4
D_{calc}	1.637 g/cm^3
F_{000}	560.00
μ(MoKα)	32.859 cm^{-1}

B. Intensity Measurements

Diffractometer	XtaLAB P200
Radiation	MoKα (λ = 0.71075 Å)
	multi-layer mirror monochromated
Voltage, Current	45kV, 66mA
Temperature	-100.0^{o}C
Detector Aperture	83.8 x 70.0 mm
Pixel Size	0.172 mm
$2\theta_{max}$	51.0^{o}
No. of Reflections Measured	Total: 13206
	Unique: 4171 (R_{int} = 0.1195)
Corrections	Lorentz-polarization
	Absorption
	(trans. factors: 0.536 - 0.936)

C. Structure Solution and Refinement

Structure Solution	Patterson Methods (DIRDIF99 PATTY)

Refinement	Full-matrix least-squares on F^2
Function Minimized	Σ w $(Fo^2 - Fc^2)^2$
Least Squares Weights	w = 1/ [$\sigma^2(Fo^2)$ + $(0.2000 \cdot P)^2$
	+ 0.0000 · P]
	where P = (Max(Fo^2,0) + 2Fc^2)/3
$2\theta_{max}$ cutoff	51.0°
Anomalous Dispersion	All non-hydrogen atoms
No. Observations (All reflections)	4171
No. Variables	290
Reflection/Parameter Ratio	14.38
Residuals: R1 (I>2.00σ(I))	0.1235
Residuals: R (All reflections)	0.1583
Residuals: wR2 (All reflections)	0.3513
Goodness of Fit Indicator	1.165
Max Shift/Error in Final Cycle	0.000
Maximum peak in Final Diff. Map	2.49 $e^-/Å^3$
Minimum peak in Final Diff. Map	-1.17 $e^-/Å^3$

[Se(Tr4)]

Experimental

Data Collection

A colorless prism crystal of $C_{21}H_{21}N_3OSe$ having approximate dimensions of 0.210 x 0.090 x 0.040 mm was mounted in a loop. All measurements were made on a Rigaku XtaLAB P200 diffractometer using multi-layer mirror monochromated Mo-Kα radiation.

The crystal-to-detector distance was 44.99 mm.

Cell constants and an orientation matrix for data collection corresponded to a primitive triclinic cell with dimensions:

a = 8.5326(19) Å α = 107.558(4)^{o}

b = 10.637(3) Å β = 101.546(4)^{o}

c = 11.589(3) Å γ = 107.498(3)^{o}

V = 906.2(4) Å^{3}

For Z = 2 and F.W. = 410.38, the calculated density is 1.504 g/cm^{3}. Based on a statistical analysis of intensity distribution, and the successful solution and refinement of the structure, the space group was determined to be:

P-1 (#2)

The data were collected at a temperature of -100 $\pm$ 1°C to a maximum 2θ value of 50.8^{o}. A total of 1080 oscillation images were collected. A sweep of data was done using ω scans from -100.0 to 80.0^{o} in 0.50^{o} step, at χ=45.0^{o} and ϕ = 0.0^{o}. The exposure rate was 10.0 [sec./^{o}]. The detector swing angle was -10.43^{o}. A second sweep was performed using ω scans from -100.0 to 80.0^{o} in 0.50^{o} step, at χ=45.0^{o} and ϕ = 90.0^{o}. The exposure rate was 10.0 [sec./^{o}]. The detector swing angle was -10.43^{o}. Another sweep was performed using ω scans from -100.0 to 80.0^{o} in 0.50^{o} step, at χ=45.0^{o} and ϕ = 180.0^{o}. The exposure rate was 10.0 [sec./^{o}]. The detector swing angle was -10.43^{o}. The crystal-to-detector distance was 44.99 mm. Readout was performed in the 0.086 mm pixel mode.

Data Reduction

Of the 11092 reflections were collected, where 3280 were unique (R_{int} = 0.0454); equivalent reflections were merged. Data were collected and processed using CrystalClear (Rigaku). [1]

The linear absorption coefficient, μ, for Mo-Kα radiation is 20.874 cm^{-1}. An empirical absorption correction was applied which resulted in transmission factors ranging from 0.654 to 0.920. The data were corrected for Lorentz and polarization effects.

Structure Solution and Refinement

The structure was solved by heavy-atom Patterson methods[2] and expanded using Fourier techniques. The non-hydrogen atoms were refined anisotropically. Hydrogen atoms were

refined using the riding model. The final cycle of full-matrix least-squares refinement[3] on F^2 was based on 3280 observed reflections and 238 variable parameters and converged (largest parameter shift was 0.00 times its esd) with unweighted and weighted agreement factors of:

$$R1 = \Sigma ||Fo| - |Fc|| / \Sigma |Fo| = 0.0241$$

$$wR2 = [\Sigma (w (Fo^2 - Fc^2)^2)/ \Sigma w(Fo^2)^2]^{1/2} = 0.0546$$

The goodness of fit[4] was 0.98. Unit weights were used. Plots of Σ w $(|Fo| - |Fc|)^2$ versus |Fo|, reflection order in data collection, sin θ/λ and various classes of indices showed no unusual trends. The maximum and minimum peaks on the final difference Fourier map corresponded to 0.35 and -0.41 $e^-/Å^3$, respectively.

Neutral atom scattering factors were taken from International Tables for Crystallography (IT), Vol. C, Table 6.1.1.4 [5]. Anomalous dispersion effects were included in Fcalc[6]; the values for $\Delta f'$ and $\Delta f''$ were those of Creagh and McAuley[7]. The values for the mass attenuation coefficients are those of Creagh and Hubbell[8]. All calculations were performed using the CrystalStructure[9] crystallographic software package except for refinement, which was performed using SHELXL2013[10].

References

(1) CrystalClear: Data Collection and Processing Software, Rigaku Corporation (1998-2014). Tokyo 196-8666, Japan.

(2) PATTY: Beurskens, P.T., Admiraal, G., Behm, H., Beurskens, G., Smits, J.M.M. and Smykalla, C. (1991). Z. f. Kristallogr. Suppl.4, p.99.

(3) Least Squares function minimized: (SHELXL2013)

$\Sigma w(F_o^2-F_c^2)^2$ where w = Least Squares weights.

(4) Goodness of fit is defined as:

$[\Sigma w(F_o^2-F_c^2)^2/(N_o-N_v)]^{1/2}$

where: N_o = number of observations

N_v = number of variables

(5) International Tables for Crystallography, Vol.C (1992). Ed. A.J.C. Wilson, Kluwer Academic Publishers, Dordrecht, Netherlands, Table 6.1.1.4, pp. 572.

(6) Ibers, J. A. & Hamilton, W. C.; Acta Crystallogr., 17, 781 (1964).

(7) Creagh, D. C. & McAuley, W.J .; "International Tables for Crystallography", Vol C, (A.J.C. Wilson, ed.), Kluwer Academic Publishers, Boston, Table 4.2.6.8, pages 219-222 (1992).

(8) Creagh, D. C. & Hubbell, J.H..; "International Tables for Crystallography", Vol C, (A.J.C. Wilson, ed.), Kluwer Academic Publishers, Boston, Table 4.2.4.3, pages 200-206 (1992).

(9) CrystalStructure 4.1: Crystal Structure Analysis Package, Rigaku Corporation (2000-2014). Tokyo 196-8666, Japan.

(10) SHELXL2013: Sheldrick, G. M. (2008). Acta Cryst. A64, 112-122.

EXPERIMENTAL DETAILS

A. Crystal Data

Empirical Formula	$C_{21}H_{21}N_3OSe$
Formula Weight	410.38
Crystal Color, Habit	colorless, prism
Crystal Dimensions	0.210 X 0.090 X 0.040 mm
Crystal System	triclinic
Lattice Type	Primitive
Lattice Parameters	a = 8.5326(19) Å
	b = 10.637(3) Å
	c = 11.589(3) Å
	α = 107.558(4) ^{o}
	β = 101.546(4) ^{o}
	γ = 107.498(3) ^{o}
	V = 906.2(4) $Å^3$
Space Group	P-1 (#2)
Z value	2

D_{calc}	1.504 g/cm^3
F_{000}	420.00
μ(MoKα)	20.874 cm^{-1}

B. Intensity Measurements

Diffractometer	XtaLAB P200
Radiation	MoKα (λ = 0.71075 Å)
	multi-layer mirror monochromated
Voltage, Current	45kV, 66mA
Temperature	-100.0^{o}C
Detector Aperture	83.8 x 70.0 mm
Data Images	1080 exposures
ω oscillation Range (χ=45.0, ϕ=0.0)	-100.0 - 80.0^{o}
Exposure Rate	10.0 sec./^{o}
Detector Swing Angle	-10.43^{o}
ω oscillation Range (χ=45.0, ϕ=90.0)	-100.0 - 80.0^{o}
Exposure Rate	10.0 sec./^{o}
Detector Swing Angle	-10.43^{o}
ω oscillation Range (χ=45.0, ϕ=180.0)	-100.0 - 80.0^{o}
Exposure Rate	10.0 sec./^{o}
Detector Swing Angle	-10.43^{o}
Detector Position	44.99 mm
Pixel Size	0.086 mm
$2\theta_{max}$	50.8^{o}

No. of Reflections Measured	Total: 11092
	Unique: 3280 (R_{int} = 0.0454)
Corrections	Lorentz-polarization
	Absorption
	(trans. factors: 0.654 - 0.920)

C. Structure Solution and Refinement

Structure Solution	Patterson Methods (DIRDIF99 PATTY)
Refinement	Full-matrix least-squares on F^2
Function Minimized	Σ w $(Fo^2 - Fc^2)^2$
Least Squares Weights	w = 1/ [$\sigma^2(Fo^2)$ + $(0.0191 \cdot P)^2$
	+ 0.0000 · P]
	where P = $(Max(Fo^2,0) + 2Fc^2)/3$
$2\theta_{max}$ cutoff	50.8^o
Anomalous Dispersion	All non-hydrogen atoms
No. Observations (All reflections)	3280
No. Variables	238
Reflection/Parameter Ratio	13.78
Residuals: R1 (I>2.00σ(I))	0.0241
Residuals: R (All reflections)	0.0265
Residuals: wR2 (All reflections)	0.0546
Goodness of Fit Indicator	0.985
Max Shift/Error in Final Cycle	0.000
Maximum peak in Final Diff. Map	0.35 $e^-/Å^3$
Minimum peak in Final Diff. Map	-0.41 $e^-/Å^3$

[Se(Tr5)]

Experimental

Data Collection

A colorless prism crystal of $C_{30}H_{35}N_3OSeSi$ having approximate dimensions of 0.100 x 0.100 x 0.100 mm was mounted in a loop. All measurements were made on a Rigaku XtaLAB P200 diffractometer using multi-layer mirror monochromated Mo-Kα radiation.

The crystal-to-detector distance was 45.02 mm.

Cell constants and an orientation matrix for data collection corresponded to a primitive orthorhombic cell with dimensions:

a = 13.2692(12) Å

b = 19.8926(16) Å

c = 20.9790(19) Å

V = 5537.6(8) Å^3

For Z = 8 and F.W. = 560.67, the calculated density is 1.345 g/cm^3. The reflection conditions of:

0kl: k = 2n

h0l: l = 2n

hk0: h = 2n

uniquely determine the space group to be:

Pbca (#61)

The data were collected at a temperature of -100 $\pm$ 1°C to a maximum 2θ value of 50.7^{o}. A total of 1080 oscillation images were collected. A sweep of data was done using ω scans from -100.0 to 80.0^{o} in 0.50^{o} step, at χ=45.0^{o} and ϕ = 0.0^{o}. The exposure rate was 20.0 [sec./^{o}]. The detector swing angle was -10.42^{o}. A second sweep was performed using ω scans from -100.0 to 80.0^{o} in 0.50^{o} step, at χ=45.0^{o} and ϕ = 90.0^{o}. The exposure rate was 20.0 [sec./^{o}]. The detector swing angle was -10.42^{o}. Another sweep was performed using ω scans from -100.0 to 80.0^{o} in 0.50^{o} step, at χ=45.0^{o} and ϕ = 180.0^{o}. The exposure rate was 20.0 [sec./^{o}]. The detector swing angle was -10.42^{o}. The crystal-to-detector distance was 45.02 mm. Readout was performed in the 0.086 mm pixel mode.

Data Reduction

Of the 64470 reflections were collected, where 5063 were unique (R_{int} = 0.0445); equivalent reflections were merged. Data were collected and processed using CrystalClear (Rigaku). [1]

The linear absorption coefficient, μ, for Mo-Kα radiation is 14.272 cm^{-1}. An empirical absorption correction was applied which resulted in transmission factors ranging from 0.824 to 0.867. The data were corrected for Lorentz and polarization effects.

Structure Solution and Refinement

The structure was solved by heavy-atom Patterson methods[2] and expanded using Fourier techniques. The non-hydrogen atoms were refined anisotropically. Hydrogen atoms were refined using the riding model. The final cycle of full-matrix least-squares refinement[3] on F^2 was based on 5063 observed reflections and 330 variable parameters and converged (largest parameter shift was 0.00 times its esd) with unweighted and weighted agreement factors of:

$$R1 = \Sigma ||Fo| - |Fc|| / \Sigma |Fo| = 0.0248$$

$$wR2 = [\Sigma (w (Fo^2 - Fc^2)^2)/ \Sigma w(Fo^2)^2]^{1/2} = 0.0616$$

The goodness of fit[4] was 1.05. Unit weights were used. Plots of Σ w $(|Fo| - |Fc|)^2$ versus $|Fo|$, reflection order in data collection, sin θ/λ and various classes of indices showed no unusual trends. The maximum and minimum peaks on the final difference Fourier map corresponded to 0.29 and -0.25 $e^-/Å^3$, respectively.

Neutral atom scattering factors were taken from International Tables for Crystallography (IT), Vol. C, Table 6.1.1.4 [5]. Anomalous dispersion effects were included in Fcalc[6]; the values for $\Delta f'$ and $\Delta f''$ were those of Creagh and McAuley[7]. The values for the mass attenuation coefficients are those of Creagh and Hubbell[8]. All calculations were performed using the CrystalStructure[9] crystallographic software package except for refinement, which was performed using SHELXL2013[10].

References

(1) CrystalClear: Data Collection and Processing Software, Rigaku Corporation (1998-2014). Tokyo 196-8666, Japan.

(2) PATTY: Beurskens, P.T., Admiraal, G., Behm, H., Beurskens, G., Smits, J.M.M. and Smykalla, C. (1991). Z. f. Kristallogr. Suppl.4, p.99.

(3) Least Squares function minimized: (SHELXL2013)

$\Sigma w(F_o^2-F_c^2)^2$ where w = Least Squares weights.

(4) Goodness of fit is defined as:

$[\Sigma w(F_o^2-F_c^2)^2/(N_o-N_v)]^{1/2}$

where: N_o = number of observations

N_v = number of variables

(5) International Tables for Crystallography, Vol.C (1992). Ed. A.J.C. Wilson, Kluwer Academic Publishers, Dordrecht, Netherlands, Table 6.1.1.4, pp. 572.

(6) Ibers, J. A. & Hamilton, W. C.; Acta Crystallogr., 17, 781 (1964).

(7) Creagh, D. C. & McAuley, W.J .; "International Tables for Crystallography", Vol C, (A.J.C. Wilson, ed.), Kluwer Academic Publishers, Boston, Table 4.2.6.8, pages 219-222 (1992).

(8) Creagh, D. C. & Hubbell, J.H..; "International Tables for Crystallography", Vol C, (A.J.C. Wilson, ed.), Kluwer Academic Publishers, Boston, Table 4.2.4.3, pages 200-206 (1992).

(9) CrystalStructure 4.1: Crystal Structure Analysis Package, Rigaku Corporation (2000-2014). Tokyo 196-8666, Japan.

(10) SHELXL2013: Sheldrick, G. M. (2008). Acta Cryst. A64, 112-122.

EXPERIMENTAL DETAILS

A. Crystal Data

Empirical Formula	$C_{30}H_{35}N_3OSeSi$
Formula Weight	560.67
Crystal Color, Habit	colorless, prism
Crystal Dimensions	0.100 X 0.100 X 0.100 mm

Crystal System	orthorhombic
Lattice Type	Primitive
Lattice Parameters	a = 13.2692(12) Å
	b = 19.8926(16) Å
	c = 20.9790(19) Å
	V = 5537.6(8) $Å^3$
Space Group	Pbca (#61)
Z value	8
D_{calc}	1.345 g/cm^3
F_{000}	2336.00
μ(MoKα)	14.272 cm^{-1}

B. Intensity Measurements

Diffractometer	XtaLAB P200
Radiation	MoKα (λ = 0.71075 Å)
	multi-layer mirror monochromated
Voltage, Current	45kV, 66mA
Temperature	-100.0^{o}C
Detector Aperture	83.8 x 70.0 mm
Data Images	1080 exposures
ω oscillation Range (χ=45.0, ϕ=0.0)	-100.0 - 80.0^{o}
Exposure Rate	20.0 sec./^{o}
Detector Swing Angle	-10.42^{o}
ω oscillation Range (χ=45.0, ϕ=90.0)	-100.0 - 80.0^{o}
Exposure Rate	20.0 sec./^{o}

Detector Swing Angle	-10.42^{o}
ω oscillation Range (χ=45.0, ϕ=180.0)	-100.0 - 80.0^{o}
Exposure Rate	20.0 sec./^{o}
Detector Swing Angle	-10.42^{o}
Detector Position	45.02 mm
Pixel Size	0.086 mm
$2\theta_{max}$	50.7^{o}
No. of Reflections Measured	Total: 64470
	Unique: 5063 (R_{int} = 0.0445)
Corrections	Lorentz-polarization
	Absorption
	(trans. factors: 0.824 - 0.867)

C. Structure Solution and Refinement

Structure Solution	Patterson Methods (DIRDIF99 PATTY)
Refinement	Full-matrix least-squares on F^2
Function Minimized	Σ w $(Fo^2 - Fc^2)^2$
Least Squares Weights	w = 1/ [$\sigma^2(Fo^2)$ + $(0.0272 \cdot P)^2$
	+ 2.7260 · P]
	where P = (Max(Fo^2,0) + 2Fc^2)/3
$2\theta_{max}$ cutoff	50.7^{o}
Anomalous Dispersion	All non-hydrogen atoms
No. Observations (All reflections)	5063
No. Variables	330
Reflection/Parameter Ratio	15.34

Residuals: R1 ($I>2.00\sigma(I)$)	0.0248
Residuals: R (All reflections)	0.0325
Residuals: wR2 (All reflections)	0.0616
Goodness of Fit Indicator	1.054
Max Shift/Error in Final Cycle	0.003
Maximum peak in Final Diff. Map	0.29 $e^-/Å^3$
Minimum peak in Final Diff. Map	-0.25 $e^-/Å^3$

Computational Details

All calculations have been performed using density functional theory (DFT) with the gradient corrections for exchange and correlation proposed by Becke[9] and Perdew[10] (BP86), as implemented in the ADF package,[11-13] in combination with a fine integration parameter (with a numerical integration parameter set to 5). A triple-ξ basis set with two polarization functions on all atoms (TZ2P) was used. Electrons of the core shells have been treated within the frozen core approximation.[14] All geometries were initially optimized without any symmetry constraint. These geometries were used to obtain the Se/P NMR chemical shielding properties, and the molecular orbital energy gap [Se(p_y) → Se-NHC(π^*)] in the case of selenoureas. Atomic partial charges have been calculated with the Hirshfeld partitioning scheme.[15]

As discussed in the main text, the major changes to the total isotropic shielding between the different systems are due to changes in the paramagnetic component (σ_p). To understand the impact of the nature of the NHC ligand on the Se paramagnetic shielding, we thus compared three systems chosen to cover the NMR scale of **Figure 2**. Namely, **I^{i}Pr^{Me}**, **SIMes** and **SIPr**, with σ_{Se} equal to −18, 110 and 190 ppm, respectively. For the sake of simplicity in the analysis, the systems were re-optimized under the constraint of C_S symmetry, with the NHC ligand located in the σ_{xy} mirror plane of the molecule. As indicated in the main text, this results in minor differences in energy and NMR total isotropic shielding.

The principal components of total paramagnetic tensor of the Se atom in the three systems, reported in **Table S1**, clearly indicates that the σ_{yy} and σ_{zz} components in the three systems are roughly the same, with a change of only 80 ppm in σ_{yy}, while the major change is in the σ_{xx} component, that spans a range of about 700 ppm, and nicely matching the trend in the experimental δ_{Se} values.

Table S1. Experimental chemical shift and principal components of total DFT paramagnetic tensor of the Se atom in selected NHC-Se adducts. Systems oriented as in **Figure S1**.

System	δ_{Se} (ppm)	σ_{xx} (ppm)	σ_{yy} (ppm)	σ_{zz} (ppm)
**I^{i}Pr**	-18	-1062	-1947	-1177
**SIMes**	110	-1394	-2028	-1194
**SIPr**	190	-1751	-2027	-1190

Analysis of the orbitals contributing to the change of the σ_{xx} component of the paramagnetic tensor indicated that the major contributions stem from coupling of occupied and virtual orbitals, specifically from the Se(p_y) → Se-NHC(π^*) transition sketched in **Figure S1**.

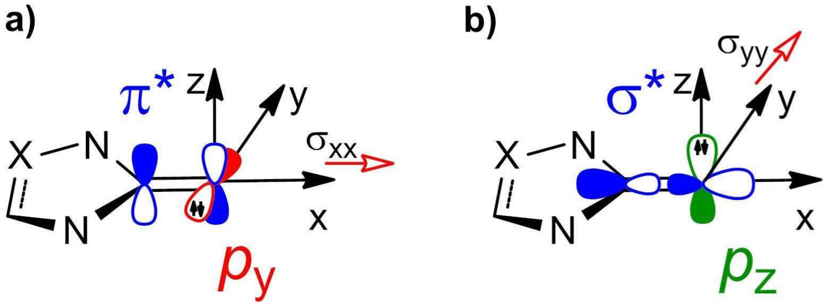

Figure S1. (a) Schematic representation of the occupied-virtual Se(p_y) → Se-NHC(π*) transition, which contributes to the σ_{xx} component of the paramagnetic tensor. (b) Schematic representation of the occupied-virtual Se(p_z) → NHC-Se(σ*) orbital, which contributes to the σ_{yy} component of the paramagnetic tensor.

However, in the case of the **I*i*Pr^{Me}** system the Se-NHC(π*) orbital combination is well localized in a single molecular orbital, see **Figure 6c** in the main text, and thus only one Se(p_y) → Se-NHC(π*) transition contributes to the σ_{xx} component. Differently, in the **SIMes** and **SIPr** systems, the Se-NHC(π*) orbital combination is spread over at least two virtual molecular orbitals, see **Figure S2**, and both these virtual orbitals participate to the σ_{xx} component of the paramagnetic tensor through Se(p_y) → Se-NHC(π*) transitions. Finally, the large value of the σ_{yy} component of the paramagnetic tensor corresponds to a transition from the occupied Se(p_z) orbital to the a NHC-Se(σ*) orbital, see **Figure S1**, and it is quite independent of the nature of the NHC ligand. In conclusion, this analysis indicates that variation in the Se chemical shielding are related to the Se(p_y) → Se-NHC(π*) energy gap, supporting the analysis performed in **Figure 7** of the main text. For completeness, the above orbital analysis and decomposition of the total paramagnetic shielding has been performed on the optimized geometries through single point energy calculations using all electrons basis sets and scalar relativistic ZORA effects.[16-20] Further, the energy gaps of **Figure 7** have been evaluated by including solvent effects, $CHCl_3$, through the continuum solvation model COSMO.[21]

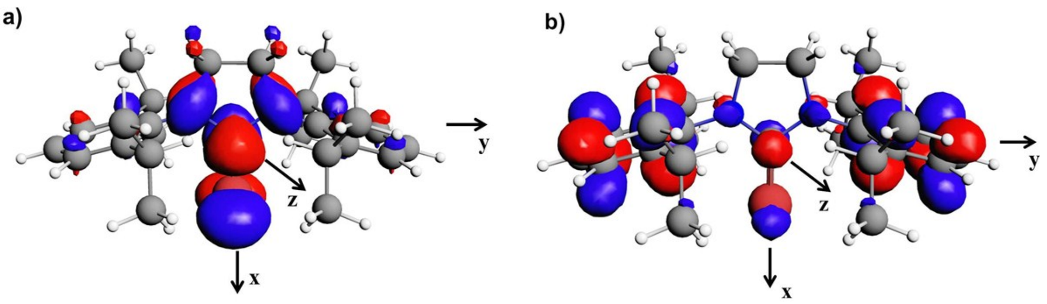

Figure S2. Representation of the virtual orbitals presenting a strong Se-NHC(π*) characters, and participating to the occupied-virtual Se(p_y) → NHC-Se(π*) transitions that contributesto the σ_{xx} component of the paramagnetic tensor.

To better understand the nature of chemical bonding in selenoureas and phosphinidene complexes, we have analyzed the bonding interaction between the NHC and Se atom or PPh fragments using a bonding energy decomposition analysis (BDA), originally developed by Morokuma[22,23] and later modified by Ziegler and Rauk.[24] The BDA is based on the interaction of two fragments rigidly extracted from the equilibrium geometry of the system, and which both have an electronic structure suitable for bond formation. This interaction energy is usually referred to as the bond snapping energy.[25] Although the bond snapping energy does not always correlate with the bond dissociation enthalpy, because the relaxation and the reorganization energy of the two fragments are not considered, the bond snapping energy is closely related to the bond enthalpy, which usually is a good approximation to bond strength values. Further, the bond snapping energy (BSE) can be decomposed into two main terms, namely steric interaction ΔE_0 and orbital interaction ΔE_{int} (Eq. S1).

$$\text{BSE} = -[\Delta E_0 + \Delta E_{\text{int}}] \qquad \text{(S1)}$$

While further elaboration of Eq. S1 is possible, for the present analysis we will limit to the partitioning scheme outlined in Eq. S1. The total orbital interaction energy ΔE_{int} can further be broken down into contributions from the orbital interactions within the various irreproducible representations Γ of the overall symmetry group of the system[26] (Eq. S2).

$$\Delta E_{\text{int}} = \sum_{\Gamma} \Delta E_{\text{int}}^{\Gamma} \qquad \text{(S2)}$$

For ΔE_{int} analysis, the geometries of all the complexes were re-optimized under the constraint of C_S symmetry; the NHC ligand is located in the σ_{xy} mirror plane of the molecule. Therefore, A' contributions to the orbital interaction energy are associated with σ-bonding and A'' contributions represent π-interactions between the NHC and either the Se atom or the PPh fragment. For the latter, the phenyl ring was oriented perpendicular to the σ_{xy} plane.

DFT Results

Table S2. Diamagnetic (σ_d) and paramagnetic (σ_p) components of the DFT calculated Se NMR chemical shielding (ppm) for 23 selenoureas, with the highest values highlighted with * and the lowest with **. The values for [Se(Tr5)] are not included in any subsequent analyses (see the manuscript text).

		δ (ppm)		
**Complex**	**Exp'l ($CDCl_3$)**	**DFT**	**Diamagnetic (σ_d)**	**Paramagnetic (σ_p)**
**[Se(ICy)]**	-22.1	1617.0	3003.6	-1386.6
**[Se(I^{i}Pr^{Me})]**	-18.2	1608.0	3003.5	-1395.5
**[Se(IDD)]**	-11.3	1618.5	3003.9	**-1385.3***
**[Se(IMes)]**	26.7	1535.7	3003.6	-1468.0
**[Se(IMe)]**	29.9	1531.1	3003.6	-1472.5
**[Se(SIDD)]**	75.0	1521.3	3003.5	-1482.1
**[Se(IPr^{OMe})]**	86.5	1443.6	3003.6	-1654.0
**[Se(IPr)]**	90.0	1441.9	3003.8	-1561.9
**[Se(IHept)]**	99.8	1435.9	3003.3	-1567.4
**[Se(IPent)]**	101.2	1427.2	3003.3	-1576.1
**[Se(INon)]**	101.9	1427.5	3003.2	-1575.6
**[Se(IPr*^{OMe})]**	104.0	1439.1	**3005.6***	-1566.4
**[Se(IPr*)]**	106.0	1442.9	3005.4	-1562.5
**[Se(SIMes)]**	109.7	1462.6	3003.3	-1540.7
**[Se(IPr^{Cl})]**	173.6	1347.4	3004.0	**-1656.6****
**[Se(I^{t}Bu)]**	182.6	1354.9	**3002.9****	-1648.0
**[Se(SIPr^{OMe})]**	184.6	1349.5	3003.9	-1560.3
**[Se(SIPr)]**	190.0	1347.2	3003.5	-1656.2
**[Se(IAd)]**	196.9	1360.6	3003.1	-1642.5
**[Se(Tr1)]**	33.5	1498.3	3003.3	-1505.0
**[Se(Tr4)]**	46.9	1508.4	3003.6	-1495.2
**[Se(Tr3)]**	73.6	1473.5	3003.4	-1530.6
**[Se(Tr2)]**	76.1	1441.8	3003.5	-1561.7
**[Se(Tr5)]**	131.2	1304.5	3003.6	-1699.1

Table S3. Calculated Hirshfeld charge (e^{-}) on Se atom and Se(p_y) → Se-NHC(π*) energy gap (in eV) for 23 selenoureas. Calculated DFT NMR chemical shielding and Hirshfeld charge on Se atom with Se-H distances constrained to ≥ 3.5 Å are highlighted in red.

		e^{-}	electron-volts eV		
**Complex**	**DFT**	**Se (HIR)**	**Se(p_y)**	**NHC-Se(π*)**	**Gap**
**[Se(ICy)]**	1617.01	-0.2941	-5.044	-1.070	3.974
**[Se(I^{i}Pr^{Me})]**	1607.98	-0.2853	-4.956	-0.966	3.990
**[Se(IDD)]**	1618.53	-0.2959	-5.065	-1.067	3.998
**[Se(IMes)]**	1535.65	-0.2601	-4.998	-1.182	3.816
**[Se(IMe)]**	1531.08	-0.2563	-5.031	-1.237	3.794
**[Se(SIDD)]**	1521.34	-0.2688	-4.950	-1.118	3.832
**[Se(IPr^{OMe})]**	1443.57	-0.2177	-4.894	-1.238	3.656
**[Se(IPr)]**	1441.90	-0.2237	-4.865	-1.214	3.651
**[Se(IHept)]**	1435.85	-0.1945	-4.774	-1.183	3.591
	1434.86	-0.2277			
**[Se(IPent)]**	1427.24	-0.1810	-4.792	-1.209	3.583
	1427.23	-0.2294			
**[Se(INon)]**	1427.54	-0.1885	-4.748	-1.207	3.541
	1428.36	-0.2279			
**[Se(IPr*^{OMe})]**	1439.13	-0.1849	-5.077	-1.536	3.541
	1438.03	-0.2327			
**[Se(IPr*)]**	1442.92	-0.1880	-5.025	-1.469	3.556
	1443.18	-0.2383			
**[Se(SIMes)]**	1462.61	-0.2345	-4.931	-1.194	3.737
**[Se(IPr^{Cl})]**	1347.41	-0.1926	-5.068	-1.676	3.392
**[Se(I^{t}Bu)]**	1354.94	-0.1985	-4.686	-1.182	3.504
**[Se(SIPr^{OMe})]**	1349.53	-0.1899	-4.816	-1.257	3.559
**[Se(SIPr)]**	1347.24	-0.1947	-4.813	-1.238	3.575
**[Se(IAd)]**	1360.64	-0.2038	-4.588	-1.086	3.502
**[Se(Tr1)]**	1498.30	-0.2756	-5.204	-1.462	3.742
**[Se(Tr4)]**	1508.40	-0.2540	-5.261	-1.634	3.627
**[Se(Tr3)]**	1472.87	-0.2257	-5.371	-1.724	3.647
**[Se(Tr2)]**	1441.79	-0.2610	-5.513	-1.948	3.565

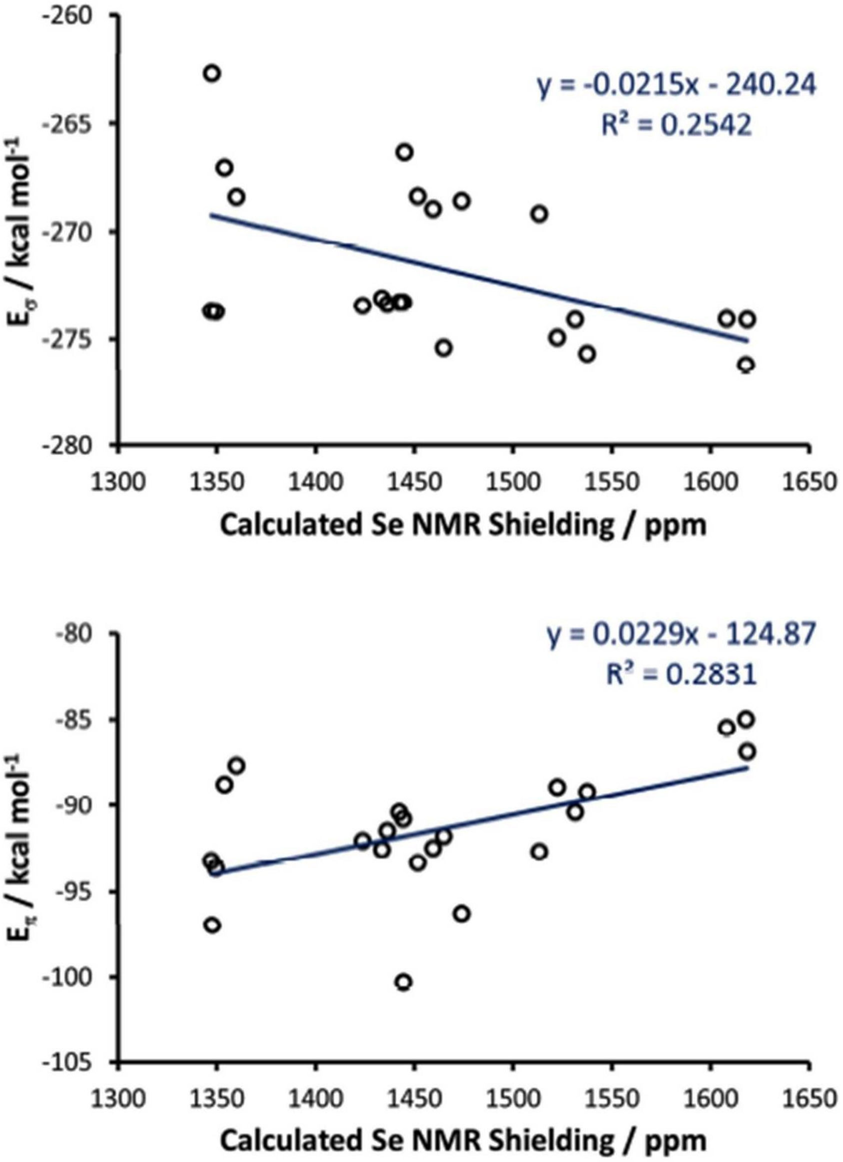

Figure S3. (a) E_σ *versus* the calculated Se shielding; (b) E_π *versus* the calculated Se shielding for 22 selenoureas (excluding [Se(**Tr4**)] and [Se(**Tr5**)].

Table S4. Calculated σ and π orbital interaction energies, E_σ and E_π, respectively, for 22 selenoureas.

**Complex**	**DFT δ (ppm)**	**E (kcal/mol)**							
		**E_σ**	**E_σ-Pred**[a]	**Resid**[b]	**E_π**	**E_π-Pred**[c]	**Residuals**[d]	**E_π-PredF**[f]	**Resid**
**[Se(ICy)]**	1617.8	**-276.2**	-275.9	0.3	**-85.0**	-86.9	-2.0	-86.1	-1.2
**[Se(I^{i}Pr^{Me})]**	1607.8	-274.1	-275.7	-1.6	-85.5	-87.2	-1.7	-86.5	-1.0
**[Se(IDD)]**	1618.3	-274.1	-275.9	-1.8	-86.9	-86.9	0.0	-86.1	0.8
**[Se(IMes)]**	1537.3	-275.7	-274.1	1.6	-89.2	-88.8	0.4	-88.7	0.5
**[Se(IMe)]**	1531.2	-274.1	-274.0	0.1	-90.4	-89.0	1.5	-88.9	1.5
**[Se(SIDD)]**	1521.8	-274.9	-273.8	1.2	-88.9	-89.2	-0.2	-89.2	-0.3
**[Se(IPr^{OMe})]**	1443.9	-273.3	-272.0	1.3	-90.8	-91.0	-0.2	-91.7	-0.9
**[Se(IPr)]**	1441.9	-273.3	-271.9	1.3	-90.4	-91.0	-0.7	-91.8	-1.4
**[Se(IHept)]**	1435.6	-273.4	-271.8	1.6	-91.5	-91.2	0.3	-92.0	-0.5
**[Se(IPent)]**	1432.9	-273.1	-271.7	1.4	-92.5	-91.2	1.3	-92.1	0.5
**[Se(INon)]**	1423.2	-273.5	-271.5	1.9	-92.1	-91.5	0.6	-92.4	-0.3
**[Se(IPr*^{OMe})]**	1451.3	-268.4	-272.2	-3.8	-93.3	-90.8	2.5	-91.5	1.9
**[Se(IPr*)]**	1458.8	-269.0	-272.3	-3.4	-92.5	-90.6	1.9	-91.2	1.2
**[Se(SIMes)]**	1464.4	-275.4	-272.5	3.0	-91.8	-90.5	1.3	-91.1	0.7
**[Se(IPr^{Cl})]**	1347.7	**-262.7**	-269.8	-7.2	**-97.0**	-93.2	3.7	-94.8	2.1
**[Se(SIPr^{OMe})]**	1349.9	-273.7	-269.9	3.9	-93.6	-93.2	0.5	-94.7	-1.1
**[Se(SIPr)]**	1347.6	-273.7	-269.8	3.9	-93.2	-93.2	0.0	-94.8	-1.6
**[Se(I^{t}Bu)]** [e]	1354.2	-267.0	-270.0	-3.0	-88.8	-93.1	-4.3		
**[Se(IAd)]** [e]	1360.3	-268.4	-270.1	-1.7	-87.7	-92.9	-5.3		
**[Se(Tr1)]**	1512.78	-269.2	-269.6	-0.4	-92.7			-92.4	0.3
**[Se(Tr3)]**	1473.47	-268.6	-268.0	0.6	-96.3			-96.8	-0.5
**[Se(Tr2)]**	1444.50	-266.3	-266.8	-0.5	-100.3			-100.0	0.3

[a] E_σ –Pred is the predicted E_σ using the equation of straight line [y = E_σ-Pred = (-0.0226*(x= DFT δ) + -239.36)]. [b] Residuals are calculated as the difference between the E_σ–Pred and E_σ values, i.e., E_i(E_σ–Pred) – E_i(E_σ). [c] E_π –Pred is the predicted E_π using the equation of straight line [y = E_π –Pred = (0.0233*(x= DFT δ) + -124.63)]. [d] Residuals are calculated as the difference between the E_π–Pred and E_π values, i.e., E_i(E_π–Pred) – E_i(E_π). [e] Se(I^{t}Bu) and Se(IAd) are the possible outliers due to large residual errors (shown in red color) thus excluded from the analysis.). [f] E_π –PredF is the final predicted E_π obtained by using the equation of straight line [y = E_π –PredF = (0.0321*(x= DFT δ) + -138.07)]. For triazoles, E_σ –Pred is obtained by using the equation of straight line [y = E_σ –Pred = (-0.0408*(x=Se DFT δ) + -207.85)], while E_π–PredF is obtained by using [y = E_π–Pred = (0.1106*(x= DFT δ) + -259.72)].

Table S5. Diamagnetic (σ_d) and paramagnetic (σ_p) components of the DFT calculated P NMR chemical shielding (ppm) and Hirshfeld charge (e^{-}) on P atom for 11 phosphinidene complexes; "ref." is the corresponding compound number in the original publication.[27] The highest values of σ_d and σ_p are marked with *, and the lowest with **.

Complex	Ref.	Exp'l [a]	DFT	δ (ppm) Diamagnetic (σ_d)	Paramagnetic (σ_p)	*e*– P (HIR)
[PPh(**I*i*Pr**)]	2-PPh	-61.2	430.2	965.3	**-535.1***	-0.0921
[PPh(**ITME**)]	1-PPh	-53.5 [b]	424.0	965.5	-541.5	-0.1074
[PPh(**BI*i*Pr**)]	3-PPh	-34.6	408.2	**966.1***	-557.8	-0.0664
[PPh(**IMes**)]	4-PPh	-23.0	402.1	964.8	-562.7	-0.0528
[PPh(**IPr**)]	5-PPh	-18.9	389.6	964.3	-574.7	-0.0333
[PPh(**SIMes**)]	6-PPh	-10.4	387.3	964.6	-577.3	-0.0231
[PPh(**SIPr**)]	7-PPh	-10.2	383.1	964.5	-581.4	-0.0073
[PPh(**6-IPr**)]	8-PPh	14.8	359.7	**963.7****	-604.0	0.0009
[PPh(**CAC-Mes**)]	10-PPh	39.7	337.1	963.9	-626.8	0.0137
[PPh(**ThIPr**)]	12-PPh	57.0	335.3	964.6	-629.3	0.0148
[PPh(**CAAC-IPr**)]	14-PPh	68.9	321.5	964.3	**-642.8****	0.0175

[a] In benzene-d_6 unless otherwise stated. [b] In THF-d_8. [c] In $CDCl_3$.

Table S6. Calculated σ and π orbital interaction energies, E_σ and E_π, respectively, for 11 phosphinidene complexes.

Complex	δ (ppm)	E (kcal/mol)					
	DFT	E_σ	E_σ–Pred[a]	Resid.[b]	E_π	E_π-Pred[c]	Resid.[d]
[PPh(**I*i*Pr**)]	430.2	-176.9	-173.0	3.8	-29.5	-30.1	-0.6
[PPh(**ITME**)]	424.0	-176.4	-173.3	3.1	-28.8	-31.3	-2.5
[PPh(**BI*i*Pr**)]	408.2	-181.2	-174.2	7.0	-35.1	-34.3	0.8
[PPh(**IMes**)]	402.1	-170.1	-174.5	-4.4	-37.1	-35.5	1.7
[PPh(**IPr**)]	389.6	-169.6	-175.2	-5.6	-38.3	-37.9	0.4
[PPh(**SIMes**)]	387.3	-172.2	-175.3	-3.1	-38.7	-38.3	0.4
[PPh(**SIPr**)]	383.1	-173.2	-175.5	-2.3	-41.9	-39.1	2.8
[PPh(**6-IPr**)]	359.7	-173.2	-176.8	-3.6	-40.9	-43.5	-2.7
[PPh(**CAC-Mes**)]	337.1	-173.2	-178.0	-4.7	-49.8	-47.8	1.9
[PPh(**ThIPr**)]	335.3	-174.9	-178.1	-3.2	-45.8	-48.2	-2.3
[PPh(**CAAC-IPr**)]	321.5	-191.8	-178.8	13.0	-50.9	-50.8	0.1

[a] E_σ–Pred is the predicted E_σ using the equation of straight line [y = E_σ-Pred = (0.053*(x= DFT δ) + -195.82)]. [b] Residuals are calculated as the difference between the E_σ–Pred and E_σ values, i.e.,

Table S7. DFT calculated Se NMR chemical shielding (ppm), Hirshfeld charge (e^{-}) on Se atom, and σ and π orbital interaction energies, E_{σ} and E_{π}, respectively, for 11 Se analogues of phosphinidene complexes considered in the present study; "ref." is the corresponding compound number in the original publication.[1]

Complex	Ref	δ (ppm)		*e*–	E (kcal/mol)	
		Exp'l ($CDCl_3$)	DFT	Se (HIR)	E_{σ}	E_{π}
[Se(**I^{i}Pr**)]			1615.3	-0.2900	-273.5	-87.4
[Se(**ITME**)]			1599.6	-0.3020	-275.7	-86.0
[Se(**BI^{i}Pr**)]	**1**	67	1488.9	-0.2404	-277.1	-93.9
[Se(**IMes**)]		26.7	1535.7	-0.2601	-275.6	-89.2
[Se(**IPr**)]	**2**	90	1441.9	-0.2237	-273.0	-90.3
[Se(**SIMes**)]		109.7	1462.6	-0.2345	-275.4	-91.8
[Se(**SIPr**)]	**3**	190	1347.2	-0.1899	-273.7	-93.2
[Se(**6-IPr**)]	**4**	271	1016.7	-0.1738	-263.6	-90.2
[Se(**CAC-Mes**)]	**5**	472	939.2	-0.1563	-271.9	-99.2
[Se(**ThIPr**)]			1050.1	-0.2157	-267.3	-97.2
[Se(**CAAC-IPr**)]			974.2	-0.1512	-290.6	-99.4

Coordinates

[Se(ICy)] E= -8.84007012 a.u.

C	-2.16693	-0.67808	0.00000
C	-2.16693	0.67808	0.00000
H	-2.99364	-1.37481	0.00000
H	-2.99364	1.37481	0.00000
N	-0.84235	-1.09326	-0.00000
N	-0.84235	1.09326	0.00000
Se	1.87777	0.00000	-0.00000
C	0.00000	0.00000	0.00000
C	-0.42176	2.50209	0.00038
H	0.67903	2.45647	-0.00315
C	-0.42176	-2.50209	0.00035
H	0.67903	-2.45647	-0.00318
C	-0.90200	3.21504	1.27526
H	-2.00133	3.13741	1.34188
H	-0.48954	2.70237	2.15651
C	-0.91026	3.21811	-1.26955
H	-2.01004	3.14131	-1.32917
H	-0.50389	2.70759	-2.15483
C	-0.90200	-3.21506	1.27523
H	-2.00133	-3.13743	1.34184
H	-0.48954	-2.70239	2.15648
C	-0.91026	-3.21809	-1.26959
H	-2.01004	-3.14129	-1.32921
H	-0.50389	-2.70757	-2.15486
C	-0.51265	4.70276	-1.26223
H	-0.90973	5.19552	-2.16205
H	0.58555	4.78532	-1.31871
C	-0.50510	4.69988	1.26911
H	-0.89797	5.19038	2.17188
H	0.59340	4.78238	1.31942
C	-0.50510	-4.69990	1.26905
H	-0.89797	-5.19040	2.17182
H	0.59340	-4.78240	1.31937
C	-0.51265	-4.70274	-1.26229
H	-0.90973	-5.19550	-2.16211
H	0.58555	-4.78530	-1.31876
C	-1.00953	5.41210	0.00545
H	-2.11386	5.42133	0.00888
H	-0.68731	6.46391	0.00630
C	-1.00953	-5.41210	0.00539
H	-0.68731	-6.46391	0.00622
H	-2.11386	-5.42133	0.00882

[Se(L^{i}Pr^{Me})] E = -6.99787038 a.u.

C	-2.17169	-0.68472	0.00000
C	-2.17190	0.68424	0.00000
N	-0.83084	-1.09729	0.00000
N	-0.83111	1.09716	0.00000
Se	1.88821	0.00000	0.00000
C	0.00000	0.00000	0.00000
C	-0.30252	2.47817	0.00000
C	-0.30206	-2.47818	0.00000
C	-0.66442	3.23788	-1.27987
H	-1.73203	3.49038	-1.33286
H	-0.10185	4.18135	-1.30978
H	-0.39569	2.65318	-2.16908
C	-0.66442	3.23788	1.27987
H	-0.10185	4.18135	1.30978
H	-1.73203	3.49038	1.33286
H	-0.39569	2.65318	2.16908
C	-0.66363	-3.23804	-1.27986
H	-0.09662	-4.17877	-1.31237
H	-1.73016	-3.49560	-1.33042
H	-0.39994	-2.65099	-2.16903
C	-0.66363	-3.23804	1.27986
H	-1.73016	-3.49560	1.33042
H	-0.09662	-4.17877	1.31237
H	-0.39994	-2.65099	2.16903
H	0.78581	-2.30943	0.00000
H	0.78532	2.30956	0.00000
C	-3.35243	1.60282	0.00000
H	-3.38078	2.25114	0.88658
H	-3.38078	2.25114	-0.88658
H	-4.27681	1.01431	0.00000
C	-3.35170	-1.60393	0.00000
H	-3.37964	-2.25226	-0.88659
H	-3.37964	-2.25226	0.88659
H	-4.27641	-1.01599	0.00000

[Se(IDD)] E = -15.94463413 a.u

C	-1.233463	-0.672812	0.000000
C	-1.232752	0.681704	0.000000
H	-2.060715	-1.367975	0.000000
H	-2.059264	1.377723	0.000000
C	0.480876	-2.516367	0.000000
C	-0.029137	-3.117787	1.336871
C	-0.029137	-3.117787	-1.336871
H	1.581725	-2.499329	0.000000
C	0.409666	-4.558105	1.709569
H	-1.131096	-3.072768	1.340768
H	0.301640	-2.418780	2.118099
C	0.409666	-4.558105	-1.709569
H	-1.131096	-3.072768	-1.340768
H	0.301640	-2.418780	-2.118099
H	0.923872	-4.539263	2.681827
H	1.158213	-4.927172	0.994494
H	0.923872	-4.539263	-2.681827
H	1.158213	-4.927172	-0.994494
C	0.483016	2.523547	0.000000
C	-0.026841	3.125002	-1.336912
C	-0.026841	3.125002	1.336912
H	1.583845	2.505968	0.000000
C	0.411258	4.565501	-1.709541
H	-1.128769	3.079671	-1.340867
H	0.304141	2.426089	-2.118152
C	0.411258	4.565501	1.709541
H	-1.128769	3.079671	1.340867
H	0.304141	2.426089	2.118152
H	0.925615	4.547026	-2.681731
H	1.159396	4.935055	-0.994303
H	0.925615	4.547026	2.681731
H	1.159396	4.935055	0.994303
C	-0.762812	-5.553419	-1.769518
H	-1.374657	-5.347753	-2.664191
H	-1.427772	-5.360432	-0.914853
C	-0.762812	-5.553419	1.769518
H	-1.374657	-5.347753	2.664191
H	-1.427772	-5.360432	0.914853
C	-0.762058	5.559795	1.769596
H	-1.373574	5.353561	2.664357
H	-1.426947	5.366137	0.915015
C	-0.762058	5.559795	-1.769596
H	-1.373574	5.353561	-2.664357
H	-1.426947	5.366137	-0.915015
C	-1.503483	-8.002191	1.331749
H	-1.638526	-8.757870	2.120167
H	-2.454605	-7.443852	1.296035
C	-1.503483	-8.002191	-1.331749
H	-1.638526	-8.757870	-2.120167
H	-2.454605	-7.443852	-1.296035
C	-1.504986	8.007882	-1.331729
H	-1.640710	8.763419	-2.120148
H	-2.455594	7.448691	-1.295985

C	-1.504986	8.007882	1.331729
H	-1.640710	8.763419	2.120148
H	-2.455594	7.448691	1.295985
C	-1.326217	-8.768985	0.000000
C	-1.328364	8.774846	0.000000
H	-0.338858	-9.262538	0.000000
H	-2.065972	-9.585930	0.000000
H	-0.341380	9.269150	0.000000
H	-2.068745	9.591207	0.000000
N	0.090284	-1.089353	0.000000
N	0.091402	1.096879	0.000000
C	0.934244	0.003346	0.000000
Se	2.810859	0.002298	0.000000
C	-0.358196	7.043624	-1.746996
H	0.027246	7.328725	-2.738022
H	0.490762	7.169442	-1.057887
C	-0.358196	7.043624	1.746996
H	0.027246	7.328725	2.738022
H	0.490762	7.169442	1.057887
C	-0.357566	-7.036895	-1.747040
H	0.027975	-7.321576	-2.738144
H	0.491638	-7.162054	-1.058038
C	-0.357566	-7.036895	1.747040
H	0.027975	-7.321576	2.738144
H	0.491638	-7.162054	1.058038

[Se(IMes)] E = -10.77374787 a.u.

C	-2.18019	-0.67741	0.00000
C	-2.18014	0.67753	0.00000
H	-2.99565	-1.38840	0.00000
H	-2.99557	1.38858	0.00000
N	-0.85056	-1.09227	0.00000
N	-0.85049	1.09232	0.00000
Se	1.86357	0.00000	0.00000
C	0.00000	0.00000	0.00000
C	-0.48558	-2.48706	0.00000
C	-0.36397	-3.15528	1.22796
C	-0.36397	-3.15528	-1.22796
C	-0.13582	-4.53489	1.20076
C	-0.13582	-4.53489	-1.20076
C	-0.03237	-5.24493	0.00000
H	-0.03926	-5.06751	2.14923
H	-0.03926	-5.06751	-2.14923
C	-0.48535	2.48704	0.00000
C	-0.36360	3.15524	-1.22796
C	-0.36360	3.15524	1.22796
C	-0.13519	4.53480	-1.20076
C	-0.13519	4.53480	1.20076
C	-0.03162	5.24483	0.00000
H	-0.03850	5.06741	-2.14923
H	-0.03850	5.06741	2.14923
C	-0.45896	-2.40918	2.53376
H	0.32298	-1.63785	2.59158
H	-1.42759	-1.89987	2.64320
H	-0.33704	-3.09334	3.38136
C	-0.45896	-2.40918	-2.53376
H	-1.42759	-1.89987	-2.64320
H	0.32298	-1.63785	-2.59158
H	-0.33704	-3.09334	-3.38136
C	0.15462	-6.74308	0.00000
H	-0.81869	-7.25789	0.00000
H	0.70194	-7.07961	-0.88930
H	0.70194	-7.07961	0.88930
C	-0.45865	2.40914	-2.53375
H	0.32314	1.63762	-2.59147
H	-1.42737	1.90003	-2.64326
H	-0.33650	3.09325	-3.38136
C	-0.45865	2.40914	2.53375
H	-1.42737	1.90003	2.64326
H	0.32314	1.63762	2.59147
H	-0.33650	3.09325	3.38136
C	0.15567	6.74293	0.00000
H	0.70306	7.07935	-0.88930
H	-0.81753	7.25793	0.00000
H	0.70306	7.07935	0.88930

[Se(IMe)] E = -9.56906621 a.u.

C	-2.18058	-0.67779	0.00000
C	-2.18070	0.67744	0.00000
H	-2.99653	-1.38839	0.00000
H	-2.99676	1.38787	0.00000
N	-0.85075	-1.09225	0.00000
N	-0.85100	1.09215	0.00000
Se	1.86373	0.00000	0.00000
C	0.00000	0.00000	0.00000
C	-0.47756	-2.48551	0.00000
C	-0.34995	-3.14758	1.23216
C	-0.34995	-3.14758	-1.23216
C	-0.09581	-4.52419	1.20777
C	-0.09581	-4.52419	-1.20777
C	0.02476	-5.20893	0.00000
H	0.00585	-5.05931	2.15247
H	0.00585	-5.05931	-2.15247
C	-0.47792	2.48545	0.00000
C	-0.34999	3.14753	-1.23215
C	-0.34999	3.14753	1.23215
C	-0.09543	4.52404	-1.20774
C	-0.09543	4.52404	1.20774
C	0.02552	5.20878	0.00000
H	0.00673	5.05894	-2.15249
H	0.00673	5.05894	2.15249
C	-0.46599	-2.40008	2.53567
H	0.30579	-1.61887	2.59869
H	-1.44221	-1.90360	2.63643
H	-0.34179	-3.08189	3.38485
C	-0.46599	-2.40008	-2.53567
H	-1.44221	-1.90360	-2.63643
H	0.30579	-1.61887	-2.59869
H	-0.34179	-3.08189	-3.38485
C	-0.46604	2.40048	-2.53593
H	0.30409	1.61764	-2.59836
H	-1.44327	1.90628	-2.63807
H	-0.33933	3.08229	-3.38477
C	-0.46604	2.40048	2.53593
H	-1.44327	1.90628	2.63807
H	0.30409	1.61764	2.59836
H	-0.33933	3.08229	3.38477
H	0.21746	6.28113	0.00000
H	0.21582	-6.28135	0.00000

[Se(SIDD)] E = -16.21428109 a.u.

C	0.339866	-2.511583	0.000000
C	-0.141184	-3.152623	1.330974
C	-0.141184	-3.152623	-1.330974
H	1.439927	-2.461792	0.000000
C	0.409903	-4.553265	1.709691
H	-1.242880	-3.205380	1.320496
H	0.123006	-2.431158	2.117751
C	0.409903	-4.553265	-1.709691
H	-1.242880	-3.205380	-1.320496
H	0.123006	-2.431158	-2.117751
H	0.921021	-4.491193	2.681919
H	1.186107	-4.861978	0.995519
H	0.921021	-4.491193	-2.681919
H	1.186107	-4.861978	-0.995519
C	0.343197	2.518737	0.000000
C	-0.137369	3.159880	-1.331089
C	-0.137369	3.159880	1.331089
H	1.443214	2.468000	0.000000
C	0.412842	4.560904	-1.709462
H	-1.239087	3.212071	-1.321140
H	0.127524	2.438628	-2.117830
C	0.412842	4.560904	1.709462
H	-1.239087	3.212071	1.321140
H	0.127524	2.438628	2.117830
H	0.924458	4.499395	-2.681468
H	1.188374	4.870335	-0.994882
H	0.924458	4.499395	2.681468
H	1.188374	4.870335	0.994882
C	-0.676854	-5.641198	-1.770943
H	-1.303189	-5.487447	-2.666134
H	-1.355993	-5.503467	-0.916696
C	-0.676854	-5.641198	1.770943
H	-1.303189	-5.487447	2.666134
H	-1.355993	-5.503467	0.916696
C	-0.675214	5.647487	1.771060
H	-1.301046	5.492867	2.666449
H	-1.354469	5.508786	0.917057
C	-0.675214	5.647487	-1.771060
H	-1.301046	5.492867	-2.666449
H	-1.354469	5.508786	-0.917057
C	-1.210850	-8.143273	1.331597
H	-1.283101	-8.907556	2.120044
H	-2.205109	-7.666039	1.294875
C	-1.210850	-8.143273	-1.331597
H	-1.283101	-8.907556	-2.120044
H	-2.205109	-7.666039	-1.294875
C	-1.213122	8.148512	-1.331608
H	-1.286655	8.912690	-2.120031
H	-2.206573	7.669579	-1.294947
C	-1.213122	8.148512	1.331608
H	-1.286655	8.912690	2.120031
H	-2.206573	7.669579	1.294947
C	-0.969799	-8.892812	0.000000

C	-0.973414	8.898443	0.000000
H	0.055676	-9.301476	0.000000
H	-1.638141	-9.769163	0.000000
H	0.051355	9.308833	0.000000
H	-1.643245	9.773656	0.000000
N	-0.093490	-1.104758	0.000000
N	-0.091812	1.112446	0.000000
C	0.702904	0.003291	0.000000
Se	2.579411	0.002122	0.000000
C	-0.150175	7.092836	-1.747104
H	0.258588	7.345865	-2.737537
H	0.705811	7.146891	-1.057144
C	-0.150175	7.092836	1.747104
H	0.258588	7.345865	2.737537
H	0.705811	7.146891	1.057144
C	-0.149706	-7.085808	-1.747155
H	0.259445	-7.338158	-2.737615
H	0.706409	-7.138560	-1.057264
C	-0.149706	-7.085808	1.747155
H	0.259445	-7.338158	2.737615
H	0.706409	-7.138560	1.057264
C	-1.519410	0.773242	0.000000
H	-2.020445	1.183999	-0.888442
H	-2.020445	1.183999	0.888442
C	-1.520660	-0.763483	0.000000
H	-2.022355	-1.173496	0.888421
H	-2.022355	-1.173496	-0.888421

[Se(IPr^{OMe})] E = -15.98612789 a.u.

N	-0.85281	-1.09242	-0.00000
N	-0.85277	1.09245	0.00000
Se	1.86334	0.00000	-0.00000
C	0.00000	0.00000	0.00000
C	-0.48622	-2.48660	-0.00000
C	-0.36252	-3.15370	1.23387
C	-0.36252	-3.15370	-1.23387
C	-0.12417	-4.53258	1.20889
C	-0.12417	-4.53258	-1.20889
C	-0.01478	-5.21591	-0.00000
H	-0.01055	-5.09174	2.13783
H	-0.01055	-5.09174	-2.13783
C	-0.48613	2.48663	0.00000
C	-0.36247	3.15373	-1.23386
C	-0.36247	3.15373	1.23386
C	-0.12437	4.53265	-1.20889
C	-0.12437	4.53265	1.20889
C	-0.01514	5.21599	0.00000
H	-0.01081	5.09181	-2.13783
H	-0.01081	5.09181	2.13783
C	-0.47616	-2.43080	2.56916
H	-0.66288	-1.36994	2.35906
C	-0.47616	-2.43080	-2.56916
H	-0.66288	-1.36994	-2.35906
C	-0.47584	2.43076	-2.56915
H	-0.66126	1.36969	-2.35903
C	-0.47584	2.43076	2.56915
H	-0.66126	1.36969	2.35903
C	-2.18149	0.67724	0.00000
H	-2.99610	1.38843	0.00000
C	-2.18153	-0.67715	-0.00000
H	-2.99615	-1.38831	-0.00000
O	0.25876	-6.57709	-0.00000
O	0.25820	6.57721	0.00000
C	-0.90982	-7.40751	-0.00000
H	-0.55437	-8.44379	-0.00000
H	-1.52527	-7.23088	0.89742
H	-1.52527	-7.23088	-0.89742
C	-0.91051	7.40743	0.00000
H	-0.55524	8.44379	0.00000
H	-1.52591	7.23070	-0.89742
H	-1.52591	7.23070	0.89742
C	-1.66005	-2.95540	3.40310
H	-2.61035	-2.86908	2.85733
H	-1.52159	-4.01316	3.67062
H	-1.75001	-2.38415	4.33834
C	0.84166	-2.51202	3.36035
H	1.08681	-3.55267	3.61850
H	1.67108	-2.09422	2.77495
H	0.75591	-1.94422	4.29819
C	0.84153	2.51353	3.36089

H	1.67169	2.09663	2.77589
H	1.08541	3.55445	3.61912
H	0.75600	1.94569	4.29871
C	-1.66066	2.95411	3.40257
H	-1.52351	4.01208	3.66996
H	-2.61064	2.86663	2.85645
H	-1.75032	2.38287	4.33785
C	-1.66005	-2.95540	-3.40310
H	-1.75001	-2.38415	-4.33834
H	-1.52159	-4.01316	-3.67062
H	-2.61035	-2.86908	-2.85733
C	0.84166	-2.51202	-3.36035
H	1.67108	-2.09422	-2.77495
H	1.08681	-3.55267	-3.61850
H	0.75591	-1.94422	-4.29819
C	0.84153	2.51353	-3.36089
H	1.08541	3.55445	-3.61912
H	1.67169	2.09663	-2.77589
H	0.75600	1.94569	-4.29871
C	-1.66066	2.95411	-3.40257
H	-2.61064	2.86663	-2.85645
H	-1.52351	4.01208	-3.66996
H	-1.75032	2.38287	-4.33785

[Se(IPr)] E = -14.34404537 a.u.

N	-0.85220	-1.09221	0.00000
N	-0.85228	1.09218	0.00000
Se	1.86382	0.00000	0.00000
C	0.00000	0.00000	0.00000
C	-0.47040	-2.48332	0.00000
C	-0.33203	-3.14466	1.23520
C	-0.33203	-3.14466	-1.23520
C	-0.04329	-4.51506	1.20627
C	-0.04329	-4.51506	-1.20627
C	0.09880	-5.19591	0.00000
H	0.06991	-5.05619	2.14621
H	0.06991	-5.05619	-2.14621
C	-0.47048	2.48327	0.00000
C	-0.33201	3.14460	-1.23520
C	-0.33201	3.14460	1.23520
C	-0.04283	4.51491	-1.20627
C	-0.04283	4.51491	1.20627
C	0.09950	5.19570	0.00000
H	0.07048	5.05603	-2.14622
H	0.07048	5.05603	2.14622
C	-0.49246	-2.42863	2.56950
H	-0.67960	-1.36764	2.36019
C	-0.49246	-2.42863	-2.56950
H	-0.67960	-1.36764	-2.36019
C	-0.49277	2.42861	-2.56948
H	-0.68078	1.36778	-2.36014
C	-0.49277	2.42861	2.56948
H	-0.68078	1.36778	2.36014
C	-2.18083	0.67745	0.00000
H	-2.99614	1.38786	0.00000
C	-2.18077	-0.67760	0.00000
H	-2.99602	-1.38810	0.00000
C	-1.70139	-2.96772	3.35809
H	-2.63320	-2.87976	2.78167
H	-1.56687	-4.02837	3.61619
H	-1.82582	-2.40797	4.29646
C	0.79629	-2.50483	3.40718
H	1.03745	-3.54422	3.67401
H	1.64448	-2.08237	2.85285
H	0.67424	-1.93914	4.34226
C	0.79616	2.50373	3.40699
H	1.64399	2.08091	2.85241
H	1.03799	3.54289	3.67408
H	0.67387	1.93784	4.34192
C	-1.70115	2.96856	3.35831
H	-1.56583	4.02909	3.61640
H	-2.63315	2.88125	2.78208
H	-1.82577	2.40889	4.29670
C	-1.70139	-2.96772	-3.35809
H	-1.82582	-2.40797	-4.29646
H	-1.56687	-4.02837	-3.61619

H	-2.63320	-2.87976	-2.78167
C	0.79629	-2.50483	-3.40718
H	1.64448	-2.08237	-2.85285
H	1.03745	-3.54422	-3.67401
H	0.67424	-1.93914	-4.34226
C	0.79616	2.50373	-3.40699
H	1.03799	3.54289	-3.67408
H	1.64399	2.08091	-2.85241
H	0.67387	1.93784	-4.34192
C	-1.70115	2.96856	-3.35831
H	-2.63315	2.88125	-2.78208
H	-1.56583	4.02909	-3.61640
H	-1.82577	2.40889	-4.29670
H	0.32159	6.26265	0.00000
H	0.32052	-6.26292	0.00000

[Se(IHept)] E = -23.89458199 a.u.

N	-0.85227	-1.09259	0.00000
N	-0.85225	1.09280	0.00000
Se	1.86558	0.00000	0.00000
C	0.00000	0.00000	0.00000
C	-0.48511	-2.48965	0.00000
C	-0.37742	-3.15547	1.23903
C	-0.37742	-3.15547	-1.23903
C	-0.11649	-4.53289	1.20657
C	-0.11649	-4.53289	-1.20657
C	0.02191	-5.21265	0.00000
H	-0.04388	-5.08404	2.14241
H	-0.04388	-5.08404	-2.14241
C	-0.48243	2.48906	0.00000
C	-0.37111	3.15439	-1.23896
C	-0.37111	3.15439	1.23896
C	-0.09672	4.52919	-1.20651
C	-0.09672	4.52919	1.20651
C	0.05049	5.20719	0.00000
H	-0.01944	5.07962	-2.14238
H	-0.01944	5.07962	2.14238
C	-0.58328	-2.43789	2.56625
H	-0.97527	-1.43753	2.33552
C	-0.58328	-2.43789	-2.56625
H	-0.97527	-1.43753	-2.33552
C	-0.58925	2.44034	-2.56614
H	-0.98903	1.44316	-2.33498
C	-0.58925	2.44034	2.56614
H	-0.98903	1.44316	2.33498
C	-2.18067	0.67752	0.00000
H	-2.99583	1.38841	0.00000
C	-2.18065	-0.67716	0.00000
H	-2.99564	-1.38818	0.00000
C	-1.67304	-3.13986	3.41178
H	-2.55191	-3.31027	2.76722
H	-1.33287	-4.14077	3.72166
C	0.74476	-2.18193	3.33157
H	1.42248	-1.64151	2.65115
H	0.52048	-1.48338	4.15487
C	0.73207	2.17351	3.33910
H	1.40894	1.62670	2.66297
H	0.49687	1.47766	4.16163
C	-1.67717	3.15422	3.40422
H	-1.32953	4.15306	3.71247
H	-2.55108	3.33064	2.75452
C	-1.67304	-3.13986	-3.41178
H	-1.33287	-4.14077	-3.72166
H	-2.55191	-3.31027	-2.76722
C	0.74476	-2.18193	-3.33157
H	1.42248	-1.64151	-2.65115
H	0.52048	-1.48338	-4.15487
C	0.73207	2.17351	-3.33910
H	1.40894	1.62670	-2.66297
H	0.49687	1.47766	-4.16163

C	-1.67717	3.15422	-3.40422
H	-2.55108	3.33064	-2.75452
H	-1.32953	4.15306	-3.71247
H	0.25938	6.27680	0.00000
H	0.21872	-6.28455	0.00000
C	-2.11571	-2.35340	-4.65204
H	-2.39670	-1.33100	-4.34894
H	-1.27026	-2.24325	-5.34814
C	-2.11571	-2.35340	4.65204
H	-2.39670	-1.33100	4.34894
H	-1.27026	-2.24325	5.34814
C	-2.13383	2.37642	-4.64485
H	-2.42350	1.35574	-4.34412
H	-1.29321	2.26052	-5.34588
C	-2.13383	2.37642	4.64485
H	-2.42350	1.35574	4.34412
H	-1.29321	2.26052	5.34588
C	1.46814	3.38698	-3.92119
H	0.78737	3.98055	-4.55440
H	1.79526	4.04948	-3.10572
C	1.47333	-3.40164	-3.91010
H	0.79143	-3.98789	-4.54889
H	1.78817	-4.06824	-3.09312
C	1.47333	-3.40164	3.91010
H	0.79143	-3.98789	4.54889
H	1.78817	-4.06824	3.09312
C	1.46814	3.38698	3.92119
H	0.78737	3.98055	4.55440
H	1.79526	4.04948	3.10572
C	-3.29140	-3.01190	-5.38206
H	-4.16872	-3.09946	-4.72401
H	-3.59287	-2.43276	-6.26583
H	-3.03129	-4.02561	-5.72074
C	-3.30668	3.04976	-5.36594
H	-3.61833	2.47796	-6.25092
H	-4.17979	3.14285	-4.70304
H	-3.03826	4.06247	-5.70112
C	-3.29140	-3.01190	5.38206
H	-3.59287	-2.43276	6.26583
H	-4.16872	-3.09946	4.72401
H	-3.03129	-4.02561	5.72074
C	-3.30668	3.04976	5.36594
H	-4.17979	3.14285	4.70304
H	-3.61833	2.47796	6.25092
H	-3.03826	4.06247	5.70112
C	2.70538	-2.99845	4.72966
H	3.22679	-3.87842	5.13149
H	3.42066	-2.43575	4.11282
H	2.42528	-2.35793	5.57951
C	2.69038	2.97284	4.74993
H	3.40469	2.40285	4.13868
H	3.21725	3.84813	5.15482
H	2.39807	2.33565	5.59819
C	2.69038	2.97284	-4.74993
H	3.21725	3.84813	-5.15482
H	3.40469	2.40285	-4.13868
H	2.39807	2.33565	-5.59819
C	2.70538	-2.99845	-4.72966

H	3.42066	-2.43575	-4.11282
H	3.22679	-3.87842	-5.13149
H	2.42528	-2.35793	-5.57951

[Se(IPent)] E = -19.10955266 a.u.

N	-0.85277	-1.09308	0.00000
N	-0.85163	1.09394	0.00000
Se	1.86695	-0.00000	0.00000
C	0.00000	0.00000	0.00000
C	-0.48219	-2.49061	0.00000
C	-0.36795	-3.15673	1.23902
C	-0.36795	-3.15673	-1.23902
C	-0.06987	-4.52679	1.20607
C	-0.06987	-4.52679	-1.20607
C	0.09132	-5.20198	0.00000
H	0.01663	-5.07639	2.14174
H	0.01663	-5.07639	-2.14174
C	-0.47708	2.49015	0.00000
C	-0.35917	3.15536	-1.23901
C	-0.35917	3.15536	1.23901
C	-0.04710	4.52226	-1.20616
C	-0.04710	4.52226	1.20616
C	0.12241	5.19536	0.00000
H	0.04409	5.07120	-2.14178
H	0.04409	5.07120	2.14178
C	-0.59963	-2.44997	2.56849
H	-1.09594	-1.49403	2.34620
C	-0.59963	-2.44997	-2.56849
H	-1.09594	-1.49403	-2.34620
C	-0.60386	2.45101	-2.56756
H	-1.11046	1.50104	-2.34280
C	-0.60386	2.45101	2.56756
H	-1.11046	1.50104	2.34280
C	-2.17978	0.67871	0.00000
H	-2.99500	1.38943	0.00000
C	-2.18053	-0.67621	0.00000
H	-2.99667	-1.38586	0.00000
C	-1.58022	-3.24690	3.46296
H	-2.44802	-3.53640	2.84926
H	-1.11615	-4.19009	3.78798
C	0.72679	-2.06503	3.28270
H	1.32414	-1.47095	2.57419
H	0.47042	-1.38674	4.11172
C	0.71425	2.04996	3.28793
H	1.30833	1.44958	2.58199
H	0.44594	1.37431	4.11541
C	-1.57950	3.25928	3.45738
H	-1.10556	4.19634	3.78591
H	-2.44014	3.56014	2.83907
C	-1.58022	-3.24690	-3.46296
H	-1.11615	-4.19009	-3.78798
H	-2.44802	-3.53640	-2.84926
C	0.72679	-2.06503	-3.28270
H	1.32414	-1.47095	-2.57419
H	0.47042	-1.38674	-4.11172
C	0.71425	2.04996	-3.28793
H	1.30833	1.44958	-2.58199
H	0.44594	1.37431	-4.11541

C	-1.57950	3.25928	-3.45738
H	-2.44014	3.56014	-2.83907
H	-1.10556	4.19634	-3.78591
H	0.36434	6.25802	0.00000
H	0.32062	-6.26737	0.00000
C	-2.06738	-2.47424	-4.69360
H	-2.81032	-3.05931	-5.25340
H	-2.54110	-1.52446	-4.40384
H	-1.24578	-2.23858	-5.38364
C	-2.06738	-2.47424	4.69360
H	-2.54110	-1.52446	4.40384
H	-2.81032	-3.05931	5.25340
H	-1.24578	-2.23858	5.38364
C	-2.08366	2.49168	-4.68436
H	-2.56876	1.54884	-4.39069
H	-2.82169	3.08601	-5.24083
H	-1.26919	2.24392	-5.37864
C	-2.08366	2.49168	4.68436
H	-2.82169	3.08601	5.24083
H	-2.56876	1.54884	4.39069
H	-1.26919	2.24392	5.37864
C	1.57565	3.19428	-3.83195
H	2.46261	2.78852	-4.33864
H	1.03659	3.81411	-4.56459
H	1.93342	3.85114	-3.02712
C	1.57774	-3.21938	-3.82228
H	1.03530	-3.83343	-4.55731
H	2.47159	-2.82375	-4.32493
H	1.92445	-3.87994	-3.01567
C	1.57774	-3.21938	3.82228
H	2.47159	-2.82375	4.32493
H	1.03530	-3.83343	4.55731
H	1.92445	-3.87994	3.01567
C	1.57565	3.19428	3.83195
H	1.03659	3.81411	4.56459
H	2.46261	2.78852	4.33864
H	1.93342	3.85114	3.02712

[Se(INon)] E = -28.68066419 a.u.

N	-0.85128	-1.09426	0.00000
N	-0.85275	1.09335	0.00000
Se	1.86581	0.00000	0.00000
C	0.00000	0.00000	0.00000
C	-0.45780	-2.48444	0.00000
C	-0.31783	-3.14543	1.23838
C	-0.31783	-3.14543	-1.23838
C	0.05804	-4.49573	1.20599
C	0.05804	-4.49573	-1.20599
C	0.26458	-5.15849	0.00000
H	0.17059	-5.04036	2.14172
H	0.17059	-5.04036	-2.14172
C	-0.45972	2.48405	0.00000
C	-0.31983	3.14527	-1.23835
C	-0.31983	3.14527	1.23835
C	0.05406	4.49614	-1.20587
C	0.05406	4.49614	1.20587
C	0.25898	5.15935	0.00000
H	0.16564	5.04112	-2.14145
H	0.16564	5.04112	2.14145
C	-0.62748	-2.46265	2.56493
H	-1.14549	-1.52190	2.33050
C	-0.62748	-2.46265	-2.56493
H	-1.14549	-1.52190	-2.33050
C	-0.62684	2.46277	-2.56560
H	-1.13890	1.51853	-2.33232
C	-0.62684	2.46277	2.56560
H	-1.13890	1.51853	2.33232
C	-2.18054	0.67609	0.00000
H	-2.99741	1.38474	0.00000
C	-2.17946	-0.67934	0.00000
H	-2.99478	-1.38987	0.00000
C	-1.62685	-3.31076	3.38814
H	-2.45121	-3.61195	2.72047
H	-1.15062	-4.24814	3.71503
C	0.64290	-2.03948	3.35129
H	1.26096	-1.42191	2.68016
H	0.31831	-1.37380	4.16798
C	0.64537	2.04924	3.35436
H	1.26878	1.43562	2.68460
H	0.32419	1.38196	4.17108
C	-1.63192	3.30731	3.38555
H	-1.16358	4.25163	3.70371
H	-2.46058	3.59617	2.71769
C	-1.62685	-3.31076	-3.38814
H	-1.15062	-4.24814	-3.71503
H	-2.45121	-3.61195	-2.72047
C	0.64290	-2.03948	-3.35129
H	1.26096	-1.42191	-2.68016
H	0.31831	-1.37380	-4.16798
C	0.64537	2.04924	-3.35436
H	1.26878	1.43562	-2.68460
H	0.32419	1.38196	-4.17108

C	-1.63192	3.30731	-3.38555
H	-2.46058	3.59617	-2.71769
H	-1.16358	4.25163	-3.70371
H	0.55266	6.20887	0.00000
H	0.56009	-6.20740	0.00000
C	-2.22041	-2.60225	-4.61102
H	-2.66317	-1.64047	-4.29841
H	-1.42416	-2.35268	-5.33090
C	-2.22041	-2.60225	4.61102
H	-2.66317	-1.64047	4.29841
H	-1.42416	-2.35268	5.33090
C	-2.21533	2.60309	-4.61557
H	-2.65227	1.63579	-4.31188
H	-1.41416	2.36481	-5.33379
C	-2.21533	2.60309	4.61557
H	-2.65227	1.63579	4.31188
H	-1.41416	2.36481	5.33379
C	1.50117	3.17352	-3.94870
H	0.88612	3.83078	-4.58839
H	1.90166	3.80719	-3.14107
C	1.50779	-3.15701	-3.94517
H	0.89928	-3.81668	-4.58857
H	1.90951	-3.78997	-3.13754
C	1.50779	-3.15701	3.94517
H	0.89928	-3.81668	4.58857
H	1.90951	-3.78997	3.13754
C	1.50117	3.17352	3.94870
H	0.88612	3.83078	4.58839
H	1.90166	3.80719	3.14107
C	-3.29007	-3.44550	-5.31776
H	-4.08386	-3.69801	-4.59524
H	-2.84544	-4.40621	-5.62567
C	-3.28790	3.44380	-5.32092
H	-4.08811	3.68323	-4.60106
H	-2.84934	4.41090	-5.61741
C	-3.29007	-3.44550	5.31776
H	-4.08386	-3.69801	4.59524
H	-2.84544	-4.40621	5.62567
C	-3.28790	3.44380	5.32092
H	-4.08811	3.68323	4.60106
H	-2.84934	4.41090	5.61741
C	2.67843	-2.60714	4.77179
H	3.29274	-1.95437	4.13152
H	2.28276	-1.96239	5.57428
C	2.67275	2.63384	4.78066
H	3.29317	1.98288	4.14444
H	2.27900	1.98935	5.58431
C	2.67275	2.63384	-4.78066
H	3.29317	1.98288	-4.14444
H	2.27900	1.98935	-5.58431
C	2.67843	-2.60714	-4.77179
H	3.29274	-1.95437	-4.13152
H	2.28276	-1.96239	-5.57428
C	-3.89278	2.75787	-6.54966
H	-4.37051	1.80525	-6.27753
H	-4.65498	3.38809	-7.02844
H	-3.12126	2.53717	-7.30193
C	-3.90838	-2.75341	-6.53629

H	-4.39265	-1.80756	-6.25215
H	-3.14359	-2.51892	-7.29124
H	-4.66811	-3.38573	-7.01622
C	-3.89278	2.75787	6.54966
H	-4.65498	3.38809	7.02844
H	-4.37051	1.80525	6.27753
H	-3.12126	2.53717	7.30193
C	-3.90838	-2.75341	6.53629
H	-4.39265	-1.80756	6.25215
H	-4.66811	-3.38573	7.01622
H	-3.14359	-2.51892	7.29124
C	3.55492	-3.70498	5.38186
H	4.38321	-3.28221	5.96729
H	2.97039	-4.35427	6.05110
H	3.99115	-4.34350	4.59963
C	3.54035	3.73961	5.38918
H	2.94981	4.38752	6.05447
H	4.36979	3.32446	5.97840
H	3.97441	4.37813	4.60573
C	3.54035	3.73961	-5.38918
H	4.36979	3.32446	-5.97840
H	2.94981	4.38752	-6.05447
H	3.97441	4.37813	-4.60573
C	3.55492	-3.70498	-5.38186
H	2.97039	-4.35427	-6.05110
H	4.38321	-3.28221	-5.96729
H	3.99115	-4.34350	-4.59963

[Se(IPr^{*OMe})] E = -31.05152019 a.u.

N	-0.84406	-1.09799	0.00000
N	-0.85198	1.09096	0.00000
Se	1.87252	0.00000	0.00000
C	0.00000	0.00000	0.00000
C	-0.42964	-2.48136	0.00000
C	-0.25162	-3.14400	1.22907
C	-0.25162	-3.14400	-1.22907
C	0.16250	-4.47937	1.20835
C	0.16250	-4.47937	-1.20835
C	0.36874	-5.14034	0.00000
H	0.35677	-5.00416	2.14355
H	0.35677	-5.00416	-2.14355
C	-0.48998	2.49115	0.00000
C	-0.36069	3.16921	-1.22957
C	-0.36069	3.16921	1.22957
C	-0.12010	4.54605	-1.20635
C	-0.12010	4.54605	1.20635
C	-0.01022	5.23170	0.00000
H	0.01785	5.09095	-2.13953
H	0.01785	5.09095	2.13953
C	-0.55555	-2.46216	2.56088
H	-0.44276	-1.38201	2.40136
C	-0.55555	-2.46216	-2.56088
H	-0.44276	-1.38201	-2.40136
C	-0.46913	2.45710	-2.57811
H	-0.31859	1.38640	-2.38282
C	-0.46913	2.45710	2.57811
H	-0.31859	1.38640	2.38282
C	-2.18129	0.66397	0.00000
H	-3.00203	1.36684	0.00000
C	-2.17561	-0.68892	0.00000
H	-2.98850	-1.40229	0.00000
O	0.83030	-6.44688	0.00000
O	0.28972	6.58342	0.00000
C	-0.20622	-7.43598	0.00000
H	0.29457	-8.41003	0.00000
H	-0.83997	-7.35046	0.89810
H	-0.83997	-7.35046	-0.89810
C	-0.85403	7.44687	0.00000
H	-0.46687	8.47152	0.00000
H	-1.47279	7.28980	-0.89877
H	-1.47279	7.28980	0.89877
C	-2.00986	-2.68019	2.99312
C	-2.68002	-3.89902	2.81684
C	-2.70115	-1.63053	3.61319
C	-3.99762	-4.06432	3.25326
H	-2.17301	-4.72843	2.32389
C	-4.01843	-1.78846	4.04603
H	-2.20566	-0.66921	3.75201
C	-4.67326	-3.00938	3.86973
H	-4.49882	-5.02074	3.10355
H	-4.53389	-0.94957	4.51314
H	-5.70362	-3.13524	4.20289
C	0.47000	-2.83307	3.63926

C	0.15207	-3.61197	4.75701
C	1.78660	-2.36742	3.49238
C	1.13001	-3.92620	5.70750
H	-0.86816	-3.97104	4.89515
C	2.75976	-2.67725	4.44077
H	2.04418	-1.76113	2.62033
C	2.43541	-3.46053	5.55332
H	0.86308	-4.53310	6.57271
H	3.77582	-2.30497	4.31119
H	3.19605	-3.70154	6.29605
C	-2.00986	-2.68019	-2.99312
C	-2.70115	-1.63053	-3.61319
C	-2.68002	-3.89902	-2.81684
C	-4.01843	-1.78846	-4.04603
H	-2.20566	-0.66921	-3.75201
C	-3.99762	-4.06432	-3.25326
H	-2.17301	-4.72843	-2.32389
C	-4.67326	-3.00938	-3.86973
H	-4.53389	-0.94957	-4.51314
H	-4.49882	-5.02074	-3.10355
H	-5.70362	-3.13524	-4.20289
C	0.47000	-2.83307	-3.63926
C	1.78660	-2.36742	-3.49238
C	0.15207	-3.61197	-4.75701
C	2.75976	-2.67725	-4.44077
H	2.04418	-1.76113	-2.62033
C	1.13001	-3.92620	-5.70750
H	-0.86816	-3.97104	-4.89515
C	2.43541	-3.46053	-5.55332
H	3.77582	-2.30497	-4.31119
H	0.86308	-4.53310	-6.57271
H	3.19605	-3.70154	-6.29605
C	-1.84710	2.58935	3.23804
C	-2.90204	3.31586	2.67390
C	-2.07214	1.94133	4.46488
C	-4.14591	3.39408	3.31115
H	-2.75259	3.83590	1.72738
C	-3.30879	2.01815	5.10369
H	-1.25758	1.38627	4.93195
C	-4.35487	2.74679	4.52809
H	-4.95058	3.96852	2.85189
H	-3.45412	1.51090	6.05716
H	-5.32134	2.81182	5.02791
C	0.67762	2.87948	3.50596
C	1.93296	2.27529	3.35140
C	0.52482	3.87669	4.47880
C	3.00923	2.65732	4.15297
H	2.06636	1.50949	2.58343
C	1.60352	4.26293	5.27907
H	-0.44840	4.34750	4.62444
C	2.84935	3.65336	5.11977
H	3.97701	2.17401	4.02013
H	1.46563	5.03978	6.03106
H	3.68997	3.95032	5.74710
C	-1.84710	2.58935	-3.23804
C	-2.90204	3.31586	-2.67390
C	-2.07214	1.94133	-4.46488
C	-4.14591	3.39408	-3.31115

H	-2.75259	3.83590	-1.72738
C	-3.30879	2.01815	-5.10369
H	-1.25758	1.38627	-4.93195
C	-4.35487	2.74679	-4.52809
H	-4.95058	3.96852	-2.85189
H	-3.45412	1.51090	-6.05716
H	-5.32134	2.81182	-5.02791
C	0.67762	2.87948	-3.50596
C	0.52482	3.87669	-4.47880
C	1.93296	2.27529	-3.35140
C	1.60352	4.26293	-5.27907
H	-0.44840	4.34750	-4.62444
C	3.00923	2.65732	-4.15297
H	2.06636	1.50949	-2.58343
C	2.84935	3.65336	-5.11977
H	1.46563	5.03978	-6.03106
H	3.97701	2.17401	-4.02013
H	3.68997	3.95032	-5.74710

[Se(IPr*)] E = -30.61589582 a.u.

N	-0.84366	-1.09741	0.00000
N	-0.85165	1.09025	0.00000
Se	1.87333	-0.00000	0.00000
C	0.00000	0.00000	0.00000
C	-0.42639	-2.47985	0.00000
C	-0.24510	-3.14353	1.22625
C	-0.24510	-3.14353	-1.22625
C	0.17935	-4.47532	1.19858
C	0.17935	-4.47532	-1.19858
C	0.39960	-5.15844	0.00000
H	0.35983	-4.98309	2.14763
H	0.35983	-4.98309	-2.14763
C	-0.48723	2.48947	0.00000
C	-0.35588	3.16909	-1.22663
C	-0.35588	3.16909	1.22663
C	-0.10790	4.54433	-1.19639
C	-0.10790	4.54433	1.19639
C	0.00779	5.25464	0.00000
H	0.01555	5.07080	-2.14374
H	0.01555	5.07080	2.14374
C	-0.55587	-2.46502	2.55826
H	-0.44389	-1.38427	2.40157
C	-0.55587	-2.46502	-2.55826
H	-0.44389	-1.38427	-2.40157
C	-0.47284	2.46151	-2.57672
H	-0.33427	1.38852	-2.38562
C	-0.47284	2.46151	2.57672
H	-0.33427	1.38852	2.38562
C	-2.18083	0.66412	0.00000
H	-3.00115	1.36765	0.00000
C	-2.17504	-0.68889	0.00000
H	-2.98755	-1.40266	0.00000
C	-2.01131	-2.68520	2.98561
C	-2.68293	-3.90064	2.79258
C	-2.70223	-1.64151	3.61647
C	-4.00191	-4.06859	3.22423
H	-2.17612	-4.72463	2.29004
C	-4.02058	-1.80243	4.04466
H	-2.20565	-0.68242	3.76690
C	-4.67696	-3.02013	3.85216
H	-4.50444	-5.02210	3.06128
H	-4.53634	-0.96846	4.52020
H	-5.70848	-3.14788	4.18105
C	0.46491	-2.83530	3.64134
C	0.14542	-3.62535	4.75076
C	1.77874	-2.35791	3.50815
C	1.11845	-3.93818	5.70675
H	-0.87302	-3.99342	4.87833
C	2.74734	-2.66660	4.46174
H	2.03741	-1.74321	2.64229
C	2.42122	-3.46058	5.56623
H	0.84969	-4.55286	6.56593
H	3.76086	-2.28344	4.34366

H	3.17783	-3.69953	6.31382
C	-2.01131	-2.68520	-2.98561
C	-2.70223	-1.64151	-3.61647
C	-2.68293	-3.90064	-2.79258
C	-4.02058	-1.80243	-4.04466
H	-2.20565	-0.68242	-3.76690
C	-4.00191	-4.06859	-3.22423
H	-2.17612	-4.72463	-2.29004
C	-4.67696	-3.02013	-3.85216
H	-4.53634	-0.96846	-4.52020
H	-4.50444	-5.02210	-3.06128
H	-5.70848	-3.14788	-4.18105
C	0.46491	-2.83530	-3.64134
C	1.77874	-2.35791	-3.50815
C	0.14542	-3.62535	-4.75076
C	2.74734	-2.66660	-4.46174
H	2.03741	-1.74321	-2.64229
C	1.11845	-3.93818	-5.70675
H	-0.87302	-3.99342	-4.87833
C	2.42122	-3.46058	-5.56623
H	3.76086	-2.28344	-4.34366
H	0.84969	-4.55286	-6.56593
H	3.17783	-3.69953	-6.31382
C	-1.84916	2.61036	3.23666
C	-2.88946	3.36445	2.68178
C	-2.08648	1.95258	4.45593
C	-4.13096	3.46017	3.32137
H	-2.72979	3.89277	1.74163
C	-3.32085	2.04645	5.09671
H	-1.28245	1.37677	4.91626
C	-4.35231	2.80287	4.53063
H	-4.92395	4.05646	2.86972
H	-3.47589	1.53119	6.04437
H	-5.31710	2.88113	5.03187
C	0.67711	2.87304	3.50581
C	1.92677	2.25647	3.35348
C	0.53295	3.87146	4.47870
C	3.00590	2.62874	4.15586
H	2.05377	1.48944	2.58556
C	1.61464	4.24839	5.27950
H	-0.43627	4.35066	4.62366
C	2.85502	3.62728	5.12166
H	3.96880	2.13511	4.02498
H	1.48288	5.02602	6.03186
H	3.69778	3.91625	5.74999
C	-1.84916	2.61036	-3.23666
C	-2.88946	3.36445	-2.68178
C	-2.08648	1.95258	-4.45593
C	-4.13096	3.46017	-3.32137
H	-2.72979	3.89277	-1.74163
C	-3.32085	2.04645	-5.09671
H	-1.28245	1.37677	-4.91626
C	-4.35231	2.80287	-4.53063
H	-4.92395	4.05646	-2.86972
H	-3.47589	1.53119	-6.04437
H	-5.31710	2.88113	-5.03187
C	0.67711	2.87304	-3.50581
C	0.53295	3.87146	-4.47870

C	1.92677	2.25647	-3.35348
C	1.61464	4.24839	-5.27950
H	-0.43627	4.35066	-4.62366
C	3.00590	2.62874	-4.15586
H	2.05377	1.48944	-2.58556
C	2.85502	3.62728	-5.12166
H	1.48288	5.02602	-6.03186
H	3.96880	2.13511	-4.02498
H	3.69778	3.91625	-5.74999
C	0.24940	6.74496	0.00000
H	0.80992	7.05742	-0.88964
H	-0.70244	7.29803	0.00000
H	0.80992	7.05742	0.88964
C	0.83625	-6.60353	0.00000
H	-0.03529	-7.27645	0.00000
H	1.43216	-6.83999	-0.89001
H	1.43216	-6.83999	0.89001

[Se(SIMes)] E = -11.04532815 a.u.

N	-0.80253	-1.10576	-0.00000
N	-0.80251	1.10577	0.00000
Se	1.86273	0.00000	-0.00000
C	0.00000	0.00000	-0.00000
C	-0.39967	-2.48227	-0.00000
C	-0.25776	-3.15608	1.22542
C	-0.25776	-3.15608	-1.22542
C	0.01122	-4.52814	1.20014
C	0.01122	-4.52814	-1.20014
C	0.13665	-5.23563	-0.00000
H	0.12774	-5.05698	2.14876
H	0.12774	-5.05698	-2.14876
C	-0.39962	2.48228	0.00000
C	-0.25769	3.15608	-1.22542
C	-0.25769	3.15608	1.22542
C	0.01123	4.52815	-1.20014
C	0.01123	4.52815	1.20014
C	0.13659	5.23564	0.00000
H	0.12772	5.05699	-2.14876
H	0.12772	5.05699	2.14876
C	-0.36171	-2.41434	2.53346
H	0.37855	-1.60229	2.57297
H	-1.35423	-1.96001	2.67214
H	-0.18399	-3.08978	3.37851
C	-0.36171	-2.41434	-2.53346
H	-1.35423	-1.96001	-2.67214
H	0.37855	-1.60229	-2.57297
H	-0.18399	-3.08978	-3.37851
C	0.37512	-6.72654	-0.00000
H	-0.57919	-7.27582	-0.00000
H	0.93431	-7.04370	-0.88917
H	0.93431	-7.04370	0.88917
C	-0.36158	2.41433	-2.53348
H	0.37864	1.60223	-2.57291
H	-1.35411	1.96007	-2.67225
H	-0.18371	3.08975	-3.37852
C	-0.36158	2.41433	2.53348
H	-1.35411	1.96007	2.67225
H	0.37864	1.60223	2.57291
H	-0.18371	3.08975	3.37852
C	0.37496	6.72656	0.00000
H	0.93411	7.04378	-0.88917
H	-0.57940	7.27575	0.00000
H	0.93411	7.04378	0.88917
C	-2.23595	0.77046	0.00000
H	-2.73130	1.19066	-0.88821
H	-2.73130	1.19066	0.88821
C	-2.23597	-0.77041	-0.00000
H	-2.73132	-1.19062	0.88821
H	-2.73132	-1.19062	-0.88821

[Se(IPr^{Cl})] E = -14.21153698 a.u.

N	-0.84541	-1.09985	0.00000
N	-0.84535	1.09991	0.00000
Se	1.85872	0.00000	0.00000
C	0.00000	0.00000	0.00000
C	-0.44233	-2.48630	0.00000
C	-0.28614	-3.14103	1.23637
C	-0.28614	-3.14103	-1.23637
C	0.05118	-4.49989	1.20652
C	0.05118	-4.49989	-1.20652
C	0.21856	-5.17422	0.00000
H	0.18171	-5.03725	2.14622
H	0.18171	-5.03725	-2.14622
C	-0.44210	2.48632	0.00000
C	-0.28573	3.14101	-1.23636
C	-0.28573	3.14101	1.23636
C	0.05167	4.49985	-1.20651
C	0.05167	4.49985	1.20651
C	0.21905	5.17420	0.00000
H	0.18234	5.03717	-2.14622
H	0.18234	5.03717	2.14622
C	-0.46878	-2.43336	2.57217
H	-0.74470	-1.39063	2.36695
C	-0.46878	-2.43336	-2.57217
H	-0.74470	-1.39063	-2.36695
C	-0.46815	2.43329	-2.57218
H	-0.74359	1.39044	-2.36696
C	-0.46815	2.43329	2.57218
H	-0.74359	1.39044	2.36696
C	-2.17919	0.67966	0.00000
C	-2.17921	-0.67955	0.00000
C	-1.60876	-3.06005	3.39740
H	-2.55695	-3.05867	2.84287
H	-1.37790	-4.10047	3.66836
H	-1.75513	-2.49745	4.33072
C	0.84608	-2.40635	3.37279
H	1.17102	-3.42453	3.63241
H	1.64483	-1.92379	2.79448
H	0.70711	-1.84927	4.31062
C	0.84671	2.40688	3.37284
H	1.64573	1.92478	2.79451
H	1.17113	3.42519	3.63259
H	0.70801	1.84963	4.31061
C	-1.60841	3.05953	3.39734
H	-1.37800	4.10009	3.66820
H	-2.55659	3.05771	2.84279
H	-1.75456	2.49695	4.33071
C	-1.60876	-3.06005	-3.39740
H	-1.75513	-2.49745	-4.33072
H	-1.37790	-4.10047	-3.66836
H	-2.55695	-3.05867	-2.84287
C	0.84608	-2.40635	-3.37279
H	1.64483	-1.92379	-2.79448
H	1.17102	-3.42453	-3.63241

H	0.70711	-1.84927	-4.31062
C	0.84671	2.40688	-3.37284
H	1.17113	3.42519	-3.63259
H	1.64573	1.92478	-2.79451
H	0.70801	1.84963	-4.31061
C	-1.60841	3.05953	-3.39734
H	-2.55659	3.05771	-2.84279
H	-1.37800	4.10009	-3.66820
H	-1.75456	2.49695	-4.33071
H	0.47877	6.23258	0.00000
H	0.47819	-6.23262	0.00000
Cl	-3.50562	-1.75266	0.00000
Cl	-3.50562	1.75276	0.00000

[Se(I^{t}Bu)] E = -6.95956736 a.u.

C	-2.15878	-0.67594	0.00000
C	-2.15891	0.67552	0.00000
H	-3.01389	-1.33003	0.00000
H	-3.01404	1.32957	0.00000
N	-0.84399	-1.10997	0.00000
N	-0.84421	1.10980	0.00000
Se	1.89729	0.00000	0.00000
C	0.00000	0.00000	0.00000
C	-0.42617	2.57300	0.00000
C	-0.42558	-2.57305	0.00000
C	0.38869	2.84589	1.27474
H	1.27723	2.20593	1.32291
H	0.70291	3.89913	1.27436
H	-0.23333	2.67184	2.16451
C	0.38869	2.84589	-1.27474
H	0.70291	3.89913	-1.27436
H	1.27723	2.20593	-1.32291
H	-0.23333	2.67184	-2.16451
C	0.38929	-2.84583	1.27477
H	0.70316	-3.89918	1.27466
H	1.27799	-2.20613	1.32279
H	-0.23270	-2.67135	2.16447
C	0.38929	-2.84583	-1.27477
H	1.27799	-2.20613	-1.32279
H	0.70316	-3.89918	-1.27466
H	-0.23270	-2.67135	-2.16447
C	-1.65753	-3.52687	0.00000
H	-2.63278	-3.03758	0.00000
H	-1.63283	-4.17220	-0.88668
H	-1.63283	-4.17220	0.88668
C	-1.65823	3.52667	0.00000
H	-2.63346	3.03729	0.00000
H	-1.63366	4.17204	0.88667
H	-1.63366	4.17204	-0.88667

[Se(SIPr^{OMe})] E = -16.25834739 a.u.

N	-0.80510	-1.10579	0.00000
N	-0.80510	1.10580	0.00000
Se	1.86181	0.00000	0.00000
C	0.00000	0.00000	0.00000
C	-0.40746	-2.48334	0.00000
C	-0.27213	-3.15867	1.23148
C	-0.27213	-3.15867	-1.23148
C	-0.00130	-4.53132	1.20833
C	-0.00130	-4.53132	-1.20833
C	0.12616	-5.21224	0.00000
H	0.12750	-5.08747	2.13738
H	0.12750	-5.08747	-2.13738
C	-0.40740	2.48334	0.00000
C	-0.27195	3.15866	-1.23148
C	-0.27195	3.15866	1.23148
C	-0.00086	4.53124	-1.20833
C	-0.00086	4.53124	1.20833
C	0.12679	5.21214	0.00000
H	0.12810	5.08736	-2.13738
H	0.12810	5.08736	2.13738
C	-0.39654	-2.43953	2.56759
H	-0.63227	-1.38856	2.35383
C	-0.39654	-2.43953	-2.56759
H	-0.63227	-1.38856	-2.35383
C	-0.39654	2.43956	-2.56760
H	-0.63231	1.38859	-2.35384
C	-0.39654	2.43956	2.56760
H	-0.63231	1.38859	2.35384
C	-2.23788	0.76992	0.00000
H	-2.73055	1.19110	-0.88886
H	-2.73055	1.19110	0.88886
C	-2.23788	-0.76990	0.00000
H	-2.73056	-1.19108	0.88886
H	-2.73056	-1.19108	-0.88886
O	0.43702	-6.56609	0.00000
O	0.43802	6.56589	0.00000
C	-0.70799	-7.42794	0.00000
H	-0.32456	-8.45431	0.00000
H	-1.32827	-7.26830	0.89736
H	-1.32827	-7.26830	-0.89736
C	-0.70671	7.42811	0.00000
H	-0.32293	8.45434	0.00000
H	-1.32704	7.26868	-0.89735
H	-1.32704	7.26868	0.89735
C	-1.53608	-3.01538	3.42894
H	-2.49917	-2.99522	2.89876
H	-1.33392	-4.05912	3.71040
H	-1.64344	-2.43636	4.35770
C	0.93836	-2.45543	3.33460
H	1.23502	-3.48357	3.58922
H	1.73595	-2.00275	2.73122
H	0.84537	-1.88935	4.27296
C	0.93824	2.45539	3.33481

H	1.73589	2.00265	2.73156
H	1.23493	3.48351	3.58944
H	0.84507	1.88936	4.27317
C	-1.53618	3.01546	3.42882
H	-1.33401	4.05917	3.71033
H	-2.49920	2.99535	2.89851
H	-1.64369	2.43640	4.35757
C	-1.53608	-3.01538	-3.42894
H	-1.64344	-2.43636	-4.35770
H	-1.33392	-4.05912	-3.71040
H	-2.49917	-2.99522	-2.89876
C	0.93836	-2.45543	-3.33460
H	1.73595	-2.00275	-2.73122
H	1.23502	-3.48357	-3.58922
H	0.84537	-1.88935	-4.27296
C	0.93824	2.45539	-3.33481
H	1.23493	3.48351	-3.58944
H	1.73589	2.00265	-2.73156
H	0.84507	1.88936	-4.27317
C	-1.53618	3.01546	-3.42882
H	-2.49920	2.99535	-2.89851
H	-1.33401	4.05917	-3.71033
H	-1.64369	2.43640	-4.35757

[Se(SIPr)] E = -14.61614435 a.u.

N	-0.80443	-1.10559	0.00000
N	-0.80435	1.10564	0.00000
Se	1.86246	0.00000	0.00000
C	0.00000	0.00000	0.00000
C	-0.39372	-2.48051	0.00000
C	-0.24636	-3.15067	1.23271
C	-0.24636	-3.15067	-1.23271
C	0.06753	-4.51527	1.20564
C	0.06753	-4.51527	-1.20564
C	0.22294	-5.19445	0.00000
H	0.19272	-5.05386	2.14586
H	0.19272	-5.05386	-2.14586
C	-0.39359	2.48055	0.00000
C	-0.24624	3.15071	-1.23272
C	-0.24624	3.15071	1.23272
C	0.06724	4.51539	-1.20564
C	0.06724	4.51539	1.20564
C	0.22236	5.19465	0.00000
H	0.19238	5.05400	-2.14586
H	0.19238	5.05400	2.14586
C	-0.41097	-2.43808	2.56813
H	-0.65316	-1.38850	2.35450
C	-0.41097	-2.43808	-2.56813
H	-0.65316	-1.38850	-2.35450
C	-0.41016	2.43797	-2.56815
H	-0.65215	1.38834	-2.35454
C	-0.41016	2.43797	2.56815
H	-0.65215	1.38834	2.35454
C	-2.23704	0.77057	0.00000
H	-2.73020	1.19104	-0.88888
H	-2.73020	1.19104	0.88888
C	-2.23710	-0.77038	0.00000
H	-2.73029	-1.19082	0.88888
H	-2.73029	-1.19082	-0.88888
C	-1.56670	-3.03234	3.39555
H	-2.51591	-3.01704	2.84082
H	-1.36182	-4.07705	3.67137
H	-1.70379	-2.46312	4.32658
C	0.90231	-2.44246	3.37141
H	1.20229	-3.46789	3.63302
H	1.71189	-1.98078	2.79124
H	0.77821	-1.87969	4.30817
C	0.90352	2.44264	3.37082
H	1.71289	1.98116	2.79020
H	1.20338	3.46816	3.63229
H	0.78003	1.87982	4.30763
C	-1.56568	3.03181	3.39617
H	-1.36105	4.07660	3.67190
H	-2.51517	3.01616	2.84194
H	-1.70208	2.46251	4.32726
C	-1.56670	-3.03234	-3.39555
H	-1.70379	-2.46312	-4.32658
H	-1.36182	-4.07705	-3.67137

H	-2.51591	-3.01704	-2.84082
C	0.90231	-2.44246	-3.37141
H	1.71189	-1.98078	-2.79124
H	1.20229	-3.46789	-3.63302
H	0.77821	-1.87969	-4.30817
C	0.90352	2.44264	-3.37082
H	1.20338	3.46816	-3.63229
H	1.71289	1.98116	-2.79020
H	0.78003	1.87982	-4.30763
C	-1.56568	3.03181	-3.39617
H	-2.51517	3.01616	-2.84194
H	-1.36105	4.07660	-3.67190
H	-1.70208	2.46251	-4.32726
H	0.46485	6.25719	0.00000
H	0.46580	-6.25691	0.00000

[Se(IAd)] E = -12.51560397 a.u.

C	-2.16373	-0.67569	-0.00000
C	-2.16364	0.67606	0.00000
H	-3.00269	-1.35170	-0.00000
H	-3.00240	1.35231	0.00000
N	-0.84448	-1.10425	-0.00000
N	-0.84434	1.10432	0.00000
Se	1.89538	0.00000	-0.00000
C	0.00000	0.00000	0.00000
C	-0.47380	2.56219	0.00000
C	-0.47396	-2.56214	-0.00000
C	0.33294	2.90737	-1.27196
H	1.23372	2.27865	-1.30772
H	-0.27991	2.67017	-2.15806
C	0.33294	2.90737	1.27196
H	1.23372	2.27865	1.30772
H	-0.27991	2.67017	2.15806
C	0.33282	-2.90742	-1.27192
H	1.23380	-2.27895	-1.30742
H	-0.27984	-2.66991	-2.15806
C	0.33282	-2.90742	1.27192
H	1.23380	-2.27895	1.30742
H	-0.27984	-2.66991	2.15806
C	-1.76039	-3.42332	-0.00000
H	-2.36716	-3.19824	0.89218
H	-2.36716	-3.19824	-0.89218
C	-1.76008	3.42358	0.00000
H	-2.36691	3.19864	-0.89218
H	-2.36691	3.19864	0.89218
C	-1.39761	4.92517	0.00000
H	-2.33360	5.50574	0.00000
C	-1.39826	-4.92493	-0.00000
H	-2.33437	-5.50533	-0.00000
C	-0.57617	-5.25848	-1.25982
H	-0.32591	-6.33213	-1.27103
H	-1.17013	-5.05388	-2.16629
C	-0.57617	-5.25848	1.25982
H	-1.17013	-5.05388	2.16629
H	-0.32591	-6.33213	1.27103
C	-0.57541	5.25870	1.25974
H	-0.32476	6.33227	1.27069
H	-1.16937	5.05451	2.16627
C	-0.57541	5.25870	-1.25974
H	-1.16937	5.05451	-2.16627
H	-0.32476	6.33227	-1.27069
C	0.70635	-4.40469	1.26103
H	1.29996	-4.62567	2.16147
C	0.70635	-4.40469	-1.26103
H	1.29996	-4.62567	-2.16147
C	0.70686	4.40453	-1.26102
H	1.30053	4.62542	-2.16145
C	0.70686	4.40453	1.26102
H	1.30053	4.62542	2.16145
C	1.53943	4.71888	0.00000

H	2.46265	4.11863	0.00000
H	1.83630	5.78089	0.00000
C	1.53886	-4.71917	-0.00000
H	2.46211	-4.11899	-0.00000
H	1.83564	-5.78122	-0.00000

[Se(Tr1)] E = -7.83027220 a.u.

C	0.00000	0.00000	0.00000
C	-2.13471	0.68739	-0.02365
C	-0.66355	2.54475	-0.03586
H	0.09466	2.86209	0.68878
H	-0.33413	2.85690	-1.03737
C	-2.10147	3.02956	0.30583
H	-2.35166	3.96185	-0.21150
H	-2.17250	3.21576	1.38493
C	-3.05957	1.86049	-0.07765
H	-3.46330	1.97768	-1.09455
H	-3.91147	1.76295	0.60490
N	-2.24216	-0.61230	-0.01996
N	-0.83087	1.09751	0.00000
N	-0.90836	-1.03914	0.00118
Se	1.85968	0.00000	0.00000
C	-0.64965	-2.45115	0.03407
C	-0.81425	-3.18936	-1.14678
C	-0.32683	-3.05844	1.25896
C	-0.63607	-4.57567	-1.07902
C	-0.15348	-4.44451	1.27057
C	-0.30656	-5.22131	0.11566
H	-0.76210	-5.16486	-1.98964
H	0.09617	-4.93134	2.21588
C	-0.18723	-2.24736	2.51994
H	0.63833	-1.52539	2.42840
H	-1.10410	-1.67691	2.72719
H	0.01208	-2.89817	3.37901
C	-1.18338	-2.51993	-2.44557
H	-2.18747	-2.07569	-2.38804
H	-0.48105	-1.71040	-2.68821
H	-1.17390	-3.24282	-3.26939
C	-0.12101	-6.71886	0.16729
H	0.91419	-6.97995	0.43038
H	-0.77249	-7.17354	0.92690
H	-0.35057	-7.18418	-0.79873

[Se(Tr2)] E = -6.03506221 a.u.

C	0.00000	0.00000	0.00000
C	-2.15619	0.63192	0.02402
C	-0.70723	2.51949	-0.00760
H	-0.13103	2.83675	0.87043
H	-0.15704	2.83028	-0.90447
C	-2.18726	3.02304	0.01303
H	-2.38938	3.64465	-0.86528
H	-2.36454	3.64384	0.89740
C	-3.12300	1.76807	0.02700
H	-3.78030	1.72605	-0.85113
H	-3.76745	1.73527	0.91467
N	-2.24213	-0.66855	0.03390
N	-0.86191	1.07176	0.00000
N	-0.89596	-1.05922	0.01331
Se	1.84832	0.00000	0.00000
C	-0.59640	-2.43599	0.01533
C	-0.85061	-3.21025	1.15312
C	-0.13016	-3.07616	-1.13829
C	-0.64919	-4.58845	1.14444
C	0.09051	-4.45150	-1.15367
C	-0.17485	-5.20689	-0.01171
F	-1.29891	-2.63056	2.27349
F	-0.90422	-5.32135	2.23899
F	0.01908	-6.53189	-0.02814
F	0.53196	-5.05738	-2.26612
F	0.08178	-2.37509	-2.25637

[Se(Tr3)] E = -6.23332783 a.u.

C	-2.12736	0.70267	0.00000
C	0.00000	0.00000	0.00000
N	-0.82824	1.11732	0.00000
N	-2.22392	-0.60192	0.00000
C	-0.64380	-2.43895	0.00000
C	-0.55838	-3.11835	-1.21450
C	-0.55838	-3.11835	1.21450
C	-0.38724	-4.50298	-1.21058
H	-0.63350	-2.56219	-2.14762
C	-0.38724	-4.50298	1.21058
H	-0.63350	-2.56219	2.14762
C	-0.30470	-5.19516	0.00000
H	-0.32541	-5.04146	-2.15532
H	-0.32541	-5.04146	2.15532
H	-0.17898	-6.27749	0.00000
N	-0.90289	-1.02937	0.00000
Se	1.85613	0.00000	0.00000
C	-3.27401	1.65712	0.00000
H	-3.89808	1.46206	-0.88773
H	-3.89808	1.46206	0.88773
C	-0.37953	2.49582	0.00000
H	0.26152	2.64051	0.87985
H	0.26152	2.64051	-0.87985
C	-1.55731	3.50946	0.00000
H	-1.45978	4.15119	-0.88736

H	-1.45978	4.15119	0.88736
O	-2.91512	3.03496	0.00000

[Se(Tr4)] E = -10.87063749 a.u.

H	-0.71963	5.35898	-0.58574
C	0.67306	3.44245	3.60206
C	0.16684	4.74595	3.62403
C	-0.51143	5.26502	2.51603
C	-0.67984	4.45901	1.39197
C	-0.17390	3.15247	1.37610
C	0.51120	2.63279	2.47223
H	1.20023	3.05288	4.47231
H	0.29702	5.36142	4.51394
H	-0.91198	6.27917	2.54008
H	0.92151	1.62392	2.44033
C	-1.37231	4.77722	0.08391
H	-2.31339	5.32969	0.19994
C	-2.13348	0.67206	-0.12079
O	-2.88777	2.95617	0.04884
C	-3.25854	1.63891	-0.33151
H	-4.12635	1.35844	0.27665
H	-3.56087	1.60830	-1.39655
N	-2.20894	-0.62975	-0.15039
N	-0.83938	1.10944	0.00000
N	-0.88499	-1.04546	-0.05959
C	0.00000	0.00000	0.00000
Se	1.86103	0.00000	0.00000
C	-0.62413	-2.45843	-0.08118
C	-0.23846	-3.10554	1.10232
C	-0.86184	-3.16044	-1.27587
C	-0.08219	-4.49604	1.06027
C	-0.69977	-4.54745	-1.25909
C	-0.31649	-5.23546	-0.10185
H	0.21787	-5.01393	1.97338
H	-0.88036	-5.10505	-2.18068
C	-1.28785	-2.45335	-2.53724
H	-2.30591	-2.05085	-2.43789
H	-0.62344	-1.60844	-2.76528
H	-1.27216	-3.14290	-3.38907
C	-0.19232	-6.73997	-0.10832
H	-1.18551	-7.21419	-0.10673
H	0.33377	-7.09419	-1.00477
H	0.34980	-7.10211	0.77330
C	-0.00989	-2.34367	2.38011
H	0.85732	-1.67310	2.27933
H	-0.87852	-1.72035	2.63498
H	0.17425	-3.03161	3.21315
C	-1.63661	3.38980	-0.51216
H	-1.69608	3.37540	-1.61358
C	-0.46158	2.52204	0.02227
H	0.42409	2.61367	-0.62962

[Se(Tr5)] E = -15.89858242 a.u.

Se	0.721096	2.218206	0.090601
Si	2.524830	-3.591721	3.040122
O	1.413095	-2.314526	3.077545
N	-0.408653	0.862282	2.413254
N	-1.820504	2.303270	3.348031
N	-1.080191	2.911891	2.333306
C	2.663443	-0.411817	2.232970
C	1.466413	-0.324198	4.496916
C	1.457588	-5.118286	3.542056
C	1.449041	-0.876259	3.060421
C	-0.241079	2.023963	1.671636
C	-1.388407	1.079284	3.350340
C	-1.341512	4.292573	2.097804
C	-1.139810	-1.238425	3.111134
H	-0.797695	-2.129890	3.640767
H	-1.878913	-1.550080	2.361440
C	0.045542	-0.540117	2.373222
H	0.110939	-0.862265	1.329748
C	-1.771782	-0.175942	4.050595
H	-1.340055	-0.208961	5.060950
H	-2.859789	-0.274310	4.141235
C	0.849308	-4.950016	4.949974
H	1.620431	-4.785489	5.717916
H	0.139554	-4.110931	4.992485
H	0.294264	-5.860844	5.235265
C	2.361159	-6.372370	3.548259
H	1.766575	-7.262802	3.816839
H	2.811151	-6.564903	2.563302
H	3.176888	-6.290341	4.282186
C	0.316754	-5.328656	2.523789
H	-0.356115	-4.459936	2.480418
H	0.701117	-5.506557	1.508693
H	-0.288995	-6.207851	2.805169
C	3.948840	-3.345493	4.257952
H	3.607077	-3.194549	5.291798
H	4.609815	-4.226275	4.244259
H	4.557245	-2.474901	3.973638
C	3.214378	-3.858443	1.302200
H	2.415854	-3.903419	0.547117
H	3.901296	-3.048552	1.017829
H	3.776462	-4.804191	1.255155
C	1.256427	-0.664029	6.900621
C	1.393123	0.706192	7.124975
C	1.573480	1.562766	6.035922
C	1.612841	1.053821	4.736630
C	1.291357	-1.171146	5.598212
C	-2.552313	4.824951	2.565712
C	-2.823257	6.180185	2.395316
C	-1.899108	7.013403	1.760135
C	-0.696058	6.475156	1.300367
C	-0.406592	5.119849	1.465935
C	2.665340	-0.516245	0.834438
C	3.802991	-0.206197	0.089257
C	4.968284	0.219594	0.726071

C	4.986852	0.323787	2.117611
C	3.847147	0.008126	2.860232
H	1.365305	1.104380	8.139316
H	1.687936	2.634378	6.195630
H	1.780164	1.735507	3.901939
H	1.122697	-1.345997	7.740621
H	1.180062	-2.238614	5.432440
H	-2.114700	8.073263	1.628623
H	-3.765646	6.584414	2.764128
H	-3.258299	4.172335	3.070859
H	0.526697	4.698679	1.102391
H	0.036572	7.114964	0.809928
H	1.775821	-0.842999	0.301799
H	3.768231	-0.284241	-0.996607
H	5.853889	0.471414	0.142665
H	5.889153	0.652190	2.632750
H	3.884403	0.090385	3.945558

[PPh(**I*****i*****Pr**)] E = -8.45015545 a.u.

N	1.352996	0.626344	0.000000
N	-0.016647	2.338491	0.000000
C	0.002960	0.945459	0.000000
C	2.123113	1.790063	0.000000
H	3.203813	1.765195	0.000000
C	1.274247	2.844549	0.000000
H	1.481363	3.905883	0.000000
C	-1.240091	3.155361	0.000000
H	-2.062678	2.426342	0.000000
C	1.951087	-0.719773	0.000000
H	1.105130	-1.411395	0.000000
C	2.769184	-0.952507	1.274913
H	3.145122	-1.984334	1.291180
H	3.635997	-0.277240	1.327758
H	2.153130	-0.794779	2.169542
C	-1.333754	3.995719	1.277022
H	-0.523753	4.738627	1.327495
H	-2.288550	4.538463	1.299087
H	-1.277470	3.356908	2.167594
C	2.769184	-0.952507	-1.274913
H	3.635997	-0.277240	-1.327758
H	3.145122	-1.984334	-1.291180
H	2.153130	-0.794779	-2.169542
C	-1.333754	3.995719	-1.277022
H	-2.288550	4.538463	-1.299087
H	-0.523753	4.738627	-1.327495
H	-1.277470	3.356908	-2.167594
P	-1.549688	0.061035	0.000000
C	-1.122155	-1.745927	0.000000
C	-1.071104	-2.472414	1.205195
C	-1.071104	-2.472414	-1.205195
C	-0.937555	-3.863300	1.207379
H	-1.144335	-1.930493	2.148978
C	-0.937555	-3.863300	-1.207379
H	-1.144335	-1.930493	-2.148978
C	-0.866437	-4.563645	0.000000
H	-0.899281	-4.402850	2.154313
H	-0.899281	-4.402850	-2.154313
H	-0.771328	-5.649768	0.000000

[PPh(**ITME**)] E = -7.25692753 a.u.

C	0.056618	-3.060548	0.000000
C	1.224675	-2.361805	0.000000
N	-0.978282	-2.122404	0.000000
N	0.889584	-0.995892	0.000000
C	-0.482239	-0.825353	0.000000
C	1.902258	0.045813	0.000000
H	1.417928	1.022203	0.000000
H	2.536585	-0.034188	0.894084
H	2.536585	-0.034188	-0.894084
C	-2.390122	-2.458055	0.000000
H	-2.653684	-3.036457	-0.896335
H	-2.653684	-3.036457	0.896335
H	-2.969128	-1.528191	0.000000
C	2.640971	-2.827637	0.000000
H	3.192435	-2.477384	-0.886331
H	3.192435	-2.477384	0.886331
H	2.678180	-3.922923	0.000000
C	-0.204822	-4.528181	0.000000
H	0.740758	-5.081945	0.000000
H	-0.775621	-4.844788	0.886613
H	-0.775621	-4.844788	-0.886613
P	-1.629862	0.543817	0.000000
C	-0.591978	2.082835	0.000000
C	-0.280967	2.741307	-1.205026
C	-0.280967	2.741307	1.205026
C	0.345589	3.990243	-1.207343
H	-0.541969	2.260395	-2.148534
C	0.345589	3.990243	1.207343
H	-0.541969	2.260395	2.148534
C	0.664037	4.618076	0.000000
H	0.576328	4.479301	-2.154307
H	0.576328	4.479301	2.154307
H	1.144828	5.596610	0.000000

[PPh(**BI*ⁱ*Pr**)]   E = -10.01818406 a.u.

C	-1.961709	1.318869	0.000000
C	-2.700744	0.120253	0.000000
C	-4.096551	0.132745	0.000000
C	-4.734739	1.380998	0.000000
C	-4.000555	2.571620	0.000000
C	-2.598009	2.559234	0.000000
C	-0.475572	-0.415846	0.000000
H	-4.672845	-0.790026	0.000000
H	-5.822877	1.420208	0.000000
H	-4.522724	3.526952	0.000000
H	-2.032089	3.488285	0.000000
N	-0.611788	0.967792	0.000000
N	-1.781493	-0.916447	0.000000
C	-2.115443	-2.350088	0.000000
H	-1.135467	-2.841933	0.000000
C	0.481528	1.954664	0.000000
H	1.385682	1.346206	0.000000
C	-2.847088	-2.758537	1.305419
H	-3.810606	-3.239657	1.094187
H	-2.240247	-3.464090	1.886787
H	-3.037936	-1.886806	1.943286
C	-2.847088	-2.758537	-1.305419
H	-2.240247	-3.464090	-1.886787
H	-3.810606	-3.239657	-1.094187
H	-3.037936	-1.886806	-1.943286
C	0.482170	2.792989	1.305683
H	1.398489	2.607530	1.880961
H	0.431969	3.869349	1.097022
H	-0.368771	2.533885	1.947499
C	0.482170	2.792989	-1.305683
H	0.431969	3.869349	-1.097022
H	1.398489	2.607530	-1.880961
H	-0.368771	2.533885	-1.947499
P	0.868898	-1.571506	0.000000
C	2.443387	-0.589904	0.000000
C	3.117686	-0.320271	1.206470
C	3.117686	-0.320271	-1.206470
C	4.401296	0.231060	1.207563
H	2.625513	-0.555359	2.150902
C	4.401296	0.231060	-1.207563
H	2.625513	-0.555359	-2.150902
C	5.046629	0.510950	0.000000
H	4.903809	0.431479	2.154227
H	4.903809	0.431479	-2.154227
H	6.052363	0.931686	0.000000

[PPh(**IMes**)] E = -13.43086320 a.u.

N	1.768899	-1.028722	0.000000
N	-0.397068	-1.396258	0.000000
C	0.538327	-0.363872	0.000000
C	3.059661	-0.391964	0.000000
C	3.680616	-0.106085	-1.227700
C	3.680616	-0.106085	1.227700
C	4.954594	0.470326	-1.200590
C	4.954594	0.470326	1.200590
C	5.610407	0.761878	0.000000
H	5.443423	0.703242	-2.149082
H	5.443423	0.703242	2.149082
C	-1.834543	-1.312056	0.000000
C	-2.517395	-1.321369	1.226983
C	-2.517395	-1.321369	-1.226983
C	-3.915638	-1.329060	1.200519
C	-3.915638	-1.329060	-1.200519
C	-4.633207	-1.335706	0.000000
H	-4.457278	-1.322308	2.148963
H	-4.457278	-1.322308	-2.148963
C	1.583686	-2.406871	0.000000
H	2.422753	-3.089475	0.000000
C	0.251415	-2.637428	0.000000
H	-0.310878	-3.561518	0.000000
C	-1.767476	-1.316183	-2.534669
H	-1.063479	-0.473398	-2.582869
H	-1.175209	-2.234201	-2.664079
H	-2.461848	-1.238110	-3.379446
C	2.985325	-0.385099	-2.535219
H	2.726323	-1.448428	-2.641037
H	2.047530	0.187752	-2.595813
H	3.622224	-0.101714	-3.381224
C	-1.767476	-1.316183	2.534669
H	-1.175209	-2.234201	2.664079
H	-1.063479	-0.473398	2.582869
H	-2.461848	-1.238110	3.379446
C	2.985325	-0.385099	2.535219
H	2.047530	0.187752	2.595813
H	2.726323	-1.448428	2.641037
H	3.622224	-0.101714	3.381224
C	-6.142800	-1.368449	0.000000
H	-6.553314	-0.873138	-0.888926
H	-6.514663	-2.404667	0.000000
H	-6.553314	-0.873138	0.888926
C	6.999346	1.354409	0.000000
H	7.764087	0.562418	0.000000
H	7.170409	1.974278	-0.889094
H	7.170409	1.974278	0.889094
P	0.533188	1.408039	0.000000
C	-1.250728	1.923450	0.000000
C	-1.895159	2.264262	1.203619
C	-1.895159	2.264262	-1.203619
C	-3.146117	2.885894	1.206630
H	-1.393312	2.056064	2.149494
C	-3.146117	2.885894	-1.206630

H	-1.393312	2.056064	-2.149494
C	-3.777676	3.197352	0.000000
H	-3.622883	3.140396	2.154005
H	-3.622883	3.140396	-2.154005
H	-4.749011	3.693046	0.000000

[PPh(**IPr**)] E = -16.99752230 a.u.

N	-0.683472	-1.653142	0.000000
N	-1.151728	0.495192	0.000000
C	-0.074665	-0.392297	0.000000
C	0.014631	-2.912614	0.000000
C	0.322610	-3.518895	-1.234990
C	0.322610	-3.518895	1.234990
C	0.934791	-4.778543	-1.206302
C	0.934791	-4.778543	1.206302
C	1.235386	-5.405827	0.000000
H	1.179489	-5.274742	-2.146003
H	1.179489	-5.274742	2.146003
H	1.709578	-6.387156	0.000000
C	-1.166683	1.939873	0.000000
C	-1.244794	2.615531	1.235696
C	-1.244794	2.615531	-1.235696
C	-1.431296	4.003715	1.206367
C	-1.431296	4.003715	-1.206367
C	-1.534928	4.690807	0.000000
H	-1.495847	4.555366	2.143907
H	-1.495847	4.555366	-2.143907
H	-1.685636	5.770163	0.000000
C	-0.000239	-2.859099	2.568977
H	-0.371691	-1.848231	2.357112
C	-0.000239	-2.859099	-2.568977
H	-0.371691	-1.848231	-2.357112
C	-1.160537	1.873938	2.565982
H	-0.636611	0.926093	2.377466
C	-1.160537	1.873938	-2.565982
H	-0.636611	0.926093	-2.377466
C	-2.360099	-0.215816	0.000000
H	-3.309373	0.300688	0.000000
C	-2.067474	-1.534126	0.000000
H	-2.709501	-2.404138	0.000000
C	1.254259	-2.708425	3.447433
H	1.004281	-2.172546	4.374839
H	1.667359	-3.687511	3.731157
H	2.032313	-2.143181	2.917405
C	-1.110670	-3.621426	3.318074
H	-2.028760	-3.692483	2.717961
H	-0.790409	-4.644948	3.563313
H	-1.356781	-3.110880	4.260635
C	-2.562578	1.540214	3.116965
H	-3.133247	2.462703	3.302287
H	-3.138451	0.916717	2.420702
H	-2.478637	0.995208	4.068777
C	-0.355934	2.643316	3.627261
H	0.620653	2.959569	3.240562
H	-0.892803	3.538882	3.972030
H	-0.192390	2.005063	4.507109
C	-2.562578	1.540214	-3.116965
H	-3.138451	0.916717	-2.420702
H	-3.133247	2.462703	-3.302287
H	-2.478637	0.995208	-4.068777
C	-1.110670	-3.621426	-3.318074

H	-0.790409	-4.644948	-3.563313
H	-2.028760	-3.692483	-2.717961
H	-1.356781	-3.110880	-4.260635
C	-0.355934	2.643316	-3.627261
H	-0.892803	3.538882	-3.972030
H	0.620653	2.959569	-3.240562
H	-0.192390	2.005063	-4.507109
C	1.254259	-2.708425	-3.447433
H	2.032313	-2.143181	-2.917405
H	1.667359	-3.687511	-3.731157
H	1.004281	-2.172546	-4.374839
P	1.696059	-0.328462	0.000000
C	2.174253	1.462937	0.000000
C	2.511995	2.108221	1.203431
C	2.511995	2.108221	-1.203431
C	3.126463	3.362792	1.206539
H	2.310477	1.600854	2.147238
C	3.126463	3.362792	-1.206539
H	2.310477	1.600854	-2.147238
C	3.432641	3.996911	0.000000
H	3.380561	3.840718	2.153384
H	3.380561	3.840718	-2.153384
H	3.921817	4.971412	0.000000

[PPh(**SIMes**)] E = -13.70373423 a.u.

N	1.773740	-1.027069	0.000000
N	-0.414589	-1.400909	0.000000
C	0.535741	-0.404859	0.000000
C	3.040938	-0.358189	0.000000
C	3.664439	-0.055102	-1.225050
C	3.664439	-0.055102	1.225050
C	4.921834	0.556301	-1.199965
C	4.921834	0.556301	1.199965
C	5.569557	0.867818	0.000000
H	5.403017	0.805009	-2.148674
H	5.403017	0.805009	2.148674
C	-1.843165	-1.291503	0.000000
C	-2.535102	-1.292164	1.224697
C	-2.535102	-1.292164	-1.224697
C	-3.933286	-1.284841	1.199907
C	-3.933286	-1.284841	-1.199907
C	-4.651817	-1.285012	0.000000
H	-4.475022	-1.267708	2.148451
H	-4.475022	-1.267708	-2.148451
C	-1.789819	-1.286561	-2.535726
H	-1.017386	-0.505630	-2.549485
H	-1.282159	-2.246210	-2.719036
H	-2.477176	-1.112272	-3.372182
C	2.980301	-0.350780	-2.535306
H	2.816570	-1.429374	-2.678285
H	1.996959	0.140622	-2.572050
H	3.582324	0.010256	-3.377701
C	-1.789819	-1.286561	2.535726
H	-1.282159	-2.246210	2.719036
H	-1.017386	-0.505630	2.549485
H	-2.477176	-1.112272	3.372182
C	2.980301	-0.350780	2.535306
H	1.996959	0.140622	2.572050
H	2.816570	-1.429374	2.678285
H	3.582324	0.010256	3.377701
C	-6.161626	-1.298623	0.000000
H	-6.566142	-0.798020	-0.888822
H	-6.547337	-2.329843	0.000000
H	-6.566142	-0.798020	0.888822
C	6.938488	1.505224	0.000000
H	7.729679	0.739586	0.000000
H	7.089200	2.130782	-0.888937
H	7.089200	2.130782	0.888937
P	0.524462	1.360649	0.000000
C	-1.250332	1.907939	0.000000
C	-1.883807	2.268185	1.203672
C	-1.883807	2.268185	-1.203672
C	-3.116297	2.925461	1.206566
H	-1.388871	2.046822	2.150224
C	-3.116297	2.925461	-1.206566
H	-1.388871	2.046822	-2.150224
C	-3.738618	3.255364	0.000000
H	-3.585155	3.193835	2.154056
H	-3.585155	3.193835	-2.154056

H	-4.695174	3.778999	0.000000
C	0.176189	-2.749415	0.000000
H	-0.148884	-3.313158	-0.888296
H	-0.148884	-3.313158	0.888296
C	1.691384	-2.486434	0.000000
H	2.186336	-2.910502	0.888727
H	2.186336	-2.910502	-0.888727

[PPh(**SIPr**)] E = -17.26906860 a.u.

N	-0.667287	-1.674827	0.000000
N	-1.175770	0.488759	0.000000
C	-0.118877	-0.399514	0.000000
C	0.070054	-2.903813	0.000000
C	0.392990	-3.514896	-1.232342
C	0.392990	-3.514896	1.232342
C	1.039186	-4.757168	-1.205557
C	1.039186	-4.757168	1.205557
C	1.358336	-5.376884	0.000000
H	1.298446	-5.246222	-2.145532
H	1.298446	-5.246222	2.145532
H	1.860224	-6.344388	0.000000
C	-1.180940	1.926149	0.000000
C	-1.261392	2.616044	1.232971
C	-1.261392	2.616044	-1.232971
C	-1.393868	4.010258	1.204319
C	-1.393868	4.010258	-1.204319
C	-1.458118	4.704638	0.000000
H	-1.450799	4.561605	2.143215
H	-1.450799	4.561605	-2.143215
H	-1.560642	5.789665	0.000000
C	0.059472	-2.864631	2.568552
H	-0.371928	-1.878417	2.351116
C	0.059472	-2.864631	-2.568552
H	-0.371928	-1.878417	-2.351116
C	-1.256478	1.890460	2.574985
H	-0.907954	0.864978	2.390842
C	-1.256478	1.890460	-2.574985
H	-0.907954	0.864978	-2.390842
C	1.321936	-2.631993	3.418140
H	1.062879	-2.110221	4.351374
H	1.800445	-3.584113	3.690893
H	2.050543	-2.022360	2.867428
C	-0.984744	-3.682682	3.353503
H	-1.902711	-3.840348	2.769126
H	-0.589321	-4.673804	3.621381
H	-1.255398	-3.166991	4.286707
C	-2.674698	1.822261	3.181817
H	-3.042982	2.833780	3.409668
H	-3.400364	1.354591	2.502626
H	-2.664571	1.246718	4.119206
C	-0.302605	2.532316	3.598065
H	0.703684	2.660881	3.183650
H	-0.665443	3.519644	3.918665
H	-0.233720	1.902278	4.496504
C	-2.674698	1.822261	-3.181817
H	-3.400364	1.354591	-2.502626
H	-3.042982	2.833780	-3.409668
H	-2.664571	1.246718	-4.119206
C	-0.984744	-3.682682	-3.353503
H	-0.589321	-4.673804	-3.621381
H	-1.902711	-3.840348	-2.769126
H	-1.255398	-3.166991	-4.286707
C	-0.302605	2.532316	-3.598065

H	-0.665443	3.519644	-3.918665
H	0.703684	2.660881	-3.183650
H	-0.233720	1.902278	-4.496504
C	1.321936	-2.631993	-3.418140
H	2.050543	-2.022360	-2.867428
H	1.800445	-3.584113	-3.690893
H	1.062879	-2.110221	-4.351374
P	1.645571	-0.319756	0.000000
C	2.164332	1.460141	0.000000
C	2.526187	2.093185	1.202493
C	2.526187	2.093185	-1.202493
C	3.170724	3.332310	1.206305
H	2.321155	1.586458	2.145508
C	3.170724	3.332310	-1.206305
H	2.321155	1.586458	-2.145508
C	3.489576	3.960605	0.000000
H	3.437820	3.802483	2.153438
H	3.437820	3.802483	-2.153438
H	4.000131	4.924047	0.000000
C	-2.126990	-1.686407	0.000000
H	-2.517325	-2.207013	0.889144
H	-2.517325	-2.207013	-0.889144
C	-2.483255	-0.193601	0.000000
H	-3.065304	0.093187	-0.887012
H	-3.065304	0.093187	0.887012

[PPh(**6-IPr**)] E = -17.84053750 a.u.

C	0.358154	-0.425389	0.000000
N	1.361125	0.516532	0.000000
N	0.763919	-1.753889	0.000000
C	1.121010	1.943323	0.000000
C	1.098064	2.636332	1.233476
C	1.098064	2.636332	-1.233476
C	0.949913	4.028874	1.204146
C	0.949913	4.028874	-1.204146
C	0.860432	4.720849	0.000000
H	0.913730	4.582232	2.143212
H	0.913730	4.582232	-2.143212
H	0.738664	5.803798	0.000000
C	-0.206781	-2.828424	0.000000
C	-0.611274	-3.388958	-1.232314
C	-0.611274	-3.388958	1.232314
C	-1.443463	-4.514672	-1.204858
C	-1.443463	-4.514672	1.204858
C	-1.858612	-5.076115	0.000000
H	-1.773274	-4.957948	-2.145465
H	-1.773274	-4.957948	2.145465
H	-2.505262	-5.953453	0.000000
C	1.315962	1.937443	2.574316
H	1.249815	0.855107	2.398355
C	-0.182521	-2.804475	2.572324
H	0.407404	-1.902365	2.361477
C	1.315962	1.937443	-2.574316
H	1.249815	0.855107	-2.398355
C	-0.182521	-2.804475	-2.572324
H	0.407404	-1.902365	-2.361477
C	-1.396631	-2.368238	3.412729
H	-2.016948	-3.232004	3.694517
H	-1.062583	-1.880773	4.340788
H	-2.018785	-1.661481	2.847938
C	0.264571	2.299772	3.636719
H	0.447731	1.725398	4.556301
H	0.313032	3.366267	3.900102
H	-0.750960	2.083926	3.290746
C	-1.396631	-2.368238	-3.412729
H	-1.062583	-1.880773	-4.340788
H	-2.016948	-3.232004	-3.694517
H	-2.018785	-1.661481	-2.847938
C	0.264571	2.299772	-3.636719
H	0.313032	3.366267	-3.900102
H	0.447731	1.725398	-4.556301
H	-0.750960	2.083926	-3.290746
C	0.701695	-3.784115	3.368839
H	1.592159	-4.090509	2.800716
H	1.037659	-3.321108	4.308589
H	0.144904	-4.697143	3.626759
C	2.717992	2.257542	3.139369
H	2.897458	1.687265	4.062746
H	3.524497	2.022559	2.431253
H	2.798476	3.327429	3.382799
C	0.701695	-3.784115	-3.368839

H	1.037659	-3.321108	-4.308589
H	1.592159	-4.090509	-2.800716
H	0.144904	-4.697143	-3.626759
C	2.717992	2.257542	-3.139369
H	3.524497	2.022559	-2.431253
H	2.897458	1.687265	-4.062746
H	2.798476	3.327429	-3.382799
C	3.253556	-1.229731	0.000000
H	3.889873	-1.400351	-0.876392
H	3.889873	-1.400351	0.876392
C	2.130548	-2.275138	0.000000
H	2.235999	-2.931937	-0.878609
H	2.235999	-2.931937	0.878609
C	2.810031	0.240347	0.000000
H	3.235819	0.748387	0.876695
H	3.235819	0.748387	-0.876695
P	-1.433426	-0.301037	0.000000
C	-2.072956	1.443463	0.000000
C	-2.518342	2.027991	-1.199968
C	-2.518342	2.027991	1.199968
C	-3.311162	3.177594	-1.205347
H	-2.269548	1.543090	-2.143031
C	-3.311162	3.177594	1.205347
H	-2.269548	1.543090	2.143031
C	-3.704292	3.764357	0.000000
H	-3.638548	3.605371	-2.153770
H	-3.638548	3.605371	2.153770
H	-4.331917	4.656128	0.000000

[PPh(**CAC-Mes**)] E = -15.47208503 a.u.

C	0.270780	0.371293	0.000000
N	0.722913	1.722981	0.000000
N	1.250932	-0.579417	0.000000
C	-0.271608	2.794080	0.000000
C	-0.705790	3.322226	1.225448
C	-0.705790	3.322226	-1.225448
C	-1.639946	4.361238	1.199172
C	-1.639946	4.361238	-1.199172
C	-2.124751	4.892378	0.000000
H	-1.997213	4.766651	2.148378
H	-1.997213	4.766651	-2.148378
C	0.987429	-2.000320	0.000000
C	0.939939	-2.686692	-1.225344
C	0.939939	-2.686692	1.225344
C	0.830880	-4.080571	-1.199693
C	0.830880	-4.080571	1.199693
C	0.781171	-4.797694	0.000000
H	0.774002	-4.619300	-2.148358
H	0.774002	-4.619300	2.148358
C	3.176787	1.134735	0.000000
C	2.704445	-0.333125	0.000000
H	3.120778	-0.850948	-0.879666
H	3.120778	-0.850948	0.879666
C	-0.186691	2.795378	2.537032
H	-0.697237	3.280186	3.377538
H	-0.346699	1.710149	2.616928
H	0.891455	2.988710	2.638616
C	-0.186691	2.795378	-2.537032
H	-0.346699	1.710149	-2.616928
H	-0.697237	3.280186	-3.377538
H	0.891455	2.988710	-2.638616
C	0.991834	-1.945588	-2.537959
H	0.747049	-2.615828	-3.370447
H	0.286157	-1.103870	-2.549600
H	1.992754	-1.530156	-2.733196
C	0.991834	-1.945588	2.537959
H	0.286157	-1.103870	2.549600
H	0.747049	-2.615828	3.370447
H	1.992754	-1.530156	2.733196
C	2.044082	2.160708	0.000000
O	2.309987	3.359801	0.000000
C	4.034693	1.407947	-1.256618
H	4.372140	2.450967	-1.261385
H	4.913546	0.746863	-1.258428
H	3.462740	1.222371	-2.177169
C	4.034693	1.407947	1.256618
H	4.913546	0.746863	1.258428
H	4.372140	2.450967	1.261385
H	3.462740	1.222371	2.177169
C	0.691027	-6.304588	0.000000
H	1.693588	-6.759563	0.000000
H	0.164017	-6.673769	0.888815
H	0.164017	-6.673769	-0.888815
C	-3.112552	6.034379	0.000000

H	-3.755438	6.008660	0.889029
H	-2.591329	7.004224	0.000000
H	-3.755438	6.008660	-0.889029
P	-1.495368	0.276032	0.000000
C	-2.068709	-1.489561	0.000000
C	-2.472350	-2.095129	1.204507
C	-2.472350	-2.095129	-1.204507
C	-3.211970	-3.279627	1.206619
H	-2.222515	-1.616492	2.152315
C	-3.211970	-3.279627	-1.206619
H	-2.222515	-1.616492	-2.152315
C	-3.583480	-3.878185	0.000000
H	-3.512699	-3.727993	2.154226
H	-3.512699	-3.727993	-2.154226
H	-4.173107	-4.795436	0.000000

[PPh(**ThIPr**)] E = -12.75099479 a.u.

C	-1.364621	-1.425489	0.000000
C	-0.535319	-2.489827	0.000000
C	0.695039	-0.273612	0.000000
S	1.151887	-1.973540	0.000000
N	-0.690630	-0.192171	0.000000
P	1.764714	1.109267	0.000000
C	3.384058	0.200908	0.000000
C	4.045213	-0.089113	-1.206034
C	4.045213	-0.089113	1.206034
C	5.319483	-0.661712	-1.207964
H	3.553409	0.144373	-2.151135
C	5.319483	-0.661712	1.207964
H	3.553409	0.144373	2.151135
C	5.960631	-0.948412	0.000000
H	5.815813	-0.877703	-2.154553
H	5.815813	-0.877703	2.154553
H	6.958529	-1.387946	0.000000
C	-1.359373	1.089339	0.000000
C	-1.669270	1.698204	1.235440
C	-1.669270	1.698204	-1.235440
C	-2.326174	2.935450	1.205523
C	-2.326174	2.935450	-1.205523
C	-2.655095	3.549078	0.000000
H	-2.578177	3.427633	2.145501
H	-2.578177	3.427633	-2.145501
H	-3.163912	4.512953	0.000000
C	-1.308423	1.070895	-2.576929
H	-0.812101	0.112031	-2.376803
C	-1.308423	1.070895	2.576929
H	-0.812101	0.112031	2.376803
C	-2.557779	0.786918	-3.432693
H	-3.074384	1.719538	-3.703064
H	-2.271062	0.281534	-4.366049
H	-3.279394	0.146763	-2.906133
C	-0.312236	1.951621	-3.356113
H	-0.019798	1.454499	-4.292627
H	-0.760773	2.921721	-3.617088
H	0.592501	2.136628	-2.761763
C	-2.557779	0.786918	3.432693
H	-2.271062	0.281534	4.366049
H	-3.074384	1.719538	3.703064
H	-3.279394	0.146763	2.906133
C	-0.312236	1.951621	3.356113
H	-0.760773	2.921721	3.617088
H	-0.019798	1.454499	4.292627
H	0.592501	2.136628	2.761763
C	-2.854662	-1.493179	0.000000
H	-3.234652	-0.938486	0.871882
H	-3.234652	-0.938486	-0.871882
C	-3.436144	-2.936001	0.000000
H	-4.096941	-3.025515	-0.871407
H	-4.096941	-3.025515	0.871407
C	-2.469825	-4.164639	0.000000
H	-2.710971	-4.785032	-0.872357

H	-2.710971	-4.785032	0.872357
C	-0.931073	-3.928404	0.000000
H	-0.483376	-4.426183	-0.875164
H	-0.483376	-4.426183	0.875164

[PPh(**CAAC-IPr**)] E = -15.28347601 a.u.

C	-0.262988	0.266808	0.000000
N	-0.654960	-1.050514	0.000000
C	-1.552704	2.060813	-1.265081
C	-1.552704	2.060813	1.265081
C	-2.748158	3.028036	-1.263444
C	-2.748158	3.028036	1.263444
C	-2.760840	3.899931	0.000000
H	-2.705170	3.657498	-2.165162
H	-2.705170	3.657498	2.165162
H	-1.869144	4.549357	0.000000
C	0.295652	-2.143356	0.000000
C	0.761587	-2.671719	1.232828
C	0.761587	-2.671719	-1.232828
C	1.602972	-3.791911	1.200675
C	1.602972	-3.791911	-1.200675
C	2.006495	-4.365047	0.000000
H	1.959044	-4.213489	2.141294
H	1.959044	-4.213489	-2.141294
H	2.655346	-5.240818	0.000000
P	1.451247	0.619146	0.000000
C	1.644744	2.466969	0.000000
C	1.857016	3.164488	1.203668
C	1.857016	3.164488	-1.203668
C	2.218582	4.513811	1.206500
H	1.744222	2.635626	2.150878
C	2.218582	4.513811	-1.206500
H	1.744222	2.635626	-2.150878
C	2.395832	5.195916	0.000000
H	2.371796	5.031169	2.154107
H	2.371796	5.031169	-2.154107
H	2.684530	6.247241	0.000000
C	0.465199	-2.042419	-2.592640
H	-0.230365	-1.209583	-2.432571
C	0.465199	-2.042419	2.592640
H	-0.230365	-1.209583	2.432571
C	1.750308	-1.451877	-3.210968
H	2.473464	-2.246968	-3.446016
H	1.511160	-0.927614	-4.148452
H	2.225234	-0.740396	-2.523151
C	1.750308	-1.451877	3.210968
H	1.511160	-0.927614	4.148452
H	2.473464	-2.246968	3.446016
H	2.225234	-0.740396	2.523151
C	-0.173291	-3.030443	-3.587165
H	0.523012	-3.844012	-3.836226
H	-1.091998	-3.485060	-3.196018
H	-0.419917	-2.513306	-4.526135
C	-0.173291	-3.030443	3.587165
H	-1.091998	-3.485060	3.196018
H	0.523012	-3.844012	3.836226
H	-0.419917	-2.513306	4.526135
C	-2.697619	0.157281	0.000000
H	-3.334816	0.309323	0.881784
H	-3.334816	0.309323	-0.881784

C	-2.139249	-1.282646	0.000000
C	-2.597783	-2.059969	-1.243080
C	-2.597783	-2.059969	1.243080
H	-2.351582	-1.524013	2.167972
H	-2.351582	-1.524013	-2.167972
H	-3.688966	-2.189748	1.203661
H	-3.688966	-2.189748	-1.203661
H	-2.140311	-3.057693	1.276774
H	-2.140311	-3.057693	-1.276774
C	-1.508460	1.161372	0.000000
H	-1.574402	1.420105	2.161423
H	-0.625011	2.646590	1.311537
H	-0.625011	2.646590	-1.311537
H	-1.574402	1.420105	-2.161423
H	-3.694951	2.464257	1.325109
H	-3.694951	2.464257	-1.325109
H	-3.638792	4.564017	0.000000

[Se(**I^{i}Pr**)] E = -5.79810801 a.u.

N	1.321002	0.704569	0.000000
N	-0.009784	2.437564	0.000000
C	-0.011902	1.058517	0.000000
C	2.119871	1.839763	0.000000
H	3.200407	1.790794	0.000000
C	1.293290	2.916265	0.000000
H	1.525454	3.972685	0.000000
C	-1.213472	3.286539	0.000000
H	-2.040682	2.561775	0.000000
C	1.829850	-0.677746	0.000000
H	0.915711	-1.289299	0.000000
C	2.632827	-0.954908	1.274273
H	2.945999	-2.007393	1.295080
H	3.541407	-0.335476	1.319609
H	2.030223	-0.755064	2.169368
C	-1.273273	4.133885	1.274379
H	-0.439638	4.851008	1.319604
H	-2.208793	4.708807	1.295481
H	-1.236006	3.499949	2.169402
C	2.632827	-0.954908	-1.274273
H	3.541407	-0.335476	-1.319609
H	2.945999	-2.007393	-1.295080
H	2.030223	-0.755064	-2.169368
C	-1.273273	4.133885	-1.274379
H	-2.208793	4.708807	-1.295481
H	-0.439638	4.851008	-1.319604
H	-1.236006	3.499949	-2.169402
Se	-1.501504	-0.086122	0.000000

[Se(**ITMe**)] E = -4.60481191 a.u.

C	0.069331	-3.134804	0.000000
C	1.212321	-2.386469	0.000000
N	-1.002890	-2.235473	0.000000
N	0.817149	-1.044000	0.000000
C	-0.553469	-0.936247	0.000000
C	1.726544	0.088795	0.000000
H	1.110243	0.995726	0.000000
H	2.361789	0.072950	0.895713
H	2.361789	0.072950	-0.895713
C	-2.404911	-2.615740	0.000000
H	-2.644642	-3.204299	-0.895685
H	-2.644642	-3.204299	0.895685
H	-2.989545	-1.688101	0.000000
C	2.646072	-2.800245	0.000000
H	3.182249	-2.429454	-0.886743
H	3.182249	-2.429454	0.886743
H	2.725524	-3.893488	0.000000
C	-0.125199	-4.614402	0.000000
H	0.844933	-5.124637	0.000000
H	-0.679573	-4.957369	0.886747

H	-0.679573	-4.957369	-0.886747
Se	-1.582091	0.635300	0.000000

[Se(**BI^{i}Pr**)] E = -7.38050106 a.u.

C	-2.015824	1.305691	0.000000
C	-2.782162	0.120247	0.000000
C	-4.179461	0.169316	0.000000
C	-4.790929	1.425628	0.000000
C	-4.030407	2.601966	0.000000
C	-2.633840	2.559804	0.000000
C	-0.584499	-0.459915	0.000000
H	-4.782892	-0.734279	0.000000
H	-5.878050	1.487531	0.000000
H	-4.532948	3.567969	0.000000
H	-2.057263	3.480767	0.000000
N	-0.676300	0.918879	0.000000
N	-1.879454	-0.942368	0.000000
C	-2.211183	-2.380723	0.000000
H	-1.222387	-2.861963	0.000000
C	0.499027	1.811985	0.000000
H	1.343528	1.107613	0.000000
C	-2.947377	-2.782051	1.281884
H	-3.934540	-2.307914	1.362611
H	-3.095024	-3.870442	1.290415
H	-2.359944	-2.508408	2.167764
C	-2.947377	-2.782051	-1.281884
H	-3.095024	-3.870442	-1.290415
H	-3.934540	-2.307914	-1.362611
H	-2.359944	-2.508408	-2.167764
C	0.562714	2.647903	1.282052
H	1.496157	3.226804	1.292422
H	-0.272883	3.356022	1.360991
H	0.550856	2.000376	2.168175
C	0.562714	2.647903	-1.282052
H	-0.272883	3.356022	-1.360991
H	1.496157	3.226804	-1.292422
H	0.550856	2.000376	-2.168175
Se	0.986364	-1.475477	0.000000

[Se(**6-IPr**)] E = -15.19778324 a.u.

C	0.469945	-0.488068	0.000000
N	1.463658	0.443965	0.000000
N	0.823769	-1.803833	0.000000
C	1.180863	1.865713	0.000000
C	1.131577	2.550303	1.232314
C	1.131577	2.550303	-1.232314
C	0.995559	3.943485	1.204845
C	0.995559	3.943485	-1.204845
C	0.926846	4.638500	0.000000
H	0.942714	4.492918	2.145712
H	0.942714	4.492918	-2.145712
H	0.822799	5.723321	0.000000
C	-0.165871	-2.863123	0.000000
C	-0.568862	-3.418735	-1.232255
C	-0.568862	-3.418735	1.232255
C	-1.421161	-4.529176	-1.204784
C	-1.421161	-4.529176	1.204784
C	-1.847443	-5.082439	0.000000
H	-1.756130	-4.967906	-2.145646
H	-1.756130	-4.967906	2.145646
H	-2.509455	-5.948109	0.000000
C	1.202687	1.826849	2.571020
H	1.302670	0.753137	2.362850
C	-0.125068	-2.843283	2.571042
H	0.526766	-1.984218	2.363006
C	1.202687	1.826849	-2.571020
H	1.302670	0.753137	-2.362850
C	-0.125068	-2.843283	-2.571042
H	0.526766	-1.984218	-2.363006
C	-1.324380	-2.314000	3.378581
H	-2.011388	-3.130425	3.645899
H	-0.978958	-1.846939	4.312602
H	-1.880006	-1.567146	2.796866
C	-0.096565	2.010591	3.376454
H	-0.051407	1.431201	4.310303
H	-0.249050	3.066613	3.643845
H	-0.961889	1.669966	2.793206
C	-1.324380	-2.314000	-3.378581
H	-0.978958	-1.846939	-4.312602
H	-2.011388	-3.130425	-3.645899
H	-1.880006	-1.567146	-2.796866
C	-0.096565	2.010591	-3.376454
H	-0.249050	3.066613	-3.643845
H	-0.051407	1.431201	-4.310303
H	-0.961889	1.669966	-2.793206
C	0.682406	-3.864037	3.396037
H	1.556639	-4.238816	2.844011
H	1.037967	-3.406411	4.330901
H	0.065072	-4.733523	3.664827
C	2.425935	2.266651	3.398491
H	2.484611	1.689531	4.333007
H	3.367258	2.123795	2.848059
H	2.360240	3.330766	3.668069
C	0.682406	-3.864037	-3.396037

H	1.037967	-3.406411	-4.330901
H	1.556639	-4.238816	-2.844011
H	0.065072	-4.733523	-3.664827
C	2.425935	2.266651	-3.398491
H	3.367258	2.123795	-2.848059
H	2.484611	1.689531	-4.333007
H	2.360240	3.330766	-3.668069
C	3.332514	-1.302911	0.000000
H	3.966383	-1.483202	-0.876263
H	3.966383	-1.483202	0.876263
C	2.195746	-2.338868	0.000000
H	2.286975	-2.995087	-0.878667
H	2.286975	-2.995087	0.878667
C	2.911540	0.176357	0.000000
H	3.334715	0.686208	0.878652
H	3.334715	0.686208	-0.878652
Se	-1.346074	0.028731	0.000000

[Se(**CAC-Mes**)] E = -12.81313863 a.u.

C	0.310520	0.426432	0.000000
N	0.723267	1.768843	0.000000
N	1.279196	-0.516623	0.000000
C	-0.274830	2.836588	0.000000
C	-0.696294	3.370191	1.225322
C	-0.696294	3.370191	-1.225322
C	-1.587366	4.446575	1.199209
C	-1.587366	4.446575	-1.199209
C	-2.042429	5.003392	0.000000
H	-1.929460	4.864295	2.148658
H	-1.929460	4.864295	-2.148658
C	0.971392	-1.933811	0.000000
C	0.908096	-2.616032	-1.226444
C	0.908096	-2.616032	1.226444
C	0.775399	-4.007482	-1.199649
C	0.775399	-4.007482	1.199649
C	0.717851	-4.724604	0.000000
H	0.714313	-4.544525	-2.148810
H	0.714313	-4.544525	2.148810
C	3.198358	1.192559	0.000000
C	2.734807	-0.278782	0.000000
H	3.145518	-0.799670	-0.879658
H	3.145518	-0.799670	0.879658
C	-0.213014	2.807981	2.536606
H	-0.684281	3.329528	3.377904
H	-0.453655	1.738034	2.618220
H	0.876526	2.919557	2.641124
C	-0.213014	2.807981	-2.536606
H	-0.453655	1.738034	-2.618220
H	-0.684281	3.329528	-3.377904
H	0.876526	2.919557	-2.641124
C	0.930841	-1.871195	-2.536100
H	0.878646	-2.568420	-3.380306
H	0.073202	-1.184957	-2.597471
H	1.841848	-1.266605	-2.657871
C	0.930841	-1.871195	2.536100
H	0.073202	-1.184957	2.597471
H	0.878646	-2.568420	3.380306
H	1.841848	-1.266605	2.657871
C	2.058173	2.215744	0.000000
O	2.314066	3.411126	0.000000
C	4.055619	1.471771	-1.256201
H	4.391564	2.515239	-1.258984
H	4.936317	0.813294	-1.256271
H	3.487412	1.285488	-2.178716
C	4.055619	1.471771	1.256201
H	4.936317	0.813294	1.256271
H	4.391564	2.515239	1.258984
H	3.487412	1.285488	2.178716
C	0.626109	-6.231555	0.000000
H	1.629209	-6.685657	0.000000
H	0.100173	-6.601511	0.889064
H	0.100173	-6.601511	-0.889064
C	-2.964572	6.198939	0.000000

H	-3.607565	6.210202	0.889183
H	-2.388400	7.137282	0.000000
H	-3.607565	6.210202	-0.889183
Se	-1.499768	-0.020899	0.000000

[Se(**ThIPr**)]   E = -10.07873139 a.u.

C	-1.374935	-1.406930	0.000000
C	-0.549329	-2.478076	0.000000
C	0.682267	-0.253346	0.000000
S	1.125759	-1.945451	0.000000
N	-0.692174	-0.174572	0.000000
Se	1.923742	1.124822	0.000000
C	-1.395526	1.092784	0.000000
C	-1.735284	1.676646	1.236249
C	-1.735284	1.676646	-1.236249
C	-2.417550	2.899713	1.206259
C	-2.417550	2.899713	-1.206259
C	-2.751961	3.509443	0.000000
H	-2.686996	3.382679	2.145954
H	-2.686996	3.382679	-2.145954
H	-3.278242	4.463727	0.000000
C	-1.379875	1.038627	-2.574085
H	-0.935595	0.054587	-2.372533
C	-1.379875	1.038627	2.574085
H	-0.935595	0.054587	2.372533
C	-2.624089	0.814594	-3.454465
H	-3.090170	1.768671	-3.739540
H	-2.342905	0.293303	-4.380566
H	-3.387239	0.211632	-2.942106
C	-0.323957	1.870712	-3.326542
H	-0.038884	1.365069	-4.260602
H	-0.719677	2.863868	-3.586365
H	0.576074	2.005418	-2.712543
C	-2.624089	0.814594	3.454465
H	-2.342905	0.293303	4.380566
H	-3.090170	1.768671	3.739540
H	-3.387239	0.211632	2.942106
C	-0.323957	1.870712	3.326542
H	-0.719677	2.863868	3.586365
H	-0.038884	1.365069	4.260602
H	0.576074	2.005418	2.712543
C	-2.866991	-1.465260	0.000000
H	-3.241879	-0.907514	0.871664
H	-3.241879	-0.907514	-0.871664
C	-3.461223	-2.902779	0.000000
H	-4.122938	-2.986574	-0.871292
H	-4.122938	-2.986574	0.871292
C	-2.504768	-4.138724	0.000000
H	-2.750822	-4.757381	-0.872156
H	-2.750822	-4.757381	0.872156
C	-0.963393	-3.914416	0.000000
H	-0.521107	-4.415501	-0.875061
H	-0.521107	-4.415501	0.875061

[Se(**CAAC-IPr**)] E = -12.61945353 a.u.

C	-0.275684	0.203593	0.000000
N	-0.645567	-1.092513	0.000000
C	-1.515635	2.013690	-1.259528
C	-1.515635	2.013690	1.259528
C	-2.694301	2.999352	-1.264784
C	-2.694301	2.999352	1.264784
C	-2.706793	3.869595	0.000000
H	-2.637519	3.629480	-2.165119
H	-2.637519	3.629480	2.165119
H	-1.815016	4.518923	0.000000
C	0.288250	-2.205604	0.000000
C	0.724938	-2.746616	1.233620
C	0.724938	-2.746616	-1.233620
C	1.527437	-3.894863	1.201453
C	1.527437	-3.894863	-1.201453
C	1.911112	-4.480586	0.000000
H	1.869802	-4.327536	2.141881
H	1.869802	-4.327536	-2.141881
H	2.529808	-5.377814	0.000000
Se	1.469169	0.836986	0.000000
C	0.450286	-2.096405	-2.587364
H	-0.249935	-1.267369	-2.429585
C	0.450286	-2.096405	2.587364
H	-0.249935	-1.267369	2.429585
C	1.745643	-1.487168	-3.162667
H	2.484199	-2.273387	-3.378567
H	1.529058	-0.958853	-4.103010
H	2.189109	-0.775074	-2.455014
C	1.745643	-1.487168	3.162667
H	1.529058	-0.958853	4.103010
H	2.484199	-2.273387	3.378567
H	2.189109	-0.775074	2.455014
C	-0.170951	-3.066210	-3.609539
H	0.529309	-3.873457	-3.866965
H	-1.093432	-3.531111	-3.238846
H	-0.407454	-2.531182	-4.540724
C	-0.170951	-3.066210	3.609539
H	-1.093432	-3.531111	3.238846
H	0.529309	-3.873457	3.866965
H	-0.407454	-2.531182	4.540724
C	-2.704378	0.114075	0.000000
H	-3.341739	0.265844	0.881960
H	-3.341739	0.265844	-0.881960
C	-2.144519	-1.328498	0.000000
C	-2.595454	-2.106943	-1.242951
C	-2.595454	-2.106943	1.242951
H	-2.356079	-1.571067	2.169519
H	-2.356079	-1.571067	-2.169519
H	-3.686441	-2.236831	1.198386
H	-3.686441	-2.236831	-1.198386
H	-2.138079	-3.104463	1.277891
H	-2.138079	-3.104463	-1.277891
C	-1.510055	1.106383	0.000000
H	-1.535895	1.383546	2.163409
H	-0.561979	2.563820	1.275719

H	-0.561979	2.563820	-1.275719
H	-1.535895	1.383546	-2.163409
H	-3.648427	2.448363	1.335996
H	-3.648427	2.448363	-1.335996
H	-3.584209	4.534278	0.000000

References.

(1) Liske, A.; Verlinden, K.; Buhl, H.; Schaper, K.; Ganter, C. *Organometallics* **2013**, *32*, 5269.

(2) Nelson, D. J.; Nahra, F.; Patrick, S. R.; Cordes, D. B.; Slawin, A. M. Z.; Nolan, S. P. *Organometallics* **2014**, *33*, 3640.

(3) Nelson, D. J.; Collado, A.; Manzini, S.; Meiries, S.; Slawin, A. M. Z.; Cordes, D. B.; Nolan, S. P. *Organometallics* **2014**, *33*, 2048.

(4) Arduengo, A. J.; Harlow, R. L.; Kline, M. *J. Am. Chem. Soc.* **1991**, *113*, 361.

(5) Fortman, G. C.; Slawin, A. M. Z.; Nolan, S. P. *Dalton Trans.* **2010**, *39*, 3923.

(6) Meiries, S.; Le Duc, G.; Chartoire, A.; Collado, A.; Speck, K.; Athukorala Arachige, K. S.; Slawin, A. M. Z.; Nolan, S. P. *Chem. Eur. J.* **2013**, *19*, 17358.

(7) Bantreil, X.; Nolan, S. P. *Nat. Protocols* **2011**, *6*, 69.

(8) Fulmer, G. R.; Miller, A. J. M.; Sherden, N. H.; Gottlieb, H. E.; Nudelman, A.; Stoltz, B. M.; Bercaw, J. E.; Goldberg, K. I. *Organometallics* **2010**, *29*, 2176.

(9) Becke, A. D. *Phys. Rev. A.: Mol., Opt. Phys.* **1988**, *38*, 3098.

(10) Perdew, J. P. *Phys. Rev. B.: Condens. Mater.* **1986**, *33*, 8822.

(11) Baerends, E. J.; Ziegler, T.; Autschbach, J.; Bashford, D.; Bérces, A.; Bickelhaupt, F. M.; Bo, C.; Boerrigter, P. M.; Cavallo, L.; Chong, D. P.; Deng, L.; Dickson, R. M.; Ellis, D. E.; van Faassen, M.; Fan, L.; Fischer, T. H.; Fonseca Guerra, C.; Franchini, M.; Ghysels, A.; Giammona, A.; van Gisbergen, S. J. A.; Götz, A. W.; Groeneveld, J. A.; Gritsenko, O. V.; Grüning, M.; Gusarov, S.; Harris, F. E.; van den Hoek, P.; Jacob, C. R.; Jacobsen, H.; Jensen, L.; Kaminski, J. W.; van Kessel, G.; Kootstra, F.; Kovalenko, A.; Krykunov, M. V.; van Lenthe, E.; McCormack, D. A.; Michalak, A.; Mitoraj, M.; Morton, S. M.; Neugebauer, J.; Nicu, V. P.; Noodleman, L.; Osinga, V. P.; Patchkovskii, S.; Pavanello, M.; Philipsen, P. H. T.; Post, D.; Pye, C. C.; Ravenek, W.; Rodríguez, J. I.; Ros, P.; Schipper, P. R. T.; Schreckenbach, G.; Seldenthuis, J. S.; Seth, M.; Snijders, J. G.; Solà, M.; Swart, M.; Swerhone, D.; te Velde, G.; Vernooijs, P.; Versluis, L.; Visscher, L.; Visser, O.; Wang, F.; Wesolowski, T. A.; van Wezenbeek, E. M.; Wiesenekker, G.; Wolff, S. K.; Woo, T. K.; Yakolev, A. L. *ADF2013.1* SCM, Theoretical Chemistry, Vrije Universiteit, Amsterdam, The Netherlands, 2013

(12) Fonseca Guerra, C.; Snijders, J. G.; te Velde, G.; Baerends, E. J. *Theor. Chem. Acc.* **1998**, *99*, 391.

(13) te Velde, G.; Bickelhaupt, F. M.; Baerends, E. J.; Fonseca Guerra, C.; van Gisbergen, S. J. A.; Snijders, J. G.; Ziegler, T. *J. Comput. Chem.* **2001**, *22*, 931.

(14) Baerends, E. J.; Ellis, D. E.; Ros, P. *Chem. Phys.* **1973**, *2*, 41.

(15) Hirshfeld, F. L. *Theoret. Chim. Acta* **1977**, *44*, 129.

(16) van Lenthe, E.; Baerends, E. J.; Snijders, J. G. *J. Chem. Phys.* **1994**, *101*, 9783.

(17) van Lenthe, E.; Baerends, E. J.; Snijders, J. G. *J. Chem. Phys.* **1993**, *99*, 4597.

(18) van Lenthe, E.; Ehlers, A.; Baerends, E.-J. *J. Chem. Phys.* **1999**, *110*, 8943.

(19) van Lenthe, E.; Snijders, J. G.; Baerends, E. J. *J. Chem. Phys.* **1996**, *105*, 6505.

(20) van Lenthe, E.; van Leeuwen, R.; Baerends, E. J.; Snijders, J. G. *Int. J. Quantum Chem.* **1996**, *57*, 281.

(21) Pye, C. C.; Ziegler, T. *Theor. Chem. Acc.* **1999**, *101*, 396.

(22) Morokuma, K. *J. Chem. Phys.* **1971**, *55*, 1236.

(23) Kitaura, K.; Morokuma, K. *Int. J. Quantum Chem.* **1976**, *10*, 325.

(24) Ziegler, T.; Rauk, A. *Theoret. Chim. Acta* **1977**, *46*, 1.

(25) Jacobsen, H.; Ziegler, T. *Comments Inorg. Chem.* **1995**, *17*, 301.

(26) Ziegler, T.; Rauk, A. *Inorg. Chem.* **1979**, *18*, 1558.

(27) Back, O.; Henry-Ellinger, M.; Martin, C. D.; Martin, D.; Bertrand, G. *Angew. Chem. Int. Ed.* **2013**, *52*, 2939.
